# The tree cover and temperature disparity in US urbanized areas: Quantifying the association with income across 5,723 communities

**DOI:** 10.1371/journal.pone.0249715

**Published:** 2021-04-28

**Authors:** Robert I. McDonald, Tanushree Biswas, Cedilla Sachar, Ian Housman, Timothy M. Boucher, Deborah Balk, David Nowak, Erica Spotswood, Charlotte K. Stanley, Stefan Leyk

**Affiliations:** 1 Center for Sustainability Science, The Nature Conservancy, Arlington, Virginia, United States of America; 2 California Program, The Nature Conservancy, Sacramento, California, United States of America; 3 CUNY Institute for Demographic Research and CUNY Graduate Center, City University of New York, New York, NY, United States of America; 4 Independent Researcher, Salt Lake City, Utah, United States of America; 5 Global Science Program, The Nature Conservancy, Arlington, Virginia, United States of America; 6 CUNY Institute for Demographic Research and Marxe School of International and Public Affairs, Baruch College, City University of New York, New York, New York, United States of America; 7 Northern Research Station, USDA Forest Service, Syracuse, New York, United States of America; 8 San Francisco Estuary Institute, Richmond, California, United States of America; 9 Geography Department, University of Colorado-Boulder, Boulder, Colorado, United States of America; 10 Institute of Behavioral Science, University of Colorado Boulder, Boulder, Colorado, United States of America; North Carolina State University, UNITED STATES

## Abstract

Urban tree cover provides benefits to human health and well-being, but previous studies suggest that tree cover is often inequitably distributed. Here, we use National Agriculture Imagery Program digital ortho photographs to survey the tree cover inequality for Census blocks in US large urbanized areas, home to 167 million people across 5,723 municipalities and other Census-designated places. We compared tree cover to summer land surface temperature, as measured using Landsat imagery. In 92% of the urbanized areas surveyed, low-income blocks have less tree cover than high-income blocks. On average, low-income blocks have 15.2% less tree cover and are 1.5⁰C hotter than high-income blocks. The greatest difference between low- and high-income blocks was found in urbanized areas in the Northeast of the United States, where low-income blocks in some urbanized areas have 30% less tree cover and are 4.0⁰C hotter. Even after controlling for population density and built-up intensity, the positive association between income and tree cover is significant, as is the positive association between proportion non-Hispanic white and tree cover. We estimate, after controlling for population density, that low-income blocks have 62 million fewer trees than high-income blocks, equal to a compensatory value of $56 billion ($1,349/person). An investment in tree planting and natural regeneration of $17.6 billion would be needed to close the tree cover disparity, benefitting 42 million people in low-income blocks.

## Introduction

An increasing number of studies, from a variety of disciplines such as ecology, economics, and environmental health, have found evidence that nature provides multiple benefits to human health and well-being [1–4]. This paper focuses on one urban natural feature, urban tree canopy cover (‘tree cover’ hereafter), the layer of leaves, branches, and stems of woody vegetation when viewed from above using remote sensing technology [5]. Tree cover provides a variety of ecosystem service benefits in cities [6, 7], including reducing air pollutant concentrations [8], mitigating stormwater runoff [7], maintaining water quality [9], encouraging physical recreation [10], and improving mental health [11]. In the United States, urban trees provide estimated annual benefits of about $18.3 billion in air pollution reduction, carbon sequestration, and lowered building energy use and power plant emissions [12]. Environmental health scientists have also found epidemiological evidence of a link between trees and human health. A recent review found that urban trees are associated with a wide range of benefits including: reduced harms such as ultraviolet radiation, air pollution-related respiratory conditions, and excess heat stress; greater restorative capacities such as cognition and attention restoration and benefits to mood and mental health; and positive health effects such as better birth outcomes, immune functioning, active living, cardiovascular function, weight status, and social cohesion [4]. Note that urban tree cover is also associated with some ecosystem disservices as well, both real and perceived [13].

This paper focuses on one benefit tree cover provides, reduced temperatures [8, 14–18]. Urban air temperatures are a function of the urban energy balance, which tree cover and many other factors influence [19]. Tree cover cools the air primarily by shading surfaces such as concrete and asphalt, thus preventing heat storage [19] and reducing the urban heat island effect. Tree cover can also reduce temperatures by transpiring water, increasing the fraction of heat going to latent rather than sensible heat [20]. Tree cover can reduce land surface temperature by 10–20°C on a summer day [20]. Effects on air temperatures are smaller, with a row of urban street trees on average lowering summertime air temperatures by 0.5–2.0°C [21]. This reduction in air temperature is nevertheless meaningful for human health. One study estimated that current urban tree cover in the United States saves 1200 lives annually during heatwaves and provides annual heat-reduction services worth $5.3–12.1 billion [22].

Despite the recognition of the numerous benefits provided by tree cover, research suggests it is unequally distributed in many cities. A meta-analysis of the literature shows that low-income neighborhoods [23] and minority communities [24] often have less tree cover. These patterns have been found both within the United States [25] and in other nations [26]. For instance, Schwarz and colleagues studied 7 incorporated cities and found that neighborhoods with lower median household income have less tree canopy cover than neighborhoods with higher income in the same city [27]. Nesbit and colleagues found that across 10 incorporated cities, neighborhoods with lower education and income had less vegetation, a pattern that was most strongly apparent in large cities [28]. Riley and Gardiner studied 9 incorporated cities, examining tree cover and ecosystem service provision, which related to socioeconomic variables in different ways in each city [29]. A similar general trend for inequality in exposure to nature has been observed with remotely sensed measures of greenness [30], which have been used to analyze vegetation in cities globally [e.g., 31], and with exposure to heat risk due to lack of trees and high impervious cover [32, 33].

Thus, research suggests tree cover is often but not always inequitably distributed in cities, which may have significant implications for ecosystem service provision and human well-being [23, 24]. The statistical interpretation of the bivariate negative association between tree cover and a socio-economic variable like income or race/ethnicity is complex for at least two reasons. First, tree cover is also associated with other factors, including differences in climate, biome, neighborhood age, urban density, the intensity of urban settlement, and other aspects of urban form [34–40]. In some studies, covariates such as terrain, housing type and density explained part of the variation between tree cover and socio-economic variables [25, 29, 39, 41, 42]. Furthermore, socio-economic variables are themselves also correlated with aspects of urban form, such as the degree of sprawl and population density [43, 44]. Second, some studies have found that the statistical significance of the relationship between tree cover and socioeconomic variables is different if spatial autocorrelation is accounted for [27, 29], and one review study observed that studies that controlled for spatial autocorrelation generally found fewer statistically significant trends [23]. Therefore, one important question when quantifying patterns in tree inequality is whether relationships with socio-economic variables like income and race/ethnicity are statistically significant after accounting for covariates and spatial autocorrelation.

Our principal goal for this study is to analyze tree cover and temperature inequality for a large sample of thousands of communities throughout the United States. To date, however, such a large survey has been hampered by the lack of fine-resolution data on urban tree cover with national coverage. For example, the National Land Cover Data (NLCD) Tree Canopy Cover datasets (2011 and 2016) provide a measure of change in tree cover over five years across the US but their 30m resolution has been found to systematically underestimate urban tree cover because small patches of tree canopy are often not detected [45], particularly in landscapes below 30% tree cover and above 70% cover [46]. Urban tree canopy (UTC) assessments, which provide fine-resolution data on tree cover [47], are available in many jurisdictions, including entire states [e.g., 5, 48]. These UTC assessments are the gold standard for accuracy, often using local training datasets to create classified tree cover maps from Lidar and high-resolution (often < 1m) imagery [e.g., 49–51]. However, UTC assessments are not available for all communities in the United States, and therefore could not be used to achieve our study’s goal of analyzing tree cover inequality for a large sample of thousands of communities throughout the United States.

Here, we analyze the 100 largest urbanized areas in the United States, housing 167 million people (nearly 55% of the total US population) and containing the urbanized portions of 5,723 incorporated places (cities and towns) and census-designated places in 2010 (see Methods for definitions of these terms). We focus on tree cover inequality relative to income, hypothesizing that high-income neighborhoods will have greater urban tree canopy cover. While it is less of a focus, we also examine tree cover relative to race/ethnicity inequality, hypothesizing that neighborhoods with a higher proportion of non-Hispanic whites will have greater tree cover. Key goals of this study include:

- Quantify for a large sample of US communities the degree to which urban tree cover varies with household income and population density.
- Map for a large sample of US communities the inequality in tree cover and summer temperature across and within US urbanized areas.
- Test if urban tree cover will be lower in blocks of lower household income even after accounting for how population density and other covariates that affect the correlation between income and tree cover.
- Quantify for a large sample of US communities the difference in summer surface temperatures between low and high-income blocks.
- Estimate for a large sample of US communities the US urban tree cover disparity, the amount of tree planting that would be required to raise low-income blocks up to the median of similarly dense high-income blocks.

## Materials and methods

Our methods proceeded in five stages: defining the study area; remote sensing and image interpretation; assembling demographic and socioeconomic data; spatial overlay operations in a geographical information system (GIS) to integrate these data sets; and statistical analysis to determine significant patterns in tree cover.

### Study area

Our study area was all US urbanized areas [52] larger than 500 km^2^, where “urbanized area” follows the definition of the US Census for the 2010 census [52]: “an urban area will comprise a densely settled core of census tracts and/or census blocks that meet minimum population density requirements, along with adjacent territory containing non-residential urban land uses as well as territory with low population density included to link outlying densely settled territory with the densely settled core.” There were 100 urbanized areas larger than 500 km^2^, with a population of 167 million people. Note that one urbanized area generally contained multiple communities: these 100 urbanized areas contained the urbanized portion of 3,520 incorporated places (e.g., municipalities) and 2,291 census-designated places (places not legally incorporated but with commonly used place names that have been mapped by the Census Bureau) [53].

In this study, tree cover is compared to demographic characteristics derived from US Census data available at the block group level (income) or block level (all other demographic and social variables).

### Remote sensing

In order to assess tree cover inequality for a large sample of thousands of communities throughout the United States, we mapped tree cover at 2m resolution. Our study explored the application of Google Earth Engine (GEE), a web-based application programming interface (API) that enables rapid analysis of large spatial datasets [54] to develop a fast, systematic method to map tree cover across large urbanized areas within the US.

The urbanized areas were grouped into ten geographically defined groups predominantly determined by their biomes (S1 Table), as defined by Olson and colleagues [55]. The justification behind this was that vegetation type, and hence spectral characteristics, are similar within each biome, allowing optimization of image classification procedures regionally. There were two exceptions to this assignment of urbanized areas to biome. First, the temperate broadleaf and mixed forests biome of the eastern United States contains many urbanized areas, and we decided to split it into subgroups to allow for more locally defined classification algorithms (S1 Fig). Generally, the assignment of urbanized areas in this biome to subgroups followed ecoregional boundaries, with adjustments to avoid having a small amount of urbanized area in any one subgroup. Second, we chose to place western urbanized areas into one group, rather than assuming urbanized areas in the western United States would be spectrally similar to urbanized areas in the eastern United States of the same biome. While that does mean western urbanized areas span a range of biome types, it allows for the classifier to optimize for the urban form of cities along the West Coast, which is different than East Coast cities. In practice, since the random forest algorithm used (see below) is quite flexible, the exact geographical groupings used in our classification do not appear to affect our results substantially, and preliminary classifications using different geographic groupings yielded qualitatively similar tree cover maps.

The analysis was based on 2m imagery from the US Department of Agriculture’s National Agriculture Imagery Program (NAIP) available on Google Earth Engine (GEE). The NAIP archive between 2014 and 2016 had four spectral bands—blue (B), green (G), red (R), and near-infrared (NIR) wavelengths—and was filtered for cloud-free images. This time period was chosen because there was near universal coverage of NAIP images with these four bands, whereas earlier time periods lack complete coverage. From these four bands, we derived six additional layers useful for image classification. Three of these were commonly-used indices, normalized band ratios that are helpful for detecting vegetation and water: Normalized Difference Vegetation Index (NDVI) [56], Green Normalized Difference Vegetation Index (GNDVI) [57], and Normalized Difference Water Index (NDWI) [58]. These were defined as:

$$NDVI=\frac{\left(NIR-R\right)}{\left(NIR+R\right)}$$ (1)

$$GNDVI=\frac{\left(NIR-G\right)}{\left(NIR+G\right)}$$ (2)

$$NDWI=\frac{\left(G-NIR\right)}{\left(G+NIR\right)}$$ (3)

To differentiate trees from other green areas such as pasture, baseball fields or golf courses, we used an entropy function in GEE (image.glcm) that calculated the gray-level co-occurrence matrix (GLCM) value [59], using as input the NDVI in a 4x4 kernel window. Finally, a binary layer was created where pixels with high texture value and high NDVI threshold were given a value of 1, and the remaining pixels were otherwise coded as 0. Thresholds for texture and NDVI varied by geographic region. Thresholds were chosen to correctly identify forested sites in the training data as having high NVDI and a high texture value, while avoiding coding areas of grass as 1. The final ten band image stack (four bands from NAIP imagery, plus six derived layers) was used in image classification.

Training data for this image classification come from a spatially explicit dataset comprising of 9,424 control points, created by Nowak and Greenfield [34]. In the Nowak and Greenfield analysis, each control point in 2014 had a known land cover assigned by a human image interpreter looking at aerial photographs. For this analysis, we simplified the Nowak and Greenfield land cover classes to tree (N = 3746) or not tree (N = 5678). In our analysis, each control point also has values in each of the ten-band image stack. This information was used to train a Random Forests (RF) classification algorithm [60, 61]. The RF classifier in GEE was run separately for each different geographic area, outputting a classified tree/non-tree image as well as information on the confusion matrix and statistics of classification accuracy using the classifier.confusionMatrix function in GEE.

#### Validation against an independent, high accuracy dataset

Our unit of analysis for this study was the census block, so we were primarily interested in making sure our tree cover estimates at that scale were accurate. We validated our method for estimating tree cover against an independent test dataset, some of the Urban Tree Canopy (UTC) assessments conducted by the US Forest Service. The goal was to validate our methodology against urban tree canopy assessments that are known to have high accuracy, to ensure our estimates of tree cover at the census block level are accurate. UTC assessments provide fine-resolution data on urban tree cover, and typically map forest cover at < 1m resolution, often using Lidar information and local training datasets [47]. Our mapping effort will not be as accurate at the UTC datasets at the pixel-level, since we do not have access to such detailed local information for all 5,723 communities in our study area. By validating census-block level tree cover estimates against this independent, high accuracy data set, we checked to make sure our estimates of forest cover at the census block level were accurate.

We obtained 34 UTC tree cover layers [5] that were available on the UTC website (http://gis.w3.uvm.edu/utc/Landcover/) in October 2019 that were contained within our study extent. Assessed areas range from small municipalities (e.g. Takoma Park, MD) to large counties (e.g., Fairfax County, VA), and are primarily in the forested and grassland biomes of the United States. Note that these are not all the available UTC layers, some of which are available from different websites and span larger geographies. For our purposes, however, this sample of UTC layers was sufficient to validate our block level tree cover estimates for forested and grassland biomes, where the majority of US urban areas lie. We calculated the UTC tree cover in census blocks (N = 155,916 census blocks) and compared it to our estimates. We tested how well our tree cover area estimates correlated with the UTC area estimates using simple linear regression, at the census block level. We also calculated the median absolute error of our block level estimates of tree cover, compared to that of the UTC estimates.

### Demographic/Socioeconomic and built-up intensity data

Based on a review of the literature [23–32], we selected covariates that have been shown in other studies to be related to urban tree canopy. Covariates also had to be available nationally at a consistent spatial and temporal scale, as well as applicable in all cities in our study area. This precluded including some covariates that have been shown to be regionally important but do not have consistent spatial data over our entire study area (e.g., information on redlined areas). The covariates we included in our study fit into two broad categories: Demographic data and information on the fraction of area built up.

Our source for demographic data was primarily the US Decennial Census (2010), downloaded from National Historic Geographic Information System (NHGIS) [62]. Our general strategy was to use the finest spatial resolution demographic data possible, and to conduct our analysis at this level where possible. We used block level data for population from the US decennial census, the finest resolution publicly available data. Specifically, we used variable TotPop in the NHGIS naming system (NHGIS variables names noted hereafter parenthetically). We acknowledge that this predates our tree cover map (2014–2016), and in some areas with large demographic changes between 2010 and 2014–2016, this may introduce a bias. However, we chose this approach because we wanted to use the most spatially detailed demographic data, which is only available for 2010. Moreover, the NAIP imagery in the period around 2010 was of lesser quality, having fewer spectral bands, so we chose not to try to map tree cover around 2010.

Total population was divided by the land area (Aland10, available at the census block level) to calculate the population density within each census block. Our hypothesis was that population density would be negatively correlated with tree cover. While we recognize that block scale population density is not the only attribute describing urban form that is related to tree canopy cover, several studies have shown evidence of a decay in tree cover as population density increases [35, 37, 63, 64]. Population density is also an easily accessible metric at broad spatial scales that is both correlated with and related to other aspects of urban form such as building type, compactness, and average block size [65], and some of these factors have also been shown to be related to tree cover [35, 36, 42].

Census data on the number of non-Hispanic white residents (NoHiWhite, available at the census block level) was used to calculate the proportion of residents that were non-Hispanic white, which we hypothesized would be positively correlated with tree cover. We also included in our analysis the median age of residents (MedAge, available at the census block level), since we expected that elderly residents are at greater risk for some diseases, such as negative health impacts during heat waves.

Per-capita income (IncPr) was taken from the most recent American Community Survey (ACS) of the Census Bureau (2013–2017 estimates), available at the census block group level. We used this source of information on income, rather than the 2010 decennial census data, to have income data that was as close as possible in time to our tree cover imagery (2016). In a small fraction of cases where block group level estimates were indicated to have low reliability (having a coefficient of variation larger than 34%), the more reliable tract level estimates were used [66]. Because block group boundaries of the ACS did not always align with the block boundaries of the decennial census, we linked to the block-level data using the centroids of the census blocks. All blocks within the same block group or tract, respectively, shared the same estimate of income. Our hypothesis was that per-capita income would be positively correlated with tree cover.

Additionally, we included in our analysis data on the built-up intensity (BUI), a ratio of indoor floor area of all buildings in a grid cell to the area of that grid cell (250m resolution). The BUI layer is part of the Historical Settlement Data Compilation of the United States (HISDAC-US) which includes public domain time series of data on the built environment in the conterminous US derived from Zillow’s ZTRAX database [67]. This information allows us to account for commercial and industrial areas that may have a small residential population but large built areas, which we hypothesized would lead to lower levels of tree cover.

### Geoprocessing and spatial overlay

Our tree cover maps were overlain in ArcGIS 10.4.1 with the census blocks, to calculate the tree cover area within each census block using the zonal statistics tool, which calculates summary statistics within spatial zones (e.g., the census block). Although information from the census is used to define urbanized areas, the boundaries of the census blocks did not precisely align with the boundaries of the urbanized areas. Thus, we included in our analysis only those census blocks whose entire area was inside the urbanized area, buffered out an additional 100m. This small buffer zone accounted for slight non-alignment of census block boundaries with the urbanized area boundaries. We also excluded from further calculations census blocks with no people in them. When making maps, we used base layers from the Natural Earth data library (https://www.naturalearthdata.com/).

#### Surface temperature

We were interested in having another response variable. We chose to examine land surface temperature (hereafter “surface temperature”), which is easily quantifiable from satellite imagery. High-resolution information on surface temperature was not available for the thousands of communities examined in this study. We chose to use Landsat-derived surface temperature, which is at 30m resolution. The unit of statistical analysis for our study (see below) was the census block, and there were multiple Landsat pixels within census blocks.

Median summer surface temperature for images between June 21—September 22 from 2000 to 2019 was calculated using Landsat thermal data. We chose to use this 20-year median summer surface temperature to average over the high temporal variability in surface temperatures, because exploratory analyses with shorter time intervals yielded spatially variable results based upon individual seasonal weather events. While a shorter time interval centered around 2016 (our date for tree cover mapping) would potentially allow for a more temporally accurate estimation of surface temperature for 30m pixels that experienced a land use or land cover change from 2000–2019, we found that shorter time intervals decreased the precision in our estimates of surface temperature and obscured spatial patterns in surface temperature.

Landsat 5 and Landsat 8 Thermal Infrared Sensor (TIRS) data within the Tier 1 Surface Reflectance collections [68] were accessed through Google Earth Engine [54]. Landsat 5 B6 and Landsat 8 B10 provides surface temperature in degrees Kelvin. In order to avoid contamination by clouds and cloud shadows, masking was performed using the pre-computed quality assurance bands that are part of the Tier 1 products. These masks were created using the cFmask algorithm [69]. Any pixel identified as bit 5 (cloud) or 2 (cloud shadow) within the "pixel_qa" band was omitted from the analysis. The final median summer temperature was calculated from the remaining values. Temperature data over surface water was masked out using the European Commission, Joint Research Centre’s version 1.1 2016 yearly historical surface water product [70]. Any area identified as permanent or seasonal water was omitted from the analysis. For more details on our surface temperature analysis, please see our code in GitHub (see Acknowledgements).

### Statistical analysis

Our statistical analysis proceeded in four steps. First, we conducted descriptive analyses, focusing on the spatial patterns of tree cover and temperature inequality, as well as the bivariate relationship of these variables with income. Second, we conducted a statistical analysis of trends among urbanized areas, after accounting for the effect of covariates and spatial autocorrelation. Third, we conducted a statistical analysis of trends within urbanized areas, after accounting for the effect of covariates and spatial autocorrelation. Finally, we calculated the tree disparity with respect to income, using information on this bivariate relationship to estimate the number of trees that would have to be planted to bring tree cover in low-income neighborhoods up to the level of tree cover in equivalently dense high-income neighborhoods.

#### Descriptive analysis

The unit of analysis for our study was the census block. Census blocks are the smallest geographic unit for which demographic data is available from the US Census Bureau, and in cities often correspond to individual city blocks bounded by streets. By using this fine spatial grain, our analysis focused in on the tree cover near people’s homes. Tree cover near homes is important since most studies of the impact of nature on human health find statistical associations most frequently when the greenness is within a few hundred meters of people’s homes. For instance, James and colleagues found a 12% lower all-cause mortality rate for women with more greenness within 250m of their home [71].

We divided census blocks into quartiles by per-capita income, defined within an urbanized area. This allowed the thresholds for being in a particular income quartile to vary by urbanized area, accounting for the large differences in income among urbanized areas of the United States. We divided census blocks into four groups by population density, using consistent ranges of population density for all urbanized areas (0–2000 people/km^2^, 2000–4000 people/km^2^, 4000–8000 people/km^2^, and > 8000 people/km^2^). These ranges were chosen to approximately divide census blocks in our study area into evenly sized groups, but with rounded break points that were easily understood. We then calculated the population-weighted median tree cover (%), by income and population density category, within each urbanized area. The population-weighted median was chosen for our analysis because our analysis focus was the average tree cover experienced by people near their home, which is what has been found relevant for human well-being and health [22]. Note that for many cities, most tree cover is in large parks where few or no people live, and most people live in neighborhoods with relatively little tree cover. In this situation, the population-weighted median is less than the mean tree cover across the entire city.

Examination of the distribution of proportion non-Hispanic white showed a distinctive bimodal distribution (i.e., most blocks are predominately white or predominately non-white, with relatively few blocks in between). Accordingly, we created a binary categorical variable, which is coded as 1 if the block is majority non-Hispanic white and 0 otherwise.

#### Analysis among urbanized areas

For this analysis, the unit of analysis is the urbanized area (N = 100). We conducted two regressions of patterns among urbanized areas. The first regression predicted median tree cover at the urbanized area level. The second regression predicted the difference in median tree cover between income quartiles (top quartile minus bottom quartile). Potential explanatory variables considered were all measured at the urbanized area level, and included: median per-capita income, variation (interquartile range) of per-capita income, median population density, variation (interquartile range) of population density, and biome (a categorical variable that describes major climate and ecological gradients [55]).

Population density was log transformed to improve normality. Tree cover was arcsine-transformed to improve normality. Arcsine transformation is a common transformation of ecological proportion data [72]. While it has been occasionally criticized as less interpretable for proportion data than the logit transformation [73], we chose to use it because tree cover often has extreme values (0’s or 1’s) that are undefined for the logit transformation but are defined for the arcsine transformation. Forward selection using AIC was used to select the variables to be included in our two regressions.

Analysis of variograms of model residuals suggested moderate spatial autocorrelation between urbanized areas closer than 500km, and the spatial autocorrelation was found to be statistically significant using Moran’s I for both regressions (P < 0.05). Accordingly, we tested for the appropriate model structure using the Lagrange Multiplier Tests, using the lm.morantest function in the spdep library in R [74]. Results indicated a spatial lag SAR model was most appropriate for both regressions, which we used for all among urbanized area regression results reported in this manuscript.

#### Analysis within urbanized areas

For this second statistical analysis, each urbanized area was analyzed separately. The unit of analysis is the census block (N = 1,938,386 total across all urbanized areas). As census blocks are contiguous with one another, and tree cover was highly spatially autocorrelated, it was necessary to account for any potential spatial autocorrelation. Tree cover was arcsine transformed to improve normality. The explanatory variables used in this within urbanized areas analysis were population density category, income quartile, BUI category, income category, and majority non-Hispanic white status. These variables were chosen for inclusion in the regression because they were correlated to the response variable and were only moderately colinear with one another, reducing problems with multicollinearity during parameter estimation and significance testing. To test for spatial autocorrelation, we first ran a simple linear regression that predicted tree cover from our explanatory variables (Proc Mixed in SAS 9.4), and then analyzed the spatial autocorrelation in the residuals (Proc Variogram), by urbanized area. Spatial autocorrelation was always significant at scales less than 1km, and for some urbanized areas was significant out to scales of 2km. To address this spatial autocorrelation and appropriately account for it in our estimation of regression parameters, we conducted a block bootstrap [75] using the R package ‘sperrorest’ [76]. Block bootstraps of 2km were drawn from each urbanized area 1000 times, and for each bootstrap a regression was calculated. The distribution of bootstrapped parameters was approximately normal, and we assessed a parameter statistically significant within an urbanized area if at least 95% of the time its value was different than zero.

#### Tree disparity with respect to income

One of the goals of this research paper was to estimate the US urban tree cover disparity, which we defined as the amount of tree planting that would be required to raise low-income blocks up to the median level of tree cover in high-income blocks in the same population density category. The intent in this calculation was not to statistically model the impact of all covariates (e.g., race/ethnicity, age, BUI), which we did in a different analysis step (see subsection “Analysis within urbanized area”). Rather, our intent was to provide a descriptive, policy-relevant measure of the disparity between low- and high-income blocks of similar urban form. We acknowledge that this is a hypothetical calculation of the amount of trees that would be required to close this urban tree cover disparity, and that it may not be feasible or desirable to entirely close this disparity, given competing land-uses and community preferences in some locations.

In order to calculate tree disparity, for each urbanized area we took the estimates of tree cover (%) in each population density and income category, and converted these estimates to the difference in tree cover area by multiplying by the area of the census block. We then estimated the number of adult trees required to fill this tree cover area, using a factor of 19.6 m^2^ of canopy cover per urban tree, which was derived from Nowak and Greenfield [12], who estimated that there were 5.5 billion urban trees in 2010 over 67.6 million acres of urban area that is on average 39.3% tree cover. To estimate the cost of planting new saplings to close the tree cover disparity, we used a factor of $283/stem, which was derived from the review of tree planting costs in multiple US cities by Krueger et al. [8]. To estimate the lost value to people represented by the current US urban tree cover disparity, we calculated the compensatory value, using the average value per stem reported by Nowak [77]. Compensatory value is one way to estimate the total value of a tree and represents what compensation should be paid to the tree’s owner if the tree is lost [77]. While we acknowledge there are other ways to value the benefits provided by trees [78], we included the compensatory value in our analysis to show that differences in tree cover between low- and high-income blocks amounted to a substantial difference in the financial value of this amenity available.

## Results

### Tree cover classification algorithm

**Fig 1:**
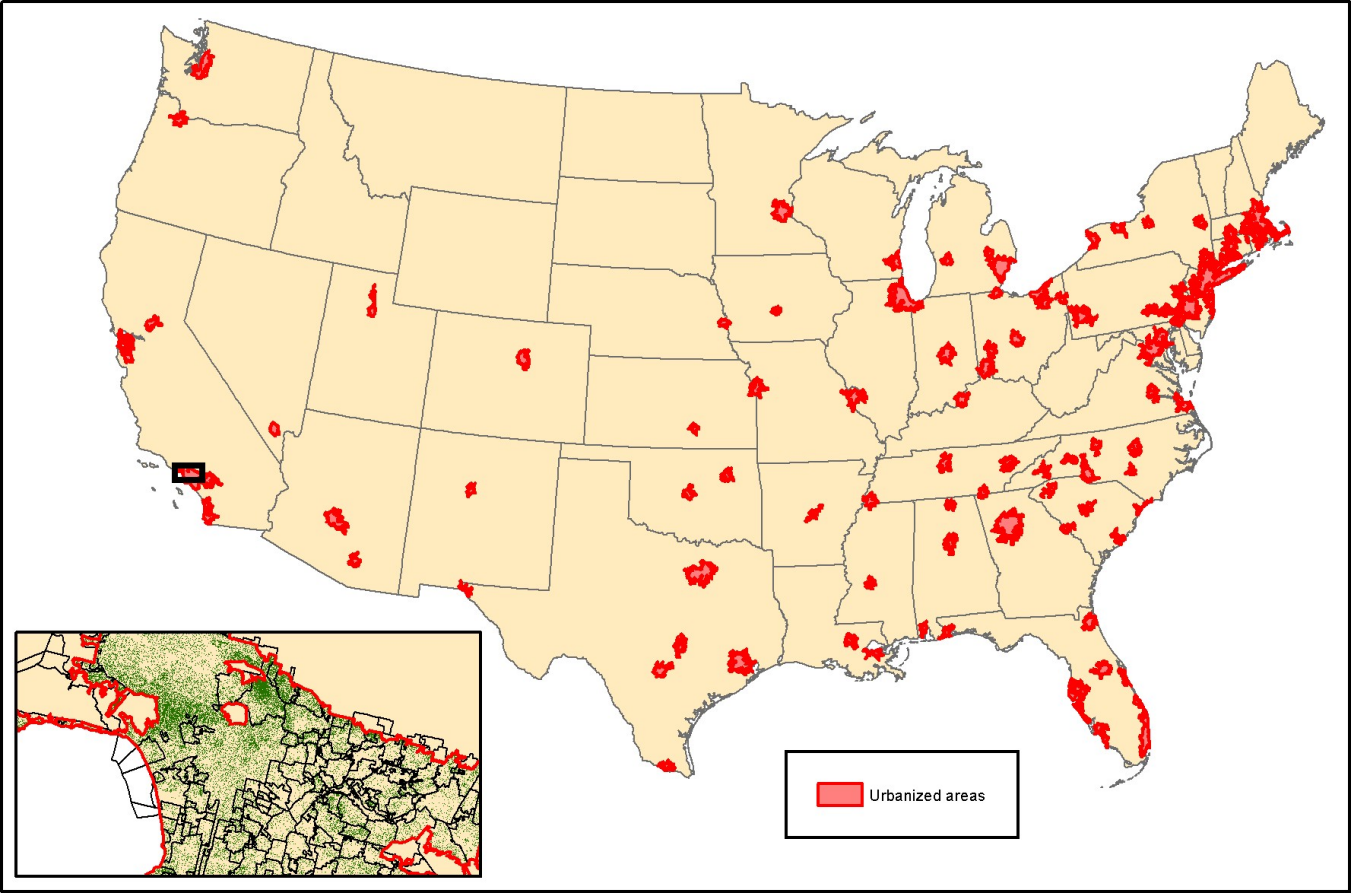
Urbanized areas mapped in this study. Tree cover was mapped for 2016. An inset map of the tree cover (green) of a portion of the Los Angeles urbanized area is shown. Within the area mapped in the inset, there were 93 municipalities and 34 census-designated places, whose outlines are shown with black lines.

Our classification method successfully mapped tree cover in the 100 largest urbanized areas in the United States (Fig 1), which cover a total area of 158,000 km^2^ or 39.6 billion pixels at 2m spatial resolution. To document the results of the random forest algorithm, pixel-level classification accuracy is shown in S1 Table, but note that we did not use pixel-level data in our analysis of tree cover disparities, but instead used aggregated results at the census block level. Pixel-level classification accuracy was greatest in forested biomes and lower accuracy in biomes like deserts and grasslands. Across all urbanized areas, average user’s accuracy of the forest class was 82.1%. The average kappa statistic was 0.45, which would be rated by Landis and Koch [79] as moderate accuracy. The kappa statistic is widely used in remote sensing, but see citation [80] for a discussion of its limitations.

### Tree cover validation against an independent dataset

Our unit of analysis for this paper was the census block level, and it was thus our goal to consistently map tree cover accurately at the census block level. Census blocks contain many 2m pixels, and if classification errors are relatively spatially uncorrelated then the estimate of tree cover at the census block level can be more accurate than at the pixel-level scale. To assess the accuracy of our forest cover estimates at the block level, we validated our estimates of tree cover at the census block level against an independent dataset, the accurate high-resolution tree cover maps produced by Urban Tree Canopy (UTC) assessments [47]. At the block level, our estimates of tree cover were highly linearly correlated with those of the UTCs (R = 0.97, S2 Fig). On average, the median absolute block-level error of our tree cover estimate, as compared against the gold standard estimates of the UTCs, was 6.0%. Thus, while the pixel-level accuracy of our classification was only moderate, our estimates of tree cover at the block-level were highly correlated with an independent validation dataset.

In much of our analysis of tree cover disparities, we analyzed aggregated information on tree cover relative to income, looking across many urbanized areas to come up with national estimates. To assess our accuracy at this scale, we calculated an estimate of median tree cover by income category across the entire validation dataset, using both our tree cover datasets and the UTC dataset. Median errors were small in income quartiles 1 (2.8%), 2 (3.4%), and income quartile 3 (3.0%) but were slightly larger in income quartile 4 (5.7%). In all income quartiles, the estimate of tree cover was slightly greater in the UTC dataset than in our tree cover dataset. Overall median error for the whole validation dataset was 3.6%.

### Tree cover, income and population density

**Fig 2:**
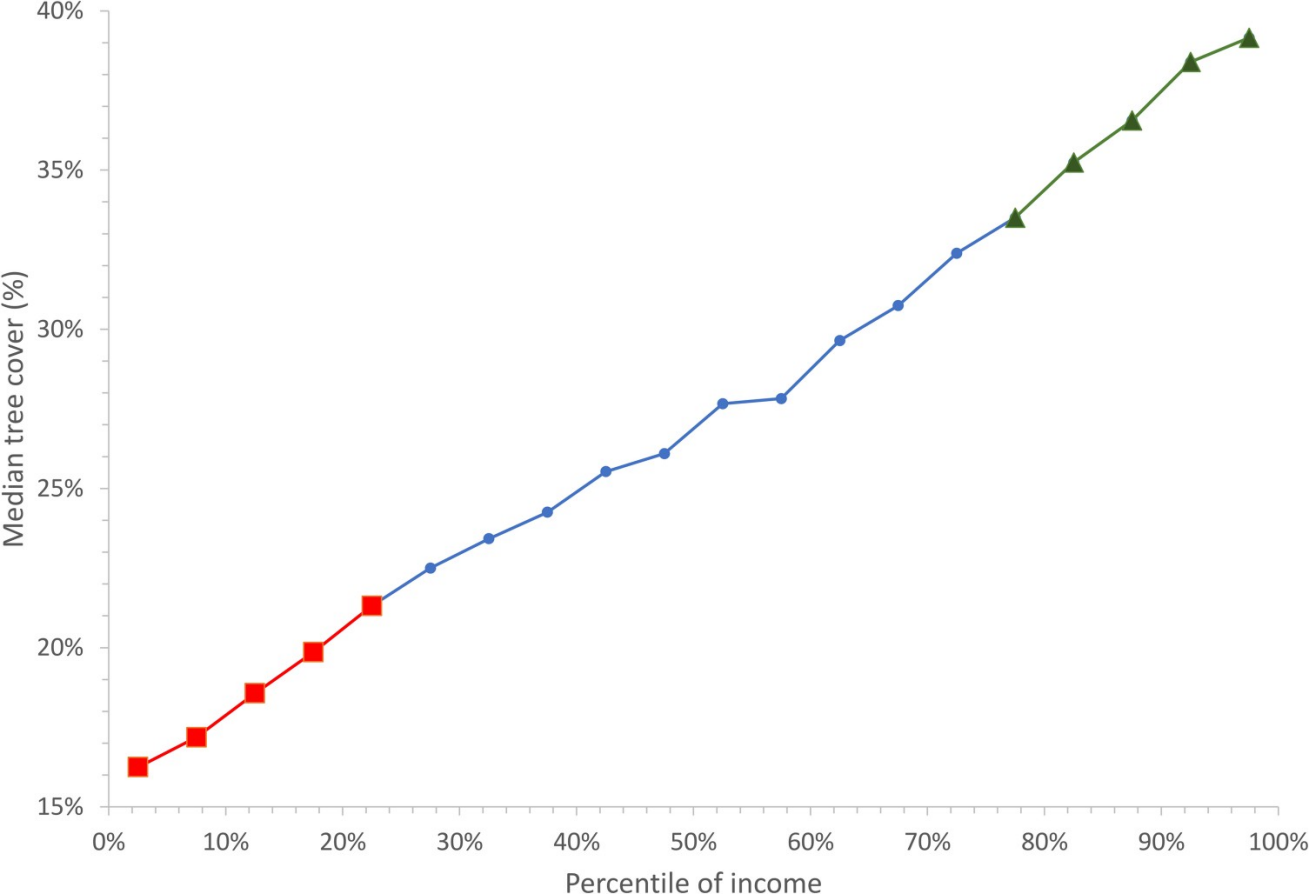
Tree cover as a function of income. We classified census blocks based upon the income distribution within its urbanized area, calculating the percentile of the income distribution that block has. For ease of display, blocks were grouped into income categories (0–5%, 5–10%, etc.) and the population-weighted median tree cover (%) across the entire study area calculated. This paper focuses most of its analysis on the lowest quartile (red squares) and highest quartile (green triangles) of income.

Income was inversely associated with tree cover (Fig 2). On average, an increase in relative income of 5% (for example, from the 15^th^ to the 20^th^ percentile) increases tree cover by 1.2%. The relationship between tree cover and relative income appears to be approximately linear. Within each urbanized area, we divided the census blocks into quartiles based upon the per-capita income distribution. Averaging across all urbanized areas, the least affluent quartile of census blocks had a median tree cover of 19.7%, while the most affluent quartile had a median tree cover of 34.9% (Table 1). Clearly, low- and high-income blocks nationwide had very different levels of tree cover, and thus likely different levels of ecosystem service (or disservice) provision.

**Table 1:** Median tree cover, by population density and income categories.

	Percent Tree cover		Population in category, millions		Area in category, km^2^	
Population density (people/km^2^)	Low-income	High-income	Low-income	High-income	Low-income	High-income
Very low (<2000)	40.0%	47.5%	8.1	23.0	12,528	40,220
Low (2000–4000)	26.7%	28.8%	10.4	10.3	3,656	3,853
Moderate (4000–8000)	17.6%	19.5%	9.9	3.6	1,816	691
High (>8000)	11.4%	10.5%	13.3	4.6	905	271
***Across all blocks***	19.7%	34.9%	41.7	41.5	18,906	45,035

Tree cover was compared between low and high-income quartiles, over 5,723 incorporated towns and other places in the US. Statistical significance of these trends is discussed in the section of the manuscript entitled “Evaluating Statistical Significance.”

The relationship between income and tree cover was partially explained by population density (Table 1). Population density is of course only one aspect of urban form that could affect urban tree canopy distribution but is a commonly measured summary statistic that we examined relative to tree cover. In the census blocks in the least affluent quartile, the majority (56%) of the population lived in blocks in the high (4000–8000 people/km^2^) or highest population density (>8000 people/km^2^) categories, typified by multi-unit buildings. In contrast, in the census blocks in the most affluent quartile, the majority (55%) of the population lived in blocks in the lowest (< 2000 people/km^2^) population density category, typified by single family homes on large lots. Tree cover was inversely associated with population density (Table 1), presumably because there was simply less area to fit trees into the landscape when a large fraction of the area is devoted to buildings and impervious surfaces. However, even when stratifying by population density, income of a census block was still associated with tree cover. In most population density categories, tree cover was greater in the most affluent income quartile than in the least affluent. Interestingly, the difference in tree cover between low- and high-income census blocks was greatest in the lowest population density category, and less in the high or highest population density categories. In other words, inequality in tree cover appeared greater in the exurbs and far suburbs—areas with greater potentially vegetated area—than in denser blocks.

### Patterns across the United States

**Fig 3:**
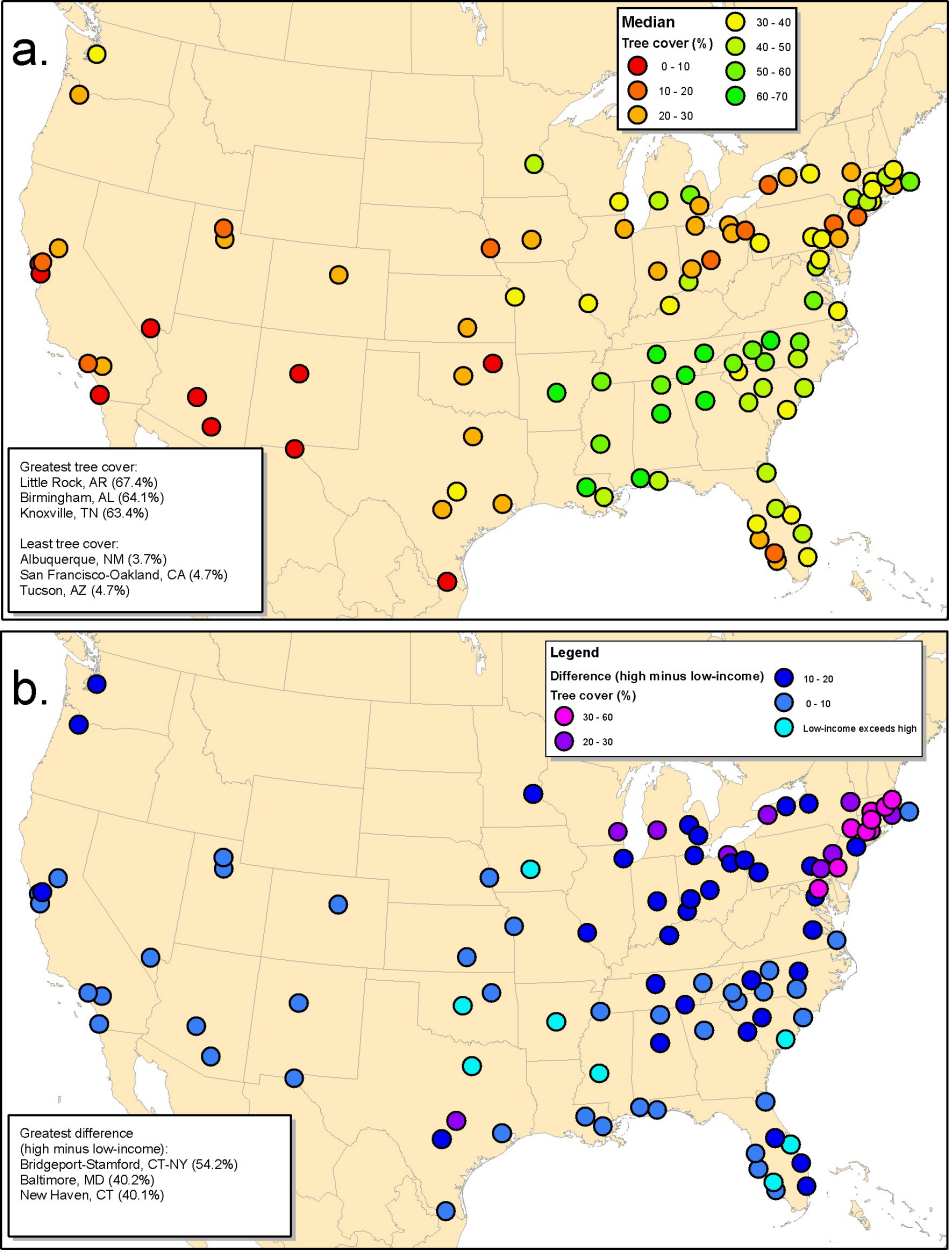
Tree cover for United States large urbanized areas. a.) Population-weighted median tree cover. b.) The absolute difference between low-income blocks (lowest quartile of income) and high-income blocks (highest quartile of income) in tree cover.

Patterns in tree cover across US urbanized areas are shown in Fig 3. Median tree cover (Fig 3A) was greatest in urbanized areas in the South like Atlanta and lowest in urbanized areas in the Southwest like Phoenix. Much of this regional pattern in median tree cover was explained by biome, with urbanized areas in forested biomes having more tree cover than those in other biomes such as desert or grassland (urbanized area level regression, S2 Table, P = 0.005). This pattern has been found in other studies as well [12], and is expected as tree cover is the natural state in forested biomes, as opposed to being intentionally planted in other biomes. Another important variable in explaining variation in tree cover was population density, with urbanized areas with higher median population density (e.g., urbanized areas in the Northeast such as New York City and Philadelphia) having lower tree cover, presumably because there was simply less area to fit trees into more densely developed areas (P = 0.0002). After accounting for these other variables, median per-capita income in an urbanized area was not a significant predictor of median tree cover in that urbanized area (P = 0.41). Median tree cover for all 5,723 incorporated places (cities and towns) and census-designated places surveyed in this paper can be found in S3 Table.

We quantified for each urbanized area the difference in tree cover between low and high-income blocks (Fig 3B). We found that a trend toward greater tree cover in high-income blocks occurred in 92% of the urbanized areas we examined (S4 Table). The greatest difference in tree cover between low- and high-income blocks was found in urbanized areas in the Northeast of the United States, particularly along the coast between Washington, DC and Boston, MA (Fig 3B). The urbanized area with the greatest difference in tree cover between low- and high-income blocks was the Stamford/Bridgeport, CT area (54%). Conversely, the West and Southwest had generally smaller differences in tree cover between low- and high-income blocks, likely because of the generally lower tree cover in those biomes. In a few urbanized areas, primarily in the South (e.g., Cape Coral, FL, Jackson MS), median tree cover was greater in low-income blocks than in high income areas (see Discussion section for description of why this trend may have occurred).

The strongest predictor of the difference in tree cover between high- and low-income blocks was income inequality (urbanized area level regression, S2 Table, P < 0.0001). Urbanized areas with greater income inequality (as measured by the interquartile range) had greater differences in tree cover between high- and low-income blocks. Urbanized areas with a greater range of population densities (as measured by the interquartile range) had greater differences in tree cover between high- and low-income blocks (P = 0.0006). Finally, urbanized areas with a greater median tree cover had smaller differences in tree cover between high- and low-income blocks (P = 0.005). After accounting for these other variables, biome was not a statistically significant predictor of the difference in tree cover between high- and low-income blocks (P = 0.77).

### Patterns within urbanized areas

**Fig 4:**
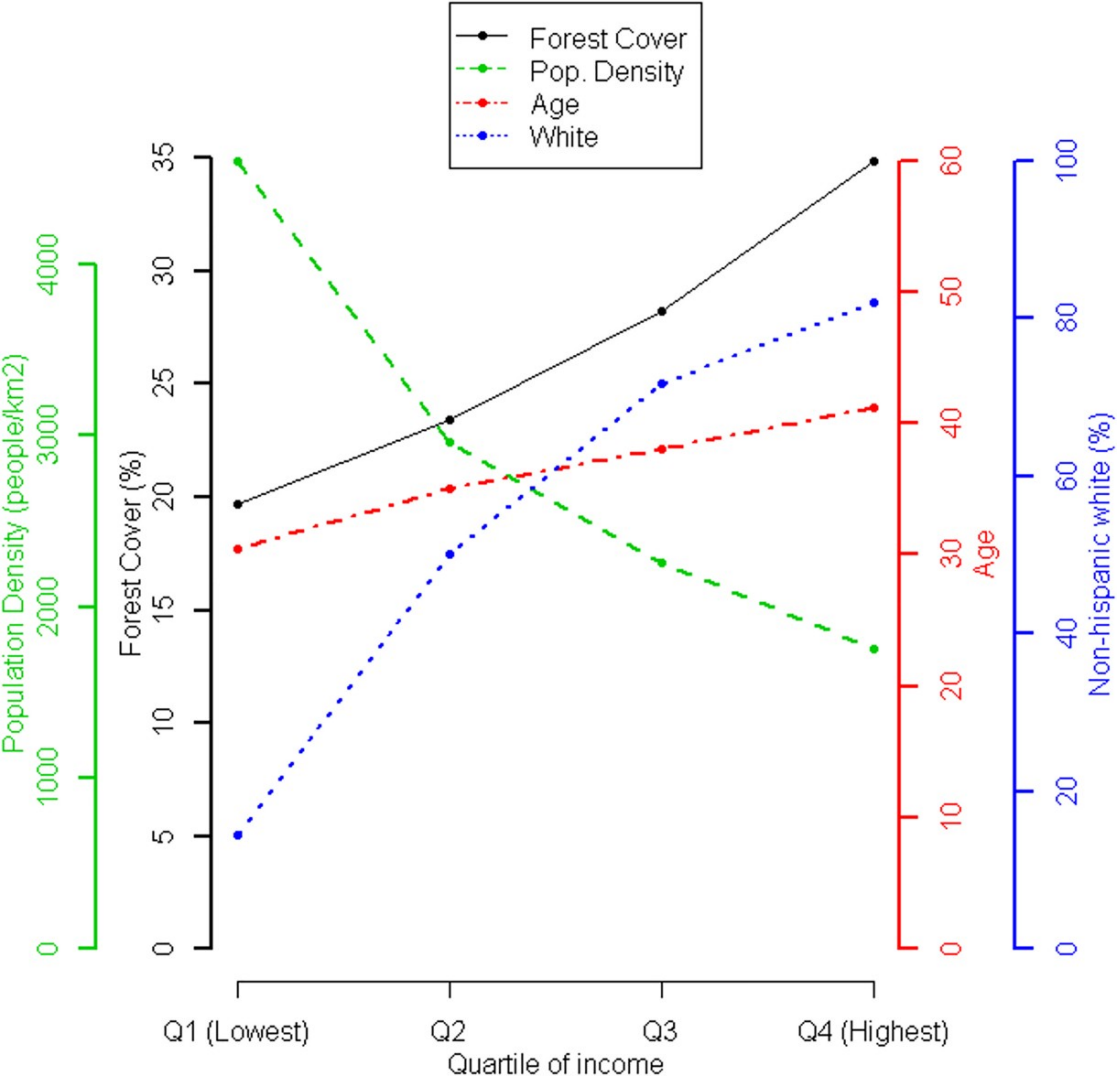
Patterns across blocks of different incomes. Census blocks within each urbanized area were classified by the quartile of income they occupy. For each quartile, we show the median tree cover, population density, age, and percent non-Hispanic white.

Patterns for individual cities can be visualized online. Generally, the highest population densities can be found in the center of an urbanized area [c.f., 81], with average income and average tree cover that were lower than the averages for the entire urbanized area. More suburban or exurban areas, often in separate municipalities or census-designated places, had lower population density, higher average income, and higher average tree cover. In other words, the spatial transition from suburban to urban core related to a gradient in both tree cover and income, a pattern that can be expected for many US urbanized areas [82]. Many other variables correlated with income and thus show similar associations with tree cover (Fig 4). More affluent census blocks had a greater proportion of non-Hispanic whites and generally had more tree cover. In contrast, blocks with a lower proportion of non-Hispanic whites generally had less tree cover. Residents of more affluent blocks also tended to be older than residents of less affluent blocks, and the median age of residents was positively associated with tree cover.

### Evaluating statistical significance

We assessed for each city separately whether income or race were significantly related to tree cover, after accounting for covariates (population density, income, built-up intensity, and race/ethnicity) and spatial autocorrelation using a block bootstrap approach. This block bootstrap approach allows an estimate of the confidence interval of regression parameter after accounting for the spatial autocorrelation, with a parameter considered significant if they are statistically different than zero.

Population density was the covariate most commonly statistically significant in our regressions (S5 Table), with the highest population density category having significantly less tree cover than the lowest population density category in 94 urbanized areas. Income was the second most common statistically significant effect (S5 Table), with the highest income category having significantly more tree cover than the lowest income category in 78 urbanized areas. Similarly, majority non-Hispanic white blocks had significantly more tree cover than did majority non-white blocks in 67 urbanized areas. Finally, we included in our statistical analysis the built up intensity (BUI), a ratio of indoor floor area of all buildings in an area to the land area. BUI was negatively related to tree cover and was statistically significant in 68 urbanized area.

From our statistical analysis, we concluded that even after accounting for spatial autocorrelation among census blocks and covariates like population density and BUI, income and race/ethnicity had statistically significant relationships with tree cover in 78% and 67% of the urbanized areas, respectively.

### Surface temperature differences

**Fig 5:**
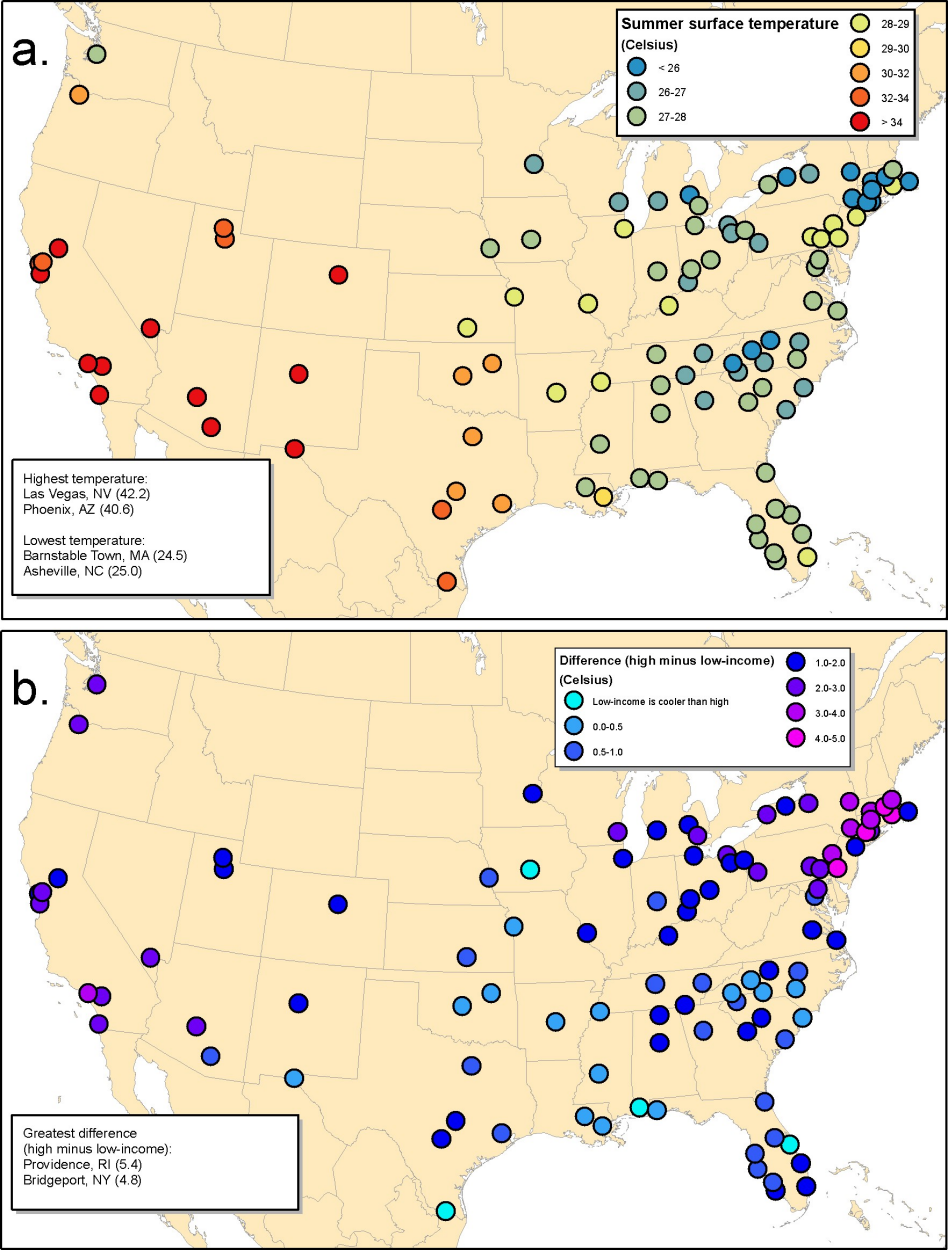
Surface temperature for United States large urbanized areas. a.) Population-weighted median summer surface temperature. b.) The absolute difference in summer surface temperature between low-income blocks (lowest quartile of income) and high-income blocks (highest quartile of income).

Not surprisingly, summer surface temperatures were highest in cities in arid biomes in the western United States (Fig 5A). Averaged across our study area, the least affluent quartile of census blocks had a summer surface temperature that was 1.5⁰C hotter than the most affluent quartile of census blocks. The variation among urbanized areas was large, however, with the greatest difference in summer surface temperature between low- and high-income blocks being 5.4⁰C in the Providence urbanized area (Fig 5B, S4 Table). The difference in summer surface temperature between low- and high-income blocks was highly correlated to the tree cover difference (R = 0.82, S3 Fig). The greatest differences in summer surface temperature between low- and high-income blocks were in the Northeast, where, in addition to Providence, three other urbanized areas had differences of more than 4.0⁰C: Bridgeport/Stamford, Worcester, and Philadelphia. Another 9 urbanized areas, including Boston, had differences greater than 3.0⁰C but less than 4.0⁰C.

### Tree cover disparity

Since tree cover was correlated with population density differences, we chose to examine the difference in tree cover between low- and high-income blocks after accounting for population density difference. Within a given population density category, we calculated the disparity in tree cover between low- and high-income blocks (Table 2). Across all urbanized areas, 1,217 km^2^ of new tree cover would hypothetically be needed. S6 Table shows these numbers broken down by urbanized area and population density category. The Seattle urbanized area had the largest disparity in tree cover in areal terms (74 km^2^), followed by the Chicago (54 km^2^) and Detroit (54 km^2^) urbanized areas.

**Table 2:** The tree cover disparity.

	Population density category (people/km^2^)				
	*Very Low (<2000)*	*Low (2000–4000)*	*Medium (4000–8000)*	*High (>8000)*	Total across all population density classes
**Total area (km2) of low-income census blocks**	12,528	3,656	1,816	905	18,906
**Total population of low-income census blocks**	8,120,985	10,431,696	9,863,540	13,326,870	41,743,091
**Tree cover disparity (%)**	9.8%	3.7%	2.6%	3.3%	4.9%
**Needed tree cover to close disparity (km**^**2**^**)**	985	171	50	11	1,217
**Trees required to close disparity**	50,367,030	8,762,004	2,547,062	575,105	62,251,201
**Compensatory value, billions (median)**	$45.5	$7.9	$2.3	$0.5	$56.3
**Compensatory value, billions (low)**	$32.9	$5.7	$1.7	$0.4	$40.7
**Compensatory value, billions (high)**	$91.3	$15.9	$4.6	$1.0	$112.8
**Cost of planting new saplings to close disparity, billions**	$14.3	$2.5	$0.7	$0.2	$17.6

Shown is the tree disparity between low- and high-income categories after accounting for population density. Compensatory values shown are the disparity in tree cover value in low- and high-income categories, in billions of USD2019. Compensatory value is the compensation to owners for loss of a tree and can be viewed as one way to value the tree as an asset.

We estimated that the least affluent quartile of blocks had 62 million fewer trees in it than the most affluent quartile (Table 2). This tree cover disparity, even after controlling for population density, meant low-income blocks had $56 billion (range: $41–113 billion) less in compensatory value. This is an average of $1,349/person less in compensatory value.

We estimated the cost of tree planting of saplings and their maintenance to close the urban tree cover disparity (Table 2). In total, approximately $18 billion in tree planting would be needed to close this tree cover disparity. Note that this figure was less than the compensatory value, because trees are assets that appreciate over time; after planting a tree sapling increases in compensatory value as it grows. Note also that our estimate assumed that all new trees are planted, but there is potential that land-use changes (e.g., stopping mowing) could allow for increased natural regeneration. Because natural regeneration is often free or very low cost, that would be a more economical way to increase urban tree canopy on some sites.

The vast majority of needed adult trees to close the tree cover disparity would be in the Very Low population density category (Table 2). This happened because low-income blocks in the Very Low population density category covered a large area (12,528 km^2^) and there was a large difference in tree cover between low-income and high-income blocks (9.8%), leading to an estimated 50 million trees needed to close the tree cover disparity in this population density category. However, relatively few people lived in low-income blocks in the Very Low population density category. Potentially, for biomes that allow natural forest regeneration, land-use management strategies that encourage natural regeneration may be more economical for this category than tree planting.

## Discussion

Our research shows that inequality in tree cover between low- and high-income blocks was widespread in the US, occurring in 92% of the urbanized areas we studied (N = 5,723 cities and other places). On average, there were 15.2% more tree cover in high-income blocks than in low-income blocks (Table 1). Our results are consistent with other studies [23–32] that show tree cover inequality relative to income for many but not all cities.

The Stamford/Bridgeport, CT area had the greatest difference in tree cover between low- and high-income blocks (54%). This example illustrates why it may be insightful to analyze inequality in tree cover across an urbanized area, in addition to analyzing trends within specific municipalities. Stamford and Bridgeport are considered one urbanized area by the US Census Bureau, and workers commonly commute between them. However, the two municipalities have very different histories. Bridgeport developed as an industrial town, where workers lived in densely populated areas near factories [83]; in the 1970s and 1980s the economy suffered as the industrial sector declined. In contrast, Stamford developed at least in part as a place for summer vacation homes for New York City residents [84], and currently is economically well-off. Today, Bridgeport’s tree cover is 20.2% and Stamford’s is 37.1%. While there are differences in tree cover within each of these two municipalities, much of the difference in tree cover between low- and high-income blocks in the entire urbanized area comes from differences between these two municipalities. Overall, our results suggest that prior studies that examined tree cover within particular municipalities [e.g., 27, 28, 33] may find a smaller difference in tree cover between low-income and high income blocks than would exist across an entire urbanized area, because US urbanized areas are subdivided into jurisdictions that often have different income distributions.

For the 100 urbanized areas we studied, we found that inequality in tree cover was correlated with differences in the average population density found in low- and high-income blocks, consistent with other studies in the literature that have discussed demographic patterns relative to tree cover patterns [85, 86]. As in other studies [e.g., 23, 27, 29], accounting for covariates and spatial autocorrelation reduced the strength of the association between income and tree cover, but income was still significantly associated with tree cover in 78% of the urbanized areas studied. Note that while population density was negatively correlated with tree cover, this correlation was not perfect, and at any given population density class there were blocks that have relatively high tree cover and others with relatively low tree cover. Accordingly, it was possible to find dense blocks that still had relatively high tree cover.

Note that there were 22% of urbanized areas where there was not a statistically significant relationship between income and tree cover. Eight percent of the 100 urbanized areas studied had median tree cover that was actually greater in low-income blocks than in high income areas. This occurred in urbanized areas with many blocks, both low-income and high-income, in the Very Low population density category. It is possible that there was still variation in population density within this Very Low population density category. For instance, in the Cape Coral urbanized area, there were many blocks in the Very Low population density category in both Naples (a relatively affluent community near the coast) and Lehigh Acres (a less affluent community more inland), but the latter had relatively greater tree cover and lower population density, presumably because the lower population density allowed larger lot sizes that are more forested. Another 14% of the 100 urbanized areas studied had tree cover that was greater in high-income areas than in low-income areas, but the trend was fully explained after accounting for covariates and spatial autocorrelation. These urbanized areas also tended to have most of their blocks in the Very Low population density category. For instance, Houston, a very low population density city, had 8.1% more tree cover in high-income than in low-income neighborhoods (S4 Table) but this trend was not statistically significant after accounting for covariates and spatial autocorrelation (S5 Table).

There are two main potential causes of this pervasive inequality in tree cover between low- and high-income blocks. First, differences among blocks in tree cover in the public right of way along roads and in other publicly owned land could be due to differential investments by municipal or local governments. Commonly, in the urban core with high-density blocks, a greater fraction of the land area is in public ownership, either as parks [87] or as part of the road right of way [88], as compared to lower density blocks in the suburbs or in rural areas. Thus, tree cover in dense blocks is particularly shaped by the actions of municipal governments. The fragmentation of US urbanized areas into many different municipalities [89], with different levels of economic development and hence different levels of potential government spending [90], worsens inequality in tree cover at the scale of the entire urbanized area. Low-income households are often clustered in low-income municipalities that have fewer resources to plant and maintain trees on public land, and so these municipalities could end up with fewer trees than their high-income counterparts. Even within one municipality, there can be differential public-sector investment in trees in low-income neighborhoods [91]. Sometimes low-income households may be less likely to participate in voluntary tree planting programs [92]. Historically, there have also been instances of explicit racism in urban planning and zoning decisions, such as the practice of redlining (refusing home loans or insurance to specific, generally minority, neighborhoods). Studies have showed that areas that were redlined have higher surface temperature [93–95], presumably in part because they have lower tree cover.

Second, differences among census blocks in tree cover on privately-owned land could be because of differential land-use decisions by private landowners, as well as differential investments by landowners in tree planting and maintenance. In incorporated places at the edges of urbanized areas, a greater fraction of the plantable (non-impervious) land area is under private ownership than in higher density blocks in the largest municipality of an urbanized area [96]. Thus, tree cover in the suburbs is particularly shaped by the actions of private landowners [38], and factors such as social pressure, power, and prestige all influence the maintenance of trees in private spaces [38]. It is interesting that we found that inequality in tree cover between low- and high-income blocks was greatest in lower density blocks that were located predominantly in the suburbs. This trend may occur simply because there was greater average tree cover in lower density blocks, and hence more potential for inequality in forest cover. However, another possible explanation for the greater inequality in tree cover in the suburbs is the relatively greater importance of actions on private land. It may be that low-income households may be less able to afford the cost, in money and time, of planting trees and maintaining them. Moreover, low-income households are more likely to be in rental units [97] and are thus less involved in making decisions about land management, while owners are primarily interested in reducing maintenance costs and thus may have less of an incentive to plant and maintain trees than the unit’s residents.

Regardless of the cause of inequality in tree cover, the tree cover disparity between low- and high-income blocks potentially has health implications. That is, whatever its historical causes, the fact that currently some neighborhoods have lower tree cover has potential effects on the health of those who live there. Our results showed a large tree cover disparity (Table 1) and surface temperature differential (S4 Table) between low- and high-income blocks. The average surface temperature differential was 1.5⁰C, but in more than a dozen cities the differential exceeded 3⁰C. Note that differences in temperature of the magnitude found in this study can be meaningful to human health. For instance, Anderson and Bell [98] found that in US urbanized areas heat wave mortality risk increased by 2.5% for each 0.6⁰C (1⁰F) increase in air temperature. While our measurement of summer average surface temperature is different than air temperature during heat waves, as measured by Anderson and Bell [98], surface temperatures and air temperatures are correlated [99], which suggests that the difference in surface temperatures shown in our study may have meaningful implications for human health [22, 98].

Finally, our research suggests that tree planting to close the tree cover disparity between low- and high-income blocks is possible. A targeted investment in tree planting of $15.8 billion would close the urban tree cover disparity for 34 million people in low-income blocks of moderate or greater population density, although it would likely take at least 5–10 years for planted trees to be large enough to deliver significant ecosystem service benefits. Some of the needed tree planting would occur through public sector investment in tree planting and maintenance on the public right of way and publicly owned land. This greater investment could be more strategically applied if government agencies responsible for urban forestry actively partnered with agencies responsible for public health, to allow for jointly planning where to plant to maximize benefits to human well-being [100]. But some of the needed tree planting would have to occur on private land, which would require incentives or regulations that motivate the private sector to conduct this tree planting. There are several such programs in existence, such as tree protection ordinances, green area ratios in planning codes, and incentives for tree planting from electric utilities. Of course, any such local programs must consider the historical causes and consequences of tree inequality in that community, and work with local stakeholders to design a tree planting program that meets local needs. Regardless of how these local programs operate, our results show that inequality in tree cover is widespread and pervasive in US urbanized areas and deserves policy attention.

## Supporting information

S1 Fig Groups of urbanized areas used for remote sensing image classification. See Methods section for details of how groups were defined.
(TIF)

S2 Fig Tree cover comparison to UTC. The tree cover area maps for this study (y-axis) was highly correlated (R = 0.97) at the census block level with the 1m tree cover area maps developed as part of the Urban Tree Canopy (UTC) assessments program (x-axis). The best-fit regression line is shown in blue (F = 2545715, df = 1, R^2^ = 0.94, P < 0.001). The slope (1.069) of this regression was significantly different than the 1:1 line (red), with our estimates of tree cover area at the census block level thus being slightly less that of the UTC estimates of tree cover area.
(TIF)

S3 Fig Tree cover difference versus temperature difference. For the urbanized areas in the study, the relationship between tree cover difference (in %, high income minus low-income areas) versus temperature difference (in Celsius, high income minus low-income quartile).
(TIF)

S1 Table Accuracy assessment statistics at the pixel-level of our classification. Shown are overall accuracy, producer’s accuracy for the tree cover class, and user’s accuracy for the tree cover class. Also shown is the kappa statistic. Note that the goal of our study was to produce accurate estimates of tree cover at the census block level, where our accuracy was greater (see text for details).
(DOCX)

S2 Table Urbanized area level regression results. We conducted two regressions of patterns among urbanized areas (N = 100). The first regression predicted median tree cover, corresponding with the data shown in Fig 3A. The second regression predicted the difference in median tree cover for income quartiles (top quartile minus bottom quartile), corresponding with the data shown in Fig 3B. In both cases tree cover was arcsine transformed to improve normality.
(DOCX)

S3 Table Population-weighted median tree cover for 5,723 incorporated places and census designated places. Data is sorted alphabetically by the name of the urbanized area and then the name of the place. We used the population-weighted median tree cover to estimate how much tree cover an average person had within their census block. Note that many incorporated places had high tree cover in large parks with relatively low population in them, which means that in these places the population-weighted median tree cover was generally less than the simple arithmetical mean.
(DOCX)

S4 Table Summary statistics of percent tree cover by urbanized area. Table is sorted by the interquartile range, so urbanized areas with the greatest tree cover gap between low- and high-income census blocks are on top. Also shown is the surface temperature difference between low- and high-income blocks.
(DOCX)

S5 Table Within urbanized area regression coefficients. For each urbanized area, we separately ran a regression predicting tree cover (arcsin transformed) as a function of population density category, income category, built-up intensity (BUI) category, and majority white status. Our statistical analysis included census blocks in all four income categories (quantiles, defined within each urbanized area). In this table we show the effect on tree cover of the highest category in each group relative to the baseline (the lowest category). For instance, for Akron, OH the highest population density category had 0.30 fewer units of tree cover (arcsine transformed) than the lowest population density category. Significant effects (one-tailed, P<0.05) are shown with an asterisk. Note that for some urbanized areas, there were no census blocks in the highest BUI category. At the median tree cover nationally (27%) a 0.1, 0.2, and 0.3 increase in arcsine transformed tree cover equaled an increase of 9.3%,19.1%, and 29.1% in tree cover, respectively.
(DOCX)

S6 Table Summary statistics of percent tree cover within cities. Shown are data stratified by low- and high-income blocks, by population density class, by urbanized area. Also shown is an estimate of the tree cover gap (%) between low- and high-income blocks, and the needed tree cover to close the gap.
(DOCX)
