# Supplementary figures and images for: The tree cover and temperature disparity in US urbanized areas: Quantifying the association with income across 5,723 communities
Source: PLoS One. 2021 Apr 28;16(4):e0249715. doi: 10.1371/journal.pone.0249715 (PMC8081227; doi:10.1371/journal.pone.0249715)

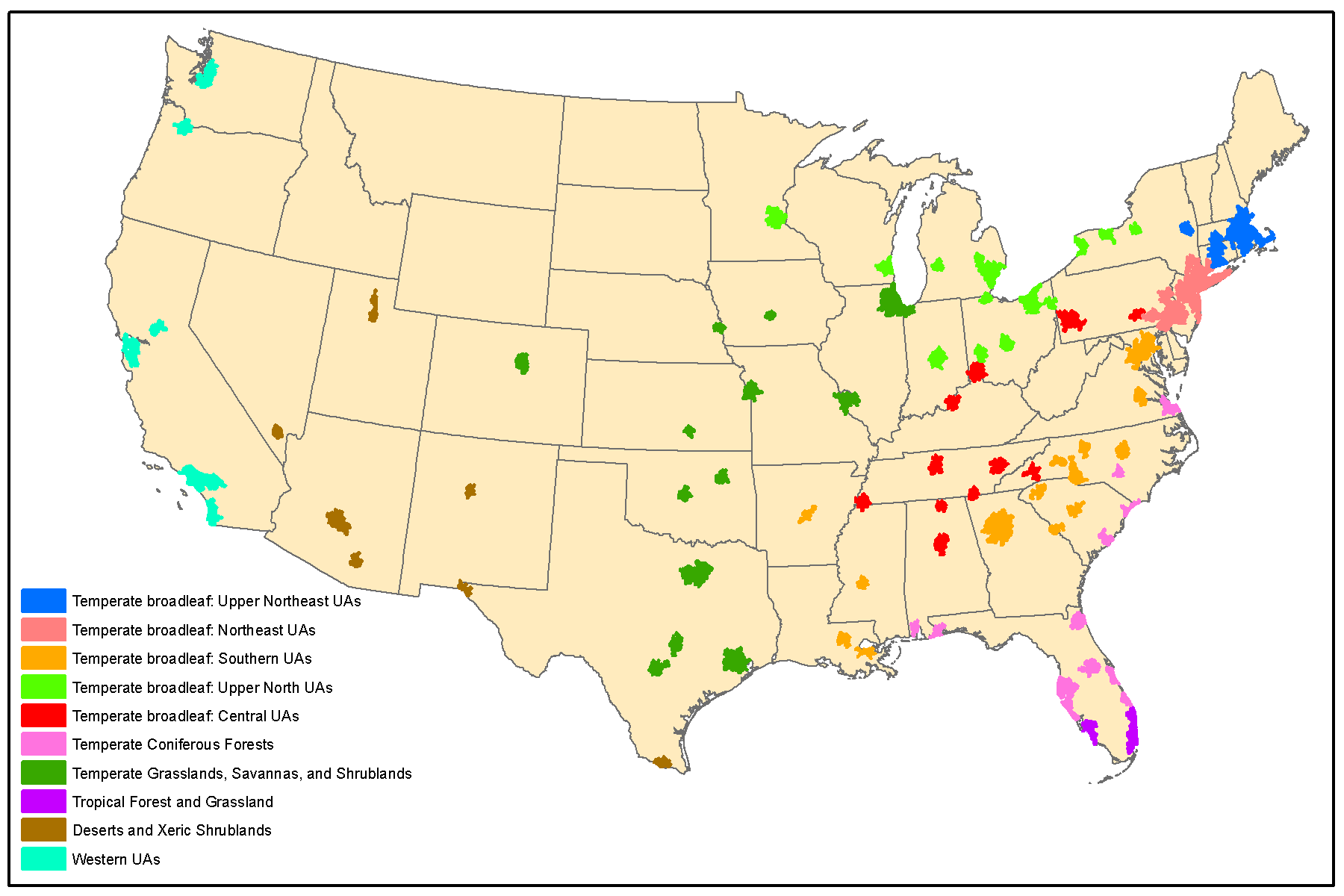

Supplement: S1 Fig — See Methods section for details of how groups were defined. (TIF) [file pone.0249715.s001.tif]

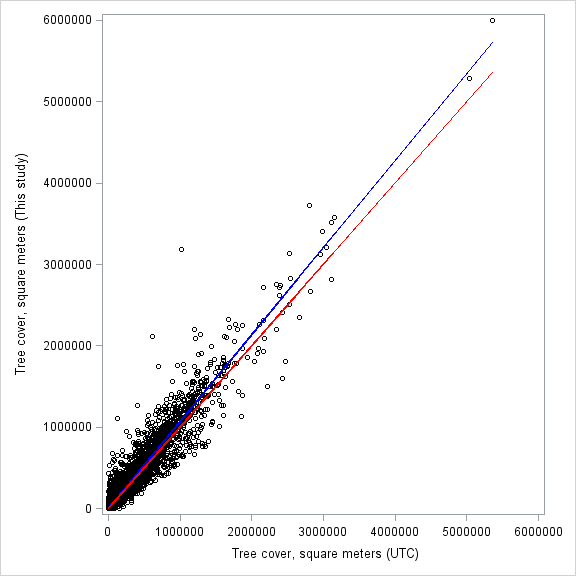

Supplement: S2 Fig — The tree cover area maps for this study (y-axis) was highly correlated (R = 0.97) at the census block level with the 1m tree cover area maps developed as part of the Urban Tree Canopy (UTC) assessments program (x-axis). The best-fit regression line is shown in blue (F = 2545715, df = 1, R2 = 0.94, P < 0.001). The slope (1.069) of this regression was significantly different than the 1:1 line (red), with our estimates of tree cover area at the census block level thus being slightly less that of the UTC estimates of tree cover area. (TIF) [file pone.0249715.s002.tif]

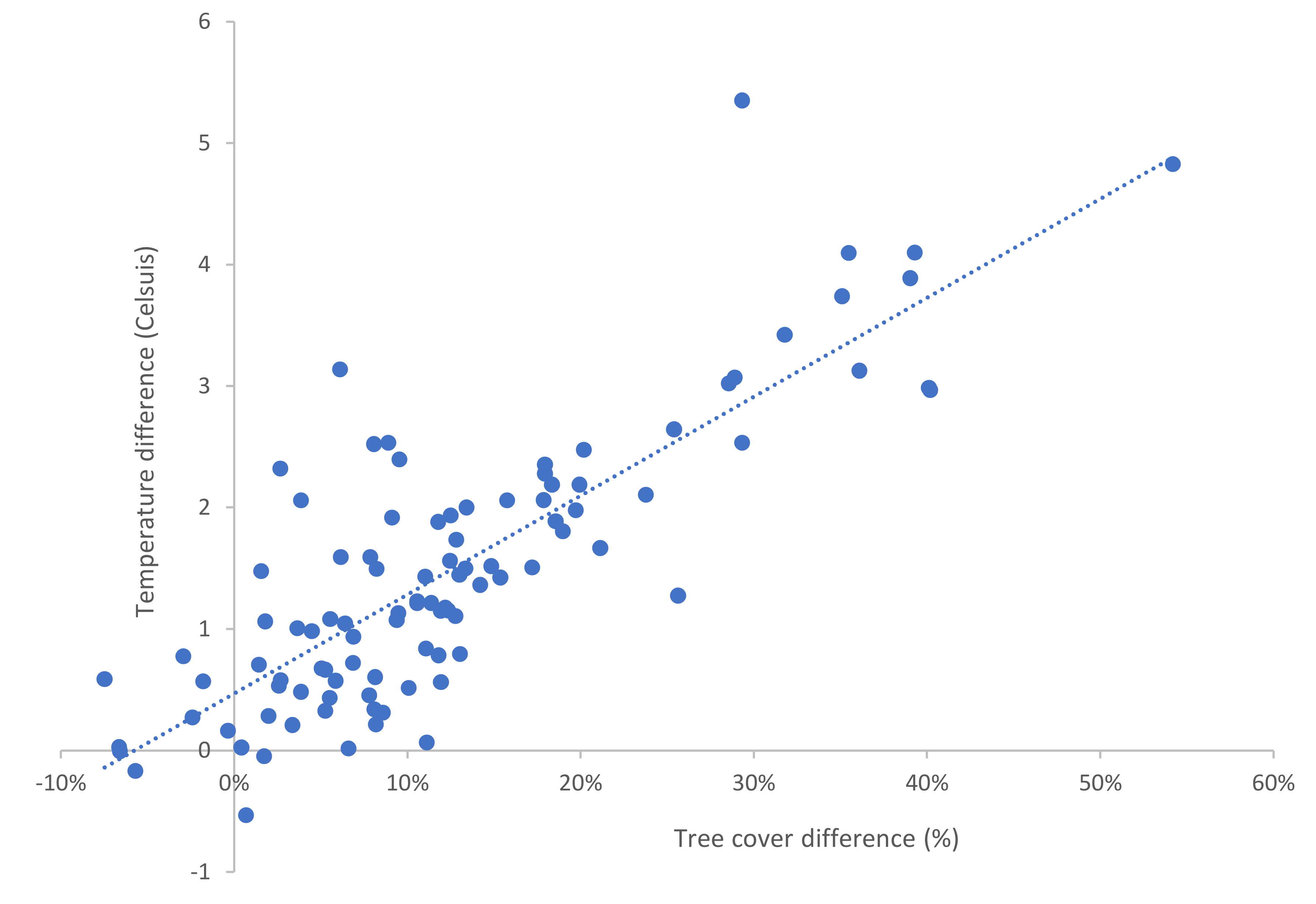

Supplement: S3 Fig — For the urbanized areas in the study, the relationship between tree cover difference (in %, high income minus low-income areas) versus temperature difference (in Celsius, high income minus low-income quartile). (TIF) [file pone.0249715.s003.tif]
