# Supplementary material for: The tree cover and temperature disparity in US urbanized areas: Quantifying the association with income across 5,723 communities
Source: PLoS One. 2021 Apr 28;16(4):e0249715. doi: 10.1371/journal.pone.0249715 (PMC8081227; doi:10.1371/journal.pone.0249715)
Supplement: S1 Table — Shown are overall accuracy, producer’s accuracy for the tree cover class, and user’s accuracy for the tree cover class. Also shown is the kappa statistic. Note that the goal of our study was to produce accurate estimates of tree cover at the census block level, where our accuracy was greater (see text for details). (DOCX) [file pone.0249715.s004.docx]

| **Biome/Region** | **Number of urbanized areas (UAs)** | **Overall Accuracy** | **Producer's Accuracy for Forest** | **User's Accuracy for Forest** | **Kappa statistic** |
| --- | --- | --- | --- | --- | --- |
| Temperate broadleaf and mixed forests |  |  |  |  |  |
| *Upper Northeast UAs* | 8 | 81.8% | 84.3% | 92.4% | 0.49 |
| *Northeast UAs* | 6 | 78.1% | 79.8% | 88.6% | 0.50 |
| *Southern UAs* | 15 | 79.6% | 70.6% | 87.2% | 0.52 |
| *Upper North UAs* | 15 | 73.5% | 73.5% | 84.3% | 0.44 |
| *Central UAs* | 11 | 73.4% | 76.2% | 83.5% | 0.41 |
| Temperate coniferous forests | 12 | 79.9% | 81.6% | 86.6% | 0.57 |
| Temperate grasslands, savannas and shrublands | 13 | 72.9% | 72.9% | 76.2% | 0.46 |
| Tropical and subtropical grasslands, savannas and shrublands | 3 | 73.6% | 79.5% | 66.0% | 0.48 |
| Deserts and xeric shrublands | 8 | 65.3% | 64.6% | 55.4% | 0.29 |
| Western UAs (Mediterranean and forest biomes) | 9 | 71.4% | 74.0% | 86.8% | 0.31 |
| **Average across all UAs** |  | **75.2%** | **75.0%** | **82.1%** | **0.45** |
