# Supplementary material for: The tree cover and temperature disparity in US urbanized areas: Quantifying the association with income across 5,723 communities
Source: PLoS One. 2021 Apr 28;16(4):e0249715. doi: 10.1371/journal.pone.0249715 (PMC8081227; doi:10.1371/journal.pone.0249715)
Supplement: S2 Table — We conducted two regressions of patterns among urbanized areas (N = 100). The first regression predicted median tree cover, corresponding with the data shown in Fig 3A. The second regression predicted the difference in median tree cover for income quartiles (top quartile minus bottom quartile), corresponding with the data shown in Fig 3B. In both cases tree cover was arcsine transformed to improve normality. (DOCX) [file pone.0249715.s005.docx]

| ***Variable:*** | ***Estimate*** | ***z-value*** | ***Likelihood ratio*** | ***P*** |
| --- | --- | --- | --- | --- |
| Intercept | 1.417 | 5.0043 |  | <0.0001 |
| Biome (categorical) | Varies from 0 to -0.203 |  | 16.549 | 0.005 |
| Density (log-transformed) | -0.133 | -3.7642 |  | 0.0002 |
| Income | -0.00000146 | -0.8262 |  | 0.41 |

**Regression of median forest cover among urbanized areas (Spatial lag SAR model, Rho: 0.39984, Log Likelihood for overall model: 76.9088, P < 0.0001)**

**Regression of difference in median forest cover with respect to income among urbanized areas (Spatial lag SAR model, Rho: 0.40101, Log Likelihood for overall model: 114.9502, P < 0.0001)**

| ***Variable:*** | ***Estimate*** | ***z-value*** | ***Likelihood ratio*** | ***P*** |
| --- | --- | --- | --- | --- |
| Intercept | -0.0715 | -1.0318 |  | 0.30 |
| Biome (categorical) | Varies from 0 to -0.091 |  | 2.5391 | 0.77 |
| Median forest cover in UA (arcsine transformed) | -0.157 | -2.7831 |  | 0.005 |
| Density Interquartile Range (log-transformed) | 0.146 | 3.4267 |  | 0.0006 |
| Income Interquartile Range | 0.00000586 | 3.9234 |  | <0.0001 |
