# Supplementary material for: The tree cover and temperature disparity in US urbanized areas: Quantifying the association with income across 5,723 communities
Source: PLoS One. 2021 Apr 28;16(4):e0249715. doi: 10.1371/journal.pone.0249715 (PMC8081227; doi:10.1371/journal.pone.0249715)
Supplement: S3 Table — Data is sorted alphabetically by the name of the urbanized area and then the name of the place. We used the population-weighted median tree cover to estimate how much tree cover an average person had within their census block. Note that many incorporated places had high tree cover in large parks with relatively low population in them, which means that in these places the population-weighted median tree cover was generally less than the simple arithmetical mean. (DOCX) [file pone.0249715.s006.docx]

| **Urbanized Area name** | **Incorporate or census-designated place name** | **Median tree cover (%)** | **Median tree cover in entire urbanized area** |
| --- | --- | --- | --- |
| Akron, OH | Akron city | 22.8% | 26.6% |
| Akron, OH | Barberton city | 15.9% | 26.6% |
| Akron, OH | Brady Lake village | 54.9% | 26.6% |
| Akron, OH | Brimfield CDP | 24.0% | 26.6% |
| Akron, OH | Clinton village | 43.5% | 26.6% |
| Akron, OH | Cuyahoga Falls city | 25.9% | 26.6% |
| Akron, OH | Doylestown village | 34.3% | 26.6% |
| Akron, OH | Fairlawn city | 26.6% | 26.6% |
| Akron, OH | Green city | 33.8% | 26.6% |
| Akron, OH | Greentown CDP | 0.0% | 26.6% |
| Akron, OH | Hartville village | 18.0% | 26.6% |
| Akron, OH | Hudson city | 31.6% | 26.6% |
| Akron, OH | Kent city | 19.9% | 26.6% |
| Akron, OH | Lakemore village | 25.0% | 26.6% |
| Akron, OH | Mogadore village | 23.9% | 26.6% |
| Akron, OH | Montrose-Ghent CDP | 51.4% | 26.6% |
| Akron, OH | Munroe Falls city | 39.8% | 26.6% |
| Akron, OH | New Franklin city | 45.7% | 26.6% |
| Akron, OH | Norton city | 33.7% | 26.6% |
| Akron, OH | Pigeon Creek CDP | 47.4% | 26.6% |
| Akron, OH | Portage Lakes CDP | 35.7% | 26.6% |
| Akron, OH | Ravenna city | 23.9% | 26.6% |
| Akron, OH | Rittman city | 28.6% | 26.6% |
| Akron, OH | Sawyerwood CDP | 34.4% | 26.6% |
| Akron, OH | Silver Lake village | 42.7% | 26.6% |
| Akron, OH | Sterling CDP | 14.0% | 26.6% |
| Akron, OH | Stow city | 34.3% | 26.6% |
| Akron, OH | Streetsboro city | 25.6% | 26.6% |
| Akron, OH | Sugar Bush Knolls village | 53.3% | 26.6% |
| Akron, OH | Tallmadge city | 36.6% | 26.6% |
| Akron, OH | Uniontown CDP | 21.0% | 26.6% |
| Akron, OH | Wadsworth city | 27.2% | 26.6% |
| Albany--Schenectady, NY | Albany city | 14.8% | 24.6% |
| Albany--Schenectady, NY | Altamont village | 39.4% | 24.6% |
| Albany--Schenectady, NY | Averill Park CDP | 44.0% | 24.6% |
| Albany--Schenectady, NY | Castleton-on-Hudson village | 58.4% | 24.6% |
| Albany--Schenectady, NY | Cohoes city | 20.6% | 24.6% |
| Albany--Schenectady, NY | Colonie village | 24.4% | 24.6% |
| Albany--Schenectady, NY | Country Knolls CDP | 46.6% | 24.6% |
| Albany--Schenectady, NY | East Glenville CDP | 41.3% | 24.6% |
| Albany--Schenectady, NY | East Greenbush CDP | 39.1% | 24.6% |
| Albany--Schenectady, NY | Green Island village | 2.5% | 24.6% |
| Albany--Schenectady, NY | Hampton Manor CDP | 23.9% | 24.6% |
| Albany--Schenectady, NY | Mechanicville city | 12.1% | 24.6% |
| Albany--Schenectady, NY | Menands village | 24.4% | 24.6% |
| Albany--Schenectady, NY | Niskayuna CDP | 38.1% | 24.6% |
| Albany--Schenectady, NY | Poestenkill CDP | 41.5% | 24.6% |
| Albany--Schenectady, NY | Rensselaer city | 23.8% | 24.6% |
| Albany--Schenectady, NY | Rotterdam CDP | 20.0% | 24.6% |
| Albany--Schenectady, NY | Round Lake village | 19.5% | 24.6% |
| Albany--Schenectady, NY | Schenectady city | 12.0% | 24.6% |
| Albany--Schenectady, NY | Scotia village | 20.9% | 24.6% |
| Albany--Schenectady, NY | Stillwater village | 28.5% | 24.6% |
| Albany--Schenectady, NY | Troy city | 7.5% | 24.6% |
| Albany--Schenectady, NY | Voorheesville village | 48.6% | 24.6% |
| Albany--Schenectady, NY | Waterford village | 5.0% | 24.6% |
| Albany--Schenectady, NY | Watervliet city | 8.6% | 24.6% |
| Albany--Schenectady, NY | West Sand Lake CDP | 41.0% | 24.6% |
| Albany--Schenectady, NY | Westmere CDP | 38.9% | 24.6% |
| Albany--Schenectady, NY | Wynantskill CDP | 27.4% | 24.6% |
| Albuquerque, NM | Albuquerque city | 4.0% | 3.7% |
| Albuquerque, NM | Algodones CDP | 5.4% | 3.7% |
| Albuquerque, NM | Bernalillo town | 2.7% | 3.7% |
| Albuquerque, NM | Carnuel CDP | 0.2% | 3.7% |
| Albuquerque, NM | Corrales village | 5.1% | 3.7% |
| Albuquerque, NM | Edith Endave CDP | 11.0% | 3.7% |
| Albuquerque, NM | Isleta Village Proper CDP | 0.5% | 3.7% |
| Albuquerque, NM | Los Ranchos de Albuquerque village | 14.7% | 3.7% |
| Albuquerque, NM | North Valley CDP | 6.6% | 3.7% |
| Albuquerque, NM | Paradise Hills CDP | 6.8% | 3.7% |
| Albuquerque, NM | Placitas CDP | 0.1% | 3.7% |
| Albuquerque, NM | Pueblo of Sandia Village CDP | 0.4% | 3.7% |
| Albuquerque, NM | Rio Rancho city | 1.0% | 3.7% |
| Albuquerque, NM | Sandia Heights CDP | 2.8% | 3.7% |
| Albuquerque, NM | Santa Ana Pueblo CDP | 0.1% | 3.7% |
| Albuquerque, NM | South Valley CDP | 8.5% | 3.7% |
| Allentown, PA--NJ | Ackermanville CDP | 40.5% | 16.6% |
| Allentown, PA--NJ | Alburtis borough | 28.8% | 16.6% |
| Allentown, PA--NJ | Allentown city | 2.2% | 16.6% |
| Allentown, PA--NJ | Alpha borough | 9.9% | 16.6% |
| Allentown, PA--NJ | Ancient Oaks CDP | 29.6% | 16.6% |
| Allentown, PA--NJ | Bangor borough | 10.1% | 16.6% |
| Allentown, PA--NJ | Bath borough | 13.4% | 16.6% |
| Allentown, PA--NJ | Belfast CDP | 34.5% | 16.6% |
| Allentown, PA--NJ | Bethlehem city | 11.7% | 16.6% |
| Allentown, PA--NJ | Bloomsbury borough | 51.4% | 16.6% |
| Allentown, PA--NJ | Bowmanstown borough | 11.2% | 16.6% |
| Allentown, PA--NJ | Breinigsville CDP | 26.7% | 16.6% |
| Allentown, PA--NJ | Catasauqua borough | 6.5% | 16.6% |
| Allentown, PA--NJ | Cementon CDP | 25.5% | 16.6% |
| Allentown, PA--NJ | Cetronia CDP | 29.3% | 16.6% |
| Allentown, PA--NJ | Chapman borough | 41.9% | 16.6% |
| Allentown, PA--NJ | Cherryville CDP | 29.0% | 16.6% |
| Allentown, PA--NJ | Coopersburg borough | 21.7% | 16.6% |
| Allentown, PA--NJ | Coplay borough | 6.0% | 16.6% |
| Allentown, PA--NJ | DeSales University CDP | 16.4% | 16.6% |
| Allentown, PA--NJ | Delaware Park CDP | 15.9% | 16.6% |
| Allentown, PA--NJ | Dorneyville CDP | 29.7% | 16.6% |
| Allentown, PA--NJ | East Bangor borough | 29.6% | 16.6% |
| Allentown, PA--NJ | Eastlawn Gardens CDP | 21.4% | 16.6% |
| Allentown, PA--NJ | Easton city | 7.1% | 16.6% |
| Allentown, PA--NJ | Egypt CDP | 29.2% | 16.6% |
| Allentown, PA--NJ | Emmaus borough | 12.7% | 16.6% |
| Allentown, PA--NJ | Fountain Hill borough | 5.2% | 16.6% |
| Allentown, PA--NJ | Freemansburg borough | 28.0% | 16.6% |
| Allentown, PA--NJ | Fullerton CDP | 9.0% | 16.6% |
| Allentown, PA--NJ | Glendon borough | 23.1% | 16.6% |
| Allentown, PA--NJ | Greenwich CDP | 8.8% | 16.6% |
| Allentown, PA--NJ | Hellertown borough | 19.8% | 16.6% |
| Allentown, PA--NJ | Hereford CDP | 38.1% | 16.6% |
| Allentown, PA--NJ | Hokendauqua CDP | 17.1% | 16.6% |
| Allentown, PA--NJ | Laurys Station CDP | 19.4% | 16.6% |
| Allentown, PA--NJ | Lehighton borough | 3.5% | 16.6% |
| Allentown, PA--NJ | Lopatcong Overlook CDP | 22.7% | 16.6% |
| Allentown, PA--NJ | Macungie borough | 22.1% | 16.6% |
| Allentown, PA--NJ | Martins Creek CDP | 40.3% | 16.6% |
| Allentown, PA--NJ | Mertztown CDP | 33.1% | 16.6% |
| Allentown, PA--NJ | Middletown CDP | 36.0% | 16.6% |
| Allentown, PA--NJ | Milford Square CDP | 39.3% | 16.6% |
| Allentown, PA--NJ | Nazareth borough | 14.2% | 16.6% |
| Allentown, PA--NJ | North Catasauqua borough | 6.2% | 16.6% |
| Allentown, PA--NJ | Northampton borough | 7.1% | 16.6% |
| Allentown, PA--NJ | Old Orchard CDP | 43.0% | 16.6% |
| Allentown, PA--NJ | Palmer Heights CDP | 27.2% | 16.6% |
| Allentown, PA--NJ | Palmerton borough | 3.1% | 16.6% |
| Allentown, PA--NJ | Parryville borough | 5.4% | 16.6% |
| Allentown, PA--NJ | Pen Argyl borough | 11.6% | 16.6% |
| Allentown, PA--NJ | Phillipsburg town | 3.6% | 16.6% |
| Allentown, PA--NJ | Quakertown borough | 11.4% | 16.6% |
| Allentown, PA--NJ | Raubsville CDP | 33.3% | 16.6% |
| Allentown, PA--NJ | Richlandtown borough | 13.3% | 16.6% |
| Allentown, PA--NJ | Roseto borough | 15.5% | 16.6% |
| Allentown, PA--NJ | Saylorsburg CDP | 42.6% | 16.6% |
| Allentown, PA--NJ | Schnecksville CDP | 38.4% | 16.6% |
| Allentown, PA--NJ | Slatedale CDP | 3.5% | 16.6% |
| Allentown, PA--NJ | Slatington borough | 5.7% | 16.6% |
| Allentown, PA--NJ | Spinnerstown CDP | 12.7% | 16.6% |
| Allentown, PA--NJ | Stewartsville CDP | 41.6% | 16.6% |
| Allentown, PA--NJ | Stiles CDP | 13.4% | 16.6% |
| Allentown, PA--NJ | Stockertown borough | 25.3% | 16.6% |
| Allentown, PA--NJ | Tatamy borough | 10.3% | 16.6% |
| Allentown, PA--NJ | Topton borough | 9.8% | 16.6% |
| Allentown, PA--NJ | Trexlertown CDP | 20.7% | 16.6% |
| Allentown, PA--NJ | Trumbauersville borough | 15.4% | 16.6% |
| Allentown, PA--NJ | Upper Pohatcong CDP | 12.6% | 16.6% |
| Allentown, PA--NJ | Upper Stewartsville CDP | 47.4% | 16.6% |
| Allentown, PA--NJ | Walnutport borough | 11.6% | 16.6% |
| Allentown, PA--NJ | Weissport East CDP | 21.3% | 16.6% |
| Allentown, PA--NJ | Weissport borough | 5.0% | 16.6% |
| Allentown, PA--NJ | Wescosville CDP | 36.8% | 16.6% |
| Allentown, PA--NJ | West Easton borough | 15.2% | 16.6% |
| Allentown, PA--NJ | Wilson borough | 4.5% | 16.6% |
| Allentown, PA--NJ | Wind Gap borough | 38.1% | 16.6% |
| Asheville, NC | Asheville city | 54.8% | 59.0% |
| Asheville, NC | Avery Creek CDP | 67.9% | 59.0% |
| Asheville, NC | Balfour CDP | 68.8% | 59.0% |
| Asheville, NC | Barker Heights CDP | 57.5% | 59.0% |
| Asheville, NC | Bent Creek CDP | 65.7% | 59.0% |
| Asheville, NC | Biltmore Forest town | 67.0% | 59.0% |
| Asheville, NC | Black Mountain town | 50.1% | 59.0% |
| Asheville, NC | Canton town | 36.7% | 59.0% |
| Asheville, NC | Clyde town | 40.8% | 59.0% |
| Asheville, NC | Dana CDP | 55.9% | 59.0% |
| Asheville, NC | East Flat Rock CDP | 52.6% | 59.0% |
| Asheville, NC | Etowah CDP | 69.9% | 59.0% |
| Asheville, NC | Fairview CDP | 72.8% | 59.0% |
| Asheville, NC | Flat Rock village | 78.7% | 59.0% |
| Asheville, NC | Fletcher town | 46.0% | 59.0% |
| Asheville, NC | Fruitland CDP | 62.4% | 59.0% |
| Asheville, NC | Hendersonville city | 47.2% | 59.0% |
| Asheville, NC | Hoopers Creek CDP | 37.2% | 59.0% |
| Asheville, NC | Horse Shoe CDP | 72.5% | 59.0% |
| Asheville, NC | Lake Junaluska CDP | 54.0% | 59.0% |
| Asheville, NC | Laurel Park town | 77.6% | 59.0% |
| Asheville, NC | Maggie Valley town | 4.6% | 59.0% |
| Asheville, NC | Mars Hill town | 58.6% | 59.0% |
| Asheville, NC | Mills River town | 64.2% | 59.0% |
| Asheville, NC | Montreat town | 64.6% | 59.0% |
| Asheville, NC | Mountain Home CDP | 71.3% | 59.0% |
| Asheville, NC | Royal Pines CDP | 78.7% | 59.0% |
| Asheville, NC | Swannanoa CDP | 35.4% | 59.0% |
| Asheville, NC | Valley Hill CDP | 70.0% | 59.0% |
| Asheville, NC | Waynesville town | 43.7% | 59.0% |
| Asheville, NC | Weaverville town | 62.0% | 59.0% |
| Asheville, NC | West Canton CDP | 36.2% | 59.0% |
| Asheville, NC | Woodfin town | 65.1% | 59.0% |
| Atlanta, GA | Acworth city | 50.2% | 62.0% |
| Atlanta, GA | Alpharetta city | 61.7% | 62.0% |
| Atlanta, GA | Atlanta city | 64.0% | 62.0% |
| Atlanta, GA | Auburn city | 64.4% | 62.0% |
| Atlanta, GA | Austell city | 65.9% | 62.0% |
| Atlanta, GA | Avondale Estates city | 67.8% | 62.0% |
| Atlanta, GA | Belvedere Park CDP | 67.4% | 62.0% |
| Atlanta, GA | Berkeley Lake city | 77.0% | 62.0% |
| Atlanta, GA | Between town | 50.9% | 62.0% |
| Atlanta, GA | Bonanza CDP | 69.4% | 62.0% |
| Atlanta, GA | Braselton town | 34.1% | 62.0% |
| Atlanta, GA | Brookhaven city | 58.0% | 62.0% |
| Atlanta, GA | Buford city | 51.6% | 62.0% |
| Atlanta, GA | Candler-McAfee CDP | 70.2% | 62.0% |
| Atlanta, GA | Canton city | 63.6% | 62.0% |
| Atlanta, GA | Carl town | 70.1% | 62.0% |
| Atlanta, GA | Chamblee city | 58.1% | 62.0% |
| Atlanta, GA | Chattahoochee Hills city | 65.8% | 62.0% |
| Atlanta, GA | Clarkston city | 50.7% | 62.0% |
| Atlanta, GA | College Park city | 60.1% | 62.0% |
| Atlanta, GA | Conley CDP | 73.8% | 62.0% |
| Atlanta, GA | Conyers city | 43.9% | 62.0% |
| Atlanta, GA | Covington city | 50.2% | 62.0% |
| Atlanta, GA | Cumming city | 49.3% | 62.0% |
| Atlanta, GA | Dacula city | 60.4% | 62.0% |
| Atlanta, GA | Dallas city | 48.6% | 62.0% |
| Atlanta, GA | Decatur city | 77.7% | 62.0% |
| Atlanta, GA | Doraville city | 48.3% | 62.0% |
| Atlanta, GA | Douglasville city | 61.5% | 62.0% |
| Atlanta, GA | Druid Hills CDP | 84.6% | 62.0% |
| Atlanta, GA | Duluth city | 53.5% | 62.0% |
| Atlanta, GA | Dunwoody city | 59.1% | 62.0% |
| Atlanta, GA | East Griffin CDP | 63.4% | 62.0% |
| Atlanta, GA | East Newnan CDP | 79.3% | 62.0% |
| Atlanta, GA | East Point city | 67.6% | 62.0% |
| Atlanta, GA | Experiment CDP | 82.1% | 62.0% |
| Atlanta, GA | Fair Oaks CDP | 61.9% | 62.0% |
| Atlanta, GA | Fairburn city | 69.2% | 62.0% |
| Atlanta, GA | Fayetteville city | 63.1% | 62.0% |
| Atlanta, GA | Flowery Branch city | 36.6% | 62.0% |
| Atlanta, GA | Forest Park city | 69.0% | 62.0% |
| Atlanta, GA | Grayson city | 48.3% | 62.0% |
| Atlanta, GA | Gresham Park CDP | 83.2% | 62.0% |
| Atlanta, GA | Griffin city | 64.9% | 62.0% |
| Atlanta, GA | Hampton city | 67.6% | 62.0% |
| Atlanta, GA | Hapeville city | 71.1% | 62.0% |
| Atlanta, GA | Heron Bay CDP | 45.1% | 62.0% |
| Atlanta, GA | Hiram city | 43.5% | 62.0% |
| Atlanta, GA | Holly Springs city | 65.0% | 62.0% |
| Atlanta, GA | Hoschton city | 62.9% | 62.0% |
| Atlanta, GA | Irondale CDP | 64.0% | 62.0% |
| Atlanta, GA | Johns Creek city | 57.1% | 62.0% |
| Atlanta, GA | Jonesboro city | 60.9% | 62.0% |
| Atlanta, GA | Kennesaw city | 47.7% | 62.0% |
| Atlanta, GA | Lake City city | 56.5% | 62.0% |
| Atlanta, GA | Lakeview Estates CDP | 34.6% | 62.0% |
| Atlanta, GA | Lawrenceville city | 51.7% | 62.0% |
| Atlanta, GA | Lilburn city | 58.6% | 62.0% |
| Atlanta, GA | Lithia Springs CDP | 76.0% | 62.0% |
| Atlanta, GA | Lithonia city | 51.3% | 62.0% |
| Atlanta, GA | Locust Grove city | 42.4% | 62.0% |
| Atlanta, GA | Loganville city | 45.7% | 62.0% |
| Atlanta, GA | Lovejoy city | 39.4% | 62.0% |
| Atlanta, GA | Mableton CDP | 74.9% | 62.0% |
| Atlanta, GA | Marietta city | 50.5% | 62.0% |
| Atlanta, GA | McDonough city | 36.9% | 62.0% |
| Atlanta, GA | Milton city | 64.3% | 62.0% |
| Atlanta, GA | Morrow city | 62.9% | 62.0% |
| Atlanta, GA | Mountain Park CDP | 59.3% | 62.0% |
| Atlanta, GA | Mountain Park city | 89.3% | 62.0% |
| Atlanta, GA | Newnan city | 59.9% | 62.0% |
| Atlanta, GA | Norcross city | 49.6% | 62.0% |
| Atlanta, GA | North Decatur CDP | 75.0% | 62.0% |
| Atlanta, GA | North Druid Hills CDP | 69.6% | 62.0% |
| Atlanta, GA | Oxford city | 51.9% | 62.0% |
| Atlanta, GA | Palmetto city | 81.0% | 62.0% |
| Atlanta, GA | Panthersville CDP | 75.7% | 62.0% |
| Atlanta, GA | Peachtree City city | 74.4% | 62.0% |
| Atlanta, GA | Peachtree Corners city | 67.5% | 62.0% |
| Atlanta, GA | Pine Lake city | 83.1% | 62.0% |
| Atlanta, GA | Porterdale city | 29.8% | 62.0% |
| Atlanta, GA | Powder Springs city | 57.9% | 62.0% |
| Atlanta, GA | Redan CDP | 61.9% | 62.0% |
| Atlanta, GA | Riverdale city | 59.6% | 62.0% |
| Atlanta, GA | Roswell city | 61.2% | 62.0% |
| Atlanta, GA | Sandy Springs city | 60.6% | 62.0% |
| Atlanta, GA | Scottdale CDP | 58.2% | 62.0% |
| Atlanta, GA | Senoia city | 74.0% | 62.0% |
| Atlanta, GA | Sharpsburg town | 81.7% | 62.0% |
| Atlanta, GA | Smyrna city | 54.4% | 62.0% |
| Atlanta, GA | Snellville city | 55.5% | 62.0% |
| Atlanta, GA | South Fulton city | 72.7% | 62.0% |
| Atlanta, GA | Stockbridge city | 43.7% | 62.0% |
| Atlanta, GA | Stone Mountain city | 61.1% | 62.0% |
| Atlanta, GA | Stonecrest city | 61.4% | 62.0% |
| Atlanta, GA | Sugar Hill city | 58.1% | 62.0% |
| Atlanta, GA | Sunny Side city | 59.2% | 62.0% |
| Atlanta, GA | Suwanee city | 49.9% | 62.0% |
| Atlanta, GA | Temple city | 67.1% | 62.0% |
| Atlanta, GA | Tucker city | 62.8% | 62.0% |
| Atlanta, GA | Turin town | 67.7% | 62.0% |
| Atlanta, GA | Tyrone town | 79.4% | 62.0% |
| Atlanta, GA | Union City city | 66.0% | 62.0% |
| Atlanta, GA | Villa Rica city | 56.2% | 62.0% |
| Atlanta, GA | Vinings CDP | 53.6% | 62.0% |
| Atlanta, GA | Walnut Grove city | 60.6% | 62.0% |
| Atlanta, GA | Woodstock city | 51.2% | 62.0% |
| Augusta-Richmond County, GA--SC | Aiken city | 47.9% | 48.1% |
| Augusta-Richmond County, GA--SC | Augusta-Richmond County consolidated gov | 43.3% | 48.1% |
| Augusta-Richmond County, GA--SC | Belvedere CDP | 40.8% | 48.1% |
| Augusta-Richmond County, GA--SC | Burnettown town | 50.8% | 48.1% |
| Augusta-Richmond County, GA--SC | Clearwater CDP | 44.4% | 48.1% |
| Augusta-Richmond County, GA--SC | Evans CDP | 57.7% | 48.1% |
| Augusta-Richmond County, GA--SC | Gloverville CDP | 66.1% | 48.1% |
| Augusta-Richmond County, GA--SC | Graniteville CDP | 58.2% | 48.1% |
| Augusta-Richmond County, GA--SC | Grovetown city | 38.0% | 48.1% |
| Augusta-Richmond County, GA--SC | Hephzibah city | 51.6% | 48.1% |
| Augusta-Richmond County, GA--SC | Langley CDP | 48.2% | 48.1% |
| Augusta-Richmond County, GA--SC | Martinez CDP | 52.7% | 48.1% |
| Augusta-Richmond County, GA--SC | Murphys Estates CDP | 46.8% | 48.1% |
| Augusta-Richmond County, GA--SC | New Ellenton town | 50.8% | 48.1% |
| Augusta-Richmond County, GA--SC | North Augusta city | 55.2% | 48.1% |
| Augusta-Richmond County, GA--SC | Warrenville CDP | 55.0% | 48.1% |
| Austin, TX | Austin city | 41.0% | 35.6% |
| Austin, TX | Barton Creek CDP | 60.9% | 35.6% |
| Austin, TX | Bear Creek village | 57.5% | 35.6% |
| Austin, TX | Bee Cave city | 26.9% | 35.6% |
| Austin, TX | Brushy Creek CDP | 42.3% | 35.6% |
| Austin, TX | Buda city | 22.0% | 35.6% |
| Austin, TX | Cedar Park city | 29.9% | 35.6% |
| Austin, TX | Georgetown city | 32.9% | 35.6% |
| Austin, TX | Hays city | 66.0% | 35.6% |
| Austin, TX | Hornsby Bend CDP | 17.4% | 35.6% |
| Austin, TX | Hudson Bend CDP | 44.7% | 35.6% |
| Austin, TX | Hutto city | 7.9% | 35.6% |
| Austin, TX | Kyle city | 19.7% | 35.6% |
| Austin, TX | Lakeway city | 45.5% | 35.6% |
| Austin, TX | Leander city | 17.2% | 35.6% |
| Austin, TX | Lost Creek CDP | 72.4% | 35.6% |
| Austin, TX | Manchaca CDP | 43.4% | 35.6% |
| Austin, TX | Manor city | 14.4% | 35.6% |
| Austin, TX | Pflugerville city | 18.2% | 35.6% |
| Austin, TX | Rollingwood city | 62.6% | 35.6% |
| Austin, TX | Round Rock city | 29.7% | 35.6% |
| Austin, TX | San Leanna village | 52.2% | 35.6% |
| Austin, TX | San Marcos city | 20.7% | 35.6% |
| Austin, TX | Serenada CDP | 65.0% | 35.6% |
| Austin, TX | Shady Hollow CDP | 58.5% | 35.6% |
| Austin, TX | Sunset Valley city | 48.6% | 35.6% |
| Austin, TX | The Hills village | 38.9% | 35.6% |
| Austin, TX | Uhland city | 8.0% | 35.6% |
| Austin, TX | Wells Branch CDP | 22.6% | 35.6% |
| Austin, TX | West Lake Hills city | 77.4% | 35.6% |
| Baltimore, MD | Annapolis Neck CDP | 71.8% | 39.9% |
| Baltimore, MD | Annapolis city | 40.5% | 39.9% |
| Baltimore, MD | Arbutus CDP | 33.6% | 39.9% |
| Baltimore, MD | Arden on the Severn CDP | 82.3% | 39.9% |
| Baltimore, MD | Arnold CDP | 69.5% | 39.9% |
| Baltimore, MD | Baltimore Highlands CDP | 26.2% | 39.9% |
| Baltimore, MD | Baltimore city | 19.3% | 39.9% |
| Baltimore, MD | Bowleys Quarters CDP | 20.9% | 39.9% |
| Baltimore, MD | Brooklyn Park CDP | 22.6% | 39.9% |
| Baltimore, MD | Burtonsville CDP | 65.0% | 39.9% |
| Baltimore, MD | Cape St. Claire CDP | 66.6% | 39.9% |
| Baltimore, MD | Carney CDP | 45.0% | 39.9% |
| Baltimore, MD | Catonsville CDP | 41.0% | 39.9% |
| Baltimore, MD | Chester CDP | 29.4% | 39.9% |
| Baltimore, MD | Cockeysville CDP | 41.7% | 39.9% |
| Baltimore, MD | Columbia CDP | 65.7% | 39.9% |
| Baltimore, MD | Crofton CDP | 55.0% | 39.9% |
| Baltimore, MD | Crownsville CDP | 80.8% | 39.9% |
| Baltimore, MD | Dundalk CDP | 12.8% | 39.9% |
| Baltimore, MD | Edgemere CDP | 42.1% | 39.9% |
| Baltimore, MD | Edgewater CDP | 42.6% | 39.9% |
| Baltimore, MD | Elkridge CDP | 42.2% | 39.9% |
| Baltimore, MD | Ellicott City CDP | 64.9% | 39.9% |
| Baltimore, MD | Essex CDP | 37.2% | 39.9% |
| Baltimore, MD | Ferndale CDP | 38.7% | 39.9% |
| Baltimore, MD | Fort Meade CDP | 34.8% | 39.9% |
| Baltimore, MD | Fulton CDP | 14.9% | 39.9% |
| Baltimore, MD | Gambrills CDP | 64.6% | 39.9% |
| Baltimore, MD | Garrison CDP | 48.6% | 39.9% |
| Baltimore, MD | Glen Burnie CDP | 42.3% | 39.9% |
| Baltimore, MD | Grasonville CDP | 29.2% | 39.9% |
| Baltimore, MD | Hampstead town | 47.0% | 39.9% |
| Baltimore, MD | Hampton CDP | 72.3% | 39.9% |
| Baltimore, MD | Herald Harbor CDP | 81.4% | 39.9% |
| Baltimore, MD | Highland Beach town | 69.7% | 39.9% |
| Baltimore, MD | Highland CDP | 74.2% | 39.9% |
| Baltimore, MD | Ilchester CDP | 61.1% | 39.9% |
| Baltimore, MD | Jessup CDP | 40.2% | 39.9% |
| Baltimore, MD | Kent Narrows CDP | 3.4% | 39.9% |
| Baltimore, MD | Konterra CDP | 31.4% | 39.9% |
| Baltimore, MD | Lake Shore CDP | 73.9% | 39.9% |
| Baltimore, MD | Lansdowne CDP | 38.4% | 39.9% |
| Baltimore, MD | Laurel city | 24.7% | 39.9% |
| Baltimore, MD | Linthicum CDP | 49.3% | 39.9% |
| Baltimore, MD | Lochearn CDP | 38.0% | 39.9% |
| Baltimore, MD | Lutherville CDP | 43.0% | 39.9% |
| Baltimore, MD | Maryland City CDP | 33.4% | 39.9% |
| Baltimore, MD | Mayo CDP | 53.8% | 39.9% |
| Baltimore, MD | Mays Chapel CDP | 51.7% | 39.9% |
| Baltimore, MD | Middle River CDP | 25.1% | 39.9% |
| Baltimore, MD | Milford Mill CDP | 31.3% | 39.9% |
| Baltimore, MD | Naval Academy CDP | 57.1% | 39.9% |
| Baltimore, MD | North Laurel CDP | 41.1% | 39.9% |
| Baltimore, MD | Odenton CDP | 41.6% | 39.9% |
| Baltimore, MD | Overlea CDP | 35.8% | 39.9% |
| Baltimore, MD | Owings Mills CDP | 52.1% | 39.9% |
| Baltimore, MD | Parkville CDP | 27.0% | 39.9% |
| Baltimore, MD | Parole CDP | 67.2% | 39.9% |
| Baltimore, MD | Pasadena CDP | 50.4% | 39.9% |
| Baltimore, MD | Perry Hall CDP | 45.8% | 39.9% |
| Baltimore, MD | Pikesville CDP | 44.0% | 39.9% |
| Baltimore, MD | Randallstown CDP | 47.9% | 39.9% |
| Baltimore, MD | Reisterstown CDP | 52.5% | 39.9% |
| Baltimore, MD | Riva CDP | 71.6% | 39.9% |
| Baltimore, MD | Riviera Beach CDP | 31.9% | 39.9% |
| Baltimore, MD | Rosedale CDP | 36.6% | 39.9% |
| Baltimore, MD | Rossville CDP | 45.3% | 39.9% |
| Baltimore, MD | Savage CDP | 61.8% | 39.9% |
| Baltimore, MD | Scaggsville CDP | 67.7% | 39.9% |
| Baltimore, MD | Severn CDP | 50.4% | 39.9% |
| Baltimore, MD | Severna Park CDP | 69.9% | 39.9% |
| Baltimore, MD | South Laurel CDP | 48.5% | 39.9% |
| Baltimore, MD | Stevensville CDP | 40.4% | 39.9% |
| Baltimore, MD | Timonium CDP | 50.2% | 39.9% |
| Baltimore, MD | Towson CDP | 41.8% | 39.9% |
| Baltimore, MD | West Laurel CDP | 68.0% | 39.9% |
| Baltimore, MD | White Marsh CDP | 53.3% | 39.9% |
| Baltimore, MD | Woodlawn CDP | 32.3% | 39.9% |
| Barnstable Town, MA | Barnstable Town city | 59.0% | 56.5% |
| Barnstable Town, MA | Bourne CDP | 42.0% | 56.5% |
| Barnstable Town, MA | Brewster CDP | 64.1% | 56.5% |
| Barnstable Town, MA | Buzzards Bay CDP | 32.6% | 56.5% |
| Barnstable Town, MA | Chatham CDP | 32.8% | 56.5% |
| Barnstable Town, MA | Dennis CDP | 65.8% | 56.5% |
| Barnstable Town, MA | Dennis Port CDP | 38.5% | 56.5% |
| Barnstable Town, MA | East Dennis CDP | 60.6% | 56.5% |
| Barnstable Town, MA | East Falmouth CDP | 52.1% | 56.5% |
| Barnstable Town, MA | East Harwich CDP | 54.2% | 56.5% |
| Barnstable Town, MA | East Sandwich CDP | 71.8% | 56.5% |
| Barnstable Town, MA | Falmouth CDP | 36.5% | 56.5% |
| Barnstable Town, MA | Forestdale CDP | 66.6% | 56.5% |
| Barnstable Town, MA | Harwich Center CDP | 61.1% | 56.5% |
| Barnstable Town, MA | Harwich Port CDP | 51.8% | 56.5% |
| Barnstable Town, MA | Marion Center CDP | 48.1% | 56.5% |
| Barnstable Town, MA | Mashpee Neck CDP | 64.6% | 56.5% |
| Barnstable Town, MA | Monomoscoy Island CDP | 38.2% | 56.5% |
| Barnstable Town, MA | Monument Beach CDP | 58.6% | 56.5% |
| Barnstable Town, MA | New Seabury CDP | 58.8% | 56.5% |
| Barnstable Town, MA | North Eastham CDP | 52.9% | 56.5% |
| Barnstable Town, MA | North Falmouth CDP | 68.2% | 56.5% |
| Barnstable Town, MA | Northwest Harwich CDP | 60.7% | 56.5% |
| Barnstable Town, MA | Onset CDP | 13.7% | 56.5% |
| Barnstable Town, MA | Orleans CDP | 44.1% | 56.5% |
| Barnstable Town, MA | Pocasset CDP | 53.9% | 56.5% |
| Barnstable Town, MA | Popponesset CDP | 33.0% | 56.5% |
| Barnstable Town, MA | Popponesset Island CDP | 25.8% | 56.5% |
| Barnstable Town, MA | Sagamore CDP | 61.7% | 56.5% |
| Barnstable Town, MA | Sandwich CDP | 55.1% | 56.5% |
| Barnstable Town, MA | Seabrook CDP | 63.2% | 56.5% |
| Barnstable Town, MA | Seconsett Island CDP | 51.7% | 56.5% |
| Barnstable Town, MA | South Dennis CDP | 43.8% | 56.5% |
| Barnstable Town, MA | South Yarmouth CDP | 48.7% | 56.5% |
| Barnstable Town, MA | Teaticket CDP | 46.5% | 56.5% |
| Barnstable Town, MA | The Pinehills CDP | 48.1% | 56.5% |
| Barnstable Town, MA | Wareham Center CDP | 31.9% | 56.5% |
| Barnstable Town, MA | West Chatham CDP | 37.1% | 56.5% |
| Barnstable Town, MA | West Dennis CDP | 39.1% | 56.5% |
| Barnstable Town, MA | West Falmouth CDP | 60.3% | 56.5% |
| Barnstable Town, MA | West Wareham CDP | 57.5% | 56.5% |
| Barnstable Town, MA | West Yarmouth CDP | 51.2% | 56.5% |
| Barnstable Town, MA | Weweantic CDP | 50.1% | 56.5% |
| Barnstable Town, MA | White Island Shores CDP | 31.8% | 56.5% |
| Barnstable Town, MA | Woods Hole CDP | 44.4% | 56.5% |
| Barnstable Town, MA | Yarmouth Port CDP | 56.1% | 56.5% |
| Baton Rouge, LA | Addis town | 56.4% | 63.3% |
| Baton Rouge, LA | Baker city | 71.5% | 63.3% |
| Baton Rouge, LA | Baton Rouge city | 56.7% | 63.3% |
| Baton Rouge, LA | Brownfields CDP | 81.3% | 63.3% |
| Baton Rouge, LA | Brusly town | 60.8% | 63.3% |
| Baton Rouge, LA | Central city | 82.3% | 63.3% |
| Baton Rouge, LA | Denham Springs city | 68.0% | 63.3% |
| Baton Rouge, LA | Gardere CDP | 46.0% | 63.3% |
| Baton Rouge, LA | Gonzales city | 62.6% | 63.3% |
| Baton Rouge, LA | Inniswold CDP | 45.1% | 63.3% |
| Baton Rouge, LA | Merrydale CDP | 66.4% | 63.3% |
| Baton Rouge, LA | Monticello CDP | 57.9% | 63.3% |
| Baton Rouge, LA | Oak Hills Place CDP | 56.2% | 63.3% |
| Baton Rouge, LA | Old Jefferson CDP | 70.6% | 63.3% |
| Baton Rouge, LA | Plaquemine city | 45.6% | 63.3% |
| Baton Rouge, LA | Port Allen city | 50.9% | 63.3% |
| Baton Rouge, LA | Port Vincent village | 91.4% | 63.3% |
| Baton Rouge, LA | Prairieville CDP | 67.0% | 63.3% |
| Baton Rouge, LA | Shenandoah CDP | 56.4% | 63.3% |
| Baton Rouge, LA | Sorrento town | 81.9% | 63.3% |
| Baton Rouge, LA | St. Gabriel city | 83.2% | 63.3% |
| Baton Rouge, LA | Village St. George CDP | 58.9% | 63.3% |
| Baton Rouge, LA | Walker town | 81.7% | 63.3% |
| Baton Rouge, LA | Watson CDP | 71.5% | 63.3% |
| Baton Rouge, LA | Westminster CDP | 61.5% | 63.3% |
| Baton Rouge, LA | Zachary city | 66.2% | 63.3% |
| Birmingham, AL | Adamsville city | 69.5% | 64.1% |
| Birmingham, AL | Alabaster city | 70.0% | 64.1% |
| Birmingham, AL | Argo town | 68.4% | 64.1% |
| Birmingham, AL | Bessemer city | 49.5% | 64.1% |
| Birmingham, AL | Birmingham city | 52.2% | 64.1% |
| Birmingham, AL | Brantleyville CDP | 90.5% | 64.1% |
| Birmingham, AL | Brighton city | 60.5% | 64.1% |
| Birmingham, AL | Brook Highland CDP | 51.8% | 64.1% |
| Birmingham, AL | Brookside town | 70.8% | 64.1% |
| Birmingham, AL | Calera city | 45.6% | 64.1% |
| Birmingham, AL | Center Point city | 69.2% | 64.1% |
| Birmingham, AL | Chelsea city | 72.1% | 64.1% |
| Birmingham, AL | Clay city | 73.9% | 64.1% |
| Birmingham, AL | Concord CDP | 74.7% | 64.1% |
| Birmingham, AL | Edgewater CDP | 61.3% | 64.1% |
| Birmingham, AL | Fairfield city | 48.2% | 64.1% |
| Birmingham, AL | Forestdale CDP | 75.2% | 64.1% |
| Birmingham, AL | Fultondale city | 80.0% | 64.1% |
| Birmingham, AL | Gardendale city | 71.4% | 64.1% |
| Birmingham, AL | Grayson Valley CDP | 63.0% | 64.1% |
| Birmingham, AL | Graysville city | 67.9% | 64.1% |
| Birmingham, AL | Helena city | 67.8% | 64.1% |
| Birmingham, AL | Highland Lakes CDP | 70.2% | 64.1% |
| Birmingham, AL | Homewood city | 64.8% | 64.1% |
| Birmingham, AL | Hoover city | 67.1% | 64.1% |
| Birmingham, AL | Hueytown city | 65.2% | 64.1% |
| Birmingham, AL | Indian Springs Village city | 83.3% | 64.1% |
| Birmingham, AL | Irondale city | 69.0% | 64.1% |
| Birmingham, AL | Kimberly city | 69.7% | 64.1% |
| Birmingham, AL | Leeds city | 65.8% | 64.1% |
| Birmingham, AL | Lipscomb city | 67.6% | 64.1% |
| Birmingham, AL | Maytown town | 71.4% | 64.1% |
| Birmingham, AL | McDonald Chapel CDP | 62.9% | 64.1% |
| Birmingham, AL | Meadowbrook CDP | 73.6% | 64.1% |
| Birmingham, AL | Midfield city | 64.1% | 64.1% |
| Birmingham, AL | Minor CDP | 74.0% | 64.1% |
| Birmingham, AL | Montevallo city | 62.7% | 64.1% |
| Birmingham, AL | Moody city | 49.1% | 64.1% |
| Birmingham, AL | Morris town | 79.9% | 64.1% |
| Birmingham, AL | Mount Olive CDP | 82.1% | 64.1% |
| Birmingham, AL | Mountain Brook city | 77.0% | 64.1% |
| Birmingham, AL | Mulga town | 59.0% | 64.1% |
| Birmingham, AL | Pelham city | 68.5% | 64.1% |
| Birmingham, AL | Pinson city | 78.4% | 64.1% |
| Birmingham, AL | Pleasant Grove city | 66.2% | 64.1% |
| Birmingham, AL | Shoal Creek CDP | 57.4% | 64.1% |
| Birmingham, AL | Sylvan Springs town | 64.3% | 64.1% |
| Birmingham, AL | Tarrant city | 61.2% | 64.1% |
| Birmingham, AL | Trussville city | 73.3% | 64.1% |
| Birmingham, AL | Vestavia Hills city | 71.2% | 64.1% |
| Birmingham, AL | Wilton town | 85.5% | 64.1% |
| Bonita Springs, FL | Bonita Springs city | 20.4% | 22.7% |
| Bonita Springs, FL | Estero village | 20.4% | 22.7% |
| Bonita Springs, FL | Golden Gate CDP | 19.3% | 22.7% |
| Bonita Springs, FL | Island Walk CDP | 24.8% | 22.7% |
| Bonita Springs, FL | Lely CDP | 14.3% | 22.7% |
| Bonita Springs, FL | Lely Resort CDP | 28.3% | 22.7% |
| Bonita Springs, FL | Marco Island city | 9.3% | 22.7% |
| Bonita Springs, FL | Naples Manor CDP | 18.9% | 22.7% |
| Bonita Springs, FL | Naples Park CDP | 11.7% | 22.7% |
| Bonita Springs, FL | Naples city | 18.2% | 22.7% |
| Bonita Springs, FL | Pelican Bay CDP | 26.7% | 22.7% |
| Bonita Springs, FL | Pine Ridge CDP | 29.4% | 22.7% |
| Bonita Springs, FL | Verona Walk CDP | 22.6% | 22.7% |
| Bonita Springs, FL | Vineyards CDP | 34.2% | 22.7% |
| Boston, MA--NH--RI | Abington CDP | 60.1% | 32.3% |
| Boston, MA--NH--RI | Amesbury Town city | 54.1% | 32.3% |
| Boston, MA--NH--RI | Andover CDP | 51.7% | 32.3% |
| Boston, MA--NH--RI | Arlington CDP | 29.7% | 32.3% |
| Boston, MA--NH--RI | Ayer CDP | 36.1% | 32.3% |
| Boston, MA--NH--RI | Bellingham CDP | 58.9% | 32.3% |
| Boston, MA--NH--RI | Belmont CDP | 25.9% | 32.3% |
| Boston, MA--NH--RI | Beverly city | 28.7% | 32.3% |
| Boston, MA--NH--RI | Boston city | 10.3% | 32.3% |
| Boston, MA--NH--RI | Boxford CDP | 77.1% | 32.3% |
| Boston, MA--NH--RI | Braintree Town city | 42.6% | 32.3% |
| Boston, MA--NH--RI | Bridgewater CDP | 35.0% | 32.3% |
| Boston, MA--NH--RI | Brockton city | 27.2% | 32.3% |
| Boston, MA--NH--RI | Brookline CDP | 23.9% | 32.3% |
| Boston, MA--NH--RI | Burlington CDP | 57.4% | 32.3% |
| Boston, MA--NH--RI | Cambridge city | 12.1% | 32.3% |
| Boston, MA--NH--RI | Chelsea city | 6.5% | 32.3% |
| Boston, MA--NH--RI | Cochituate CDP | 64.2% | 32.3% |
| Boston, MA--NH--RI | Cordaville CDP | 68.4% | 32.3% |
| Boston, MA--NH--RI | Danvers CDP | 40.3% | 32.3% |
| Boston, MA--NH--RI | Dedham CDP | 44.8% | 32.3% |
| Boston, MA--NH--RI | Devens CDP | 28.1% | 32.3% |
| Boston, MA--NH--RI | Dover CDP | 68.2% | 32.3% |
| Boston, MA--NH--RI | Duxbury CDP | 55.9% | 32.3% |
| Boston, MA--NH--RI | Essex CDP | 51.5% | 32.3% |
| Boston, MA--NH--RI | Everett city | 5.5% | 32.3% |
| Boston, MA--NH--RI | Foxborough CDP | 54.7% | 32.3% |
| Boston, MA--NH--RI | Framingham city | 40.6% | 32.3% |
| Boston, MA--NH--RI | Franklin Town city | 65.1% | 32.3% |
| Boston, MA--NH--RI | Gloucester city | 27.0% | 32.3% |
| Boston, MA--NH--RI | Green Harbor-Cedar Crest CDP | 24.6% | 32.3% |
| Boston, MA--NH--RI | Groton CDP | 50.3% | 32.3% |
| Boston, MA--NH--RI | Hanscom AFB CDP | 27.6% | 32.3% |
| Boston, MA--NH--RI | Hanson CDP | 63.8% | 32.3% |
| Boston, MA--NH--RI | Haverhill city | 37.6% | 32.3% |
| Boston, MA--NH--RI | Hingham CDP | 62.1% | 32.3% |
| Boston, MA--NH--RI | Holbrook CDP | 55.6% | 32.3% |
| Boston, MA--NH--RI | Hopedale CDP | 36.1% | 32.3% |
| Boston, MA--NH--RI | Hopkinton CDP | 57.9% | 32.3% |
| Boston, MA--NH--RI | Hudson CDP | 44.7% | 32.3% |
| Boston, MA--NH--RI | Hull CDP | 6.0% | 32.3% |
| Boston, MA--NH--RI | Ipswich CDP | 28.8% | 32.3% |
| Boston, MA--NH--RI | Kingston CDP | 54.3% | 32.3% |
| Boston, MA--NH--RI | Lawrence city | 9.8% | 32.3% |
| Boston, MA--NH--RI | Lexington CDP | 61.2% | 32.3% |
| Boston, MA--NH--RI | Littleton Common CDP | 59.8% | 32.3% |
| Boston, MA--NH--RI | Lowell city | 13.9% | 32.3% |
| Boston, MA--NH--RI | Lynn city | 10.0% | 32.3% |
| Boston, MA--NH--RI | Lynnfield CDP | 65.0% | 32.3% |
| Boston, MA--NH--RI | Malden city | 13.1% | 32.3% |
| Boston, MA--NH--RI | Mansfield Center CDP | 42.5% | 32.3% |
| Boston, MA--NH--RI | Marblehead CDP | 26.0% | 32.3% |
| Boston, MA--NH--RI | Marlborough city | 43.4% | 32.3% |
| Boston, MA--NH--RI | Marshfield CDP | 62.3% | 32.3% |
| Boston, MA--NH--RI | Marshfield Hills CDP | 76.0% | 32.3% |
| Boston, MA--NH--RI | Maynard CDP | 49.1% | 32.3% |
| Boston, MA--NH--RI | Medfield CDP | 57.4% | 32.3% |
| Boston, MA--NH--RI | Medford city | 14.1% | 32.3% |
| Boston, MA--NH--RI | Melrose city | 28.9% | 32.3% |
| Boston, MA--NH--RI | Methuen Town city | 40.2% | 32.3% |
| Boston, MA--NH--RI | Middleborough Center CDP | 44.1% | 32.3% |
| Boston, MA--NH--RI | Milford CDP | 37.7% | 32.3% |
| Boston, MA--NH--RI | Millis-Clicquot CDP | 52.5% | 32.3% |
| Boston, MA--NH--RI | Milton CDP | 42.2% | 32.3% |
| Boston, MA--NH--RI | Nahant CDP | 18.5% | 32.3% |
| Boston, MA--NH--RI | Needham CDP | 48.6% | 32.3% |
| Boston, MA--NH--RI | Newburyport city | 26.1% | 32.3% |
| Boston, MA--NH--RI | Newton city | 39.5% | 32.3% |
| Boston, MA--NH--RI | North Lakeville CDP | 64.8% | 32.3% |
| Boston, MA--NH--RI | North Pembroke CDP | 70.6% | 32.3% |
| Boston, MA--NH--RI | North Plymouth CDP | 45.7% | 32.3% |
| Boston, MA--NH--RI | North Scituate CDP | 65.2% | 32.3% |
| Boston, MA--NH--RI | Norton Center CDP | 49.0% | 32.3% |
| Boston, MA--NH--RI | Norwood CDP | 37.4% | 32.3% |
| Boston, MA--NH--RI | Ocean Bluff-Brant Rock CDP | 25.2% | 32.3% |
| Boston, MA--NH--RI | Peabody city | 31.3% | 32.3% |
| Boston, MA--NH--RI | Pinehurst CDP | 57.6% | 32.3% |
| Boston, MA--NH--RI | Plymouth CDP | 38.7% | 32.3% |
| Boston, MA--NH--RI | Quincy city | 16.5% | 32.3% |
| Boston, MA--NH--RI | Randolph CDP | 45.0% | 32.3% |
| Boston, MA--NH--RI | Raymond CDP | 61.4% | 32.3% |
| Boston, MA--NH--RI | Raynham Center CDP | 69.0% | 32.3% |
| Boston, MA--NH--RI | Reading CDP | 51.6% | 32.3% |
| Boston, MA--NH--RI | Revere city | 8.1% | 32.3% |
| Boston, MA--NH--RI | Rockport CDP | 49.1% | 32.3% |
| Boston, MA--NH--RI | Rowley CDP | 61.8% | 32.3% |
| Boston, MA--NH--RI | Salem city | 13.7% | 32.3% |
| Boston, MA--NH--RI | Salisbury CDP | 52.2% | 32.3% |
| Boston, MA--NH--RI | Saugus CDP | 38.0% | 32.3% |
| Boston, MA--NH--RI | Scituate CDP | 56.9% | 32.3% |
| Boston, MA--NH--RI | Seabrook Beach CDP | 0.6% | 32.3% |
| Boston, MA--NH--RI | Sharon CDP | 58.9% | 32.3% |
| Boston, MA--NH--RI | Shirley CDP | 43.3% | 32.3% |
| Boston, MA--NH--RI | Somerville city | 8.0% | 32.3% |
| Boston, MA--NH--RI | South Duxbury CDP | 68.0% | 32.3% |
| Boston, MA--NH--RI | Southfield CDP | 69.3% | 32.3% |
| Boston, MA--NH--RI | Stoneham CDP | 35.7% | 32.3% |
| Boston, MA--NH--RI | Swampscott CDP | 21.0% | 32.3% |
| Boston, MA--NH--RI | Taunton city | 51.6% | 32.3% |
| Boston, MA--NH--RI | Topsfield CDP | 72.8% | 32.3% |
| Boston, MA--NH--RI | Townsend CDP | 52.1% | 32.3% |
| Boston, MA--NH--RI | Wakefield CDP | 40.4% | 32.3% |
| Boston, MA--NH--RI | Walpole CDP | 53.2% | 32.3% |
| Boston, MA--NH--RI | Waltham city | 20.5% | 32.3% |
| Boston, MA--NH--RI | Watertown Town city | 13.0% | 32.3% |
| Boston, MA--NH--RI | Wellesley CDP | 54.8% | 32.3% |
| Boston, MA--NH--RI | West Concord CDP | 49.5% | 32.3% |
| Boston, MA--NH--RI | Weymouth Town city | 41.6% | 32.3% |
| Boston, MA--NH--RI | Wilmington CDP | 68.2% | 32.3% |
| Boston, MA--NH--RI | Winchester CDP | 49.0% | 32.3% |
| Boston, MA--NH--RI | Winthrop Town city | 7.1% | 32.3% |
| Boston, MA--NH--RI | Woburn city | 37.2% | 32.3% |
| Bridgeport--Stamford, CT--NY | Ansonia city | 47.7% | 44.5% |
| Bridgeport--Stamford, CT--NY | Bridgeport city | 13.3% | 44.5% |
| Bridgeport--Stamford, CT--NY | Byram CDP | 26.6% | 44.5% |
| Bridgeport--Stamford, CT--NY | Cannondale CDP | 91.9% | 44.5% |
| Bridgeport--Stamford, CT--NY | Cos Cob CDP | 45.9% | 44.5% |
| Bridgeport--Stamford, CT--NY | Danbury city | 88.0% | 44.5% |
| Bridgeport--Stamford, CT--NY | Darien CDP | 61.9% | 44.5% |
| Bridgeport--Stamford, CT--NY | Derby city | 46.8% | 44.5% |
| Bridgeport--Stamford, CT--NY | Georgetown CDP | 90.1% | 44.5% |
| Bridgeport--Stamford, CT--NY | Glenville CDP | 52.4% | 44.5% |
| Bridgeport--Stamford, CT--NY | Greenwich CDP | 29.8% | 44.5% |
| Bridgeport--Stamford, CT--NY | Harrison village | 57.6% | 44.5% |
| Bridgeport--Stamford, CT--NY | Heritage Village CDP | 46.8% | 44.5% |
| Bridgeport--Stamford, CT--NY | Milford city (balance) | 43.0% | 44.5% |
| Bridgeport--Stamford, CT--NY | Norwalk city | 36.4% | 44.5% |
| Bridgeport--Stamford, CT--NY | Old Greenwich CDP | 40.2% | 44.5% |
| Bridgeport--Stamford, CT--NY | Orange CDP | 79.6% | 44.5% |
| Bridgeport--Stamford, CT--NY | Pemberwick CDP | 32.1% | 44.5% |
| Bridgeport--Stamford, CT--NY | Port Chester village | 11.2% | 44.5% |
| Bridgeport--Stamford, CT--NY | Ridgefield CDP | 83.0% | 44.5% |
| Bridgeport--Stamford, CT--NY | Riverside CDP | 47.9% | 44.5% |
| Bridgeport--Stamford, CT--NY | Rye Brook village | 52.5% | 44.5% |
| Bridgeport--Stamford, CT--NY | Rye city | 48.3% | 44.5% |
| Bridgeport--Stamford, CT--NY | Shelton city | 71.4% | 44.5% |
| Bridgeport--Stamford, CT--NY | Southport CDP | 50.5% | 44.5% |
| Bridgeport--Stamford, CT--NY | Stamford city | 19.6% | 44.5% |
| Bridgeport--Stamford, CT--NY | Stratford CDP | 30.6% | 44.5% |
| Bridgeport--Stamford, CT--NY | Trumbull CDP | 68.1% | 44.5% |
| Bridgeport--Stamford, CT--NY | West Haven city | 44.7% | 44.5% |
| Bridgeport--Stamford, CT--NY | Westport CDP | 67.5% | 44.5% |
| Bridgeport--Stamford, CT--NY | Wilton Center CDP | 54.5% | 44.5% |
| Bridgeport--Stamford, CT--NY | Woodbury Center CDP | 56.5% | 44.5% |
| Bridgeport--Stamford, CT--NY | Woodmont borough | 22.9% | 44.5% |
| Buffalo, NY | Angola on the Lake CDP | 57.5% | 16.8% |
| Buffalo, NY | Angola village | 50.5% | 16.8% |
| Buffalo, NY | Blasdell village | 10.6% | 16.8% |
| Buffalo, NY | Buffalo city | 7.8% | 16.8% |
| Buffalo, NY | Cheektowaga CDP | 12.2% | 16.8% |
| Buffalo, NY | Clarence CDP | 62.6% | 16.8% |
| Buffalo, NY | Clarence Center CDP | 57.9% | 16.8% |
| Buffalo, NY | Depew village | 15.4% | 16.8% |
| Buffalo, NY | Eden CDP | 58.5% | 16.8% |
| Buffalo, NY | Eggertsville CDP | 18.9% | 16.8% |
| Buffalo, NY | Elma Center CDP | 67.4% | 16.8% |
| Buffalo, NY | Grandyle Village CDP | 26.7% | 16.8% |
| Buffalo, NY | Hamburg village | 33.0% | 16.8% |
| Buffalo, NY | Harris Hill CDP | 44.0% | 16.8% |
| Buffalo, NY | Kenmore village | 4.8% | 16.8% |
| Buffalo, NY | Lackawanna city | 11.2% | 16.8% |
| Buffalo, NY | Lake Erie Beach CDP | 43.1% | 16.8% |
| Buffalo, NY | Lancaster village | 24.3% | 16.8% |
| Buffalo, NY | Lewiston village | 35.5% | 16.8% |
| Buffalo, NY | Niagara Falls city | 11.0% | 16.8% |
| Buffalo, NY | North Boston CDP | 55.9% | 16.8% |
| Buffalo, NY | North Tonawanda city | 26.3% | 16.8% |
| Buffalo, NY | Orchard Park village | 51.0% | 16.8% |
| Buffalo, NY | Rapids CDP | 59.6% | 16.8% |
| Buffalo, NY | Sanborn CDP | 43.6% | 16.8% |
| Buffalo, NY | Sloan village | 6.9% | 16.8% |
| Buffalo, NY | Tonawanda CDP | 12.4% | 16.8% |
| Buffalo, NY | Tonawanda city | 18.0% | 16.8% |
| Buffalo, NY | Town Line CDP | 62.1% | 16.8% |
| Buffalo, NY | University at Buffalo CDP | 45.0% | 16.8% |
| Buffalo, NY | Wanakah CDP | 34.6% | 16.8% |
| Buffalo, NY | West Seneca CDP | 28.4% | 16.8% |
| Buffalo, NY | Williamsville village | 20.0% | 16.8% |
| Buffalo, NY | Youngstown village | 46.1% | 16.8% |
| Cape Coral, FL | Bonita Springs city | 42.9% | 19.0% |
| Cape Coral, FL | Buckingham CDP | 30.6% | 19.0% |
| Cape Coral, FL | Cape Coral city | 14.2% | 19.0% |
| Cape Coral, FL | Cypress Lake CDP | 17.8% | 19.0% |
| Cape Coral, FL | Estero village | 15.8% | 19.0% |
| Cape Coral, FL | Fort Myers Beach town | 4.8% | 19.0% |
| Cape Coral, FL | Fort Myers Shores CDP | 28.1% | 19.0% |
| Cape Coral, FL | Fort Myers city | 18.9% | 19.0% |
| Cape Coral, FL | Gateway CDP | 20.4% | 19.0% |
| Cape Coral, FL | Harlem Heights CDP | 16.3% | 19.0% |
| Cape Coral, FL | Iona CDP | 5.5% | 19.0% |
| Cape Coral, FL | Lehigh Acres CDP | 29.3% | 19.0% |
| Cape Coral, FL | Lochmoor Waterway Estates CDP | 21.2% | 19.0% |
| Cape Coral, FL | Matlacha CDP | 1.5% | 19.0% |
| Cape Coral, FL | Matlacha Isles-Matlacha Shores CDP | 8.6% | 19.0% |
| Cape Coral, FL | McGregor CDP | 17.4% | 19.0% |
| Cape Coral, FL | North Fort Myers CDP | 17.1% | 19.0% |
| Cape Coral, FL | Olga CDP | 22.8% | 19.0% |
| Cape Coral, FL | Page Park CDP | 43.1% | 19.0% |
| Cape Coral, FL | Palmona Park CDP | 31.7% | 19.0% |
| Cape Coral, FL | Pine Manor CDP | 27.1% | 19.0% |
| Cape Coral, FL | Punta Rassa CDP | 10.6% | 19.0% |
| Cape Coral, FL | San Carlos Park CDP | 18.2% | 19.0% |
| Cape Coral, FL | Sanibel city | 20.0% | 19.0% |
| Cape Coral, FL | Suncoast Estates CDP | 42.0% | 19.0% |
| Cape Coral, FL | Three Oaks CDP | 10.4% | 19.0% |
| Cape Coral, FL | Tice CDP | 39.2% | 19.0% |
| Cape Coral, FL | Villas CDP | 36.4% | 19.0% |
| Cape Coral, FL | Whiskey Creek CDP | 22.6% | 19.0% |
| Charleston--North Charleston, SC | Charleston city | 30.3% | 39.7% |
| Charleston--North Charleston, SC | Folly Beach city | 42.3% | 39.7% |
| Charleston--North Charleston, SC | Goose Creek city | 45.3% | 39.7% |
| Charleston--North Charleston, SC | Hanahan city | 44.8% | 39.7% |
| Charleston--North Charleston, SC | Hollywood town | 27.3% | 39.7% |
| Charleston--North Charleston, SC | Isle of Palms city | 43.3% | 39.7% |
| Charleston--North Charleston, SC | James Island town | 62.6% | 39.7% |
| Charleston--North Charleston, SC | Ladson CDP | 38.2% | 39.7% |
| Charleston--North Charleston, SC | Lincolnville town | 70.2% | 39.7% |
| Charleston--North Charleston, SC | Moncks Corner town | 38.2% | 39.7% |
| Charleston--North Charleston, SC | Mount Pleasant town | 30.7% | 39.7% |
| Charleston--North Charleston, SC | North Charleston city | 38.4% | 39.7% |
| Charleston--North Charleston, SC | Ravenel town | 50.8% | 39.7% |
| Charleston--North Charleston, SC | Sangaree CDP | 33.6% | 39.7% |
| Charleston--North Charleston, SC | Sullivan's Island town | 24.6% | 39.7% |
| Charleston--North Charleston, SC | Summerville town | 44.4% | 39.7% |
| Charlotte, NC--SC | Charlotte city | 55.8% | 55.7% |
| Charlotte, NC--SC | Concord city | 39.5% | 55.7% |
| Charlotte, NC--SC | Cornelius town | 46.8% | 55.7% |
| Charlotte, NC--SC | Davidson town | 53.1% | 55.7% |
| Charlotte, NC--SC | Fort Mill town | 48.8% | 55.7% |
| Charlotte, NC--SC | Harrisburg town | 8.1% | 55.7% |
| Charlotte, NC--SC | Hemby Bridge town | 62.6% | 55.7% |
| Charlotte, NC--SC | Huntersville town | 55.3% | 55.7% |
| Charlotte, NC--SC | Indian Trail town | 46.9% | 55.7% |
| Charlotte, NC--SC | Kannapolis city | 44.6% | 55.7% |
| Charlotte, NC--SC | Lake Norman of Catawba CDP | 53.2% | 55.7% |
| Charlotte, NC--SC | Lake Park village | 42.3% | 55.7% |
| Charlotte, NC--SC | Lake Wylie CDP | 59.8% | 55.7% |
| Charlotte, NC--SC | Lowesville CDP | 61.5% | 55.7% |
| Charlotte, NC--SC | Marshville town | 56.8% | 55.7% |
| Charlotte, NC--SC | Marvin village | 57.0% | 55.7% |
| Charlotte, NC--SC | Matthews town | 65.3% | 55.7% |
| Charlotte, NC--SC | Mineral Springs town | 78.9% | 55.7% |
| Charlotte, NC--SC | Mint Hill town | 68.9% | 55.7% |
| Charlotte, NC--SC | Monroe city | 50.1% | 55.7% |
| Charlotte, NC--SC | Mooresville town | 44.3% | 55.7% |
| Charlotte, NC--SC | Mount Holly city | 58.0% | 55.7% |
| Charlotte, NC--SC | Pineville town | 39.2% | 55.7% |
| Charlotte, NC--SC | Riverview CDP | 30.8% | 55.7% |
| Charlotte, NC--SC | Stallings town | 51.3% | 55.7% |
| Charlotte, NC--SC | Statesville city | 60.3% | 55.7% |
| Charlotte, NC--SC | Tega Cay city | 57.8% | 55.7% |
| Charlotte, NC--SC | Troutman town | 49.2% | 55.7% |
| Charlotte, NC--SC | Unionville town | 39.2% | 55.7% |
| Charlotte, NC--SC | Waxhaw town | 53.7% | 55.7% |
| Charlotte, NC--SC | Weddington town | 69.1% | 55.7% |
| Charlotte, NC--SC | Wesley Chapel village | 63.7% | 55.7% |
| Charlotte, NC--SC | Westport CDP | 63.4% | 55.7% |
| Charlotte, NC--SC | Wingate town | 55.1% | 55.7% |
| Chattanooga, TN--GA | Apison CDP | 73.1% | 61.6% |
| Chattanooga, TN--GA | Chattanooga Valley CDP | 64.2% | 61.6% |
| Chattanooga, TN--GA | Chattanooga city | 55.6% | 61.6% |
| Chattanooga, TN--GA | Chickamauga city | 56.9% | 61.6% |
| Chattanooga, TN--GA | Collegedale city | 64.5% | 61.6% |
| Chattanooga, TN--GA | East Ridge city | 54.0% | 61.6% |
| Chattanooga, TN--GA | Fairmount CDP | 83.2% | 61.6% |
| Chattanooga, TN--GA | Fairview CDP | 76.4% | 61.6% |
| Chattanooga, TN--GA | Falling Water CDP | 67.9% | 61.6% |
| Chattanooga, TN--GA | Fort Oglethorpe city | 40.7% | 61.6% |
| Chattanooga, TN--GA | Harrison CDP | 75.1% | 61.6% |
| Chattanooga, TN--GA | Indian Springs CDP | 75.8% | 61.6% |
| Chattanooga, TN--GA | Lakesite city | 70.6% | 61.6% |
| Chattanooga, TN--GA | Lakeview CDP | 62.8% | 61.6% |
| Chattanooga, TN--GA | Lone Oak CDP | 54.5% | 61.6% |
| Chattanooga, TN--GA | Lookout Mountain city | 78.8% | 61.6% |
| Chattanooga, TN--GA | Lookout Mountain town | 75.7% | 61.6% |
| Chattanooga, TN--GA | Middle Valley CDP | 63.5% | 61.6% |
| Chattanooga, TN--GA | Ooltewah CDP | 54.0% | 61.6% |
| Chattanooga, TN--GA | Red Bank city | 70.2% | 61.6% |
| Chattanooga, TN--GA | Ridgeside city | 62.5% | 61.6% |
| Chattanooga, TN--GA | Ringgold city | 28.8% | 61.6% |
| Chattanooga, TN--GA | Rossville city | 53.7% | 61.6% |
| Chattanooga, TN--GA | Signal Mountain town | 81.2% | 61.6% |
| Chattanooga, TN--GA | Soddy-Daisy city | 70.1% | 61.6% |
| Chattanooga, TN--GA | Walden town | 89.2% | 61.6% |
| Chicago, IL--IN | Aberdeen CDP | 23.9% | 27.1% |
| Chicago, IL--IN | Addison village | 19.9% | 27.1% |
| Chicago, IL--IN | Algonquin village | 39.2% | 27.1% |
| Chicago, IL--IN | Alsip village | 25.0% | 27.1% |
| Chicago, IL--IN | Arlington Heights village | 33.4% | 27.1% |
| Chicago, IL--IN | Aurora city | 28.4% | 27.1% |
| Chicago, IL--IN | Bannockburn village | 47.6% | 27.1% |
| Chicago, IL--IN | Barrington Hills village | 73.5% | 27.1% |
| Chicago, IL--IN | Barrington village | 52.5% | 27.1% |
| Chicago, IL--IN | Bartlett village | 27.9% | 27.1% |
| Chicago, IL--IN | Batavia city | 40.8% | 27.1% |
| Chicago, IL--IN | Beach Park village | 55.5% | 27.1% |
| Chicago, IL--IN | Bedford Park village | 22.5% | 27.1% |
| Chicago, IL--IN | Bellwood village | 26.9% | 27.1% |
| Chicago, IL--IN | Bensenville village | 29.5% | 27.1% |
| Chicago, IL--IN | Berkeley village | 32.4% | 27.1% |
| Chicago, IL--IN | Berwyn city | 19.7% | 27.1% |
| Chicago, IL--IN | Bloomingdale village | 30.2% | 27.1% |
| Chicago, IL--IN | Blue Island city | 27.4% | 27.1% |
| Chicago, IL--IN | Bolingbrook village | 26.8% | 27.1% |
| Chicago, IL--IN | Boulder Hill CDP | 43.0% | 27.1% |
| Chicago, IL--IN | Bridgeview village | 18.1% | 27.1% |
| Chicago, IL--IN | Broadview village | 28.7% | 27.1% |
| Chicago, IL--IN | Brookfield village | 29.8% | 27.1% |
| Chicago, IL--IN | Buffalo Grove village | 33.5% | 27.1% |
| Chicago, IL--IN | Bull Valley village | 61.9% | 27.1% |
| Chicago, IL--IN | Burbank city | 20.5% | 27.1% |
| Chicago, IL--IN | Burnham village | 13.2% | 27.1% |
| Chicago, IL--IN | Burns Harbor town | 32.4% | 27.1% |
| Chicago, IL--IN | Burr Ridge village | 55.1% | 27.1% |
| Chicago, IL--IN | Calumet City city | 12.6% | 27.1% |
| Chicago, IL--IN | Calumet Park village | 18.6% | 27.1% |
| Chicago, IL--IN | Campton Hills village | 51.2% | 27.1% |
| Chicago, IL--IN | Carol Stream village | 26.2% | 27.1% |
| Chicago, IL--IN | Carpentersville village | 37.0% | 27.1% |
| Chicago, IL--IN | Cary village | 43.5% | 27.1% |
| Chicago, IL--IN | Cedar Lake town | 43.2% | 27.1% |
| Chicago, IL--IN | Channahon village | 26.5% | 27.1% |
| Chicago, IL--IN | Chesterton town | 32.2% | 27.1% |
| Chicago, IL--IN | Chicago Heights city | 31.4% | 27.1% |
| Chicago, IL--IN | Chicago Ridge village | 22.0% | 27.1% |
| Chicago, IL--IN | Chicago city | 20.7% | 27.1% |
| Chicago, IL--IN | Cicero town | 18.6% | 27.1% |
| Chicago, IL--IN | Clarendon Hills village | 54.9% | 27.1% |
| Chicago, IL--IN | Country Club Hills city | 34.8% | 27.1% |
| Chicago, IL--IN | Countryside city | 39.6% | 27.1% |
| Chicago, IL--IN | Crest Hill city | 23.0% | 27.1% |
| Chicago, IL--IN | Crestwood village | 30.3% | 27.1% |
| Chicago, IL--IN | Crete village | 50.4% | 27.1% |
| Chicago, IL--IN | Crown Point city | 25.3% | 27.1% |
| Chicago, IL--IN | Crystal Lake city | 39.3% | 27.1% |
| Chicago, IL--IN | Crystal Lawns CDP | 41.7% | 27.1% |
| Chicago, IL--IN | Darien city | 37.6% | 27.1% |
| Chicago, IL--IN | Deer Park village | 63.9% | 27.1% |
| Chicago, IL--IN | Deerfield village | 52.1% | 27.1% |
| Chicago, IL--IN | Des Plaines city | 29.9% | 27.1% |
| Chicago, IL--IN | Dixmoor village | 29.8% | 27.1% |
| Chicago, IL--IN | Dolton village | 27.2% | 27.1% |
| Chicago, IL--IN | Downers Grove village | 45.9% | 27.1% |
| Chicago, IL--IN | Dyer town | 20.7% | 27.1% |
| Chicago, IL--IN | East Chicago city | 14.2% | 27.1% |
| Chicago, IL--IN | East Dundee village | 46.9% | 27.1% |
| Chicago, IL--IN | East Hazel Crest village | 47.8% | 27.1% |
| Chicago, IL--IN | Elburn village | 23.7% | 27.1% |
| Chicago, IL--IN | Elgin city | 34.3% | 27.1% |
| Chicago, IL--IN | Elk Grove Village village | 19.9% | 27.1% |
| Chicago, IL--IN | Elmhurst city | 41.3% | 27.1% |
| Chicago, IL--IN | Elmwood Park village | 21.4% | 27.1% |
| Chicago, IL--IN | Elwood village | 23.8% | 27.1% |
| Chicago, IL--IN | Evanston city | 43.3% | 27.1% |
| Chicago, IL--IN | Evergreen Park village | 26.9% | 27.1% |
| Chicago, IL--IN | Fairmont CDP | 42.4% | 27.1% |
| Chicago, IL--IN | Flossmoor village | 58.8% | 27.1% |
| Chicago, IL--IN | Ford Heights village | 27.0% | 27.1% |
| Chicago, IL--IN | Forest Lake CDP | 40.9% | 27.1% |
| Chicago, IL--IN | Forest Park village | 28.6% | 27.1% |
| Chicago, IL--IN | Forest View village | 35.7% | 27.1% |
| Chicago, IL--IN | Fox River Grove village | 54.0% | 27.1% |
| Chicago, IL--IN | Frankfort Square CDP | 33.7% | 27.1% |
| Chicago, IL--IN | Frankfort village | 30.9% | 27.1% |
| Chicago, IL--IN | Franklin Park village | 22.6% | 27.1% |
| Chicago, IL--IN | Gary city | 30.5% | 27.1% |
| Chicago, IL--IN | Geneva city | 39.8% | 27.1% |
| Chicago, IL--IN | Gilberts village | 24.6% | 27.1% |
| Chicago, IL--IN | Glen Ellyn village | 48.7% | 27.1% |
| Chicago, IL--IN | Glencoe village | 67.4% | 27.1% |
| Chicago, IL--IN | Glendale Heights village | 22.3% | 27.1% |
| Chicago, IL--IN | Glenview village | 46.6% | 27.1% |
| Chicago, IL--IN | Glenwood village | 40.0% | 27.1% |
| Chicago, IL--IN | Golf village | 62.6% | 27.1% |
| Chicago, IL--IN | Green Oaks village | 50.0% | 27.1% |
| Chicago, IL--IN | Griffith town | 29.4% | 27.1% |
| Chicago, IL--IN | Gurnee village | 41.3% | 27.1% |
| Chicago, IL--IN | Hammond city | 17.8% | 27.1% |
| Chicago, IL--IN | Hampshire village | 29.9% | 27.1% |
| Chicago, IL--IN | Hanover Park village | 28.7% | 27.1% |
| Chicago, IL--IN | Harvey city | 33.7% | 27.1% |
| Chicago, IL--IN | Harwood Heights village | 14.3% | 27.1% |
| Chicago, IL--IN | Hawthorn Woods village | 43.4% | 27.1% |
| Chicago, IL--IN | Hazel Crest village | 45.0% | 27.1% |
| Chicago, IL--IN | Hickory Hills city | 29.8% | 27.1% |
| Chicago, IL--IN | Highland Park city | 62.4% | 27.1% |
| Chicago, IL--IN | Highland town | 24.7% | 27.1% |
| Chicago, IL--IN | Highwood city | 36.8% | 27.1% |
| Chicago, IL--IN | Hillside village | 29.5% | 27.1% |
| Chicago, IL--IN | Hinsdale village | 57.8% | 27.1% |
| Chicago, IL--IN | Hobart city | 26.8% | 27.1% |
| Chicago, IL--IN | Hodgkins village | 14.0% | 27.1% |
| Chicago, IL--IN | Hoffman Estates village | 30.1% | 27.1% |
| Chicago, IL--IN | Holiday Hills village | 46.9% | 27.1% |
| Chicago, IL--IN | Homer Glen village | 38.0% | 27.1% |
| Chicago, IL--IN | Hometown city | 22.2% | 27.1% |
| Chicago, IL--IN | Homewood village | 53.1% | 27.1% |
| Chicago, IL--IN | Huntley village | 21.1% | 27.1% |
| Chicago, IL--IN | Indian Creek village | 39.0% | 27.1% |
| Chicago, IL--IN | Indian Head Park village | 44.4% | 27.1% |
| Chicago, IL--IN | Ingalls Park CDP | 39.2% | 27.1% |
| Chicago, IL--IN | Inverness village | 62.1% | 27.1% |
| Chicago, IL--IN | Island Lake village | 43.7% | 27.1% |
| Chicago, IL--IN | Itasca village | 29.3% | 27.1% |
| Chicago, IL--IN | Joliet city | 21.0% | 27.1% |
| Chicago, IL--IN | Justice village | 21.2% | 27.1% |
| Chicago, IL--IN | Kenilworth village | 58.4% | 27.1% |
| Chicago, IL--IN | Kildeer village | 45.5% | 27.1% |
| Chicago, IL--IN | Knollwood CDP | 57.7% | 27.1% |
| Chicago, IL--IN | La Grange Park village | 45.6% | 27.1% |
| Chicago, IL--IN | La Grange village | 48.2% | 27.1% |
| Chicago, IL--IN | Lake Barrington village | 54.9% | 27.1% |
| Chicago, IL--IN | Lake Bluff village | 69.1% | 27.1% |
| Chicago, IL--IN | Lake Dalecarlia CDP | 43.8% | 27.1% |
| Chicago, IL--IN | Lake Forest city | 67.7% | 27.1% |
| Chicago, IL--IN | Lake Station city | 36.3% | 27.1% |
| Chicago, IL--IN | Lake Zurich village | 43.3% | 27.1% |
| Chicago, IL--IN | Lake in the Hills village | 27.6% | 27.1% |
| Chicago, IL--IN | Lakes of the Four Seasons CDP | 25.6% | 27.1% |
| Chicago, IL--IN | Lakewood village | 47.5% | 27.1% |
| Chicago, IL--IN | Lansing village | 19.6% | 27.1% |
| Chicago, IL--IN | Lemont village | 30.1% | 27.1% |
| Chicago, IL--IN | Libertyville village | 47.7% | 27.1% |
| Chicago, IL--IN | Lily Lake village | 39.8% | 27.1% |
| Chicago, IL--IN | Lincolnshire village | 63.1% | 27.1% |
| Chicago, IL--IN | Lincolnwood village | 36.6% | 27.1% |
| Chicago, IL--IN | Lisle village | 41.7% | 27.1% |
| Chicago, IL--IN | Lockport city | 19.9% | 27.1% |
| Chicago, IL--IN | Lombard village | 33.1% | 27.1% |
| Chicago, IL--IN | Long Grove village | 48.9% | 27.1% |
| Chicago, IL--IN | Lynwood village | 14.4% | 27.1% |
| Chicago, IL--IN | Lyons village | 28.5% | 27.1% |
| Chicago, IL--IN | Manhattan village | 13.6% | 27.1% |
| Chicago, IL--IN | Markham city | 46.6% | 27.1% |
| Chicago, IL--IN | Matteson village | 30.4% | 27.1% |
| Chicago, IL--IN | Maywood village | 25.1% | 27.1% |
| Chicago, IL--IN | McCook village | 12.4% | 27.1% |
| Chicago, IL--IN | Melrose Park village | 16.9% | 27.1% |
| Chicago, IL--IN | Merrillville town | 30.1% | 27.1% |
| Chicago, IL--IN | Merrionette Park village | 23.4% | 27.1% |
| Chicago, IL--IN | Mettawa village | 53.2% | 27.1% |
| Chicago, IL--IN | Midlothian village | 39.9% | 27.1% |
| Chicago, IL--IN | Minooka village | 19.2% | 27.1% |
| Chicago, IL--IN | Mokena village | 29.3% | 27.1% |
| Chicago, IL--IN | Monee village | 28.4% | 27.1% |
| Chicago, IL--IN | Montgomery village | 14.8% | 27.1% |
| Chicago, IL--IN | Morton Grove village | 31.4% | 27.1% |
| Chicago, IL--IN | Mount Prospect village | 35.8% | 27.1% |
| Chicago, IL--IN | Mundelein village | 32.1% | 27.1% |
| Chicago, IL--IN | Munster town | 27.3% | 27.1% |
| Chicago, IL--IN | Naperville city | 35.9% | 27.1% |
| Chicago, IL--IN | New Chicago town | 37.5% | 27.1% |
| Chicago, IL--IN | New Lenox village | 25.5% | 27.1% |
| Chicago, IL--IN | Niles village | 24.8% | 27.1% |
| Chicago, IL--IN | Norridge village | 15.3% | 27.1% |
| Chicago, IL--IN | North Aurora village | 25.5% | 27.1% |
| Chicago, IL--IN | North Barrington village | 75.0% | 27.1% |
| Chicago, IL--IN | North Chicago city | 23.5% | 27.1% |
| Chicago, IL--IN | North Riverside village | 28.2% | 27.1% |
| Chicago, IL--IN | Northbrook village | 46.4% | 27.1% |
| Chicago, IL--IN | Northfield village | 62.1% | 27.1% |
| Chicago, IL--IN | Northlake city | 27.4% | 27.1% |
| Chicago, IL--IN | Oak Brook village | 47.5% | 27.1% |
| Chicago, IL--IN | Oak Forest city | 33.5% | 27.1% |
| Chicago, IL--IN | Oak Lawn village | 27.9% | 27.1% |
| Chicago, IL--IN | Oak Park village | 34.3% | 27.1% |
| Chicago, IL--IN | Oakbrook Terrace city | 26.0% | 27.1% |
| Chicago, IL--IN | Oakwood Hills village | 55.5% | 27.1% |
| Chicago, IL--IN | Ogden Dunes town | 58.1% | 27.1% |
| Chicago, IL--IN | Olympia Fields village | 57.4% | 27.1% |
| Chicago, IL--IN | Orland Hills village | 14.4% | 27.1% |
| Chicago, IL--IN | Orland Park village | 30.9% | 27.1% |
| Chicago, IL--IN | Oswego village | 21.5% | 27.1% |
| Chicago, IL--IN | Palatine village | 28.5% | 27.1% |
| Chicago, IL--IN | Palos Heights city | 46.6% | 27.1% |
| Chicago, IL--IN | Palos Hills city | 34.1% | 27.1% |
| Chicago, IL--IN | Palos Park village | 70.0% | 27.1% |
| Chicago, IL--IN | Park City city | 22.7% | 27.1% |
| Chicago, IL--IN | Park Forest village | 50.3% | 27.1% |
| Chicago, IL--IN | Park Ridge city | 38.0% | 27.1% |
| Chicago, IL--IN | Phoenix village | 24.2% | 27.1% |
| Chicago, IL--IN | Pingree Grove village | 14.8% | 27.1% |
| Chicago, IL--IN | Plainfield village | 19.2% | 27.1% |
| Chicago, IL--IN | Plano city | 21.7% | 27.1% |
| Chicago, IL--IN | Port Barrington village | 42.3% | 27.1% |
| Chicago, IL--IN | Portage city | 31.4% | 27.1% |
| Chicago, IL--IN | Porter town | 39.2% | 27.1% |
| Chicago, IL--IN | Posen village | 23.4% | 27.1% |
| Chicago, IL--IN | Prairie Grove village | 64.3% | 27.1% |
| Chicago, IL--IN | Prestbury CDP | 48.7% | 27.1% |
| Chicago, IL--IN | Preston Heights CDP | 37.9% | 27.1% |
| Chicago, IL--IN | Prospect Heights city | 29.3% | 27.1% |
| Chicago, IL--IN | Richton Park village | 35.8% | 27.1% |
| Chicago, IL--IN | River Forest village | 53.3% | 27.1% |
| Chicago, IL--IN | River Grove village | 16.9% | 27.1% |
| Chicago, IL--IN | Riverdale village | 28.6% | 27.1% |
| Chicago, IL--IN | Riverside village | 56.5% | 27.1% |
| Chicago, IL--IN | Riverwoods village | 66.5% | 27.1% |
| Chicago, IL--IN | Robbins village | 40.5% | 27.1% |
| Chicago, IL--IN | Rockdale village | 22.7% | 27.1% |
| Chicago, IL--IN | Rolling Meadows city | 31.1% | 27.1% |
| Chicago, IL--IN | Romeoville village | 18.4% | 27.1% |
| Chicago, IL--IN | Roselle village | 32.4% | 27.1% |
| Chicago, IL--IN | Rosemont village | 7.7% | 27.1% |
| Chicago, IL--IN | Salt Creek Commons CDP | 20.2% | 27.1% |
| Chicago, IL--IN | Sandwich city | 32.7% | 27.1% |
| Chicago, IL--IN | Sauk Village village | 34.5% | 27.1% |
| Chicago, IL--IN | Schaumburg village | 30.5% | 27.1% |
| Chicago, IL--IN | Schererville town | 28.1% | 27.1% |
| Chicago, IL--IN | Schiller Park village | 15.2% | 27.1% |
| Chicago, IL--IN | Shorewood village | 21.3% | 27.1% |
| Chicago, IL--IN | Skokie village | 32.3% | 27.1% |
| Chicago, IL--IN | Sleepy Hollow village | 56.6% | 27.1% |
| Chicago, IL--IN | South Barrington village | 48.7% | 27.1% |
| Chicago, IL--IN | South Chicago Heights village | 24.7% | 27.1% |
| Chicago, IL--IN | South Elgin village | 24.0% | 27.1% |
| Chicago, IL--IN | South Haven CDP | 30.9% | 27.1% |
| Chicago, IL--IN | South Holland village | 25.1% | 27.1% |
| Chicago, IL--IN | St. Charles city | 39.1% | 27.1% |
| Chicago, IL--IN | St. John town | 32.4% | 27.1% |
| Chicago, IL--IN | Steger village | 39.8% | 27.1% |
| Chicago, IL--IN | Stickney village | 22.4% | 27.1% |
| Chicago, IL--IN | Stone Park village | 16.6% | 27.1% |
| Chicago, IL--IN | Streamwood village | 29.4% | 27.1% |
| Chicago, IL--IN | Sugar Grove village | 23.8% | 27.1% |
| Chicago, IL--IN | Summit village | 17.8% | 27.1% |
| Chicago, IL--IN | Thornton village | 34.3% | 27.1% |
| Chicago, IL--IN | Tinley Park village | 22.0% | 27.1% |
| Chicago, IL--IN | Tower Lakes village | 72.7% | 27.1% |
| Chicago, IL--IN | Trout Valley village | 82.6% | 27.1% |
| Chicago, IL--IN | University Park village | 46.4% | 27.1% |
| Chicago, IL--IN | Valparaiso city | 24.4% | 27.1% |
| Chicago, IL--IN | Vernon Hills village | 30.3% | 27.1% |
| Chicago, IL--IN | Villa Park village | 37.1% | 27.1% |
| Chicago, IL--IN | Wadsworth village | 58.9% | 27.1% |
| Chicago, IL--IN | Warrenville city | 37.4% | 27.1% |
| Chicago, IL--IN | Wauconda village | 40.1% | 27.1% |
| Chicago, IL--IN | Waukegan city | 35.4% | 27.1% |
| Chicago, IL--IN | Wayne village | 59.9% | 27.1% |
| Chicago, IL--IN | West Chicago city | 34.2% | 27.1% |
| Chicago, IL--IN | West Dundee village | 39.2% | 27.1% |
| Chicago, IL--IN | Westchester village | 33.3% | 27.1% |
| Chicago, IL--IN | Western Springs village | 50.4% | 27.1% |
| Chicago, IL--IN | Westmont village | 32.4% | 27.1% |
| Chicago, IL--IN | Wheaton city | 45.6% | 27.1% |
| Chicago, IL--IN | Wheeler CDP | 21.6% | 27.1% |
| Chicago, IL--IN | Wheeling village | 28.8% | 27.1% |
| Chicago, IL--IN | Whiting city | 10.1% | 27.1% |
| Chicago, IL--IN | Willow Springs village | 49.4% | 27.1% |
| Chicago, IL--IN | Willowbrook CDP | 36.0% | 27.1% |
| Chicago, IL--IN | Willowbrook village | 36.0% | 27.1% |
| Chicago, IL--IN | Wilmette village | 57.3% | 27.1% |
| Chicago, IL--IN | Winfield town | 21.1% | 27.1% |
| Chicago, IL--IN | Winfield village | 36.9% | 27.1% |
| Chicago, IL--IN | Winnetka village | 68.9% | 27.1% |
| Chicago, IL--IN | Winthrop Harbor village | 50.2% | 27.1% |
| Chicago, IL--IN | Wood Dale city | 27.4% | 27.1% |
| Chicago, IL--IN | Woodridge village | 33.6% | 27.1% |
| Chicago, IL--IN | Worth village | 38.8% | 27.1% |
| Chicago, IL--IN | Yorkville city | 20.2% | 27.1% |
| Chicago, IL--IN | Zion city | 38.4% | 27.1% |
| Cincinnati, OH--KY--IN | Addyston village | 52.6% | 46.3% |
| Cincinnati, OH--KY--IN | Alexandria city | 59.1% | 46.3% |
| Cincinnati, OH--KY--IN | Amberley village | 64.5% | 46.3% |
| Cincinnati, OH--KY--IN | Amelia village | 60.4% | 46.3% |
| Cincinnati, OH--KY--IN | Arlington Heights village | 38.6% | 46.3% |
| Cincinnati, OH--KY--IN | Batavia village | 43.6% | 46.3% |
| Cincinnati, OH--KY--IN | Beckett Ridge CDP | 55.4% | 46.3% |
| Cincinnati, OH--KY--IN | Bellevue city | 12.2% | 46.3% |
| Cincinnati, OH--KY--IN | Blue Ash city | 53.0% | 46.3% |
| Cincinnati, OH--KY--IN | Brecon CDP | 35.5% | 46.3% |
| Cincinnati, OH--KY--IN | Bridgetown CDP | 40.1% | 46.3% |
| Cincinnati, OH--KY--IN | Bright CDP | 76.3% | 46.3% |
| Cincinnati, OH--KY--IN | Bromley city | 26.1% | 46.3% |
| Cincinnati, OH--KY--IN | Burlington CDP | 35.5% | 46.3% |
| Cincinnati, OH--KY--IN | Camp Dennison CDP | 66.2% | 46.3% |
| Cincinnati, OH--KY--IN | Cherry Grove CDP | 36.4% | 46.3% |
| Cincinnati, OH--KY--IN | Cheviot city | 31.1% | 46.3% |
| Cincinnati, OH--KY--IN | Cincinnati city | 44.6% | 46.3% |
| Cincinnati, OH--KY--IN | Claryville CDP | 46.9% | 46.3% |
| Cincinnati, OH--KY--IN | Cleves village | 59.4% | 46.3% |
| Cincinnati, OH--KY--IN | Cold Spring city | 47.5% | 46.3% |
| Cincinnati, OH--KY--IN | Coldstream CDP | 64.1% | 46.3% |
| Cincinnati, OH--KY--IN | Concorde Hills CDP | 73.2% | 46.3% |
| Cincinnati, OH--KY--IN | Covedale CDP | 49.4% | 46.3% |
| Cincinnati, OH--KY--IN | Covington city | 30.5% | 46.3% |
| Cincinnati, OH--KY--IN | Crescent Springs city | 46.8% | 46.3% |
| Cincinnati, OH--KY--IN | Crestview Hills city | 39.4% | 46.3% |
| Cincinnati, OH--KY--IN | Crestview city | 29.1% | 46.3% |
| Cincinnati, OH--KY--IN | Day Heights CDP | 60.2% | 46.3% |
| Cincinnati, OH--KY--IN | Dayton city | 14.2% | 46.3% |
| Cincinnati, OH--KY--IN | Deer Park city | 40.8% | 46.3% |
| Cincinnati, OH--KY--IN | Delhi Hills CDP | 35.1% | 46.3% |
| Cincinnati, OH--KY--IN | Delshire CDP | 30.2% | 46.3% |
| Cincinnati, OH--KY--IN | Dent CDP | 61.6% | 46.3% |
| Cincinnati, OH--KY--IN | Dillonvale CDP | 54.9% | 46.3% |
| Cincinnati, OH--KY--IN | Dry Ridge CDP | 63.9% | 46.3% |
| Cincinnati, OH--KY--IN | Dry Run CDP | 66.8% | 46.3% |
| Cincinnati, OH--KY--IN | Dunlap CDP | 82.5% | 46.3% |
| Cincinnati, OH--KY--IN | Edgewood city | 50.3% | 46.3% |
| Cincinnati, OH--KY--IN | Elizabethtown CDP | 33.5% | 46.3% |
| Cincinnati, OH--KY--IN | Elmwood Place village | 33.0% | 46.3% |
| Cincinnati, OH--KY--IN | Elsmere city | 37.2% | 46.3% |
| Cincinnati, OH--KY--IN | Erlanger city | 37.7% | 46.3% |
| Cincinnati, OH--KY--IN | Evendale village | 61.7% | 46.3% |
| Cincinnati, OH--KY--IN | Fairfax village | 40.8% | 46.3% |
| Cincinnati, OH--KY--IN | Fairfield city | 45.3% | 46.3% |
| Cincinnati, OH--KY--IN | Fairview city | 71.9% | 46.3% |
| Cincinnati, OH--KY--IN | Finneytown CDP | 52.9% | 46.3% |
| Cincinnati, OH--KY--IN | Florence city | 30.5% | 46.3% |
| Cincinnati, OH--KY--IN | Forest Park city | 37.6% | 46.3% |
| Cincinnati, OH--KY--IN | Forestville CDP | 52.6% | 46.3% |
| Cincinnati, OH--KY--IN | Fort Mitchell city | 41.6% | 46.3% |
| Cincinnati, OH--KY--IN | Fort Thomas city | 50.5% | 46.3% |
| Cincinnati, OH--KY--IN | Fort Wright city | 49.4% | 46.3% |
| Cincinnati, OH--KY--IN | Four Bridges CDP | 40.0% | 46.3% |
| Cincinnati, OH--KY--IN | Francisville CDP | 55.7% | 46.3% |
| Cincinnati, OH--KY--IN | Fruit Hill CDP | 63.3% | 46.3% |
| Cincinnati, OH--KY--IN | Glendale village | 64.3% | 46.3% |
| Cincinnati, OH--KY--IN | Golf Manor village | 44.1% | 46.3% |
| Cincinnati, OH--KY--IN | Goshen CDP | 59.6% | 46.3% |
| Cincinnati, OH--KY--IN | Grandview CDP | 70.6% | 46.3% |
| Cincinnati, OH--KY--IN | Greenhills village | 57.9% | 46.3% |
| Cincinnati, OH--KY--IN | Groesbeck CDP | 47.2% | 46.3% |
| Cincinnati, OH--KY--IN | Hamilton city | 30.5% | 46.3% |
| Cincinnati, OH--KY--IN | Hebron CDP | 38.4% | 46.3% |
| Cincinnati, OH--KY--IN | Hidden Valley CDP | 78.2% | 46.3% |
| Cincinnati, OH--KY--IN | Highland Heights city | 44.0% | 46.3% |
| Cincinnati, OH--KY--IN | Highpoint CDP | 56.0% | 46.3% |
| Cincinnati, OH--KY--IN | Hooven CDP | 17.6% | 46.3% |
| Cincinnati, OH--KY--IN | Independence city | 47.3% | 46.3% |
| Cincinnati, OH--KY--IN | Kenton Vale city | 69.8% | 46.3% |
| Cincinnati, OH--KY--IN | Kenwood CDP | 55.0% | 46.3% |
| Cincinnati, OH--KY--IN | Kings Mills CDP | 47.7% | 46.3% |
| Cincinnati, OH--KY--IN | Lakeside Park city | 49.4% | 46.3% |
| Cincinnati, OH--KY--IN | Landen CDP | 53.0% | 46.3% |
| Cincinnati, OH--KY--IN | Lebanon city | 40.9% | 46.3% |
| Cincinnati, OH--KY--IN | Lincoln Heights village | 42.4% | 46.3% |
| Cincinnati, OH--KY--IN | Lockland village | 47.2% | 46.3% |
| Cincinnati, OH--KY--IN | Loveland Park CDP | 73.6% | 46.3% |
| Cincinnati, OH--KY--IN | Loveland city | 52.0% | 46.3% |
| Cincinnati, OH--KY--IN | Ludlow city | 10.5% | 46.3% |
| Cincinnati, OH--KY--IN | Mack CDP | 63.9% | 46.3% |
| Cincinnati, OH--KY--IN | Madeira city | 64.1% | 46.3% |
| Cincinnati, OH--KY--IN | Maineville village | 48.5% | 46.3% |
| Cincinnati, OH--KY--IN | Mariemont village | 64.7% | 46.3% |
| Cincinnati, OH--KY--IN | Mason city | 39.5% | 46.3% |
| Cincinnati, OH--KY--IN | Melbourne city | 50.9% | 46.3% |
| Cincinnati, OH--KY--IN | Miami Heights CDP | 61.2% | 46.3% |
| Cincinnati, OH--KY--IN | Miamitown CDP | 65.9% | 46.3% |
| Cincinnati, OH--KY--IN | Miamiville CDP | 65.4% | 46.3% |
| Cincinnati, OH--KY--IN | Middletown city | 0.0% | 46.3% |
| Cincinnati, OH--KY--IN | Milford city | 52.4% | 46.3% |
| Cincinnati, OH--KY--IN | Millville village | 47.1% | 46.3% |
| Cincinnati, OH--KY--IN | Monfort Heights CDP | 60.1% | 46.3% |
| Cincinnati, OH--KY--IN | Monroe city | 44.3% | 46.3% |
| Cincinnati, OH--KY--IN | Montgomery city | 62.9% | 46.3% |
| Cincinnati, OH--KY--IN | Morrow village | 55.7% | 46.3% |
| Cincinnati, OH--KY--IN | Mount Carmel CDP | 52.9% | 46.3% |
| Cincinnati, OH--KY--IN | Mount Healthy Heights CDP | 40.1% | 46.3% |
| Cincinnati, OH--KY--IN | Mount Healthy city | 43.4% | 46.3% |
| Cincinnati, OH--KY--IN | Mount Repose CDP | 55.5% | 46.3% |
| Cincinnati, OH--KY--IN | Mulberry CDP | 58.5% | 46.3% |
| Cincinnati, OH--KY--IN | New Baltimore CDP | 39.6% | 46.3% |
| Cincinnati, OH--KY--IN | New Burlington CDP | 60.5% | 46.3% |
| Cincinnati, OH--KY--IN | New Miami village | 38.8% | 46.3% |
| Cincinnati, OH--KY--IN | Newport city | 17.8% | 46.3% |
| Cincinnati, OH--KY--IN | Newtown village | 57.7% | 46.3% |
| Cincinnati, OH--KY--IN | North Bend village | 40.4% | 46.3% |
| Cincinnati, OH--KY--IN | North College Hill city | 42.1% | 46.3% |
| Cincinnati, OH--KY--IN | Northbrook CDP | 41.1% | 46.3% |
| Cincinnati, OH--KY--IN | Northgate CDP | 39.3% | 46.3% |
| Cincinnati, OH--KY--IN | Norwood city | 35.4% | 46.3% |
| Cincinnati, OH--KY--IN | Oakbrook CDP | 39.2% | 46.3% |
| Cincinnati, OH--KY--IN | Olde West Chester CDP | 63.8% | 46.3% |
| Cincinnati, OH--KY--IN | Owensville village | 56.1% | 46.3% |
| Cincinnati, OH--KY--IN | Park Hills city | 46.2% | 46.3% |
| Cincinnati, OH--KY--IN | Plainville CDP | 57.1% | 46.3% |
| Cincinnati, OH--KY--IN | Pleasant Hills CDP | 54.9% | 46.3% |
| Cincinnati, OH--KY--IN | Pleasant Run CDP | 39.2% | 46.3% |
| Cincinnati, OH--KY--IN | Pleasant Run Farm CDP | 44.0% | 46.3% |
| Cincinnati, OH--KY--IN | Reading city | 50.0% | 46.3% |
| Cincinnati, OH--KY--IN | Remington CDP | 66.4% | 46.3% |
| Cincinnati, OH--KY--IN | Ross CDP | 44.7% | 46.3% |
| Cincinnati, OH--KY--IN | Rossmoyne CDP | 48.9% | 46.3% |
| Cincinnati, OH--KY--IN | Ryland Heights city | 19.6% | 46.3% |
| Cincinnati, OH--KY--IN | Salem Heights CDP | 64.2% | 46.3% |
| Cincinnati, OH--KY--IN | Seven Mile village | 50.6% | 46.3% |
| Cincinnati, OH--KY--IN | Sharonville city | 51.4% | 46.3% |
| Cincinnati, OH--KY--IN | Sherwood CDP | 62.0% | 46.3% |
| Cincinnati, OH--KY--IN | Silver Grove city | 39.0% | 46.3% |
| Cincinnati, OH--KY--IN | Silverton village | 51.1% | 46.3% |
| Cincinnati, OH--KY--IN | Sixteen Mile Stand CDP | 58.2% | 46.3% |
| Cincinnati, OH--KY--IN | Skyline Acres CDP | 52.8% | 46.3% |
| Cincinnati, OH--KY--IN | South Lebanon village | 44.7% | 46.3% |
| Cincinnati, OH--KY--IN | Southgate city | 54.0% | 46.3% |
| Cincinnati, OH--KY--IN | Springdale city | 41.0% | 46.3% |
| Cincinnati, OH--KY--IN | St. Bernard village | 37.3% | 46.3% |
| Cincinnati, OH--KY--IN | Summerside CDP | 53.7% | 46.3% |
| Cincinnati, OH--KY--IN | Taylor Creek CDP | 67.5% | 46.3% |
| Cincinnati, OH--KY--IN | Taylor Mill city | 58.4% | 46.3% |
| Cincinnati, OH--KY--IN | Terrace Park village | 71.2% | 46.3% |
| Cincinnati, OH--KY--IN | The Village of Indian Hill city | 80.3% | 46.3% |
| Cincinnati, OH--KY--IN | Turpin Hills CDP | 68.8% | 46.3% |
| Cincinnati, OH--KY--IN | Union city | 40.4% | 46.3% |
| Cincinnati, OH--KY--IN | Villa Hills city | 41.6% | 46.3% |
| Cincinnati, OH--KY--IN | Walton city | 34.8% | 46.3% |
| Cincinnati, OH--KY--IN | Wetherington CDP | 43.5% | 46.3% |
| Cincinnati, OH--KY--IN | White Oak CDP | 46.6% | 46.3% |
| Cincinnati, OH--KY--IN | Wilder city | 57.3% | 46.3% |
| Cincinnati, OH--KY--IN | Williamsburg village | 49.4% | 46.3% |
| Cincinnati, OH--KY--IN | Williamsdale CDP | 41.1% | 46.3% |
| Cincinnati, OH--KY--IN | Withamsville CDP | 53.3% | 46.3% |
| Cincinnati, OH--KY--IN | Woodlawn city | 50.6% | 46.3% |
| Cincinnati, OH--KY--IN | Woodlawn village | 59.3% | 46.3% |
| Cincinnati, OH--KY--IN | Wyoming city | 66.5% | 46.3% |
| Cleveland, OH | Aurora city | 39.4% | 21.3% |
| Cleveland, OH | Avon Lake city | 29.5% | 21.3% |
| Cleveland, OH | Avon city | 21.2% | 21.3% |
| Cleveland, OH | Bainbridge CDP | 42.4% | 21.3% |
| Cleveland, OH | Bay Village city | 38.4% | 21.3% |
| Cleveland, OH | Beachwood city | 22.7% | 21.3% |
| Cleveland, OH | Bedford Heights city | 18.6% | 21.3% |
| Cleveland, OH | Bedford city | 13.9% | 21.3% |
| Cleveland, OH | Bentleyville village | 37.6% | 21.3% |
| Cleveland, OH | Berea city | 22.8% | 21.3% |
| Cleveland, OH | Boston Heights village | 44.1% | 21.3% |
| Cleveland, OH | Bratenahl village | 36.3% | 21.3% |
| Cleveland, OH | Brecksville city | 45.4% | 21.3% |
| Cleveland, OH | Broadview Heights city | 38.6% | 21.3% |
| Cleveland, OH | Brook Park city | 12.4% | 21.3% |
| Cleveland, OH | Brooklyn Heights village | 20.8% | 21.3% |
| Cleveland, OH | Brooklyn city | 12.6% | 21.3% |
| Cleveland, OH | Brunswick city | 29.0% | 21.3% |
| Cleveland, OH | Chagrin Falls village | 29.3% | 21.3% |
| Cleveland, OH | Chesterland CDP | 44.8% | 21.3% |
| Cleveland, OH | Cleveland Heights city | 14.9% | 21.3% |
| Cleveland, OH | Cleveland city | 12.3% | 21.3% |
| Cleveland, OH | Cuyahoga Heights village | 33.0% | 21.3% |
| Cleveland, OH | East Cleveland city | 11.8% | 21.3% |
| Cleveland, OH | Eastlake city | 15.1% | 21.3% |
| Cleveland, OH | Euclid city | 17.6% | 21.3% |
| Cleveland, OH | Fairport Harbor village | 17.1% | 21.3% |
| Cleveland, OH | Fairview Park city | 31.2% | 21.3% |
| Cleveland, OH | Garfield Heights city | 7.1% | 21.3% |
| Cleveland, OH | Gates Mills village | 46.4% | 21.3% |
| Cleveland, OH | Glenwillow village | 38.6% | 21.3% |
| Cleveland, OH | Grand River village | 25.2% | 21.3% |
| Cleveland, OH | Highland Heights city | 29.2% | 21.3% |
| Cleveland, OH | Highland Hills village | 18.6% | 21.3% |
| Cleveland, OH | Hudson city | 38.8% | 21.3% |
| Cleveland, OH | Hunting Valley village | 33.2% | 21.3% |
| Cleveland, OH | Independence city | 39.6% | 21.3% |
| Cleveland, OH | Kirtland city | 46.9% | 21.3% |
| Cleveland, OH | Lakeline village | 28.1% | 21.3% |
| Cleveland, OH | Lakewood city | 12.7% | 21.3% |
| Cleveland, OH | Linndale village | 6.8% | 21.3% |
| Cleveland, OH | Lyndhurst city | 28.0% | 21.3% |
| Cleveland, OH | Macedonia city | 31.0% | 21.3% |
| Cleveland, OH | Madison village | 40.3% | 21.3% |
| Cleveland, OH | Maple Heights city | 7.0% | 21.3% |
| Cleveland, OH | Mayfield Heights city | 12.7% | 21.3% |
| Cleveland, OH | Mayfield village | 34.5% | 21.3% |
| Cleveland, OH | Medina city | 27.2% | 21.3% |
| Cleveland, OH | Mentor city | 32.6% | 21.3% |
| Cleveland, OH | Mentor-on-the-Lake city | 23.0% | 21.3% |
| Cleveland, OH | Middleburg Heights city | 27.3% | 21.3% |
| Cleveland, OH | Moreland Hills village | 47.0% | 21.3% |
| Cleveland, OH | Newburgh Heights village | 10.2% | 21.3% |
| Cleveland, OH | North Madison CDP | 37.9% | 21.3% |
| Cleveland, OH | North Olmsted city | 31.2% | 21.3% |
| Cleveland, OH | North Perry village | 29.7% | 21.3% |
| Cleveland, OH | North Randall village | 11.8% | 21.3% |
| Cleveland, OH | North Ridgeville city | 28.3% | 21.3% |
| Cleveland, OH | North Royalton city | 36.2% | 21.3% |
| Cleveland, OH | Northfield village | 16.5% | 21.3% |
| Cleveland, OH | Oakwood village | 31.0% | 21.3% |
| Cleveland, OH | Olmsted Falls city | 37.4% | 21.3% |
| Cleveland, OH | Orange village | 36.7% | 21.3% |
| Cleveland, OH | Painesville city | 28.4% | 21.3% |
| Cleveland, OH | Parma Heights city | 26.8% | 21.3% |
| Cleveland, OH | Parma city | 14.5% | 21.3% |
| Cleveland, OH | Pepper Pike city | 38.3% | 21.3% |
| Cleveland, OH | Perry village | 36.6% | 21.3% |
| Cleveland, OH | Reminderville village | 38.3% | 21.3% |
| Cleveland, OH | Richfield village | 52.8% | 21.3% |
| Cleveland, OH | Richmond Heights city | 32.8% | 21.3% |
| Cleveland, OH | Rocky River city | 27.0% | 21.3% |
| Cleveland, OH | Seven Hills city | 32.4% | 21.3% |
| Cleveland, OH | Shaker Heights city | 17.1% | 21.3% |
| Cleveland, OH | Solon city | 34.6% | 21.3% |
| Cleveland, OH | South Euclid city | 22.3% | 21.3% |
| Cleveland, OH | South Russell village | 40.0% | 21.3% |
| Cleveland, OH | Streetsboro city | 32.9% | 21.3% |
| Cleveland, OH | Strongsville city | 30.1% | 21.3% |
| Cleveland, OH | Timberlake village | 34.5% | 21.3% |
| Cleveland, OH | Twinsburg Heights CDP | 17.1% | 21.3% |
| Cleveland, OH | Twinsburg city | 30.4% | 21.3% |
| Cleveland, OH | University Heights city | 15.9% | 21.3% |
| Cleveland, OH | Valley View village | 46.5% | 21.3% |
| Cleveland, OH | Walton Hills village | 27.7% | 21.3% |
| Cleveland, OH | Warrensville Heights city | 9.4% | 21.3% |
| Cleveland, OH | Westlake city | 32.8% | 21.3% |
| Cleveland, OH | Wickliffe city | 14.2% | 21.3% |
| Cleveland, OH | Willoughby Hills city | 33.0% | 21.3% |
| Cleveland, OH | Willoughby city | 15.1% | 21.3% |
| Cleveland, OH | Willowick city | 9.3% | 21.3% |
| Cleveland, OH | Woodmere village | 2.3% | 21.3% |
| Columbia, SC | Arcadia Lakes town | 50.2% | 44.2% |
| Columbia, SC | Blythewood town | 49.8% | 44.2% |
| Columbia, SC | Cayce city | 36.4% | 44.2% |
| Columbia, SC | Chapin town | 49.4% | 44.2% |
| Columbia, SC | Columbia city | 40.3% | 44.2% |
| Columbia, SC | Dentsville CDP | 41.3% | 44.2% |
| Columbia, SC | Elgin town | 34.7% | 44.2% |
| Columbia, SC | Forest Acres city | 52.0% | 44.2% |
| Columbia, SC | Gaston town | 44.0% | 44.2% |
| Columbia, SC | Irmo town | 47.1% | 44.2% |
| Columbia, SC | Lake Murray of Richland CDP | 44.6% | 44.2% |
| Columbia, SC | Lexington town | 45.6% | 44.2% |
| Columbia, SC | Lugoff CDP | 54.3% | 44.2% |
| Columbia, SC | Oak Grove CDP | 50.4% | 44.2% |
| Columbia, SC | Pine Ridge town | 53.2% | 44.2% |
| Columbia, SC | Red Bank CDP | 49.0% | 44.2% |
| Columbia, SC | Seven Oaks CDP | 53.6% | 44.2% |
| Columbia, SC | South Congaree town | 38.9% | 44.2% |
| Columbia, SC | Springdale town | 47.1% | 44.2% |
| Columbia, SC | St. Andrews CDP | 50.0% | 44.2% |
| Columbia, SC | West Columbia city | 40.3% | 44.2% |
| Columbia, SC | Woodfield CDP | 40.3% | 44.2% |
| Columbus, OH | Beechwood Trails CDP | 31.1% | 13.0% |
| Columbus, OH | Bexley city | 18.5% | 13.0% |
| Columbus, OH | Blacklick Estates CDP | 16.1% | 13.0% |
| Columbus, OH | Brice village | 24.4% | 13.0% |
| Columbus, OH | Canal Winchester city | 19.9% | 13.0% |
| Columbus, OH | Columbus city | 9.9% | 13.0% |
| Columbus, OH | Delaware city | 19.8% | 13.0% |
| Columbus, OH | Dublin city | 22.5% | 13.0% |
| Columbus, OH | Etna CDP | 11.6% | 13.0% |
| Columbus, OH | Gahanna city | 18.2% | 13.0% |
| Columbus, OH | Galena village | 44.6% | 13.0% |
| Columbus, OH | Grandview Heights city | 9.3% | 13.0% |
| Columbus, OH | Grove City city | 8.7% | 13.0% |
| Columbus, OH | Groveport city | 20.6% | 13.0% |
| Columbus, OH | Hilliard city | 6.1% | 13.0% |
| Columbus, OH | Huber Ridge CDP | 19.6% | 13.0% |
| Columbus, OH | Lake Darby CDP | 2.7% | 13.0% |
| Columbus, OH | Lincoln Village CDP | 6.4% | 13.0% |
| Columbus, OH | Lithopolis village | 34.9% | 13.0% |
| Columbus, OH | Lockbourne village | 20.8% | 13.0% |
| Columbus, OH | Marble Cliff village | 20.0% | 13.0% |
| Columbus, OH | Minerva Park village | 30.8% | 13.0% |
| Columbus, OH | New Albany city | 25.6% | 13.0% |
| Columbus, OH | Obetz village | 10.8% | 13.0% |
| Columbus, OH | Pataskala city | 16.4% | 13.0% |
| Columbus, OH | Pickerington city | 16.8% | 13.0% |
| Columbus, OH | Powell city | 24.3% | 13.0% |
| Columbus, OH | Reynoldsburg city | 11.2% | 13.0% |
| Columbus, OH | Riverlea village | 37.2% | 13.0% |
| Columbus, OH | Shawnee Hills village | 22.4% | 13.0% |
| Columbus, OH | Sunbury village | 18.7% | 13.0% |
| Columbus, OH | Upper Arlington city | 21.7% | 13.0% |
| Columbus, OH | Urbancrest village | 5.4% | 13.0% |
| Columbus, OH | Valleyview village | 11.7% | 13.0% |
| Columbus, OH | Westerville city | 23.3% | 13.0% |
| Columbus, OH | Whitehall city | 10.0% | 13.0% |
| Columbus, OH | Worthington city | 30.5% | 13.0% |
| Concord, CA | Acalanes Ridge CDP | 34.6% | 13.9% |
| Concord, CA | Alamo CDP | 37.4% | 13.9% |
| Concord, CA | Alhambra Valley CDP | 52.6% | 13.9% |
| Concord, CA | Blackhawk CDP | 16.5% | 13.9% |
| Concord, CA | Camino Tassajara CDP | 2.0% | 13.9% |
| Concord, CA | Castle Hill CDP | 43.0% | 13.9% |
| Concord, CA | Clayton city | 14.2% | 13.9% |
| Concord, CA | Clyde CDP | 6.2% | 13.9% |
| Concord, CA | Concord city | 9.7% | 13.9% |
| Concord, CA | Contra Costa Centre CDP | 17.4% | 13.9% |
| Concord, CA | Danville town | 21.8% | 13.9% |
| Concord, CA | Diablo CDP | 45.8% | 13.9% |
| Concord, CA | Dublin city | 5.3% | 13.9% |
| Concord, CA | Lafayette city | 42.3% | 13.9% |
| Concord, CA | Martinez city | 10.6% | 13.9% |
| Concord, CA | Moraga town | 34.2% | 13.9% |
| Concord, CA | Mountain View CDP | 10.5% | 13.9% |
| Concord, CA | Norris Canyon CDP | 19.5% | 13.9% |
| Concord, CA | North Gate CDP | 25.5% | 13.9% |
| Concord, CA | Orinda city | 60.2% | 13.9% |
| Concord, CA | Pacheco CDP | 2.2% | 13.9% |
| Concord, CA | Pleasant Hill city | 16.5% | 13.9% |
| Concord, CA | Pleasanton city | 10.3% | 13.9% |
| Concord, CA | Reliez Valley CDP | 33.3% | 13.9% |
| Concord, CA | San Miguel CDP | 35.5% | 13.9% |
| Concord, CA | San Ramon city | 10.8% | 13.9% |
| Concord, CA | Saranap CDP | 33.4% | 13.9% |
| Concord, CA | Shell Ridge CDP | 26.6% | 13.9% |
| Concord, CA | Vine Hill CDP | 4.2% | 13.9% |
| Concord, CA | Walnut Creek city | 21.0% | 13.9% |
| Dallas--Fort Worth--Arlington, TX | Addison town | 19.7% | 26.2% |
| Dallas--Fort Worth--Arlington, TX | Allen city | 15.1% | 26.2% |
| Dallas--Fort Worth--Arlington, TX | Arlington city | 29.3% | 26.2% |
| Dallas--Fort Worth--Arlington, TX | Azle city | 55.1% | 26.2% |
| Dallas--Fort Worth--Arlington, TX | Balch Springs city | 35.0% | 26.2% |
| Dallas--Fort Worth--Arlington, TX | Bedford city | 31.2% | 26.2% |
| Dallas--Fort Worth--Arlington, TX | Benbrook city | 30.2% | 26.2% |
| Dallas--Fort Worth--Arlington, TX | Blue Mound city | 38.2% | 26.2% |
| Dallas--Fort Worth--Arlington, TX | Briar CDP | 40.8% | 26.2% |
| Dallas--Fort Worth--Arlington, TX | Briaroaks city | 51.8% | 26.2% |
| Dallas--Fort Worth--Arlington, TX | Burleson city | 21.1% | 26.2% |
| Dallas--Fort Worth--Arlington, TX | Carrollton city | 27.2% | 26.2% |
| Dallas--Fort Worth--Arlington, TX | Cedar Hill city | 17.2% | 26.2% |
| Dallas--Fort Worth--Arlington, TX | Cockrell Hill city | 37.4% | 26.2% |
| Dallas--Fort Worth--Arlington, TX | Colleyville city | 46.7% | 26.2% |
| Dallas--Fort Worth--Arlington, TX | Coppell city | 41.1% | 26.2% |
| Dallas--Fort Worth--Arlington, TX | Cross Roads town | 42.8% | 26.2% |
| Dallas--Fort Worth--Arlington, TX | Cross Timber town | 24.5% | 26.2% |
| Dallas--Fort Worth--Arlington, TX | Crowley city | 17.7% | 26.2% |
| Dallas--Fort Worth--Arlington, TX | Dallas city | 29.4% | 26.2% |
| Dallas--Fort Worth--Arlington, TX | Dalworthington Gardens city | 39.6% | 26.2% |
| Dallas--Fort Worth--Arlington, TX | DeSoto city | 22.6% | 26.2% |
| Dallas--Fort Worth--Arlington, TX | Duncanville city | 36.4% | 26.2% |
| Dallas--Fort Worth--Arlington, TX | Edgecliff Village town | 31.7% | 26.2% |
| Dallas--Fort Worth--Arlington, TX | Euless city | 37.9% | 26.2% |
| Dallas--Fort Worth--Arlington, TX | Everman city | 27.6% | 26.2% |
| Dallas--Fort Worth--Arlington, TX | Fairview town | 27.7% | 26.2% |
| Dallas--Fort Worth--Arlington, TX | Farmers Branch city | 42.1% | 26.2% |
| Dallas--Fort Worth--Arlington, TX | Fate city | 5.7% | 26.2% |
| Dallas--Fort Worth--Arlington, TX | Ferris city | 10.0% | 26.2% |
| Dallas--Fort Worth--Arlington, TX | Flower Mound town | 0.0% | 26.2% |
| Dallas--Fort Worth--Arlington, TX | Forest Hill city | 33.6% | 26.2% |
| Dallas--Fort Worth--Arlington, TX | Fort Worth city | 27.4% | 26.2% |
| Dallas--Fort Worth--Arlington, TX | Frisco city | 16.1% | 26.2% |
| Dallas--Fort Worth--Arlington, TX | Garland city | 24.7% | 26.2% |
| Dallas--Fort Worth--Arlington, TX | Glenn Heights city | 15.8% | 26.2% |
| Dallas--Fort Worth--Arlington, TX | Grand Prairie city | 25.1% | 26.2% |
| Dallas--Fort Worth--Arlington, TX | Grapevine city | 46.2% | 26.2% |
| Dallas--Fort Worth--Arlington, TX | Hackberry town | 30.9% | 26.2% |
| Dallas--Fort Worth--Arlington, TX | Haltom City city | 32.9% | 26.2% |
| Dallas--Fort Worth--Arlington, TX | Haslet city | 28.5% | 26.2% |
| Dallas--Fort Worth--Arlington, TX | Heath city | 25.3% | 26.2% |
| Dallas--Fort Worth--Arlington, TX | Hebron town | 30.8% | 26.2% |
| Dallas--Fort Worth--Arlington, TX | Highland Park town | 50.7% | 26.2% |
| Dallas--Fort Worth--Arlington, TX | Hurst city | 37.2% | 26.2% |
| Dallas--Fort Worth--Arlington, TX | Hutchins city | 36.5% | 26.2% |
| Dallas--Fort Worth--Arlington, TX | Irving city | 29.1% | 26.2% |
| Dallas--Fort Worth--Arlington, TX | Joshua city | 26.6% | 26.2% |
| Dallas--Fort Worth--Arlington, TX | Keller city | 37.2% | 26.2% |
| Dallas--Fort Worth--Arlington, TX | Kennedale city | 28.9% | 26.2% |
| Dallas--Fort Worth--Arlington, TX | Lake Worth city | 38.7% | 26.2% |
| Dallas--Fort Worth--Arlington, TX | Lakeside town | 51.0% | 26.2% |
| Dallas--Fort Worth--Arlington, TX | Lakewood Village city | 36.0% | 26.2% |
| Dallas--Fort Worth--Arlington, TX | Lancaster city | 15.5% | 26.2% |
| Dallas--Fort Worth--Arlington, TX | Lavon city | 5.0% | 26.2% |
| Dallas--Fort Worth--Arlington, TX | Lewisville city | 18.6% | 26.2% |
| Dallas--Fort Worth--Arlington, TX | Lincoln Park town | 25.4% | 26.2% |
| Dallas--Fort Worth--Arlington, TX | Little Elm city | 18.9% | 26.2% |
| Dallas--Fort Worth--Arlington, TX | Lucas city | 26.4% | 26.2% |
| Dallas--Fort Worth--Arlington, TX | Mansfield city | 23.0% | 26.2% |
| Dallas--Fort Worth--Arlington, TX | McKinney city | 2.0% | 26.2% |
| Dallas--Fort Worth--Arlington, TX | McLendon-Chisholm city | 7.6% | 26.2% |
| Dallas--Fort Worth--Arlington, TX | Mesquite city | 20.0% | 26.2% |
| Dallas--Fort Worth--Arlington, TX | Midlothian city | 14.7% | 26.2% |
| Dallas--Fort Worth--Arlington, TX | Mobile City city | 6.2% | 26.2% |
| Dallas--Fort Worth--Arlington, TX | Murphy city | 10.7% | 26.2% |
| Dallas--Fort Worth--Arlington, TX | North Richland Hills city | 32.7% | 26.2% |
| Dallas--Fort Worth--Arlington, TX | Northlake town | 18.9% | 26.2% |
| Dallas--Fort Worth--Arlington, TX | Oak Leaf city | 25.2% | 26.2% |
| Dallas--Fort Worth--Arlington, TX | Oak Point city | 36.6% | 26.2% |
| Dallas--Fort Worth--Arlington, TX | Ovilla city | 25.9% | 26.2% |
| Dallas--Fort Worth--Arlington, TX | Pantego town | 27.7% | 26.2% |
| Dallas--Fort Worth--Arlington, TX | Parker city | 24.1% | 26.2% |
| Dallas--Fort Worth--Arlington, TX | Pecan Acres CDP | 4.1% | 26.2% |
| Dallas--Fort Worth--Arlington, TX | Pecan Hill city | 22.9% | 26.2% |
| Dallas--Fort Worth--Arlington, TX | Pelican Bay city | 47.3% | 26.2% |
| Dallas--Fort Worth--Arlington, TX | Plano city | 22.0% | 26.2% |
| Dallas--Fort Worth--Arlington, TX | Prosper town | 16.4% | 26.2% |
| Dallas--Fort Worth--Arlington, TX | Providence Village town | 13.0% | 26.2% |
| Dallas--Fort Worth--Arlington, TX | Red Oak city | 12.8% | 26.2% |
| Dallas--Fort Worth--Arlington, TX | Rendon CDP | 34.0% | 26.2% |
| Dallas--Fort Worth--Arlington, TX | Reno city | 27.8% | 26.2% |
| Dallas--Fort Worth--Arlington, TX | Richardson city | 25.4% | 26.2% |
| Dallas--Fort Worth--Arlington, TX | Richland Hills city | 40.5% | 26.2% |
| Dallas--Fort Worth--Arlington, TX | River Oaks city | 54.4% | 26.2% |
| Dallas--Fort Worth--Arlington, TX | Roanoke city | 27.0% | 26.2% |
| Dallas--Fort Worth--Arlington, TX | Rockwall city | 14.4% | 26.2% |
| Dallas--Fort Worth--Arlington, TX | Rowlett city | 15.3% | 26.2% |
| Dallas--Fort Worth--Arlington, TX | Royse City city | 4.4% | 26.2% |
| Dallas--Fort Worth--Arlington, TX | Sachse city | 12.2% | 26.2% |
| Dallas--Fort Worth--Arlington, TX | Saginaw city | 21.2% | 26.2% |
| Dallas--Fort Worth--Arlington, TX | Sanctuary town | 40.3% | 26.2% |
| Dallas--Fort Worth--Arlington, TX | Sansom Park city | 46.0% | 26.2% |
| Dallas--Fort Worth--Arlington, TX | Savannah CDP | 7.9% | 26.2% |
| Dallas--Fort Worth--Arlington, TX | Seagoville city | 24.8% | 26.2% |
| Dallas--Fort Worth--Arlington, TX | Southlake city | 44.9% | 26.2% |
| Dallas--Fort Worth--Arlington, TX | St. Paul town | 9.0% | 26.2% |
| Dallas--Fort Worth--Arlington, TX | Sunnyvale town | 25.7% | 26.2% |
| Dallas--Fort Worth--Arlington, TX | The Colony city | 28.2% | 26.2% |
| Dallas--Fort Worth--Arlington, TX | Travis Ranch CDP | 3.7% | 26.2% |
| Dallas--Fort Worth--Arlington, TX | Trophy Club town | 34.9% | 26.2% |
| Dallas--Fort Worth--Arlington, TX | University Park city | 42.9% | 26.2% |
| Dallas--Fort Worth--Arlington, TX | Watauga city | 27.4% | 26.2% |
| Dallas--Fort Worth--Arlington, TX | Waxahachie city | 19.6% | 26.2% |
| Dallas--Fort Worth--Arlington, TX | Westlake town | 53.8% | 26.2% |
| Dallas--Fort Worth--Arlington, TX | Westover Hills town | 67.6% | 26.2% |
| Dallas--Fort Worth--Arlington, TX | Westworth Village city | 42.1% | 26.2% |
| Dallas--Fort Worth--Arlington, TX | White Settlement city | 26.6% | 26.2% |
| Dallas--Fort Worth--Arlington, TX | Wilmer city | 23.2% | 26.2% |
| Dallas--Fort Worth--Arlington, TX | Wylie city | 9.3% | 26.2% |
| Dayton, OH | Beavercreek city | 36.5% | 24.5% |
| Dayton, OH | Bellbrook city | 36.4% | 24.5% |
| Dayton, OH | Brookville city | 11.4% | 24.5% |
| Dayton, OH | Centerville city | 32.1% | 24.5% |
| Dayton, OH | Clayton city | 33.8% | 24.5% |
| Dayton, OH | Corwin village | 29.3% | 24.5% |
| Dayton, OH | Crystal Lakes CDP | 39.3% | 24.5% |
| Dayton, OH | Dayton city | 15.8% | 24.5% |
| Dayton, OH | Drexel CDP | 30.9% | 24.5% |
| Dayton, OH | Englewood city | 26.3% | 24.5% |
| Dayton, OH | Enon village | 40.2% | 24.5% |
| Dayton, OH | Fairborn city | 23.3% | 24.5% |
| Dayton, OH | Five Points CDP | 37.2% | 24.5% |
| Dayton, OH | Green Meadows CDP | 22.6% | 24.5% |
| Dayton, OH | Holiday Valley CDP | 23.6% | 24.5% |
| Dayton, OH | Huber Heights city | 19.5% | 24.5% |
| Dayton, OH | Kettering city | 23.4% | 24.5% |
| Dayton, OH | Miamisburg city | 22.3% | 24.5% |
| Dayton, OH | Moraine city | 18.1% | 24.5% |
| Dayton, OH | New Carlisle city | 17.6% | 24.5% |
| Dayton, OH | Oakwood city | 22.2% | 24.5% |
| Dayton, OH | Park Layne CDP | 18.1% | 24.5% |
| Dayton, OH | Phillipsburg village | 15.6% | 24.5% |
| Dayton, OH | Riverside city | 25.9% | 24.5% |
| Dayton, OH | Springboro city | 24.9% | 24.5% |
| Dayton, OH | Tipp City city | 37.7% | 24.5% |
| Dayton, OH | Trotwood city | 23.6% | 24.5% |
| Dayton, OH | Troy city | 40.6% | 24.5% |
| Dayton, OH | Union city | 33.6% | 24.5% |
| Dayton, OH | Vandalia city | 25.2% | 24.5% |
| Dayton, OH | Waynesville village | 23.3% | 24.5% |
| Dayton, OH | West Carrollton city | 20.5% | 24.5% |
| Dayton, OH | West Milton village | 71.6% | 24.5% |
| Dayton, OH | Wright-Patterson AFB CDP | 22.0% | 24.5% |
| Dayton, OH | Xenia city | 23.4% | 24.5% |
| Denver--Aurora, CO | Acres Green CDP | 28.7% | 21.8% |
| Denver--Aurora, CO | Applewood CDP | 41.7% | 21.8% |
| Denver--Aurora, CO | Arvada city | 28.7% | 21.8% |
| Denver--Aurora, CO | Aurora city | 18.5% | 21.8% |
| Denver--Aurora, CO | Berkley CDP | 21.3% | 21.8% |
| Denver--Aurora, CO | Bow Mar town | 46.0% | 21.8% |
| Denver--Aurora, CO | Brighton city | 11.0% | 21.8% |
| Denver--Aurora, CO | Broomfield city | 24.7% | 21.8% |
| Denver--Aurora, CO | Castle Pines Village CDP | 48.7% | 21.8% |
| Denver--Aurora, CO | Castle Pines city | 31.9% | 21.8% |
| Denver--Aurora, CO | Castle Rock town | 17.2% | 21.8% |
| Denver--Aurora, CO | Centennial city | 30.6% | 21.8% |
| Denver--Aurora, CO | Cherry Creek CDP | 30.0% | 21.8% |
| Denver--Aurora, CO | Cherry Hills Village city | 49.0% | 21.8% |
| Denver--Aurora, CO | Columbine CDP | 33.2% | 21.8% |
| Denver--Aurora, CO | Columbine Valley town | 50.1% | 21.8% |
| Denver--Aurora, CO | Commerce City city | 12.7% | 21.8% |
| Denver--Aurora, CO | Dakota Ridge CDP | 26.1% | 21.8% |
| Denver--Aurora, CO | Denver city | 22.9% | 21.8% |
| Denver--Aurora, CO | Derby CDP | 13.4% | 21.8% |
| Denver--Aurora, CO | Dove Valley CDP | 11.4% | 21.8% |
| Denver--Aurora, CO | East Pleasant View CDP | 18.4% | 21.8% |
| Denver--Aurora, CO | Edgewater city | 29.3% | 21.8% |
| Denver--Aurora, CO | Englewood city | 23.5% | 21.8% |
| Denver--Aurora, CO | Fairmount CDP | 18.4% | 21.8% |
| Denver--Aurora, CO | Federal Heights city | 15.9% | 21.8% |
| Denver--Aurora, CO | Foxfield town | 13.8% | 21.8% |
| Denver--Aurora, CO | Glendale city | 16.6% | 21.8% |
| Denver--Aurora, CO | Golden city | 15.8% | 21.8% |
| Denver--Aurora, CO | Grand View Estates CDP | 8.5% | 21.8% |
| Denver--Aurora, CO | Greenwood Village city | 36.9% | 21.8% |
| Denver--Aurora, CO | Highlands Ranch CDP | 21.6% | 21.8% |
| Denver--Aurora, CO | Holly Hills CDP | 41.0% | 21.8% |
| Denver--Aurora, CO | Inverness CDP | 8.0% | 21.8% |
| Denver--Aurora, CO | Ken Caryl CDP | 22.1% | 21.8% |
| Denver--Aurora, CO | Lakeside town | 8.0% | 21.8% |
| Denver--Aurora, CO | Lakewood city | 28.2% | 21.8% |
| Denver--Aurora, CO | Littleton city | 35.7% | 21.8% |
| Denver--Aurora, CO | Lochbuie town | 4.0% | 21.8% |
| Denver--Aurora, CO | Lone Tree city | 19.5% | 21.8% |
| Denver--Aurora, CO | Meridian CDP | 15.9% | 21.8% |
| Denver--Aurora, CO | Morrison town | 34.6% | 21.8% |
| Denver--Aurora, CO | Mountain View town | 27.8% | 21.8% |
| Denver--Aurora, CO | North Washington CDP | 9.1% | 21.8% |
| Denver--Aurora, CO | Northglenn city | 20.8% | 21.8% |
| Denver--Aurora, CO | Parker town | 14.7% | 21.8% |
| Denver--Aurora, CO | Shaw Heights CDP | 27.7% | 21.8% |
| Denver--Aurora, CO | Sheridan city | 19.0% | 21.8% |
| Denver--Aurora, CO | Sherrelwood CDP | 20.6% | 21.8% |
| Denver--Aurora, CO | Stonegate CDP | 22.5% | 21.8% |
| Denver--Aurora, CO | The Pinery CDP | 20.5% | 21.8% |
| Denver--Aurora, CO | Thornton city | 18.4% | 21.8% |
| Denver--Aurora, CO | Todd Creek CDP | 8.5% | 21.8% |
| Denver--Aurora, CO | Twin Lakes CDP | 18.7% | 21.8% |
| Denver--Aurora, CO | Welby CDP | 15.7% | 21.8% |
| Denver--Aurora, CO | West Pleasant View CDP | 13.1% | 21.8% |
| Denver--Aurora, CO | Westminster city | 25.1% | 21.8% |
| Denver--Aurora, CO | Wheat Ridge city | 30.8% | 21.8% |
| Des Moines, IA | Altoona city | 17.4% | 29.9% |
| Des Moines, IA | Ankeny city | 30.4% | 29.9% |
| Des Moines, IA | Bondurant city | 13.9% | 29.9% |
| Des Moines, IA | Clive city | 29.3% | 29.9% |
| Des Moines, IA | Des Moines city | 33.5% | 29.9% |
| Des Moines, IA | Grimes city | 15.8% | 29.9% |
| Des Moines, IA | Johnston city | 18.8% | 29.9% |
| Des Moines, IA | Norwalk city | 24.3% | 29.9% |
| Des Moines, IA | Pleasant Hill city | 40.5% | 29.9% |
| Des Moines, IA | Saylorville CDP | 51.0% | 29.9% |
| Des Moines, IA | Urbandale city | 27.8% | 29.9% |
| Des Moines, IA | Waukee city | 14.5% | 29.9% |
| Des Moines, IA | West Des Moines city | 28.4% | 29.9% |
| Des Moines, IA | Windsor Heights city | 32.5% | 29.9% |
| Detroit, MI | Algonac city | 46.4% | 29.9% |
| Detroit, MI | Allen Park city | 18.3% | 29.9% |
| Detroit, MI | Auburn Hills city | 39.6% | 29.9% |
| Detroit, MI | Berkley city | 39.1% | 29.9% |
| Detroit, MI | Beverly Hills village | 52.2% | 29.9% |
| Detroit, MI | Bingham Farms village | 61.3% | 29.9% |
| Detroit, MI | Birmingham city | 36.8% | 29.9% |
| Detroit, MI | Bloomfield Hills city | 56.2% | 29.9% |
| Detroit, MI | Carleton village | 40.9% | 29.9% |
| Detroit, MI | Center Line city | 18.5% | 29.9% |
| Detroit, MI | Clawson city | 30.1% | 29.9% |
| Detroit, MI | Dearborn Heights city | 24.0% | 29.9% |
| Detroit, MI | Dearborn city | 14.0% | 29.9% |
| Detroit, MI | Detroit city | 27.4% | 29.9% |
| Detroit, MI | Eastpointe city | 17.3% | 29.9% |
| Detroit, MI | Ecorse city | 10.6% | 29.9% |
| Detroit, MI | Farmington Hills city | 41.6% | 29.9% |
| Detroit, MI | Farmington city | 36.6% | 29.9% |
| Detroit, MI | Ferndale city | 34.7% | 29.9% |
| Detroit, MI | Flat Rock city | 21.6% | 29.9% |
| Detroit, MI | Franklin village | 67.5% | 29.9% |
| Detroit, MI | Fraser city | 18.6% | 29.9% |
| Detroit, MI | Garden City city | 21.9% | 29.9% |
| Detroit, MI | Gibraltar city | 23.2% | 29.9% |
| Detroit, MI | Grosse Pointe Farms city | 55.0% | 29.9% |
| Detroit, MI | Grosse Pointe Park city | 46.4% | 29.9% |
| Detroit, MI | Grosse Pointe Woods city | 41.8% | 29.9% |
| Detroit, MI | Grosse Pointe city | 50.8% | 29.9% |
| Detroit, MI | Hamtramck city | 5.3% | 29.9% |
| Detroit, MI | Harper Woods city | 34.5% | 29.9% |
| Detroit, MI | Hazel Park city | 29.1% | 29.9% |
| Detroit, MI | Highland Park city | 19.5% | 29.9% |
| Detroit, MI | Huntington Woods city | 53.4% | 29.9% |
| Detroit, MI | Inkster city | 30.6% | 29.9% |
| Detroit, MI | Keego Harbor city | 24.5% | 29.9% |
| Detroit, MI | Lake Angelus city | 72.8% | 29.9% |
| Detroit, MI | Lake Orion village | 51.6% | 29.9% |
| Detroit, MI | Lathrup Village city | 47.3% | 29.9% |
| Detroit, MI | Lincoln Park city | 17.1% | 29.9% |
| Detroit, MI | Livonia city | 34.2% | 29.9% |
| Detroit, MI | Madison Heights city | 20.4% | 29.9% |
| Detroit, MI | Melvindale city | 12.8% | 29.9% |
| Detroit, MI | Milford village | 47.5% | 29.9% |
| Detroit, MI | Mount Clemens city | 31.0% | 29.9% |
| Detroit, MI | New Baltimore city | 46.6% | 29.9% |
| Detroit, MI | New Haven village | 38.9% | 29.9% |
| Detroit, MI | Northville city | 43.2% | 29.9% |
| Detroit, MI | Novi city | 36.1% | 29.9% |
| Detroit, MI | Oak Park city | 35.8% | 29.9% |
| Detroit, MI | Orchard Lake Village city | 57.3% | 29.9% |
| Detroit, MI | Oxford village | 55.2% | 29.9% |
| Detroit, MI | Pearl Beach CDP | 27.8% | 29.9% |
| Detroit, MI | Pleasant Ridge city | 54.9% | 29.9% |
| Detroit, MI | Plymouth city | 37.8% | 29.9% |
| Detroit, MI | Pontiac city | 31.5% | 29.9% |
| Detroit, MI | River Rouge city | 8.4% | 29.9% |
| Detroit, MI | Riverview city | 16.9% | 29.9% |
| Detroit, MI | Rochester Hills city | 48.6% | 29.9% |
| Detroit, MI | Rochester city | 47.5% | 29.9% |
| Detroit, MI | Rockwood city | 24.5% | 29.9% |
| Detroit, MI | Romeo village | 57.9% | 29.9% |
| Detroit, MI | Romulus city | 30.3% | 29.9% |
| Detroit, MI | Roseville city | 15.6% | 29.9% |
| Detroit, MI | Royal Oak city | 39.5% | 29.9% |
| Detroit, MI | South Rockwood village | 29.3% | 29.9% |
| Detroit, MI | Southfield city | 31.4% | 29.9% |
| Detroit, MI | Southgate city | 14.6% | 29.9% |
| Detroit, MI | St. Clair Shores city | 21.2% | 29.9% |
| Detroit, MI | Sterling Heights city | 21.1% | 29.9% |
| Detroit, MI | Sylvan Lake city | 47.7% | 29.9% |
| Detroit, MI | Taylor city | 22.3% | 29.9% |
| Detroit, MI | Trenton city | 18.3% | 29.9% |
| Detroit, MI | Troy city | 36.5% | 29.9% |
| Detroit, MI | Utica city | 33.4% | 29.9% |
| Detroit, MI | Village of Clarkston city | 57.3% | 29.9% |
| Detroit, MI | Village of Grosse Pointe Shores city | 51.4% | 29.9% |
| Detroit, MI | Walled Lake city | 35.8% | 29.9% |
| Detroit, MI | Warren city | 22.2% | 29.9% |
| Detroit, MI | Wayne city | 28.1% | 29.9% |
| Detroit, MI | Westland city | 25.2% | 29.9% |
| Detroit, MI | Wixom city | 45.9% | 29.9% |
| Detroit, MI | Wolverine Lake village | 40.2% | 29.9% |
| Detroit, MI | Woodhaven city | 18.0% | 29.9% |
| Detroit, MI | Wyandotte city | 13.2% | 29.9% |
| El Paso, TX--NM | Agua Dulce CDP | 3.0% | 5.4% |
| El Paso, TX--NM | Anthony city | 4.1% | 5.4% |
| El Paso, TX--NM | Anthony town | 3.1% | 5.4% |
| El Paso, TX--NM | Berino CDP | 1.8% | 5.4% |
| El Paso, TX--NM | Canutillo CDP | 6.1% | 5.4% |
| El Paso, TX--NM | Clint town | 11.1% | 5.4% |
| El Paso, TX--NM | El Paso city | 5.8% | 5.4% |
| El Paso, TX--NM | Fort Bliss CDP | 2.3% | 5.4% |
| El Paso, TX--NM | Homestead Meadows North CDP | 0.6% | 5.4% |
| El Paso, TX--NM | Homestead Meadows South CDP | 2.8% | 5.4% |
| El Paso, TX--NM | Horizon City city | 1.9% | 5.4% |
| El Paso, TX--NM | Morning Glory CDP | 16.9% | 5.4% |
| El Paso, TX--NM | Prado Verde CDP | 13.4% | 5.4% |
| El Paso, TX--NM | San Elizario city | 10.1% | 5.4% |
| El Paso, TX--NM | Santa Teresa CDP | 4.1% | 5.4% |
| El Paso, TX--NM | Socorro city | 8.4% | 5.4% |
| El Paso, TX--NM | Sparks CDP | 0.8% | 5.4% |
| El Paso, TX--NM | Sunland Park city | 1.7% | 5.4% |
| El Paso, TX--NM | Vinton village | 2.5% | 5.4% |
| El Paso, TX--NM | Westway CDP | 2.0% | 5.4% |
| Fayetteville, NC | Fayetteville city | 47.6% | 48.5% |
| Fayetteville, NC | Hope Mills town | 42.7% | 48.5% |
| Fayetteville, NC | Parkton town | 64.0% | 48.5% |
| Fayetteville, NC | Raeford city | 66.7% | 48.5% |
| Fayetteville, NC | Rockfish CDP | 62.6% | 48.5% |
| Fayetteville, NC | Silver City CDP | 71.6% | 48.5% |
| Fayetteville, NC | Spring Lake town | 30.6% | 48.5% |
| Fayetteville, NC | Vander CDP | 49.5% | 48.5% |
| Flint, MI | Argentine CDP | 81.3% | 53.7% |
| Flint, MI | Beecher CDP | 55.9% | 53.7% |
| Flint, MI | Burton city | 50.7% | 53.7% |
| Flint, MI | Clio city | 54.1% | 53.7% |
| Flint, MI | Davison city | 38.3% | 53.7% |
| Flint, MI | Fenton city | 55.1% | 53.7% |
| Flint, MI | Flint city | 47.4% | 53.7% |
| Flint, MI | Flushing city | 64.1% | 53.7% |
| Flint, MI | Grand Blanc city | 53.8% | 53.7% |
| Flint, MI | Lake Fenton CDP | 63.4% | 53.7% |
| Flint, MI | Linden city | 56.2% | 53.7% |
| Flint, MI | Mount Morris city | 54.7% | 53.7% |
| Flint, MI | Swartz Creek city | 51.4% | 53.7% |
| Grand Rapids, MI | Allendale CDP | 41.8% | 49.8% |
| Grand Rapids, MI | Byron Center CDP | 45.6% | 49.8% |
| Grand Rapids, MI | Caledonia village | 44.6% | 49.8% |
| Grand Rapids, MI | Comstock Park CDP | 51.8% | 49.8% |
| Grand Rapids, MI | Cutlerville CDP | 42.0% | 49.8% |
| Grand Rapids, MI | East Grand Rapids city | 72.3% | 49.8% |
| Grand Rapids, MI | Forest Hills CDP | 71.4% | 49.8% |
| Grand Rapids, MI | Grand Rapids city | 48.6% | 49.8% |
| Grand Rapids, MI | Grandville city | 36.9% | 49.8% |
| Grand Rapids, MI | Hudsonville city | 36.1% | 49.8% |
| Grand Rapids, MI | Jenison CDP | 44.6% | 49.8% |
| Grand Rapids, MI | Kentwood city | 54.2% | 49.8% |
| Grand Rapids, MI | Northview CDP | 66.0% | 49.8% |
| Grand Rapids, MI | Rockford city | 56.6% | 49.8% |
| Grand Rapids, MI | Walker city | 54.8% | 49.8% |
| Grand Rapids, MI | Wyoming city | 38.1% | 49.8% |
| Greenville, SC | Arial CDP | 39.0% | 37.1% |
| Greenville, SC | Berea CDP | 41.5% | 37.1% |
| Greenville, SC | Central town | 22.2% | 37.1% |
| Greenville, SC | City View CDP | 33.7% | 37.1% |
| Greenville, SC | Clemson city | 39.8% | 37.1% |
| Greenville, SC | Dunean CDP | 33.6% | 37.1% |
| Greenville, SC | Easley city | 38.3% | 37.1% |
| Greenville, SC | Gantt CDP | 35.8% | 37.1% |
| Greenville, SC | Golden Grove CDP | 45.7% | 37.1% |
| Greenville, SC | Greenville city | 31.7% | 37.1% |
| Greenville, SC | Greer city | 22.8% | 37.1% |
| Greenville, SC | Judson CDP | 38.1% | 37.1% |
| Greenville, SC | Liberty city | 36.5% | 37.1% |
| Greenville, SC | Lyman town | 30.2% | 37.1% |
| Greenville, SC | Mauldin city | 26.9% | 37.1% |
| Greenville, SC | Norris town | 33.7% | 37.1% |
| Greenville, SC | Parker CDP | 35.0% | 37.1% |
| Greenville, SC | Pelzer town | 22.2% | 37.1% |
| Greenville, SC | Pendleton town | 45.8% | 37.1% |
| Greenville, SC | Pickens city | 23.7% | 37.1% |
| Greenville, SC | Piedmont CDP | 40.1% | 37.1% |
| Greenville, SC | Powdersville CDP | 40.8% | 37.1% |
| Greenville, SC | Sans Souci CDP | 36.2% | 37.1% |
| Greenville, SC | Slater-Marietta CDP | 38.2% | 37.1% |
| Greenville, SC | Taylors CDP | 39.2% | 37.1% |
| Greenville, SC | Travelers Rest city | 36.6% | 37.1% |
| Greenville, SC | Wade Hampton CDP | 39.9% | 37.1% |
| Greenville, SC | Welcome CDP | 41.9% | 37.1% |
| Greenville, SC | West Pelzer town | 38.2% | 37.1% |
| Greenville, SC | Williamston town | 38.0% | 37.1% |
| Harrisburg, PA | Boiling Springs CDP | 64.5% | 36.3% |
| Harrisburg, PA | Bressler CDP | 37.6% | 36.3% |
| Harrisburg, PA | Camp Hill borough | 31.3% | 36.3% |
| Harrisburg, PA | Campbelltown CDP | 34.4% | 36.3% |
| Harrisburg, PA | Carlisle borough | 44.9% | 36.3% |
| Harrisburg, PA | Colonial Park CDP | 37.8% | 36.3% |
| Harrisburg, PA | Dauphin borough | 24.1% | 36.3% |
| Harrisburg, PA | Dillsburg borough | 19.0% | 36.3% |
| Harrisburg, PA | Enhaut CDP | 42.3% | 36.3% |
| Harrisburg, PA | Enola CDP | 28.8% | 36.3% |
| Harrisburg, PA | Franklintown borough | 9.6% | 36.3% |
| Harrisburg, PA | Goldsboro borough | 33.2% | 36.3% |
| Harrisburg, PA | Harrisburg city | 30.6% | 36.3% |
| Harrisburg, PA | Hershey CDP | 35.9% | 36.3% |
| Harrisburg, PA | Highspire borough | 16.8% | 36.3% |
| Harrisburg, PA | Hummelstown borough | 20.9% | 36.3% |
| Harrisburg, PA | Lawnton CDP | 23.6% | 36.3% |
| Harrisburg, PA | Lemoyne borough | 24.8% | 36.3% |
| Harrisburg, PA | Lewisberry borough | 36.7% | 36.3% |
| Harrisburg, PA | Linglestown CDP | 45.2% | 36.3% |
| Harrisburg, PA | Lower Allen CDP | 28.9% | 36.3% |
| Harrisburg, PA | Marysville borough | 16.4% | 36.3% |
| Harrisburg, PA | Mechanicsburg borough | 27.0% | 36.3% |
| Harrisburg, PA | Messiah College CDP | 35.4% | 36.3% |
| Harrisburg, PA | Middletown borough | 28.4% | 36.3% |
| Harrisburg, PA | New Cumberland borough | 33.1% | 36.3% |
| Harrisburg, PA | New Kingstown CDP | 41.3% | 36.3% |
| Harrisburg, PA | New Market CDP | 26.1% | 36.3% |
| Harrisburg, PA | Oberlin CDP | 35.8% | 36.3% |
| Harrisburg, PA | Palmdale CDP | 16.6% | 36.3% |
| Harrisburg, PA | Palmyra borough | 20.5% | 36.3% |
| Harrisburg, PA | Paxtang borough | 39.1% | 36.3% |
| Harrisburg, PA | Paxtonia CDP | 37.3% | 36.3% |
| Harrisburg, PA | Penbrook borough | 27.2% | 36.3% |
| Harrisburg, PA | Progress CDP | 48.1% | 36.3% |
| Harrisburg, PA | Royalton borough | 29.1% | 36.3% |
| Harrisburg, PA | Rutherford CDP | 25.2% | 36.3% |
| Harrisburg, PA | Schlusser CDP | 60.0% | 36.3% |
| Harrisburg, PA | Shiremanstown borough | 30.9% | 36.3% |
| Harrisburg, PA | Skyline View CDP | 43.8% | 36.3% |
| Harrisburg, PA | Steelton borough | 29.2% | 36.3% |
| Harrisburg, PA | Union Deposit CDP | 41.2% | 36.3% |
| Harrisburg, PA | Valley Green CDP | 36.6% | 36.3% |
| Harrisburg, PA | West Fairview CDP | 22.3% | 36.3% |
| Harrisburg, PA | Wormleysburg borough | 27.0% | 36.3% |
| Harrisburg, PA | York Haven borough | 28.2% | 36.3% |
| Hartford, CT | Blue Hills CDP | 35.2% | 39.1% |
| Hartford, CT | Bristol city | 38.7% | 39.1% |
| Hartford, CT | Canton Valley CDP | 58.8% | 39.1% |
| Hartford, CT | Collinsville CDP | 60.7% | 39.1% |
| Hartford, CT | Crystal Lake CDP | 74.9% | 39.1% |
| Hartford, CT | East Hartford CDP | 33.8% | 39.1% |
| Hartford, CT | Glastonbury Center CDP | 56.8% | 39.1% |
| Hartford, CT | Hartford city | 13.2% | 39.1% |
| Hartford, CT | Higganum CDP | 58.2% | 39.1% |
| Hartford, CT | Kensington CDP | 48.6% | 39.1% |
| Hartford, CT | Manchester CDP | 26.7% | 39.1% |
| Hartford, CT | Meriden city | 51.1% | 39.1% |
| Hartford, CT | Middletown city | 44.7% | 39.1% |
| Hartford, CT | New Britain city | 21.0% | 39.1% |
| Hartford, CT | New Hartford Center CDP | 57.7% | 39.1% |
| Hartford, CT | Newington CDP | 38.3% | 39.1% |
| Hartford, CT | North Granby CDP | 71.2% | 39.1% |
| Hartford, CT | Plantsville CDP | 50.0% | 39.1% |
| Hartford, CT | Portland CDP | 50.1% | 39.1% |
| Hartford, CT | Rockville CDP | 39.5% | 39.1% |
| Hartford, CT | Salmon Brook CDP | 53.2% | 39.1% |
| Hartford, CT | Simsbury Center CDP | 58.1% | 39.1% |
| Hartford, CT | Storrs CDP | 23.3% | 39.1% |
| Hartford, CT | Tariffville CDP | 52.4% | 39.1% |
| Hartford, CT | Terramuggus CDP | 65.7% | 39.1% |
| Hartford, CT | Terryville CDP | 46.3% | 39.1% |
| Hartford, CT | Thomaston CDP | 49.7% | 39.1% |
| Hartford, CT | Weatogue CDP | 70.2% | 39.1% |
| Hartford, CT | West Hartford CDP | 30.0% | 39.1% |
| Hartford, CT | West Simsbury CDP | 63.3% | 39.1% |
| Hartford, CT | Wethersfield CDP | 35.4% | 39.1% |
| Hickory, NC | Bethlehem CDP | 53.9% | 53.5% |
| Hickory, NC | Brookford town | 53.9% | 53.5% |
| Hickory, NC | Cajah's Mountain town | 45.7% | 53.5% |
| Hickory, NC | Claremont city | 43.1% | 53.5% |
| Hickory, NC | Connelly Springs town | 57.3% | 53.5% |
| Hickory, NC | Conover city | 60.0% | 53.5% |
| Hickory, NC | Drexel town | 49.9% | 53.5% |
| Hickory, NC | Gamewell town | 39.9% | 53.5% |
| Hickory, NC | Glen Alpine town | 49.0% | 53.5% |
| Hickory, NC | Granite Falls town | 44.3% | 53.5% |
| Hickory, NC | Hickory city | 52.4% | 53.5% |
| Hickory, NC | Hildebran town | 48.8% | 53.5% |
| Hickory, NC | Hudson town | 46.1% | 53.5% |
| Hickory, NC | Icard CDP | 51.3% | 53.5% |
| Hickory, NC | Lenoir city | 46.3% | 53.5% |
| Hickory, NC | Long View town | 48.6% | 53.5% |
| Hickory, NC | Maiden town | 22.0% | 53.5% |
| Hickory, NC | Morganton city | 49.2% | 53.5% |
| Hickory, NC | Mountain View CDP | 57.1% | 53.5% |
| Hickory, NC | Newton city | 57.6% | 53.5% |
| Hickory, NC | Northlakes CDP | 71.4% | 53.5% |
| Hickory, NC | Rhodhiss town | 65.6% | 53.5% |
| Hickory, NC | Rutherford College town | 50.5% | 53.5% |
| Hickory, NC | Salem CDP | 61.8% | 53.5% |
| Hickory, NC | Sawmills town | 48.0% | 53.5% |
| Hickory, NC | St. Stephens CDP | 63.4% | 53.5% |
| Hickory, NC | Valdese town | 55.1% | 53.5% |
| Houston, TX | Aldine CDP | 33.9% | 28.1% |
| Houston, TX | Alvin city | 33.5% | 28.1% |
| Houston, TX | Arcola city | 27.5% | 28.1% |
| Houston, TX | Atascocita CDP | 28.2% | 28.1% |
| Houston, TX | Bacliff CDP | 35.4% | 28.1% |
| Houston, TX | Barrett CDP | 33.7% | 28.1% |
| Houston, TX | Baytown city | 29.1% | 28.1% |
| Houston, TX | Bellaire city | 37.8% | 28.1% |
| Houston, TX | Brookside Village city | 38.0% | 28.1% |
| Houston, TX | Bunker Hill Village city | 70.4% | 28.1% |
| Houston, TX | Channelview CDP | 22.9% | 28.1% |
| Houston, TX | Cinco Ranch CDP | 39.2% | 28.1% |
| Houston, TX | Clear Lake Shores city | 17.3% | 28.1% |
| Houston, TX | Cloverleaf CDP | 23.3% | 28.1% |
| Houston, TX | Cove city | 24.4% | 28.1% |
| Houston, TX | Crosby CDP | 32.1% | 28.1% |
| Houston, TX | Cumings CDP | 8.2% | 28.1% |
| Houston, TX | Deer Park city | 14.6% | 28.1% |
| Houston, TX | El Lago city | 34.5% | 28.1% |
| Houston, TX | Fifth Street CDP | 37.9% | 28.1% |
| Houston, TX | Four Corners CDP | 16.7% | 28.1% |
| Houston, TX | Fresno CDP | 20.8% | 28.1% |
| Houston, TX | Friendswood city | 36.9% | 28.1% |
| Houston, TX | Galena Park city | 32.0% | 28.1% |
| Houston, TX | Hedwig Village city | 61.5% | 28.1% |
| Houston, TX | Highlands CDP | 34.9% | 28.1% |
| Houston, TX | Hillcrest village | 45.4% | 28.1% |
| Houston, TX | Hilshire Village city | 62.4% | 28.1% |
| Houston, TX | Houston city | 25.7% | 28.1% |
| Houston, TX | Humble city | 31.1% | 28.1% |
| Houston, TX | Hunters Creek Village city | 62.7% | 28.1% |
| Houston, TX | Iowa Colony village | 40.9% | 28.1% |
| Houston, TX | Jacinto City city | 31.6% | 28.1% |
| Houston, TX | Jersey Village city | 46.5% | 28.1% |
| Houston, TX | Katy city | 31.6% | 28.1% |
| Houston, TX | Kemah city | 25.5% | 28.1% |
| Houston, TX | La Porte city | 18.7% | 28.1% |
| Houston, TX | League City city | 13.7% | 28.1% |
| Houston, TX | Manvel city | 9.3% | 28.1% |
| Houston, TX | Meadows Place city | 34.9% | 28.1% |
| Houston, TX | Mission Bend CDP | 34.5% | 28.1% |
| Houston, TX | Missouri City city | 31.7% | 28.1% |
| Houston, TX | Mont Belvieu city | 5.3% | 28.1% |
| Houston, TX | Morgan's Point city | 30.4% | 28.1% |
| Houston, TX | Nassau Bay city | 14.9% | 28.1% |
| Houston, TX | Pasadena city | 17.3% | 28.1% |
| Houston, TX | Patton Village city | 69.0% | 28.1% |
| Houston, TX | Pearland city | 19.8% | 28.1% |
| Houston, TX | Pecan Grove CDP | 40.9% | 28.1% |
| Houston, TX | Pinehurst CDP | 69.4% | 28.1% |
| Houston, TX | Piney Point Village city | 63.1% | 28.1% |
| Houston, TX | Pleak village | 12.4% | 28.1% |
| Houston, TX | Porter Heights CDP | 55.0% | 28.1% |
| Houston, TX | Richmond city | 38.2% | 28.1% |
| Houston, TX | Roman Forest city | 65.0% | 28.1% |
| Houston, TX | Rosenberg city | 23.3% | 28.1% |
| Houston, TX | San Leon CDP | 14.6% | 28.1% |
| Houston, TX | Seabrook city | 23.4% | 28.1% |
| Houston, TX | Sheldon CDP | 22.0% | 28.1% |
| Houston, TX | Shoreacres city | 34.1% | 28.1% |
| Houston, TX | Sienna Plantation CDP | 28.8% | 28.1% |
| Houston, TX | South Houston city | 24.4% | 28.1% |
| Houston, TX | Southside Place city | 44.6% | 28.1% |
| Houston, TX | Splendora city | 51.3% | 28.1% |
| Houston, TX | Spring CDP | 39.7% | 28.1% |
| Houston, TX | Spring Valley Village city | 58.9% | 28.1% |
| Houston, TX | Stafford city | 22.6% | 28.1% |
| Houston, TX | Stagecoach town | 71.4% | 28.1% |
| Houston, TX | Sugar Land city | 33.0% | 28.1% |
| Houston, TX | Taylor Lake Village city | 37.3% | 28.1% |
| Houston, TX | Texas City city | 8.6% | 28.1% |
| Houston, TX | The Woodlands CDP | 30.9% | 28.1% |
| Houston, TX | Tomball city | 48.0% | 28.1% |
| Houston, TX | Webster city | 10.8% | 28.1% |
| Houston, TX | West University Place city | 46.1% | 28.1% |
| Houston, TX | Woodbranch city | 64.3% | 28.1% |
| Huntsville, AL | Harvest CDP | 56.6% | 51.8% |
| Huntsville, AL | Hazel Green CDP | 83.7% | 51.8% |
| Huntsville, AL | Huntsville city | 51.7% | 51.8% |
| Huntsville, AL | Madison city | 43.3% | 51.8% |
| Huntsville, AL | Meridianville CDP | 61.7% | 51.8% |
| Huntsville, AL | Moores Mill CDP | 67.8% | 51.8% |
| Huntsville, AL | Redstone Arsenal CDP | 39.1% | 51.8% |
| Huntsville, AL | Triana town | 57.9% | 51.8% |
| Indianapolis, IN | Arcadia town | 27.0% | 24.0% |
| Indianapolis, IN | Avon town | 22.6% | 24.0% |
| Indianapolis, IN | Bargersville town | 20.7% | 24.0% |
| Indianapolis, IN | Beech Grove city | 16.5% | 24.0% |
| Indianapolis, IN | Bethany town | 62.5% | 24.0% |
| Indianapolis, IN | Brooklyn town | 26.3% | 24.0% |
| Indianapolis, IN | Brownsburg town | 20.9% | 24.0% |
| Indianapolis, IN | Carmel city | 30.4% | 24.0% |
| Indianapolis, IN | Cicero town | 27.1% | 24.0% |
| Indianapolis, IN | Clermont town | 32.7% | 24.0% |
| Indianapolis, IN | Crows Nest town | 74.1% | 24.0% |
| Indianapolis, IN | Cumberland town | 24.3% | 24.0% |
| Indianapolis, IN | Danville town | 19.4% | 24.0% |
| Indianapolis, IN | Fishers city | 37.8% | 24.0% |
| Indianapolis, IN | Fortville town | 22.3% | 24.0% |
| Indianapolis, IN | Franklin city | 17.3% | 24.0% |
| Indianapolis, IN | Greenfield city | 13.3% | 24.0% |
| Indianapolis, IN | Greenwood city | 14.3% | 24.0% |
| Indianapolis, IN | Homecroft town | 30.3% | 24.0% |
| Indianapolis, IN | Indianapolis city (balance) | 23.0% | 24.0% |
| Indianapolis, IN | Ingalls town | 3.5% | 24.0% |
| Indianapolis, IN | Lawrence city | 22.6% | 24.0% |
| Indianapolis, IN | McCordsville town | 13.7% | 24.0% |
| Indianapolis, IN | Meridian Hills town | 46.4% | 24.0% |
| Indianapolis, IN | Mooresville town | 20.9% | 24.0% |
| Indianapolis, IN | New Palestine town | 22.0% | 24.0% |
| Indianapolis, IN | New Whiteland town | 20.8% | 24.0% |
| Indianapolis, IN | Noblesville city | 32.3% | 24.0% |
| Indianapolis, IN | North Crows Nest town | 59.0% | 24.0% |
| Indianapolis, IN | Pittsboro town | 20.2% | 24.0% |
| Indianapolis, IN | Plainfield town | 20.0% | 24.0% |
| Indianapolis, IN | Rocky Ripple town | 58.2% | 24.0% |
| Indianapolis, IN | Southport city | 25.1% | 24.0% |
| Indianapolis, IN | Speedway town | 18.2% | 24.0% |
| Indianapolis, IN | Spring Hill town | 62.8% | 24.0% |
| Indianapolis, IN | Spring Lake town | 37.2% | 24.0% |
| Indianapolis, IN | Warren Park town | 14.5% | 24.0% |
| Indianapolis, IN | Westfield city | 20.0% | 24.0% |
| Indianapolis, IN | Whiteland town | 16.5% | 24.0% |
| Indianapolis, IN | Whitestown town | 6.6% | 24.0% |
| Indianapolis, IN | Williams Creek town | 51.7% | 24.0% |
| Indianapolis, IN | Wynnedale town | 63.8% | 24.0% |
| Indianapolis, IN | Zionsville town | 27.9% | 24.0% |
| Jackson, MS | Brandon city | 57.9% | 56.3% |
| Jackson, MS | Byram city | 50.1% | 56.3% |
| Jackson, MS | Cleary CDP | 78.5% | 56.3% |
| Jackson, MS | Clinton city | 60.4% | 56.3% |
| Jackson, MS | Florence city | 70.9% | 56.3% |
| Jackson, MS | Flowood city | 36.0% | 56.3% |
| Jackson, MS | Jackson city | 61.0% | 56.3% |
| Jackson, MS | Madison city | 50.1% | 56.3% |
| Jackson, MS | Pearl city | 45.3% | 56.3% |
| Jackson, MS | Richland city | 49.4% | 56.3% |
| Jackson, MS | Ridgeland city | 44.0% | 56.3% |
| Jacksonville, FL | Asbury Lake CDP | 28.0% | 44.0% |
| Jacksonville, FL | Atlantic Beach city | 29.3% | 44.0% |
| Jacksonville, FL | Bellair-Meadowbrook Terrace CDP | 22.7% | 44.0% |
| Jacksonville, FL | Fleming Island CDP | 46.3% | 44.0% |
| Jacksonville, FL | Fruit Cove CDP | 65.4% | 44.0% |
| Jacksonville, FL | Green Cove Springs city | 51.3% | 44.0% |
| Jacksonville, FL | Jacksonville Beach city | 24.3% | 44.0% |
| Jacksonville, FL | Jacksonville city | 43.2% | 44.0% |
| Jacksonville, FL | Lakeside CDP | 42.6% | 44.0% |
| Jacksonville, FL | Middleburg CDP | 71.4% | 44.0% |
| Jacksonville, FL | Neptune Beach city | 33.6% | 44.0% |
| Jacksonville, FL | Nocatee CDP | 44.6% | 44.0% |
| Jacksonville, FL | Oakleaf Plantation CDP | 47.0% | 44.0% |
| Jacksonville, FL | Orange Park town | 33.8% | 44.0% |
| Jacksonville, FL | Palm Valley CDP | 45.5% | 44.0% |
| Jacksonville, FL | Sawgrass CDP | 46.5% | 44.0% |
| Kansas City, MO--KS | Avondale city | 36.7% | 36.6% |
| Kansas City, MO--KS | Belton city | 24.5% | 36.6% |
| Kansas City, MO--KS | Birmingham village | 17.3% | 36.6% |
| Kansas City, MO--KS | Blue Springs city | 30.1% | 36.6% |
| Kansas City, MO--KS | Bonner Springs city | 45.8% | 36.6% |
| Kansas City, MO--KS | Claycomo village | 29.4% | 36.6% |
| Kansas City, MO--KS | Edwardsville city | 46.3% | 36.6% |
| Kansas City, MO--KS | Fairway city | 69.3% | 36.6% |
| Kansas City, MO--KS | Gardner city | 11.5% | 36.6% |
| Kansas City, MO--KS | Gladstone city | 31.2% | 36.6% |
| Kansas City, MO--KS | Glenaire city | 42.2% | 36.6% |
| Kansas City, MO--KS | Grain Valley city | 10.7% | 36.6% |
| Kansas City, MO--KS | Grandview city | 35.2% | 36.6% |
| Kansas City, MO--KS | Houston Lake city | 50.0% | 36.6% |
| Kansas City, MO--KS | Independence city | 37.4% | 36.6% |
| Kansas City, MO--KS | Kansas City city | 35.3% | 36.6% |
| Kansas City, MO--KS | Lake Quivira city | 81.1% | 36.6% |
| Kansas City, MO--KS | Lake Tapawingo city | 27.0% | 36.6% |
| Kansas City, MO--KS | Lake Waukomis city | 51.4% | 36.6% |
| Kansas City, MO--KS | Leawood city | 50.3% | 36.6% |
| Kansas City, MO--KS | Lee's Summit city | 32.4% | 36.6% |
| Kansas City, MO--KS | Lenexa city | 42.7% | 36.6% |
| Kansas City, MO--KS | Liberty city | 35.1% | 36.6% |
| Kansas City, MO--KS | Merriam city | 56.2% | 36.6% |
| Kansas City, MO--KS | Mission Hills city | 66.7% | 36.6% |
| Kansas City, MO--KS | Mission Woods city | 55.1% | 36.6% |
| Kansas City, MO--KS | Mission city | 59.3% | 36.6% |
| Kansas City, MO--KS | North Kansas City city | 27.1% | 36.6% |
| Kansas City, MO--KS | Northmoor city | 56.3% | 36.6% |
| Kansas City, MO--KS | Oak Grove city | 23.4% | 36.6% |
| Kansas City, MO--KS | Oaks village | 47.4% | 36.6% |
| Kansas City, MO--KS | Oakview village | 45.2% | 36.6% |
| Kansas City, MO--KS | Oakwood Park village | 46.3% | 36.6% |
| Kansas City, MO--KS | Oakwood village | 53.7% | 36.6% |
| Kansas City, MO--KS | Olathe city | 32.1% | 36.6% |
| Kansas City, MO--KS | Overland Park city | 41.2% | 36.6% |
| Kansas City, MO--KS | Parkville city | 51.3% | 36.6% |
| Kansas City, MO--KS | Peculiar city | 24.7% | 36.6% |
| Kansas City, MO--KS | Platte Woods city | 46.0% | 36.6% |
| Kansas City, MO--KS | Pleasant Valley city | 29.9% | 36.6% |
| Kansas City, MO--KS | Prairie Village city | 68.1% | 36.6% |
| Kansas City, MO--KS | Randolph village | 31.6% | 36.6% |
| Kansas City, MO--KS | Raymore city | 17.5% | 36.6% |
| Kansas City, MO--KS | Raytown city | 40.8% | 36.6% |
| Kansas City, MO--KS | Riverside city | 50.1% | 36.6% |
| Kansas City, MO--KS | Roeland Park city | 60.8% | 36.6% |
| Kansas City, MO--KS | Shawnee city | 42.7% | 36.6% |
| Kansas City, MO--KS | Smithville city | 27.3% | 36.6% |
| Kansas City, MO--KS | Sugar Creek city | 34.7% | 36.6% |
| Kansas City, MO--KS | Weatherby Lake city | 46.8% | 36.6% |
| Kansas City, MO--KS | Westwood Hills city | 52.9% | 36.6% |
| Kansas City, MO--KS | Westwood city | 54.4% | 36.6% |
| Knoxville, TN | Alcoa city | 61.9% | 63.4% |
| Knoxville, TN | Clinton city | 52.2% | 63.4% |
| Knoxville, TN | Coalfield CDP | 84.3% | 63.4% |
| Knoxville, TN | Eagleton Village CDP | 71.3% | 63.4% |
| Knoxville, TN | Farragut town | 58.9% | 63.4% |
| Knoxville, TN | Friendsville city | 51.1% | 63.4% |
| Knoxville, TN | Knoxville city | 65.5% | 63.4% |
| Knoxville, TN | Lenoir City city | 45.6% | 63.4% |
| Knoxville, TN | Loudon town | 49.8% | 63.4% |
| Knoxville, TN | Louisville city | 70.7% | 63.4% |
| Knoxville, TN | Maryville city | 64.8% | 63.4% |
| Knoxville, TN | Mascot CDP | 61.1% | 63.4% |
| Knoxville, TN | Oak Ridge city | 64.7% | 63.4% |
| Knoxville, TN | Oliver Springs town | 48.9% | 63.4% |
| Knoxville, TN | Rockford city | 76.2% | 63.4% |
| Knoxville, TN | Sevierville city | 76.0% | 63.4% |
| Knoxville, TN | Seymour CDP | 57.9% | 63.4% |
| Knoxville, TN | Tellico Village CDP | 59.9% | 63.4% |
| Lancaster, PA | Adamstown borough | 31.8% | 36.9% |
| Lancaster, PA | Akron borough | 47.7% | 36.9% |
| Lancaster, PA | Bainbridge CDP | 53.2% | 36.9% |
| Lancaster, PA | Bird-in-Hand CDP | 33.0% | 36.9% |
| Lancaster, PA | Blue Ball CDP | 38.8% | 36.9% |
| Lancaster, PA | Bowmansville CDP | 37.9% | 36.9% |
| Lancaster, PA | Brickerville CDP | 54.4% | 36.9% |
| Lancaster, PA | Brownstown CDP | 32.8% | 36.9% |
| Lancaster, PA | Clay CDP | 26.8% | 36.9% |
| Lancaster, PA | Columbia borough | 16.1% | 36.9% |
| Lancaster, PA | Conestoga CDP | 64.2% | 36.9% |
| Lancaster, PA | Denver borough | 30.5% | 36.9% |
| Lancaster, PA | East Earl CDP | 53.2% | 36.9% |
| Lancaster, PA | East Petersburg borough | 44.7% | 36.9% |
| Lancaster, PA | Elizabethtown borough | 36.9% | 36.9% |
| Lancaster, PA | Ephrata borough | 33.7% | 36.9% |
| Lancaster, PA | Fivepointville CDP | 53.7% | 36.9% |
| Lancaster, PA | Gordonville CDP | 34.9% | 36.9% |
| Lancaster, PA | Hopeland CDP | 33.7% | 36.9% |
| Lancaster, PA | Intercourse CDP | 43.7% | 36.9% |
| Lancaster, PA | Lampeter CDP | 30.6% | 36.9% |
| Lancaster, PA | Lancaster city | 15.3% | 36.9% |
| Lancaster, PA | Landisville CDP | 49.7% | 36.9% |
| Lancaster, PA | Leola CDP | 31.5% | 36.9% |
| Lancaster, PA | Lititz borough | 27.8% | 36.9% |
| Lancaster, PA | Manheim borough | 38.7% | 36.9% |
| Lancaster, PA | Marietta borough | 38.1% | 36.9% |
| Lancaster, PA | Maytown CDP | 45.2% | 36.9% |
| Lancaster, PA | Millersville borough | 38.6% | 36.9% |
| Lancaster, PA | Mount Joy borough | 37.6% | 36.9% |
| Lancaster, PA | Mountville borough | 26.5% | 36.9% |
| Lancaster, PA | New Holland borough | 23.3% | 36.9% |
| Lancaster, PA | Paradise CDP | 32.0% | 36.9% |
| Lancaster, PA | Penryn CDP | 47.1% | 36.9% |
| Lancaster, PA | Reamstown CDP | 46.9% | 36.9% |
| Lancaster, PA | Reinholds CDP | 38.0% | 36.9% |
| Lancaster, PA | Rheems CDP | 42.0% | 36.9% |
| Lancaster, PA | Ronks CDP | 27.1% | 36.9% |
| Lancaster, PA | Rothsville CDP | 45.1% | 36.9% |
| Lancaster, PA | Salunga CDP | 45.7% | 36.9% |
| Lancaster, PA | Schoeneck CDP | 40.0% | 36.9% |
| Lancaster, PA | Smoketown CDP | 36.3% | 36.9% |
| Lancaster, PA | Soudersburg CDP | 39.8% | 36.9% |
| Lancaster, PA | Stevens CDP | 43.9% | 36.9% |
| Lancaster, PA | Strasburg borough | 32.8% | 36.9% |
| Lancaster, PA | Swartzville CDP | 33.8% | 36.9% |
| Lancaster, PA | Terre Hill borough | 45.4% | 36.9% |
| Lancaster, PA | Washington Boro CDP | 48.7% | 36.9% |
| Lancaster, PA | Willow Street CDP | 34.2% | 36.9% |
| Lancaster, PA | Witmer CDP | 21.4% | 36.9% |
| Lancaster, PA | Wrightsville borough | 27.4% | 36.9% |
| Las Vegas--Henderson, NV | Enterprise CDP | 3.9% | 6.0% |
| Las Vegas--Henderson, NV | Henderson city | 8.0% | 6.0% |
| Las Vegas--Henderson, NV | Las Vegas city | 7.0% | 6.0% |
| Las Vegas--Henderson, NV | Nellis AFB CDP | 2.5% | 6.0% |
| Las Vegas--Henderson, NV | North Las Vegas city | 3.2% | 6.0% |
| Las Vegas--Henderson, NV | Paradise CDP | 7.6% | 6.0% |
| Las Vegas--Henderson, NV | Spring Valley CDP | 6.4% | 6.0% |
| Las Vegas--Henderson, NV | Summerlin South CDP | 13.0% | 6.0% |
| Las Vegas--Henderson, NV | Sunrise Manor CDP | 4.3% | 6.0% |
| Las Vegas--Henderson, NV | Whitney CDP | 4.2% | 6.0% |
| Las Vegas--Henderson, NV | Winchester CDP | 8.8% | 6.0% |
| Little Rock, AR | Alexander city | 81.5% | 67.4% |
| Little Rock, AR | Austin city | 78.0% | 67.4% |
| Little Rock, AR | Bauxite town | 87.0% | 67.4% |
| Little Rock, AR | Benton city | 70.2% | 67.4% |
| Little Rock, AR | Bryant city | 76.9% | 67.4% |
| Little Rock, AR | Cabot city | 71.1% | 67.4% |
| Little Rock, AR | Cammack Village city | 74.5% | 67.4% |
| Little Rock, AR | College Station CDP | 70.2% | 67.4% |
| Little Rock, AR | Gibson CDP | 87.2% | 67.4% |
| Little Rock, AR | Haskell city | 80.8% | 67.4% |
| Little Rock, AR | Jacksonville city | 69.4% | 67.4% |
| Little Rock, AR | Little Rock city | 66.3% | 67.4% |
| Little Rock, AR | Maumelle city | 54.3% | 67.4% |
| Little Rock, AR | McAlmont CDP | 79.1% | 67.4% |
| Little Rock, AR | North Little Rock city | 65.0% | 67.4% |
| Little Rock, AR | Salem CDP | 85.9% | 67.4% |
| Little Rock, AR | Shannon Hills city | 68.0% | 67.4% |
| Little Rock, AR | Sherwood city | 65.7% | 67.4% |
| Little Rock, AR | Sweet Home CDP | 85.9% | 67.4% |
| Little Rock, AR | Ward city | 83.3% | 67.4% |
| Little Rock, AR | Wrightsville city | 44.8% | 67.4% |
| Los Angeles--Long Beach--Anaheim, CA | Alhambra city | 9.2% | 17.2% |
| Los Angeles--Long Beach--Anaheim, CA | Alondra Park CDP | 12.8% | 17.2% |
| Los Angeles--Long Beach--Anaheim, CA | Altadena CDP | 32.6% | 17.2% |
| Los Angeles--Long Beach--Anaheim, CA | Anaheim city | 9.6% | 17.2% |
| Los Angeles--Long Beach--Anaheim, CA | Arcadia city | 23.5% | 17.2% |
| Los Angeles--Long Beach--Anaheim, CA | Artesia city | 7.0% | 17.2% |
| Los Angeles--Long Beach--Anaheim, CA | Avocado Heights CDP | 14.6% | 17.2% |
| Los Angeles--Long Beach--Anaheim, CA | Azusa city | 11.4% | 17.2% |
| Los Angeles--Long Beach--Anaheim, CA | Baldwin Park city | 8.5% | 17.2% |
| Los Angeles--Long Beach--Anaheim, CA | Bell Canyon CDP | 47.9% | 17.2% |
| Los Angeles--Long Beach--Anaheim, CA | Bell Gardens city | 15.6% | 17.2% |
| Los Angeles--Long Beach--Anaheim, CA | Bell city | 14.9% | 17.2% |
| Los Angeles--Long Beach--Anaheim, CA | Bellflower city | 9.0% | 17.2% |
| Los Angeles--Long Beach--Anaheim, CA | Beverly Hills city | 25.5% | 17.2% |
| Los Angeles--Long Beach--Anaheim, CA | Bradbury city | 45.3% | 17.2% |
| Los Angeles--Long Beach--Anaheim, CA | Brea city | 14.7% | 17.2% |
| Los Angeles--Long Beach--Anaheim, CA | Buena Park city | 8.3% | 17.2% |
| Los Angeles--Long Beach--Anaheim, CA | Burbank city | 26.3% | 17.2% |
| Los Angeles--Long Beach--Anaheim, CA | Calabasas city | 37.8% | 17.2% |
| Los Angeles--Long Beach--Anaheim, CA | Carson city | 24.6% | 17.2% |
| Los Angeles--Long Beach--Anaheim, CA | Cerritos city | 16.0% | 17.2% |
| Los Angeles--Long Beach--Anaheim, CA | Charter Oak CDP | 8.0% | 17.2% |
| Los Angeles--Long Beach--Anaheim, CA | Chino Hills city | 15.0% | 17.2% |
| Los Angeles--Long Beach--Anaheim, CA | Chino city | 15.4% | 17.2% |
| Los Angeles--Long Beach--Anaheim, CA | Citrus CDP | 12.1% | 17.2% |
| Los Angeles--Long Beach--Anaheim, CA | Claremont city | 22.3% | 17.2% |
| Los Angeles--Long Beach--Anaheim, CA | Commerce city | 9.2% | 17.2% |
| Los Angeles--Long Beach--Anaheim, CA | Compton city | 26.8% | 17.2% |
| Los Angeles--Long Beach--Anaheim, CA | Costa Mesa city | 19.1% | 17.2% |
| Los Angeles--Long Beach--Anaheim, CA | Covina city | 9.6% | 17.2% |
| Los Angeles--Long Beach--Anaheim, CA | Cudahy city | 14.5% | 17.2% |
| Los Angeles--Long Beach--Anaheim, CA | Culver City city | 22.8% | 17.2% |
| Los Angeles--Long Beach--Anaheim, CA | Cypress city | 9.9% | 17.2% |
| Los Angeles--Long Beach--Anaheim, CA | Del Aire CDP | 21.8% | 17.2% |
| Los Angeles--Long Beach--Anaheim, CA | Diamond Bar city | 11.9% | 17.2% |
| Los Angeles--Long Beach--Anaheim, CA | Downey city | 11.2% | 17.2% |
| Los Angeles--Long Beach--Anaheim, CA | Duarte city | 14.8% | 17.2% |
| Los Angeles--Long Beach--Anaheim, CA | East Los Angeles CDP | 10.8% | 17.2% |
| Los Angeles--Long Beach--Anaheim, CA | East Pasadena CDP | 28.2% | 17.2% |
| Los Angeles--Long Beach--Anaheim, CA | East Rancho Dominguez CDP | 19.8% | 17.2% |
| Los Angeles--Long Beach--Anaheim, CA | East San Gabriel CDP | 18.4% | 17.2% |
| Los Angeles--Long Beach--Anaheim, CA | East Whittier CDP | 7.6% | 17.2% |
| Los Angeles--Long Beach--Anaheim, CA | Eastvale city | 4.3% | 17.2% |
| Los Angeles--Long Beach--Anaheim, CA | El Monte city | 11.3% | 17.2% |
| Los Angeles--Long Beach--Anaheim, CA | El Segundo city | 17.2% | 17.2% |
| Los Angeles--Long Beach--Anaheim, CA | Florence-Graham CDP | 26.9% | 17.2% |
| Los Angeles--Long Beach--Anaheim, CA | Fontana city | 34.2% | 17.2% |
| Los Angeles--Long Beach--Anaheim, CA | Fountain Valley city | 11.0% | 17.2% |
| Los Angeles--Long Beach--Anaheim, CA | Fullerton city | 14.9% | 17.2% |
| Los Angeles--Long Beach--Anaheim, CA | Garden Grove city | 9.0% | 17.2% |
| Los Angeles--Long Beach--Anaheim, CA | Gardena city | 15.2% | 17.2% |
| Los Angeles--Long Beach--Anaheim, CA | Glendale city | 30.4% | 17.2% |
| Los Angeles--Long Beach--Anaheim, CA | Glendora city | 15.5% | 17.2% |
| Los Angeles--Long Beach--Anaheim, CA | Hacienda Heights CDP | 13.0% | 17.2% |
| Los Angeles--Long Beach--Anaheim, CA | Hawaiian Gardens city | 6.8% | 17.2% |
| Los Angeles--Long Beach--Anaheim, CA | Hawthorne city | 12.1% | 17.2% |
| Los Angeles--Long Beach--Anaheim, CA | Hermosa Beach city | 11.7% | 17.2% |
| Los Angeles--Long Beach--Anaheim, CA | Hidden Hills city | 45.6% | 17.2% |
| Los Angeles--Long Beach--Anaheim, CA | Huntington Beach city | 9.2% | 17.2% |
| Los Angeles--Long Beach--Anaheim, CA | Huntington Park city | 21.9% | 17.2% |
| Los Angeles--Long Beach--Anaheim, CA | Industry city | 5.6% | 17.2% |
| Los Angeles--Long Beach--Anaheim, CA | Inglewood city | 18.5% | 17.2% |
| Los Angeles--Long Beach--Anaheim, CA | Irvine city | 14.4% | 17.2% |
| Los Angeles--Long Beach--Anaheim, CA | Irwindale city | 4.5% | 17.2% |
| Los Angeles--Long Beach--Anaheim, CA | La CaÃ±ada Flintridge city | 55.9% | 17.2% |
| Los Angeles--Long Beach--Anaheim, CA | La Crescenta-Montrose CDP | 50.7% | 17.2% |
| Los Angeles--Long Beach--Anaheim, CA | La Habra Heights city | 37.9% | 17.2% |
| Los Angeles--Long Beach--Anaheim, CA | La Habra city | 8.3% | 17.2% |
| Los Angeles--Long Beach--Anaheim, CA | La Mirada city | 11.1% | 17.2% |
| Los Angeles--Long Beach--Anaheim, CA | La Palma city | 9.7% | 17.2% |
| Los Angeles--Long Beach--Anaheim, CA | La Puente city | 7.9% | 17.2% |
| Los Angeles--Long Beach--Anaheim, CA | La Verne city | 13.2% | 17.2% |
| Los Angeles--Long Beach--Anaheim, CA | Ladera Heights CDP | 14.0% | 17.2% |
| Los Angeles--Long Beach--Anaheim, CA | Lake Forest city | 4.1% | 17.2% |
| Los Angeles--Long Beach--Anaheim, CA | Lakewood city | 12.5% | 17.2% |
| Los Angeles--Long Beach--Anaheim, CA | Lawndale city | 14.5% | 17.2% |
| Los Angeles--Long Beach--Anaheim, CA | Lennox CDP | 15.3% | 17.2% |
| Los Angeles--Long Beach--Anaheim, CA | Lomita city | 20.0% | 17.2% |
| Los Angeles--Long Beach--Anaheim, CA | Long Beach city | 15.0% | 17.2% |
| Los Angeles--Long Beach--Anaheim, CA | Los Alamitos city | 9.8% | 17.2% |
| Los Angeles--Long Beach--Anaheim, CA | Los Angeles city | 22.1% | 17.2% |
| Los Angeles--Long Beach--Anaheim, CA | Lynwood city | 24.8% | 17.2% |
| Los Angeles--Long Beach--Anaheim, CA | Malibu city | 31.6% | 17.2% |
| Los Angeles--Long Beach--Anaheim, CA | Manhattan Beach city | 21.7% | 17.2% |
| Los Angeles--Long Beach--Anaheim, CA | Marina del Rey CDP | 17.1% | 17.2% |
| Los Angeles--Long Beach--Anaheim, CA | Mayflower Village CDP | 13.0% | 17.2% |
| Los Angeles--Long Beach--Anaheim, CA | Maywood city | 14.3% | 17.2% |
| Los Angeles--Long Beach--Anaheim, CA | Midway City CDP | 5.5% | 17.2% |
| Los Angeles--Long Beach--Anaheim, CA | Monrovia city | 19.7% | 17.2% |
| Los Angeles--Long Beach--Anaheim, CA | Montclair city | 12.6% | 17.2% |
| Los Angeles--Long Beach--Anaheim, CA | Montebello city | 7.6% | 17.2% |
| Los Angeles--Long Beach--Anaheim, CA | Monterey Park city | 8.6% | 17.2% |
| Los Angeles--Long Beach--Anaheim, CA | Newport Beach city | 20.8% | 17.2% |
| Los Angeles--Long Beach--Anaheim, CA | North El Monte CDP | 16.1% | 17.2% |
| Los Angeles--Long Beach--Anaheim, CA | North Tustin CDP | 13.8% | 17.2% |
| Los Angeles--Long Beach--Anaheim, CA | Norwalk city | 7.2% | 17.2% |
| Los Angeles--Long Beach--Anaheim, CA | Ontario city | 17.4% | 17.2% |
| Los Angeles--Long Beach--Anaheim, CA | Orange city | 8.6% | 17.2% |
| Los Angeles--Long Beach--Anaheim, CA | Palos Verdes Estates city | 40.8% | 17.2% |
| Los Angeles--Long Beach--Anaheim, CA | Paramount city | 12.9% | 17.2% |
| Los Angeles--Long Beach--Anaheim, CA | Pasadena city | 25.8% | 17.2% |
| Los Angeles--Long Beach--Anaheim, CA | Pico Rivera city | 9.4% | 17.2% |
| Los Angeles--Long Beach--Anaheim, CA | Placentia city | 10.5% | 17.2% |
| Los Angeles--Long Beach--Anaheim, CA | Pomona city | 8.3% | 17.2% |
| Los Angeles--Long Beach--Anaheim, CA | Rancho Cucamonga city | 24.8% | 17.2% |
| Los Angeles--Long Beach--Anaheim, CA | Rancho Palos Verdes city | 36.2% | 17.2% |
| Los Angeles--Long Beach--Anaheim, CA | Redondo Beach city | 18.9% | 17.2% |
| Los Angeles--Long Beach--Anaheim, CA | Rolling Hills Estates city | 46.0% | 17.2% |
| Los Angeles--Long Beach--Anaheim, CA | Rolling Hills city | 57.6% | 17.2% |
| Los Angeles--Long Beach--Anaheim, CA | Rose Hills CDP | 34.6% | 17.2% |
| Los Angeles--Long Beach--Anaheim, CA | Rosemead city | 10.0% | 17.2% |
| Los Angeles--Long Beach--Anaheim, CA | Rossmoor CDP | 14.1% | 17.2% |
| Los Angeles--Long Beach--Anaheim, CA | Rowland Heights CDP | 12.3% | 17.2% |
| Los Angeles--Long Beach--Anaheim, CA | San Antonio Heights CDP | 42.2% | 17.2% |
| Los Angeles--Long Beach--Anaheim, CA | San Dimas city | 15.6% | 17.2% |
| Los Angeles--Long Beach--Anaheim, CA | San Fernando city | 17.7% | 17.2% |
| Los Angeles--Long Beach--Anaheim, CA | San Gabriel city | 10.7% | 17.2% |
| Los Angeles--Long Beach--Anaheim, CA | San Marino city | 40.7% | 17.2% |
| Los Angeles--Long Beach--Anaheim, CA | San Pasqual CDP | 29.5% | 17.2% |
| Los Angeles--Long Beach--Anaheim, CA | Santa Ana city | 12.8% | 17.2% |
| Los Angeles--Long Beach--Anaheim, CA | Santa Fe Springs city | 8.9% | 17.2% |
| Los Angeles--Long Beach--Anaheim, CA | Santa Monica city | 23.2% | 17.2% |
| Los Angeles--Long Beach--Anaheim, CA | Seal Beach city | 8.6% | 17.2% |
| Los Angeles--Long Beach--Anaheim, CA | Sierra Madre city | 45.0% | 17.2% |
| Los Angeles--Long Beach--Anaheim, CA | Signal Hill city | 14.0% | 17.2% |
| Los Angeles--Long Beach--Anaheim, CA | South El Monte city | 11.7% | 17.2% |
| Los Angeles--Long Beach--Anaheim, CA | South Gate city | 23.0% | 17.2% |
| Los Angeles--Long Beach--Anaheim, CA | South Monrovia Island CDP | 16.6% | 17.2% |
| Los Angeles--Long Beach--Anaheim, CA | South Pasadena city | 31.3% | 17.2% |
| Los Angeles--Long Beach--Anaheim, CA | South San Gabriel CDP | 17.4% | 17.2% |
| Los Angeles--Long Beach--Anaheim, CA | South San Jose Hills CDP | 10.4% | 17.2% |
| Los Angeles--Long Beach--Anaheim, CA | South Whittier CDP | 11.1% | 17.2% |
| Los Angeles--Long Beach--Anaheim, CA | Stanton city | 7.2% | 17.2% |
| Los Angeles--Long Beach--Anaheim, CA | Temple City city | 18.6% | 17.2% |
| Los Angeles--Long Beach--Anaheim, CA | Topanga CDP | 62.8% | 17.2% |
| Los Angeles--Long Beach--Anaheim, CA | Torrance city | 20.6% | 17.2% |
| Los Angeles--Long Beach--Anaheim, CA | Tustin city | 9.7% | 17.2% |
| Los Angeles--Long Beach--Anaheim, CA | Upland city | 27.8% | 17.2% |
| Los Angeles--Long Beach--Anaheim, CA | Valinda CDP | 10.8% | 17.2% |
| Los Angeles--Long Beach--Anaheim, CA | Vernon city | 9.7% | 17.2% |
| Los Angeles--Long Beach--Anaheim, CA | View Park-Windsor Hills CDP | 25.0% | 17.2% |
| Los Angeles--Long Beach--Anaheim, CA | Villa Park city | 17.2% | 17.2% |
| Los Angeles--Long Beach--Anaheim, CA | Vincent CDP | 8.6% | 17.2% |
| Los Angeles--Long Beach--Anaheim, CA | Walnut Park CDP | 26.9% | 17.2% |
| Los Angeles--Long Beach--Anaheim, CA | Walnut city | 15.1% | 17.2% |
| Los Angeles--Long Beach--Anaheim, CA | West Athens CDP | 16.8% | 17.2% |
| Los Angeles--Long Beach--Anaheim, CA | West Carson CDP | 19.7% | 17.2% |
| Los Angeles--Long Beach--Anaheim, CA | West Covina city | 12.5% | 17.2% |
| Los Angeles--Long Beach--Anaheim, CA | West Hollywood city | 26.6% | 17.2% |
| Los Angeles--Long Beach--Anaheim, CA | West Puente Valley CDP | 7.3% | 17.2% |
| Los Angeles--Long Beach--Anaheim, CA | West Rancho Dominguez CDP | 25.5% | 17.2% |
| Los Angeles--Long Beach--Anaheim, CA | West Whittier-Los Nietos CDP | 12.2% | 17.2% |
| Los Angeles--Long Beach--Anaheim, CA | Westminster city | 6.1% | 17.2% |
| Los Angeles--Long Beach--Anaheim, CA | Westmont CDP | 18.1% | 17.2% |
| Los Angeles--Long Beach--Anaheim, CA | Whittier city | 18.2% | 17.2% |
| Los Angeles--Long Beach--Anaheim, CA | Willowbrook CDP | 34.7% | 17.2% |
| Los Angeles--Long Beach--Anaheim, CA | Yorba Linda city | 9.8% | 17.2% |
| Louisville/Jefferson County, KY--IN | Anchorage city | 65.6% | 34.9% |
| Louisville/Jefferson County, KY--IN | Audubon Park city | 54.1% | 34.9% |
| Louisville/Jefferson County, KY--IN | Bancroft city | 39.8% | 34.9% |
| Louisville/Jefferson County, KY--IN | Barbourmeade city | 32.3% | 34.9% |
| Louisville/Jefferson County, KY--IN | Beechwood Village city | 61.7% | 34.9% |
| Louisville/Jefferson County, KY--IN | Bellemeade city | 41.7% | 34.9% |
| Louisville/Jefferson County, KY--IN | Bellewood city | 72.5% | 34.9% |
| Louisville/Jefferson County, KY--IN | Blue Ridge Manor city | 40.6% | 34.9% |
| Louisville/Jefferson County, KY--IN | Briarwood city | 25.1% | 34.9% |
| Louisville/Jefferson County, KY--IN | Broeck Pointe city | 36.8% | 34.9% |
| Louisville/Jefferson County, KY--IN | Brooks CDP | 28.2% | 34.9% |
| Louisville/Jefferson County, KY--IN | Brownsboro Farm city | 35.3% | 34.9% |
| Louisville/Jefferson County, KY--IN | Brownsboro Village city | 62.6% | 34.9% |
| Louisville/Jefferson County, KY--IN | Buckner CDP | 30.0% | 34.9% |
| Louisville/Jefferson County, KY--IN | Cambridge city | 50.6% | 34.9% |
| Louisville/Jefferson County, KY--IN | Charlestown city | 62.8% | 34.9% |
| Louisville/Jefferson County, KY--IN | Clarksville town | 50.7% | 34.9% |
| Louisville/Jefferson County, KY--IN | Coldstream city | 32.7% | 34.9% |
| Louisville/Jefferson County, KY--IN | Creekside city | 29.7% | 34.9% |
| Louisville/Jefferson County, KY--IN | Crestwood city | 35.2% | 34.9% |
| Louisville/Jefferson County, KY--IN | Crossgate city | 71.2% | 34.9% |
| Louisville/Jefferson County, KY--IN | Douglass Hills city | 40.7% | 34.9% |
| Louisville/Jefferson County, KY--IN | Druid Hills city | 76.4% | 34.9% |
| Louisville/Jefferson County, KY--IN | Fincastle city | 42.1% | 34.9% |
| Louisville/Jefferson County, KY--IN | Forest Hills city | 38.5% | 34.9% |
| Louisville/Jefferson County, KY--IN | Fox Chase city | 54.2% | 34.9% |
| Louisville/Jefferson County, KY--IN | Galena CDP | 70.1% | 34.9% |
| Louisville/Jefferson County, KY--IN | Georgetown town | 49.8% | 34.9% |
| Louisville/Jefferson County, KY--IN | Glenview Hills city | 70.2% | 34.9% |
| Louisville/Jefferson County, KY--IN | Glenview Manor city | 70.7% | 34.9% |
| Louisville/Jefferson County, KY--IN | Glenview city | 85.8% | 34.9% |
| Louisville/Jefferson County, KY--IN | Goose Creek city | 38.4% | 34.9% |
| Louisville/Jefferson County, KY--IN | Goshen city | 71.7% | 34.9% |
| Louisville/Jefferson County, KY--IN | Graymoor-Devondale city | 30.9% | 34.9% |
| Louisville/Jefferson County, KY--IN | Green Spring city | 36.6% | 34.9% |
| Louisville/Jefferson County, KY--IN | Hebron Estates city | 21.0% | 34.9% |
| Louisville/Jefferson County, KY--IN | Heritage Creek city | 7.2% | 34.9% |
| Louisville/Jefferson County, KY--IN | Hickory Hill city | 28.3% | 34.9% |
| Louisville/Jefferson County, KY--IN | Hills and Dales city | 83.8% | 34.9% |
| Louisville/Jefferson County, KY--IN | Hillview city | 19.7% | 34.9% |
| Louisville/Jefferson County, KY--IN | Hollow Creek city | 28.0% | 34.9% |
| Louisville/Jefferson County, KY--IN | Hollyvilla city | 63.5% | 34.9% |
| Louisville/Jefferson County, KY--IN | Houston Acres city | 38.7% | 34.9% |
| Louisville/Jefferson County, KY--IN | Hunters Hollow city | 22.1% | 34.9% |
| Louisville/Jefferson County, KY--IN | Hurstbourne Acres city | 25.4% | 34.9% |
| Louisville/Jefferson County, KY--IN | Hurstbourne city | 31.1% | 34.9% |
| Louisville/Jefferson County, KY--IN | Indian Hills city | 73.9% | 34.9% |
| Louisville/Jefferson County, KY--IN | Jeffersontown city | 32.9% | 34.9% |
| Louisville/Jefferson County, KY--IN | Jeffersonville city | 42.9% | 34.9% |
| Louisville/Jefferson County, KY--IN | Kingsley city | 33.9% | 34.9% |
| Louisville/Jefferson County, KY--IN | La Grange city | 24.1% | 34.9% |
| Louisville/Jefferson County, KY--IN | Langdon Place city | 27.6% | 34.9% |
| Louisville/Jefferson County, KY--IN | Lincolnshire city | 42.2% | 34.9% |
| Louisville/Jefferson County, KY--IN | Louisville city | 33.0% | 34.9% |
| Louisville/Jefferson County, KY--IN | Louisville/Jefferson County metro govern | 32.2% | 34.9% |
| Louisville/Jefferson County, KY--IN | Lyndon city | 29.5% | 34.9% |
| Louisville/Jefferson County, KY--IN | Lynnview city | 34.9% | 34.9% |
| Louisville/Jefferson County, KY--IN | Manor Creek city | 32.5% | 34.9% |
| Louisville/Jefferson County, KY--IN | Maryhill Estates city | 73.0% | 34.9% |
| Louisville/Jefferson County, KY--IN | Meadow Vale city | 37.0% | 34.9% |
| Louisville/Jefferson County, KY--IN | Meadowbrook Farm city | 40.4% | 34.9% |
| Louisville/Jefferson County, KY--IN | Meadowview Estates city | 34.7% | 34.9% |
| Louisville/Jefferson County, KY--IN | Middletown city | 32.5% | 34.9% |
| Louisville/Jefferson County, KY--IN | Mockingbird Valley city | 82.6% | 34.9% |
| Louisville/Jefferson County, KY--IN | Moorland city | 45.0% | 34.9% |
| Louisville/Jefferson County, KY--IN | Mount Washington city | 21.1% | 34.9% |
| Louisville/Jefferson County, KY--IN | Murray Hill city | 47.3% | 34.9% |
| Louisville/Jefferson County, KY--IN | New Albany city | 44.9% | 34.9% |
| Louisville/Jefferson County, KY--IN | Norbourne Estates city | 52.9% | 34.9% |
| Louisville/Jefferson County, KY--IN | Northfield city | 71.0% | 34.9% |
| Louisville/Jefferson County, KY--IN | Norwood city | 39.5% | 34.9% |
| Louisville/Jefferson County, KY--IN | Old Brownsboro Place city | 32.6% | 34.9% |
| Louisville/Jefferson County, KY--IN | Orchard Grass Hills city | 45.8% | 34.9% |
| Louisville/Jefferson County, KY--IN | Parkway Village city | 30.1% | 34.9% |
| Louisville/Jefferson County, KY--IN | Pewee Valley city | 68.5% | 34.9% |
| Louisville/Jefferson County, KY--IN | Pioneer Village city | 14.8% | 34.9% |
| Louisville/Jefferson County, KY--IN | Plantation city | 24.8% | 34.9% |
| Louisville/Jefferson County, KY--IN | Poplar Hills city | 23.8% | 34.9% |
| Louisville/Jefferson County, KY--IN | Prospect city | 52.6% | 34.9% |
| Louisville/Jefferson County, KY--IN | Richlawn city | 62.5% | 34.9% |
| Louisville/Jefferson County, KY--IN | River Bluff city | 74.5% | 34.9% |
| Louisville/Jefferson County, KY--IN | Riverwood city | 77.7% | 34.9% |
| Louisville/Jefferson County, KY--IN | Rolling Fields city | 71.7% | 34.9% |
| Louisville/Jefferson County, KY--IN | Rolling Hills city | 26.3% | 34.9% |
| Louisville/Jefferson County, KY--IN | Sellersburg town | 56.0% | 34.9% |
| Louisville/Jefferson County, KY--IN | Seneca Gardens city | 37.4% | 34.9% |
| Louisville/Jefferson County, KY--IN | Shepherdsville city | 18.9% | 34.9% |
| Louisville/Jefferson County, KY--IN | Shively city | 37.8% | 34.9% |
| Louisville/Jefferson County, KY--IN | Spring Mill city | 38.3% | 34.9% |
| Louisville/Jefferson County, KY--IN | Spring Valley city | 51.4% | 34.9% |
| Louisville/Jefferson County, KY--IN | St. Matthews city | 34.0% | 34.9% |
| Louisville/Jefferson County, KY--IN | St. Regis Park city | 34.7% | 34.9% |
| Louisville/Jefferson County, KY--IN | Strathmoor Manor city | 43.3% | 34.9% |
| Louisville/Jefferson County, KY--IN | Strathmoor Village city | 34.6% | 34.9% |
| Louisville/Jefferson County, KY--IN | Sycamore city | 19.4% | 34.9% |
| Louisville/Jefferson County, KY--IN | Ten Broeck city | 73.9% | 34.9% |
| Louisville/Jefferson County, KY--IN | Thornhill city | 76.6% | 34.9% |
| Louisville/Jefferson County, KY--IN | Utica town | 52.5% | 34.9% |
| Louisville/Jefferson County, KY--IN | Watterson Park city | 12.8% | 34.9% |
| Louisville/Jefferson County, KY--IN | Wellington city | 28.1% | 34.9% |
| Louisville/Jefferson County, KY--IN | West Buechel city | 11.6% | 34.9% |
| Louisville/Jefferson County, KY--IN | Westwood city | 31.4% | 34.9% |
| Louisville/Jefferson County, KY--IN | Wildwood city | 43.1% | 34.9% |
| Louisville/Jefferson County, KY--IN | Windy Hills city | 70.2% | 34.9% |
| Louisville/Jefferson County, KY--IN | Woodland Hills city | 33.7% | 34.9% |
| Louisville/Jefferson County, KY--IN | Woodlawn Park city | 57.4% | 34.9% |
| Louisville/Jefferson County, KY--IN | Worthington Hills city | 30.4% | 34.9% |
| McAllen, TX | Abram CDP | 18.3% | 6.9% |
| McAllen, TX | Alamo city | 5.6% | 6.9% |
| McAllen, TX | Alton city | 6.6% | 6.9% |
| McAllen, TX | Citrus City CDP | 5.8% | 6.9% |
| McAllen, TX | CÃ©sar ChÃ¡vez CDP | 13.1% | 6.9% |
| McAllen, TX | Doffing CDP | 8.1% | 6.9% |
| McAllen, TX | Donna city | 13.8% | 6.9% |
| McAllen, TX | Doolittle CDP | 10.0% | 6.9% |
| McAllen, TX | Edcouch city | 14.9% | 6.9% |
| McAllen, TX | Edinburg city | 5.4% | 6.9% |
| McAllen, TX | Elsa city | 15.1% | 6.9% |
| McAllen, TX | Faysville CDP | 2.7% | 6.9% |
| McAllen, TX | Granjeno city | 4.1% | 6.9% |
| McAllen, TX | Havana CDP | 10.3% | 6.9% |
| McAllen, TX | Heidelberg CDP | 7.6% | 6.9% |
| McAllen, TX | Hidalgo city | 5.4% | 6.9% |
| McAllen, TX | Indian Hills CDP | 12.4% | 6.9% |
| McAllen, TX | La Blanca CDP | 7.7% | 6.9% |
| McAllen, TX | La Homa CDP | 5.9% | 6.9% |
| McAllen, TX | La Joya city | 10.5% | 6.9% |
| McAllen, TX | La Villa city | 19.1% | 6.9% |
| McAllen, TX | Laguna Seca CDP | 3.8% | 6.9% |
| McAllen, TX | Llano Grande CDP | 23.4% | 6.9% |
| McAllen, TX | Lopezville CDP | 9.3% | 6.9% |
| McAllen, TX | McAllen city | 8.3% | 6.9% |
| McAllen, TX | Mercedes city | 6.6% | 6.9% |
| McAllen, TX | Midway North CDP | 12.6% | 6.9% |
| McAllen, TX | Midway South CDP | 9.2% | 6.9% |
| McAllen, TX | Mila Doce CDP | 10.3% | 6.9% |
| McAllen, TX | Mission city | 6.8% | 6.9% |
| McAllen, TX | Muniz CDP | 5.8% | 6.9% |
| McAllen, TX | Murillo CDP | 5.8% | 6.9% |
| McAllen, TX | North Alamo CDP | 6.5% | 6.9% |
| McAllen, TX | Olivarez CDP | 12.1% | 6.9% |
| McAllen, TX | Palmhurst city | 10.3% | 6.9% |
| McAllen, TX | Palmview South CDP | 8.9% | 6.9% |
| McAllen, TX | Palmview city | 6.9% | 6.9% |
| McAllen, TX | Penitas city | 7.8% | 6.9% |
| McAllen, TX | Perezville CDP | 5.2% | 6.9% |
| McAllen, TX | Pharr city | 5.2% | 6.9% |
| McAllen, TX | Progreso city | 5.9% | 6.9% |
| McAllen, TX | San Carlos CDP | 6.8% | 6.9% |
| McAllen, TX | San Juan city | 6.6% | 6.9% |
| McAllen, TX | Scissors CDP | 6.9% | 6.9% |
| McAllen, TX | South Alamo CDP | 4.5% | 6.9% |
| McAllen, TX | Villa Verde CDP | 20.3% | 6.9% |
| McAllen, TX | Weslaco city | 10.1% | 6.9% |
| McAllen, TX | West Sharyland CDP | 6.6% | 6.9% |
| Memphis, TN--MS--AR | Bartlett city | 55.3% | 53.5% |
| Memphis, TN--MS--AR | Bridgetown CDP | 69.3% | 53.5% |
| Memphis, TN--MS--AR | Clarkedale city | 76.2% | 53.5% |
| Memphis, TN--MS--AR | Collierville town | 55.3% | 53.5% |
| Memphis, TN--MS--AR | Germantown city | 69.2% | 53.5% |
| Memphis, TN--MS--AR | Hernando city | 46.6% | 53.5% |
| Memphis, TN--MS--AR | Horn Lake city | 52.1% | 53.5% |
| Memphis, TN--MS--AR | Lakeland city | 62.1% | 53.5% |
| Memphis, TN--MS--AR | Lynchburg CDP | 56.6% | 53.5% |
| Memphis, TN--MS--AR | Marion city | 60.1% | 53.5% |
| Memphis, TN--MS--AR | Memphis city | 53.5% | 53.5% |
| Memphis, TN--MS--AR | Millington city | 49.1% | 53.5% |
| Memphis, TN--MS--AR | Olive Branch city | 45.6% | 53.5% |
| Memphis, TN--MS--AR | Piperton city | 71.2% | 53.5% |
| Memphis, TN--MS--AR | Southaven city | 44.6% | 53.5% |
| Memphis, TN--MS--AR | Sunset town | 58.4% | 53.5% |
| Memphis, TN--MS--AR | Walls town | 15.2% | 53.5% |
| Memphis, TN--MS--AR | West Memphis city | 54.2% | 53.5% |
| Miami, FL | Acacia Villas CDP | 35.4% | 30.2% |
| Miami, FL | Atlantis city | 44.1% | 30.2% |
| Miami, FL | Aventura city | 37.5% | 30.2% |
| Miami, FL | Bal Harbour village | 23.2% | 30.2% |
| Miami, FL | Bay Harbor Islands town | 22.1% | 30.2% |
| Miami, FL | Biscayne Park village | 55.3% | 30.2% |
| Miami, FL | Boca Raton city | 45.0% | 30.2% |
| Miami, FL | Boulevard Gardens CDP | 31.8% | 30.2% |
| Miami, FL | Boynton Beach city | 40.7% | 30.2% |
| Miami, FL | Briny Breezes town | 2.7% | 30.2% |
| Miami, FL | Broadview Park CDP | 30.8% | 30.2% |
| Miami, FL | Brownsville CDP | 26.2% | 30.2% |
| Miami, FL | Cabana Colony CDP | 38.4% | 30.2% |
| Miami, FL | Cloud Lake town | 71.1% | 30.2% |
| Miami, FL | Coconut Creek city | 49.0% | 30.2% |
| Miami, FL | Cooper City city | 46.9% | 30.2% |
| Miami, FL | Coral Gables city | 10.5% | 30.2% |
| Miami, FL | Coral Springs city | 45.5% | 30.2% |
| Miami, FL | Coral Terrace CDP | 6.2% | 30.2% |
| Miami, FL | Country Club CDP | 32.2% | 30.2% |
| Miami, FL | Country Walk CDP | 20.6% | 30.2% |
| Miami, FL | Cutler Bay town | 16.3% | 30.2% |
| Miami, FL | Dania Beach city | 33.7% | 30.2% |
| Miami, FL | Davie town | 44.6% | 30.2% |
| Miami, FL | Deerfield Beach city | 35.8% | 30.2% |
| Miami, FL | Delray Beach city | 43.3% | 30.2% |
| Miami, FL | Doral city | 15.7% | 30.2% |
| Miami, FL | El Portal village | 41.8% | 30.2% |
| Miami, FL | Fisher Island CDP | 45.9% | 30.2% |
| Miami, FL | Florida City city | 26.8% | 30.2% |
| Miami, FL | Fort Lauderdale city | 33.3% | 30.2% |
| Miami, FL | Fountainebleau CDP | 20.4% | 30.2% |
| Miami, FL | Franklin Park CDP | 19.9% | 30.2% |
| Miami, FL | Gladeview CDP | 21.4% | 30.2% |
| Miami, FL | Glen Ridge town | 65.8% | 30.2% |
| Miami, FL | Glenvar Heights CDP | 7.0% | 30.2% |
| Miami, FL | Golden Beach town | 41.3% | 30.2% |
| Miami, FL | Golden Glades CDP | 41.9% | 30.2% |
| Miami, FL | Golf village | 64.5% | 30.2% |
| Miami, FL | Goulds CDP | 22.2% | 30.2% |
| Miami, FL | Greenacres city | 38.1% | 30.2% |
| Miami, FL | Gulf Stream town | 53.2% | 30.2% |
| Miami, FL | Gun Club Estates CDP | 34.2% | 30.2% |
| Miami, FL | Hallandale Beach city | 26.2% | 30.2% |
| Miami, FL | Haverhill town | 54.1% | 30.2% |
| Miami, FL | Hialeah Gardens city | 4.2% | 30.2% |
| Miami, FL | Hialeah city | 13.8% | 30.2% |
| Miami, FL | Highland Beach town | 49.2% | 30.2% |
| Miami, FL | Hillsboro Beach town | 47.2% | 30.2% |
| Miami, FL | Hillsboro Pines CDP | 48.9% | 30.2% |
| Miami, FL | Hollywood city | 31.0% | 30.2% |
| Miami, FL | Homestead Base CDP | 14.8% | 30.2% |
| Miami, FL | Homestead city | 26.4% | 30.2% |
| Miami, FL | Hypoluxo town | 31.0% | 30.2% |
| Miami, FL | Indian Creek village | 35.3% | 30.2% |
| Miami, FL | Ives Estates CDP | 29.9% | 30.2% |
| Miami, FL | Juno Beach town | 27.6% | 30.2% |
| Miami, FL | Juno Ridge CDP | 34.3% | 30.2% |
| Miami, FL | Jupiter Inlet Colony town | 25.2% | 30.2% |
| Miami, FL | Jupiter town | 41.5% | 30.2% |
| Miami, FL | Kendale Lakes CDP | 19.2% | 30.2% |
| Miami, FL | Kendall CDP | 25.1% | 30.2% |
| Miami, FL | Kendall West CDP | 16.4% | 30.2% |
| Miami, FL | Kenwood Estates CDP | 44.1% | 30.2% |
| Miami, FL | Key Biscayne village | 36.1% | 30.2% |
| Miami, FL | Lake Belvedere Estates CDP | 39.0% | 30.2% |
| Miami, FL | Lake Clarke Shores town | 50.4% | 30.2% |
| Miami, FL | Lake Park town | 29.1% | 30.2% |
| Miami, FL | Lake Worth city | 26.2% | 30.2% |
| Miami, FL | Lantana town | 22.6% | 30.2% |
| Miami, FL | Lauderdale Lakes city | 26.7% | 30.2% |
| Miami, FL | Lauderdale-by-the-Sea town | 32.6% | 30.2% |
| Miami, FL | Lauderhill city | 31.6% | 30.2% |
| Miami, FL | Lazy Lake village | 69.3% | 30.2% |
| Miami, FL | Leisure City CDP | 30.9% | 30.2% |
| Miami, FL | Lighthouse Point city | 35.6% | 30.2% |
| Miami, FL | Limestone Creek CDP | 49.6% | 30.2% |
| Miami, FL | Loxahatchee Groves town | 59.9% | 30.2% |
| Miami, FL | Manalapan town | 33.3% | 30.2% |
| Miami, FL | Mangonia Park town | 31.5% | 30.2% |
| Miami, FL | Margate city | 34.8% | 30.2% |
| Miami, FL | Medley town | 2.6% | 30.2% |
| Miami, FL | Miami Beach city | 1.5% | 30.2% |
| Miami, FL | Miami Gardens city | 30.8% | 30.2% |
| Miami, FL | Miami Lakes town | 33.9% | 30.2% |
| Miami, FL | Miami Shores village | 36.6% | 30.2% |
| Miami, FL | Miami Springs city | 35.5% | 30.2% |
| Miami, FL | Miami city | 13.8% | 30.2% |
| Miami, FL | Miramar city | 36.9% | 30.2% |
| Miami, FL | Naranja CDP | 29.8% | 30.2% |
| Miami, FL | North Bay Village city | 13.5% | 30.2% |
| Miami, FL | North Lauderdale city | 34.3% | 30.2% |
| Miami, FL | North Miami Beach city | 25.9% | 30.2% |
| Miami, FL | North Miami city | 32.2% | 30.2% |
| Miami, FL | North Palm Beach village | 34.3% | 30.2% |
| Miami, FL | Oakland Park city | 30.0% | 30.2% |
| Miami, FL | Ocean Ridge town | 33.9% | 30.2% |
| Miami, FL | Ojus CDP | 25.2% | 30.2% |
| Miami, FL | Olympia Heights CDP | 20.8% | 30.2% |
| Miami, FL | Opa-locka city | 26.5% | 30.2% |
| Miami, FL | Palm Beach Gardens city | 43.9% | 30.2% |
| Miami, FL | Palm Beach Shores town | 12.2% | 30.2% |
| Miami, FL | Palm Beach town | 25.6% | 30.2% |
| Miami, FL | Palm Springs North CDP | 22.6% | 30.2% |
| Miami, FL | Palm Springs village | 38.8% | 30.2% |
| Miami, FL | Palmetto Bay village | 23.2% | 30.2% |
| Miami, FL | Palmetto Estates CDP | 24.0% | 30.2% |
| Miami, FL | Parkland city | 53.2% | 30.2% |
| Miami, FL | Pembroke Park town | 25.5% | 30.2% |
| Miami, FL | Pembroke Pines city | 38.4% | 30.2% |
| Miami, FL | Pine Air CDP | 42.6% | 30.2% |
| Miami, FL | Pinecrest village | 27.4% | 30.2% |
| Miami, FL | Pinewood CDP | 31.4% | 30.2% |
| Miami, FL | Plantation Mobile Home Park CDP | 32.1% | 30.2% |
| Miami, FL | Plantation city | 49.6% | 30.2% |
| Miami, FL | Pompano Beach city | 32.3% | 30.2% |
| Miami, FL | Princeton CDP | 14.9% | 30.2% |
| Miami, FL | Richmond Heights CDP | 21.2% | 30.2% |
| Miami, FL | Richmond West CDP | 9.2% | 30.2% |
| Miami, FL | Riviera Beach city | 26.7% | 30.2% |
| Miami, FL | Roosevelt Gardens CDP | 25.2% | 30.2% |
| Miami, FL | Royal Palm Beach village | 31.8% | 30.2% |
| Miami, FL | Royal Palm Estates CDP | 41.5% | 30.2% |
| Miami, FL | San Castle CDP | 34.2% | 30.2% |
| Miami, FL | Schall Circle CDP | 43.5% | 30.2% |
| Miami, FL | Sea Ranch Lakes village | 38.6% | 30.2% |
| Miami, FL | Seminole Manor CDP | 48.5% | 30.2% |
| Miami, FL | South Miami Heights CDP | 18.5% | 30.2% |
| Miami, FL | South Miami city | 5.0% | 30.2% |
| Miami, FL | South Palm Beach town | 4.4% | 30.2% |
| Miami, FL | Southwest Ranches town | 64.6% | 30.2% |
| Miami, FL | Stacey Street CDP | 39.1% | 30.2% |
| Miami, FL | Sunny Isles Beach city | 32.0% | 30.2% |
| Miami, FL | Sunrise city | 37.8% | 30.2% |
| Miami, FL | Sunset CDP | 21.6% | 30.2% |
| Miami, FL | Surfside town | 17.4% | 30.2% |
| Miami, FL | Sweetwater city | 12.8% | 30.2% |
| Miami, FL | Tamarac city | 38.0% | 30.2% |
| Miami, FL | Tamiami CDP | 13.1% | 30.2% |
| Miami, FL | Tequesta village | 26.6% | 30.2% |
| Miami, FL | The Acreage CDP | 53.0% | 30.2% |
| Miami, FL | The Crossings CDP | 20.8% | 30.2% |
| Miami, FL | The Hammocks CDP | 16.4% | 30.2% |
| Miami, FL | Three Lakes CDP | 23.3% | 30.2% |
| Miami, FL | University Park CDP | 18.5% | 30.2% |
| Miami, FL | Virginia Gardens village | 24.7% | 30.2% |
| Miami, FL | Washington Park CDP | 32.4% | 30.2% |
| Miami, FL | Watergate CDP | 36.3% | 30.2% |
| Miami, FL | Wellington village | 38.8% | 30.2% |
| Miami, FL | West Little River CDP | 21.5% | 30.2% |
| Miami, FL | West Miami city | 6.1% | 30.2% |
| Miami, FL | West Palm Beach city | 33.6% | 30.2% |
| Miami, FL | West Park city | 27.9% | 30.2% |
| Miami, FL | West Perrine CDP | 24.0% | 30.2% |
| Miami, FL | Westchester CDP | 14.2% | 30.2% |
| Miami, FL | Westgate CDP | 40.2% | 30.2% |
| Miami, FL | Weston city | 40.1% | 30.2% |
| Miami, FL | Westview CDP | 37.2% | 30.2% |
| Miami, FL | Westwood Lakes CDP | 25.1% | 30.2% |
| Miami, FL | Wilton Manors city | 33.4% | 30.2% |
| Milwaukee, WI | Bayside village | 59.3% | 31.8% |
| Milwaukee, WI | Big Bend village | 50.5% | 31.8% |
| Milwaukee, WI | Brookfield city | 60.0% | 31.8% |
| Milwaukee, WI | Brown Deer village | 44.2% | 31.8% |
| Milwaukee, WI | Butler village | 26.2% | 31.8% |
| Milwaukee, WI | Caledonia village | 40.4% | 31.8% |
| Milwaukee, WI | Cedarburg city | 47.5% | 31.8% |
| Milwaukee, WI | Chenequa village | 43.0% | 31.8% |
| Milwaukee, WI | Cudahy city | 18.4% | 31.8% |
| Milwaukee, WI | Delafield city | 59.7% | 31.8% |
| Milwaukee, WI | Dousman village | 44.6% | 31.8% |
| Milwaukee, WI | Elm Grove village | 64.8% | 31.8% |
| Milwaukee, WI | Fox Point village | 57.6% | 31.8% |
| Milwaukee, WI | Franklin city | 45.0% | 31.8% |
| Milwaukee, WI | Germantown village | 44.9% | 31.8% |
| Milwaukee, WI | Glendale city | 47.4% | 31.8% |
| Milwaukee, WI | Grafton village | 38.8% | 31.8% |
| Milwaukee, WI | Greendale village | 49.6% | 31.8% |
| Milwaukee, WI | Greenfield city | 36.8% | 31.8% |
| Milwaukee, WI | Hales Corners village | 53.9% | 31.8% |
| Milwaukee, WI | Hartland village | 43.9% | 31.8% |
| Milwaukee, WI | Ixonia CDP | 37.8% | 31.8% |
| Milwaukee, WI | Lac La Belle village | 82.1% | 31.8% |
| Milwaukee, WI | Lannon village | 38.5% | 31.8% |
| Milwaukee, WI | Menomonee Falls village | 45.3% | 31.8% |
| Milwaukee, WI | Mequon city | 61.7% | 31.8% |
| Milwaukee, WI | Merton village | 54.4% | 31.8% |
| Milwaukee, WI | Milwaukee city | 21.0% | 31.8% |
| Milwaukee, WI | Muskego city | 48.6% | 31.8% |
| Milwaukee, WI | Nashotah village | 52.4% | 31.8% |
| Milwaukee, WI | New Berlin city | 51.0% | 31.8% |
| Milwaukee, WI | Oak Creek city | 36.3% | 31.8% |
| Milwaukee, WI | Oconomowoc Lake village | 76.8% | 31.8% |
| Milwaukee, WI | Oconomowoc city | 41.4% | 31.8% |
| Milwaukee, WI | Okauchee Lake CDP | 59.0% | 31.8% |
| Milwaukee, WI | Pewaukee city | 51.9% | 31.8% |
| Milwaukee, WI | Pewaukee village | 42.0% | 31.8% |
| Milwaukee, WI | Port Washington city | 36.5% | 31.8% |
| Milwaukee, WI | Richfield village | 66.7% | 31.8% |
| Milwaukee, WI | River Hills village | 73.3% | 31.8% |
| Milwaukee, WI | Saukville village | 41.7% | 31.8% |
| Milwaukee, WI | Shorewood village | 24.2% | 31.8% |
| Milwaukee, WI | South Milwaukee city | 21.6% | 31.8% |
| Milwaukee, WI | St. Francis city | 25.8% | 31.8% |
| Milwaukee, WI | Summit village | 65.9% | 31.8% |
| Milwaukee, WI | Sussex village | 46.9% | 31.8% |
| Milwaukee, WI | Thiensville village | 52.5% | 31.8% |
| Milwaukee, WI | Tichigan CDP | 54.4% | 31.8% |
| Milwaukee, WI | Wales village | 62.2% | 31.8% |
| Milwaukee, WI | Waukesha city | 36.4% | 31.8% |
| Milwaukee, WI | Wauwatosa city | 38.7% | 31.8% |
| Milwaukee, WI | West Allis city | 21.6% | 31.8% |
| Milwaukee, WI | West Milwaukee village | 8.7% | 31.8% |
| Milwaukee, WI | Whitefish Bay village | 39.1% | 31.8% |
| Milwaukee, WI | Wind Lake CDP | 57.7% | 31.8% |
| Minneapolis--St. Paul, MN--WI | Albertville city | 47.3% | 47.5% |
| Minneapolis--St. Paul, MN--WI | Andover city | 55.4% | 47.5% |
| Minneapolis--St. Paul, MN--WI | Anoka city | 45.5% | 47.5% |
| Minneapolis--St. Paul, MN--WI | Apple Valley city | 46.5% | 47.5% |
| Minneapolis--St. Paul, MN--WI | Arden Hills city | 57.7% | 47.5% |
| Minneapolis--St. Paul, MN--WI | Bayport city | 31.2% | 47.5% |
| Minneapolis--St. Paul, MN--WI | Birchwood Village city | 74.0% | 47.5% |
| Minneapolis--St. Paul, MN--WI | Blaine city | 56.5% | 47.5% |
| Minneapolis--St. Paul, MN--WI | Bloomington city | 49.2% | 47.5% |
| Minneapolis--St. Paul, MN--WI | Brooklyn Center city | 47.7% | 47.5% |
| Minneapolis--St. Paul, MN--WI | Brooklyn Park city | 45.5% | 47.5% |
| Minneapolis--St. Paul, MN--WI | Burnsville city | 48.4% | 47.5% |
| Minneapolis--St. Paul, MN--WI | Carver city | 59.1% | 47.5% |
| Minneapolis--St. Paul, MN--WI | Centerville city | 49.0% | 47.5% |
| Minneapolis--St. Paul, MN--WI | Champlin city | 54.9% | 47.5% |
| Minneapolis--St. Paul, MN--WI | Chanhassen city | 58.8% | 47.5% |
| Minneapolis--St. Paul, MN--WI | Chaska city | 47.7% | 47.5% |
| Minneapolis--St. Paul, MN--WI | Circle Pines city | 59.9% | 47.5% |
| Minneapolis--St. Paul, MN--WI | Columbia Heights city | 49.5% | 47.5% |
| Minneapolis--St. Paul, MN--WI | Coon Rapids city | 55.5% | 47.5% |
| Minneapolis--St. Paul, MN--WI | Corcoran city | 51.8% | 47.5% |
| Minneapolis--St. Paul, MN--WI | Cottage Grove city | 49.3% | 47.5% |
| Minneapolis--St. Paul, MN--WI | Crystal city | 52.2% | 47.5% |
| Minneapolis--St. Paul, MN--WI | Dayton city | 57.1% | 47.5% |
| Minneapolis--St. Paul, MN--WI | Deephaven city | 75.9% | 47.5% |
| Minneapolis--St. Paul, MN--WI | Dellwood city | 91.4% | 47.5% |
| Minneapolis--St. Paul, MN--WI | Eagan city | 49.5% | 47.5% |
| Minneapolis--St. Paul, MN--WI | East Bethel city | 58.1% | 47.5% |
| Minneapolis--St. Paul, MN--WI | Eden Prairie city | 56.8% | 47.5% |
| Minneapolis--St. Paul, MN--WI | Edina city | 51.6% | 47.5% |
| Minneapolis--St. Paul, MN--WI | Elk River city | 49.9% | 47.5% |
| Minneapolis--St. Paul, MN--WI | Excelsior city | 49.6% | 47.5% |
| Minneapolis--St. Paul, MN--WI | Falcon Heights city | 41.6% | 47.5% |
| Minneapolis--St. Paul, MN--WI | Farmington city | 40.4% | 47.5% |
| Minneapolis--St. Paul, MN--WI | Fridley city | 57.1% | 47.5% |
| Minneapolis--St. Paul, MN--WI | Gem Lake city | 73.2% | 47.5% |
| Minneapolis--St. Paul, MN--WI | Golden Valley city | 60.9% | 47.5% |
| Minneapolis--St. Paul, MN--WI | Grant city | 73.0% | 47.5% |
| Minneapolis--St. Paul, MN--WI | Greenwood city | 75.8% | 47.5% |
| Minneapolis--St. Paul, MN--WI | Ham Lake city | 67.0% | 47.5% |
| Minneapolis--St. Paul, MN--WI | Hanover city | 47.8% | 47.5% |
| Minneapolis--St. Paul, MN--WI | Hilltop city | 25.2% | 47.5% |
| Minneapolis--St. Paul, MN--WI | Hopkins city | 43.7% | 47.5% |
| Minneapolis--St. Paul, MN--WI | Houlton CDP | 53.6% | 47.5% |
| Minneapolis--St. Paul, MN--WI | Hugo city | 40.3% | 47.5% |
| Minneapolis--St. Paul, MN--WI | Inver Grove Heights city | 51.3% | 47.5% |
| Minneapolis--St. Paul, MN--WI | Lake Elmo city | 62.6% | 47.5% |
| Minneapolis--St. Paul, MN--WI | Lakeville city | 45.7% | 47.5% |
| Minneapolis--St. Paul, MN--WI | Landfall city | 23.6% | 47.5% |
| Minneapolis--St. Paul, MN--WI | Lauderdale city | 48.4% | 47.5% |
| Minneapolis--St. Paul, MN--WI | Lexington city | 58.9% | 47.5% |
| Minneapolis--St. Paul, MN--WI | Lilydale city | 33.7% | 47.5% |
| Minneapolis--St. Paul, MN--WI | Lino Lakes city | 53.3% | 47.5% |
| Minneapolis--St. Paul, MN--WI | Little Canada city | 45.8% | 47.5% |
| Minneapolis--St. Paul, MN--WI | Long Lake city | 64.6% | 47.5% |
| Minneapolis--St. Paul, MN--WI | Mahtomedi city | 63.3% | 47.5% |
| Minneapolis--St. Paul, MN--WI | Maple Grove city | 50.5% | 47.5% |
| Minneapolis--St. Paul, MN--WI | Maplewood city | 54.5% | 47.5% |
| Minneapolis--St. Paul, MN--WI | Medicine Lake city | 73.7% | 47.5% |
| Minneapolis--St. Paul, MN--WI | Medina city | 55.5% | 47.5% |
| Minneapolis--St. Paul, MN--WI | Mendota Heights city | 56.0% | 47.5% |
| Minneapolis--St. Paul, MN--WI | Mendota city | 67.3% | 47.5% |
| Minneapolis--St. Paul, MN--WI | Minneapolis city | 36.4% | 47.5% |
| Minneapolis--St. Paul, MN--WI | Minnetonka Beach city | 71.1% | 47.5% |
| Minneapolis--St. Paul, MN--WI | Minnetonka city | 65.9% | 47.5% |
| Minneapolis--St. Paul, MN--WI | Minnetrista city | 58.9% | 47.5% |
| Minneapolis--St. Paul, MN--WI | Mound city | 57.5% | 47.5% |
| Minneapolis--St. Paul, MN--WI | Mounds View city | 58.2% | 47.5% |
| Minneapolis--St. Paul, MN--WI | New Brighton city | 46.9% | 47.5% |
| Minneapolis--St. Paul, MN--WI | New Hope city | 56.2% | 47.5% |
| Minneapolis--St. Paul, MN--WI | Newport city | 59.9% | 47.5% |
| Minneapolis--St. Paul, MN--WI | North Oaks city | 73.6% | 47.5% |
| Minneapolis--St. Paul, MN--WI | North St. Paul city | 47.8% | 47.5% |
| Minneapolis--St. Paul, MN--WI | Nowthen city | 79.7% | 47.5% |
| Minneapolis--St. Paul, MN--WI | Oak Grove city | 50.3% | 47.5% |
| Minneapolis--St. Paul, MN--WI | Oak Park Heights city | 53.8% | 47.5% |
| Minneapolis--St. Paul, MN--WI | Oakdale city | 48.7% | 47.5% |
| Minneapolis--St. Paul, MN--WI | Orono city | 71.0% | 47.5% |
| Minneapolis--St. Paul, MN--WI | Osseo city | 41.9% | 47.5% |
| Minneapolis--St. Paul, MN--WI | Otsego city | 43.9% | 47.5% |
| Minneapolis--St. Paul, MN--WI | Pine Springs city | 79.5% | 47.5% |
| Minneapolis--St. Paul, MN--WI | Plymouth city | 54.1% | 47.5% |
| Minneapolis--St. Paul, MN--WI | Prior Lake city | 51.3% | 47.5% |
| Minneapolis--St. Paul, MN--WI | Ramsey city | 54.9% | 47.5% |
| Minneapolis--St. Paul, MN--WI | Richfield city | 41.7% | 47.5% |
| Minneapolis--St. Paul, MN--WI | Robbinsdale city | 41.6% | 47.5% |
| Minneapolis--St. Paul, MN--WI | Rogers city | 51.5% | 47.5% |
| Minneapolis--St. Paul, MN--WI | Rosemount city | 42.3% | 47.5% |
| Minneapolis--St. Paul, MN--WI | Roseville city | 52.0% | 47.5% |
| Minneapolis--St. Paul, MN--WI | Savage city | 47.7% | 47.5% |
| Minneapolis--St. Paul, MN--WI | Shakopee city | 37.5% | 47.5% |
| Minneapolis--St. Paul, MN--WI | Shoreview city | 53.1% | 47.5% |
| Minneapolis--St. Paul, MN--WI | Shorewood city | 70.1% | 47.5% |
| Minneapolis--St. Paul, MN--WI | South St. Paul city | 38.0% | 47.5% |
| Minneapolis--St. Paul, MN--WI | Spring Lake Park city | 56.2% | 47.5% |
| Minneapolis--St. Paul, MN--WI | Spring Park city | 35.6% | 47.5% |
| Minneapolis--St. Paul, MN--WI | St. Anthony city | 39.9% | 47.5% |
| Minneapolis--St. Paul, MN--WI | St. Bonifacius city | 55.3% | 47.5% |
| Minneapolis--St. Paul, MN--WI | St. Louis Park city | 45.3% | 47.5% |
| Minneapolis--St. Paul, MN--WI | St. Michael city | 46.7% | 47.5% |
| Minneapolis--St. Paul, MN--WI | St. Paul Park city | 47.0% | 47.5% |
| Minneapolis--St. Paul, MN--WI | St. Paul city | 36.0% | 47.5% |
| Minneapolis--St. Paul, MN--WI | Stillwater city | 53.5% | 47.5% |
| Minneapolis--St. Paul, MN--WI | Sunfish Lake city | 72.2% | 47.5% |
| Minneapolis--St. Paul, MN--WI | Tonka Bay city | 63.7% | 47.5% |
| Minneapolis--St. Paul, MN--WI | Vadnais Heights city | 52.1% | 47.5% |
| Minneapolis--St. Paul, MN--WI | Victoria city | 53.0% | 47.5% |
| Minneapolis--St. Paul, MN--WI | Wayzata city | 60.1% | 47.5% |
| Minneapolis--St. Paul, MN--WI | West St. Paul city | 44.1% | 47.5% |
| Minneapolis--St. Paul, MN--WI | White Bear Lake city | 54.2% | 47.5% |
| Minneapolis--St. Paul, MN--WI | Willernie city | 64.1% | 47.5% |
| Minneapolis--St. Paul, MN--WI | Woodbury city | 48.7% | 47.5% |
| Minneapolis--St. Paul, MN--WI | Woodland city | 76.1% | 47.5% |
| Mobile, AL | Axis CDP | 56.1% | 60.1% |
| Mobile, AL | Bayou La Batre city | 70.9% | 60.1% |
| Mobile, AL | Chickasaw city | 58.3% | 60.1% |
| Mobile, AL | Creola city | 65.2% | 60.1% |
| Mobile, AL | Mobile city | 58.6% | 60.1% |
| Mobile, AL | Prichard city | 58.9% | 60.1% |
| Mobile, AL | Saraland city | 63.6% | 60.1% |
| Mobile, AL | Satsuma city | 64.4% | 60.1% |
| Mobile, AL | Semmes city | 68.0% | 60.1% |
| Mobile, AL | Theodore CDP | 66.2% | 60.1% |
| Mobile, AL | Tillmans Corner CDP | 63.6% | 60.1% |
| Myrtle Beach--Socastee, SC--NC | Atlantic Beach town | 41.0% | 41.2% |
| Myrtle Beach--Socastee, SC--NC | Briarcliffe Acres town | 74.0% | 41.2% |
| Myrtle Beach--Socastee, SC--NC | Calabash town | 53.6% | 41.2% |
| Myrtle Beach--Socastee, SC--NC | Carolina Shores town | 45.1% | 41.2% |
| Myrtle Beach--Socastee, SC--NC | Conway city | 37.1% | 41.2% |
| Myrtle Beach--Socastee, SC--NC | Forestbrook CDP | 49.7% | 41.2% |
| Myrtle Beach--Socastee, SC--NC | Garden City CDP | 37.5% | 41.2% |
| Myrtle Beach--Socastee, SC--NC | Little River CDP | 47.6% | 41.2% |
| Myrtle Beach--Socastee, SC--NC | Murrells Inlet CDP | 52.5% | 41.2% |
| Myrtle Beach--Socastee, SC--NC | Myrtle Beach city | 31.2% | 41.2% |
| Myrtle Beach--Socastee, SC--NC | North Myrtle Beach city | 34.5% | 41.2% |
| Myrtle Beach--Socastee, SC--NC | Ocean Isle Beach town | 0.9% | 41.2% |
| Myrtle Beach--Socastee, SC--NC | Red Hill CDP | 35.9% | 41.2% |
| Myrtle Beach--Socastee, SC--NC | Shallotte town | 62.2% | 41.2% |
| Myrtle Beach--Socastee, SC--NC | Socastee CDP | 42.0% | 41.2% |
| Myrtle Beach--Socastee, SC--NC | Sunset Beach town | 55.6% | 41.2% |
| Myrtle Beach--Socastee, SC--NC | Surfside Beach town | 44.7% | 41.2% |
| Nashville-Davidson, TN | Belle Meade city | 92.3% | 62.5% |
| Nashville-Davidson, TN | Berry Hill city | 33.8% | 62.5% |
| Nashville-Davidson, TN | Brentwood city | 69.8% | 62.5% |
| Nashville-Davidson, TN | Forest Hills city | 88.7% | 62.5% |
| Nashville-Davidson, TN | Franklin city | 61.3% | 62.5% |
| Nashville-Davidson, TN | Gallatin city | 50.7% | 62.5% |
| Nashville-Davidson, TN | Goodlettsville city | 73.3% | 62.5% |
| Nashville-Davidson, TN | Green Hill CDP | 77.6% | 62.5% |
| Nashville-Davidson, TN | Greenbrier town | 63.6% | 62.5% |
| Nashville-Davidson, TN | Hendersonville city | 61.3% | 62.5% |
| Nashville-Davidson, TN | La Vergne city | 49.8% | 62.5% |
| Nashville-Davidson, TN | Millersville city | 77.3% | 62.5% |
| Nashville-Davidson, TN | Mount Juliet city | 61.5% | 62.5% |
| Nashville-Davidson, TN | Murfreesboro city | 31.6% | 62.5% |
| Nashville-Davidson, TN | Nashville-Davidson metropolitan governme | 62.5% | 62.5% |
| Nashville-Davidson, TN | Nolensville town | 64.6% | 62.5% |
| Nashville-Davidson, TN | Oak Hill city | 81.4% | 62.5% |
| Nashville-Davidson, TN | Ridgetop city | 84.9% | 62.5% |
| Nashville-Davidson, TN | Shackle Island CDP | 66.5% | 62.5% |
| Nashville-Davidson, TN | Smyrna town | 51.0% | 62.5% |
| Nashville-Davidson, TN | Springfield city | 49.5% | 62.5% |
| Nashville-Davidson, TN | White House city | 57.1% | 62.5% |
| New Haven, CT | Branford Center CDP | 34.4% | 38.9% |
| New Haven, CT | Cheshire Village CDP | 59.7% | 38.9% |
| New Haven, CT | Chester Center CDP | 67.1% | 38.9% |
| New Haven, CT | Clinton CDP | 39.7% | 38.9% |
| New Haven, CT | Deep River Center CDP | 50.7% | 38.9% |
| New Haven, CT | Durham CDP | 63.5% | 38.9% |
| New Haven, CT | East Haven CDP | 35.6% | 38.9% |
| New Haven, CT | Essex Village CDP | 61.3% | 38.9% |
| New Haven, CT | Fenwick borough | 46.2% | 38.9% |
| New Haven, CT | Guilford Center CDP | 47.5% | 38.9% |
| New Haven, CT | Madison Center CDP | 56.8% | 38.9% |
| New Haven, CT | Meriden city | 33.4% | 38.9% |
| New Haven, CT | Middletown city | 74.8% | 38.9% |
| New Haven, CT | New Haven city | 20.3% | 38.9% |
| New Haven, CT | North Haven CDP | 52.5% | 38.9% |
| New Haven, CT | Old Saybrook Center CDP | 46.7% | 38.9% |
| New Haven, CT | Orange CDP | 67.8% | 38.9% |
| New Haven, CT | Saybrook Manor CDP | 38.7% | 38.9% |
| New Haven, CT | Wallingford Center CDP | 26.7% | 38.9% |
| New Haven, CT | West Haven city | 25.7% | 38.9% |
| New Haven, CT | Westbrook Center CDP | 38.7% | 38.9% |
| New Orleans, LA | Ama CDP | 84.4% | 40.8% |
| New Orleans, LA | Arabi CDP | 26.6% | 40.8% |
| New Orleans, LA | Avondale CDP | 40.0% | 40.8% |
| New Orleans, LA | Belle Chasse CDP | 32.2% | 40.8% |
| New Orleans, LA | Boutte CDP | 54.9% | 40.8% |
| New Orleans, LA | Bridge City CDP | 46.9% | 40.8% |
| New Orleans, LA | Chalmette CDP | 17.4% | 40.8% |
| New Orleans, LA | Des Allemands CDP | 56.7% | 40.8% |
| New Orleans, LA | Destrehan CDP | 42.8% | 40.8% |
| New Orleans, LA | Elmwood CDP | 42.2% | 40.8% |
| New Orleans, LA | Estelle CDP | 42.9% | 40.8% |
| New Orleans, LA | Garyville CDP | 29.5% | 40.8% |
| New Orleans, LA | Gretna city | 36.0% | 40.8% |
| New Orleans, LA | Hahnville CDP | 62.3% | 40.8% |
| New Orleans, LA | Harahan city | 49.4% | 40.8% |
| New Orleans, LA | Harvey CDP | 36.0% | 40.8% |
| New Orleans, LA | Jefferson CDP | 45.9% | 40.8% |
| New Orleans, LA | Kenner city | 37.4% | 40.8% |
| New Orleans, LA | Laplace CDP | 43.6% | 40.8% |
| New Orleans, LA | Luling CDP | 44.1% | 40.8% |
| New Orleans, LA | Marrero CDP | 42.3% | 40.8% |
| New Orleans, LA | Meraux CDP | 16.8% | 40.8% |
| New Orleans, LA | Metairie CDP | 38.6% | 40.8% |
| New Orleans, LA | Montz CDP | 60.3% | 40.8% |
| New Orleans, LA | New Orleans city | 42.1% | 40.8% |
| New Orleans, LA | New Sarpy CDP | 57.0% | 40.8% |
| New Orleans, LA | Norco CDP | 37.4% | 40.8% |
| New Orleans, LA | Paradis CDP | 44.3% | 40.8% |
| New Orleans, LA | Poydras CDP | 35.6% | 40.8% |
| New Orleans, LA | Reserve CDP | 56.3% | 40.8% |
| New Orleans, LA | River Ridge CDP | 61.2% | 40.8% |
| New Orleans, LA | St. Rose CDP | 43.3% | 40.8% |
| New Orleans, LA | Terrytown CDP | 42.2% | 40.8% |
| New Orleans, LA | Timberlane CDP | 35.0% | 40.8% |
| New Orleans, LA | Violet CDP | 24.4% | 40.8% |
| New Orleans, LA | Waggaman CDP | 46.4% | 40.8% |
| New Orleans, LA | Westwego city | 40.6% | 40.8% |
| New Orleans, LA | Woodmere CDP | 35.1% | 40.8% |
| New York--Newark, NY--NJ--CT | Airmont village | 59.5% | 16.0% |
| New York--Newark, NY--NJ--CT | Albertson CDP | 22.0% | 16.0% |
| New York--Newark, NY--NJ--CT | Allendale borough | 65.3% | 16.0% |
| New York--Newark, NY--NJ--CT | Allenhurst borough | 24.2% | 16.0% |
| New York--Newark, NY--NJ--CT | Allenwood CDP | 67.4% | 16.0% |
| New York--Newark, NY--NJ--CT | Alpine borough | 69.6% | 16.0% |
| New York--Newark, NY--NJ--CT | Amityville village | 21.3% | 16.0% |
| New York--Newark, NY--NJ--CT | Annandale CDP | 53.1% | 16.0% |
| New York--Newark, NY--NJ--CT | Aquebogue CDP | 37.7% | 16.0% |
| New York--Newark, NY--NJ--CT | Ardsley village | 35.8% | 16.0% |
| New York--Newark, NY--NJ--CT | Armonk CDP | 62.9% | 16.0% |
| New York--Newark, NY--NJ--CT | Asbury Park city | 8.7% | 16.0% |
| New York--Newark, NY--NJ--CT | Asharoken village | 35.0% | 16.0% |
| New York--Newark, NY--NJ--CT | Atlantic Beach village | 1.5% | 16.0% |
| New York--Newark, NY--NJ--CT | Atlantic Highlands borough | 31.7% | 16.0% |
| New York--Newark, NY--NJ--CT | Avenel CDP | 18.4% | 16.0% |
| New York--Newark, NY--NJ--CT | Avon-by-the-Sea borough | 2.7% | 16.0% |
| New York--Newark, NY--NJ--CT | Babylon village | 21.8% | 16.0% |
| New York--Newark, NY--NJ--CT | Baldwin CDP | 18.2% | 16.0% |
| New York--Newark, NY--NJ--CT | Baldwin Harbor CDP | 10.0% | 16.0% |
| New York--Newark, NY--NJ--CT | Bardonia CDP | 60.1% | 16.0% |
| New York--Newark, NY--NJ--CT | Barnegat CDP | 46.2% | 16.0% |
| New York--Newark, NY--NJ--CT | Barnegat Light borough | 4.0% | 16.0% |
| New York--Newark, NY--NJ--CT | Barnum Island CDP | 3.2% | 16.0% |
| New York--Newark, NY--NJ--CT | Baxter Estates village | 55.2% | 16.0% |
| New York--Newark, NY--NJ--CT | Bay Head borough | 15.1% | 16.0% |
| New York--Newark, NY--NJ--CT | Bay Park CDP | 11.9% | 16.0% |
| New York--Newark, NY--NJ--CT | Bay Shore CDP | 24.6% | 16.0% |
| New York--Newark, NY--NJ--CT | Bayonne city | 8.3% | 16.0% |
| New York--Newark, NY--NJ--CT | Bayport CDP | 21.7% | 16.0% |
| New York--Newark, NY--NJ--CT | Bayville village | 6.9% | 16.0% |
| New York--Newark, NY--NJ--CT | Baywood CDP | 40.8% | 16.0% |
| New York--Newark, NY--NJ--CT | Beach Haven West CDP | 0.0% | 16.0% |
| New York--Newark, NY--NJ--CT | Beach Haven borough | 0.2% | 16.0% |
| New York--Newark, NY--NJ--CT | Beachwood borough | 16.6% | 16.0% |
| New York--Newark, NY--NJ--CT | Beattystown CDP | 37.9% | 16.0% |
| New York--Newark, NY--NJ--CT | Bedford Hills CDP | 32.2% | 16.0% |
| New York--Newark, NY--NJ--CT | Belford CDP | 44.1% | 16.0% |
| New York--Newark, NY--NJ--CT | Belle Mead CDP | 61.4% | 16.0% |
| New York--Newark, NY--NJ--CT | Belle Terre village | 32.0% | 16.0% |
| New York--Newark, NY--NJ--CT | Bellerose Terrace CDP | 5.6% | 16.0% |
| New York--Newark, NY--NJ--CT | Bellerose village | 39.2% | 16.0% |
| New York--Newark, NY--NJ--CT | Bellmore CDP | 6.1% | 16.0% |
| New York--Newark, NY--NJ--CT | Bellport village | 27.2% | 16.0% |
| New York--Newark, NY--NJ--CT | Belmar borough | 1.8% | 16.0% |
| New York--Newark, NY--NJ--CT | Bergenfield borough | 29.0% | 16.0% |
| New York--Newark, NY--NJ--CT | Bernardsville borough | 56.2% | 16.0% |
| New York--Newark, NY--NJ--CT | Bethpage CDP | 8.7% | 16.0% |
| New York--Newark, NY--NJ--CT | Blackwells Mills CDP | 38.7% | 16.0% |
| New York--Newark, NY--NJ--CT | Blauvelt CDP | 52.2% | 16.0% |
| New York--Newark, NY--NJ--CT | Blawenburg CDP | 50.1% | 16.0% |
| New York--Newark, NY--NJ--CT | Bloomingdale borough | 52.4% | 16.0% |
| New York--Newark, NY--NJ--CT | Blue Point CDP | 17.7% | 16.0% |
| New York--Newark, NY--NJ--CT | Bogota borough | 31.8% | 16.0% |
| New York--Newark, NY--NJ--CT | Bohemia CDP | 29.4% | 16.0% |
| New York--Newark, NY--NJ--CT | Boonton town | 27.8% | 16.0% |
| New York--Newark, NY--NJ--CT | Bound Brook borough | 20.0% | 16.0% |
| New York--Newark, NY--NJ--CT | Bradley Beach borough | 2.6% | 16.0% |
| New York--Newark, NY--NJ--CT | Bradley Gardens CDP | 34.8% | 16.0% |
| New York--Newark, NY--NJ--CT | Brentwood CDP | 28.9% | 16.0% |
| New York--Newark, NY--NJ--CT | Brewster Hill CDP | 26.3% | 16.0% |
| New York--Newark, NY--NJ--CT | Brewster village | 18.0% | 16.0% |
| New York--Newark, NY--NJ--CT | Briarcliff Manor village | 59.3% | 16.0% |
| New York--Newark, NY--NJ--CT | Brielle borough | 53.4% | 16.0% |
| New York--Newark, NY--NJ--CT | Brightwaters village | 42.7% | 16.0% |
| New York--Newark, NY--NJ--CT | Bronxville village | 50.6% | 16.0% |
| New York--Newark, NY--NJ--CT | Brookdale CDP | 48.2% | 16.0% |
| New York--Newark, NY--NJ--CT | Brookhaven CDP | 22.4% | 16.0% |
| New York--Newark, NY--NJ--CT | Brookville village | 69.4% | 16.0% |
| New York--Newark, NY--NJ--CT | Brownville CDP | 53.5% | 16.0% |
| New York--Newark, NY--NJ--CT | Buchanan village | 37.8% | 16.0% |
| New York--Newark, NY--NJ--CT | Budd Lake CDP | 60.9% | 16.0% |
| New York--Newark, NY--NJ--CT | Butler borough | 37.4% | 16.0% |
| New York--Newark, NY--NJ--CT | Byram Center CDP | 82.7% | 16.0% |
| New York--Newark, NY--NJ--CT | Caldwell borough | 41.8% | 16.0% |
| New York--Newark, NY--NJ--CT | Calverton CDP | 23.8% | 16.0% |
| New York--Newark, NY--NJ--CT | Carle Place CDP | 17.2% | 16.0% |
| New York--Newark, NY--NJ--CT | Carlstadt borough | 17.3% | 16.0% |
| New York--Newark, NY--NJ--CT | Carmel Hamlet CDP | 59.9% | 16.0% |
| New York--Newark, NY--NJ--CT | Carteret borough | 14.6% | 16.0% |
| New York--Newark, NY--NJ--CT | Cedar Glen Lakes CDP | 45.8% | 16.0% |
| New York--Newark, NY--NJ--CT | Cedar Glen West CDP | 51.0% | 16.0% |
| New York--Newark, NY--NJ--CT | Cedarhurst village | 14.1% | 16.0% |
| New York--Newark, NY--NJ--CT | Center Moriches CDP | 22.3% | 16.0% |
| New York--Newark, NY--NJ--CT | Centereach CDP | 27.4% | 16.0% |
| New York--Newark, NY--NJ--CT | Centerport CDP | 47.5% | 16.0% |
| New York--Newark, NY--NJ--CT | Central Islip CDP | 25.9% | 16.0% |
| New York--Newark, NY--NJ--CT | Centre Island village | 53.2% | 16.0% |
| New York--Newark, NY--NJ--CT | Chappaqua CDP | 42.8% | 16.0% |
| New York--Newark, NY--NJ--CT | Chatham borough | 50.4% | 16.0% |
| New York--Newark, NY--NJ--CT | Chester borough | 55.3% | 16.0% |
| New York--Newark, NY--NJ--CT | Chestnut Ridge village | 69.3% | 16.0% |
| New York--Newark, NY--NJ--CT | Clearbrook Park CDP | 15.8% | 16.0% |
| New York--Newark, NY--NJ--CT | Cliffside Park borough | 7.3% | 16.0% |
| New York--Newark, NY--NJ--CT | Cliffwood Beach CDP | 46.6% | 16.0% |
| New York--Newark, NY--NJ--CT | Clifton city | 24.3% | 16.0% |
| New York--Newark, NY--NJ--CT | Clinton town | 50.1% | 16.0% |
| New York--Newark, NY--NJ--CT | Closter borough | 57.9% | 16.0% |
| New York--Newark, NY--NJ--CT | Clyde CDP | 65.1% | 16.0% |
| New York--Newark, NY--NJ--CT | Cold Spring Harbor CDP | 62.7% | 16.0% |
| New York--Newark, NY--NJ--CT | Colonia CDP | 25.9% | 16.0% |
| New York--Newark, NY--NJ--CT | Commack CDP | 34.7% | 16.0% |
| New York--Newark, NY--NJ--CT | Concordia CDP | 20.4% | 16.0% |
| New York--Newark, NY--NJ--CT | Congers CDP | 34.5% | 16.0% |
| New York--Newark, NY--NJ--CT | Copiague CDP | 12.9% | 16.0% |
| New York--Newark, NY--NJ--CT | Coram CDP | 30.5% | 16.0% |
| New York--Newark, NY--NJ--CT | Cove Neck village | 69.9% | 16.0% |
| New York--Newark, NY--NJ--CT | Cresskill borough | 43.9% | 16.0% |
| New York--Newark, NY--NJ--CT | Crestwood Village CDP | 31.1% | 16.0% |
| New York--Newark, NY--NJ--CT | Crompond CDP | 56.2% | 16.0% |
| New York--Newark, NY--NJ--CT | Croton-on-Hudson village | 31.7% | 16.0% |
| New York--Newark, NY--NJ--CT | Crugers CDP | 57.3% | 16.0% |
| New York--Newark, NY--NJ--CT | Cutchogue CDP | 44.1% | 16.0% |
| New York--Newark, NY--NJ--CT | Dayton CDP | 29.8% | 16.0% |
| New York--Newark, NY--NJ--CT | Deal borough | 33.0% | 16.0% |
| New York--Newark, NY--NJ--CT | Deer Park CDP | 21.5% | 16.0% |
| New York--Newark, NY--NJ--CT | Demarest borough | 60.2% | 16.0% |
| New York--Newark, NY--NJ--CT | Dix Hills CDP | 53.2% | 16.0% |
| New York--Newark, NY--NJ--CT | Dobbs Ferry village | 39.1% | 16.0% |
| New York--Newark, NY--NJ--CT | Dover Beaches North CDP | 0.3% | 16.0% |
| New York--Newark, NY--NJ--CT | Dover Beaches South CDP | 0.3% | 16.0% |
| New York--Newark, NY--NJ--CT | Dover town | 21.6% | 16.0% |
| New York--Newark, NY--NJ--CT | Dumont borough | 30.7% | 16.0% |
| New York--Newark, NY--NJ--CT | Dunellen borough | 30.1% | 16.0% |
| New York--Newark, NY--NJ--CT | East Atlantic Beach CDP | 1.2% | 16.0% |
| New York--Newark, NY--NJ--CT | East Farmingdale CDP | 13.7% | 16.0% |
| New York--Newark, NY--NJ--CT | East Franklin CDP | 19.9% | 16.0% |
| New York--Newark, NY--NJ--CT | East Freehold CDP | 60.6% | 16.0% |
| New York--Newark, NY--NJ--CT | East Hills village | 48.8% | 16.0% |
| New York--Newark, NY--NJ--CT | East Islip CDP | 23.9% | 16.0% |
| New York--Newark, NY--NJ--CT | East Massapequa CDP | 5.9% | 16.0% |
| New York--Newark, NY--NJ--CT | East Meadow CDP | 11.2% | 16.0% |
| New York--Newark, NY--NJ--CT | East Millstone CDP | 51.4% | 16.0% |
| New York--Newark, NY--NJ--CT | East Moriches CDP | 26.6% | 16.0% |
| New York--Newark, NY--NJ--CT | East Newark borough | 7.9% | 16.0% |
| New York--Newark, NY--NJ--CT | East Northport CDP | 44.1% | 16.0% |
| New York--Newark, NY--NJ--CT | East Norwich CDP | 45.6% | 16.0% |
| New York--Newark, NY--NJ--CT | East Orange city | 27.8% | 16.0% |
| New York--Newark, NY--NJ--CT | East Patchogue CDP | 25.5% | 16.0% |
| New York--Newark, NY--NJ--CT | East Quogue CDP | 13.8% | 16.0% |
| New York--Newark, NY--NJ--CT | East Rockaway village | 18.8% | 16.0% |
| New York--Newark, NY--NJ--CT | East Rocky Hill CDP | 68.2% | 16.0% |
| New York--Newark, NY--NJ--CT | East Rutherford borough | 19.7% | 16.0% |
| New York--Newark, NY--NJ--CT | East Shoreham CDP | 24.8% | 16.0% |
| New York--Newark, NY--NJ--CT | East Williston village | 40.3% | 16.0% |
| New York--Newark, NY--NJ--CT | Eastchester CDP | 24.2% | 16.0% |
| New York--Newark, NY--NJ--CT | Eastport CDP | 27.8% | 16.0% |
| New York--Newark, NY--NJ--CT | Eatons Neck CDP | 63.2% | 16.0% |
| New York--Newark, NY--NJ--CT | Eatontown borough | 45.5% | 16.0% |
| New York--Newark, NY--NJ--CT | Edgewater borough | 9.0% | 16.0% |
| New York--Newark, NY--NJ--CT | Elizabeth city | 12.7% | 16.0% |
| New York--Newark, NY--NJ--CT | Elmont CDP | 12.4% | 16.0% |
| New York--Newark, NY--NJ--CT | Elmsford village | 17.7% | 16.0% |
| New York--Newark, NY--NJ--CT | Elmwood Park borough | 24.2% | 16.0% |
| New York--Newark, NY--NJ--CT | Elwood CDP | 47.5% | 16.0% |
| New York--Newark, NY--NJ--CT | Emerson borough | 36.3% | 16.0% |
| New York--Newark, NY--NJ--CT | Englewood Cliffs borough | 33.7% | 16.0% |
| New York--Newark, NY--NJ--CT | Englewood city | 35.8% | 16.0% |
| New York--Newark, NY--NJ--CT | Englishtown borough | 35.2% | 16.0% |
| New York--Newark, NY--NJ--CT | Essex Fells borough | 75.4% | 16.0% |
| New York--Newark, NY--NJ--CT | Fair Haven borough | 55.7% | 16.0% |
| New York--Newark, NY--NJ--CT | Fair Lawn borough | 35.7% | 16.0% |
| New York--Newark, NY--NJ--CT | Fairview CDP | 39.3% | 16.0% |
| New York--Newark, NY--NJ--CT | Fairview borough | 4.1% | 16.0% |
| New York--Newark, NY--NJ--CT | Fanwood borough | 43.3% | 16.0% |
| New York--Newark, NY--NJ--CT | Far Hills borough | 19.1% | 16.0% |
| New York--Newark, NY--NJ--CT | Farmingdale borough | 48.5% | 16.0% |
| New York--Newark, NY--NJ--CT | Farmingdale village | 12.5% | 16.0% |
| New York--Newark, NY--NJ--CT | Farmingville CDP | 30.1% | 16.0% |
| New York--Newark, NY--NJ--CT | Finderne CDP | 36.0% | 16.0% |
| New York--Newark, NY--NJ--CT | Flanders CDP | 19.1% | 16.0% |
| New York--Newark, NY--NJ--CT | Flemington borough | 28.7% | 16.0% |
| New York--Newark, NY--NJ--CT | Floral Park village | 21.8% | 16.0% |
| New York--Newark, NY--NJ--CT | Florham Park borough | 54.4% | 16.0% |
| New York--Newark, NY--NJ--CT | Flower Hill village | 59.2% | 16.0% |
| New York--Newark, NY--NJ--CT | Fords CDP | 17.1% | 16.0% |
| New York--Newark, NY--NJ--CT | Forked River CDP | 14.0% | 16.0% |
| New York--Newark, NY--NJ--CT | Fort Lee borough | 22.1% | 16.0% |
| New York--Newark, NY--NJ--CT | Fort Salonga CDP | 62.4% | 16.0% |
| New York--Newark, NY--NJ--CT | Franklin Center CDP | 38.0% | 16.0% |
| New York--Newark, NY--NJ--CT | Franklin Lakes borough | 65.0% | 16.0% |
| New York--Newark, NY--NJ--CT | Franklin Park CDP | 35.2% | 16.0% |
| New York--Newark, NY--NJ--CT | Franklin Square CDP | 13.8% | 16.0% |
| New York--Newark, NY--NJ--CT | Freehold borough | 39.9% | 16.0% |
| New York--Newark, NY--NJ--CT | Freeport village | 13.1% | 16.0% |
| New York--Newark, NY--NJ--CT | Garden City Park CDP | 17.6% | 16.0% |
| New York--Newark, NY--NJ--CT | Garden City South CDP | 15.3% | 16.0% |
| New York--Newark, NY--NJ--CT | Garden City village | 39.6% | 16.0% |
| New York--Newark, NY--NJ--CT | Garfield city | 13.1% | 16.0% |
| New York--Newark, NY--NJ--CT | Garwood borough | 13.3% | 16.0% |
| New York--Newark, NY--NJ--CT | Glen Cove city | 27.5% | 16.0% |
| New York--Newark, NY--NJ--CT | Glen Head CDP | 37.2% | 16.0% |
| New York--Newark, NY--NJ--CT | Glen Ridge borough | 58.7% | 16.0% |
| New York--Newark, NY--NJ--CT | Glen Rock borough | 61.6% | 16.0% |
| New York--Newark, NY--NJ--CT | Glenwood Landing CDP | 38.6% | 16.0% |
| New York--Newark, NY--NJ--CT | Golden's Bridge CDP | 73.2% | 16.0% |
| New York--Newark, NY--NJ--CT | Gordon Heights CDP | 32.7% | 16.0% |
| New York--Newark, NY--NJ--CT | Grand View-on-Hudson village | 69.9% | 16.0% |
| New York--Newark, NY--NJ--CT | Great Neck Estates village | 58.2% | 16.0% |
| New York--Newark, NY--NJ--CT | Great Neck Gardens CDP | 49.4% | 16.0% |
| New York--Newark, NY--NJ--CT | Great Neck Plaza village | 12.9% | 16.0% |
| New York--Newark, NY--NJ--CT | Great Neck village | 42.4% | 16.0% |
| New York--Newark, NY--NJ--CT | Great River CDP | 35.4% | 16.0% |
| New York--Newark, NY--NJ--CT | Green Knoll CDP | 59.8% | 16.0% |
| New York--Newark, NY--NJ--CT | Greenlawn CDP | 31.5% | 16.0% |
| New York--Newark, NY--NJ--CT | Greenvale CDP | 19.2% | 16.0% |
| New York--Newark, NY--NJ--CT | Greenville CDP | 37.2% | 16.0% |
| New York--Newark, NY--NJ--CT | Griggstown CDP | 72.1% | 16.0% |
| New York--Newark, NY--NJ--CT | Guttenberg town | 3.4% | 16.0% |
| New York--Newark, NY--NJ--CT | Hackensack city | 20.0% | 16.0% |
| New York--Newark, NY--NJ--CT | Hackettstown town | 35.4% | 16.0% |
| New York--Newark, NY--NJ--CT | Haledon borough | 20.3% | 16.0% |
| New York--Newark, NY--NJ--CT | Halesite CDP | 54.1% | 16.0% |
| New York--Newark, NY--NJ--CT | Hampton Bays CDP | 11.1% | 16.0% |
| New York--Newark, NY--NJ--CT | Harbor Hills CDP | 61.3% | 16.0% |
| New York--Newark, NY--NJ--CT | Harbor Isle CDP | 3.4% | 16.0% |
| New York--Newark, NY--NJ--CT | Harlingen CDP | 62.7% | 16.0% |
| New York--Newark, NY--NJ--CT | Harrington Park borough | 59.2% | 16.0% |
| New York--Newark, NY--NJ--CT | Harrison town | 9.4% | 16.0% |
| New York--Newark, NY--NJ--CT | Harrison village | 30.4% | 16.0% |
| New York--Newark, NY--NJ--CT | Hartsdale CDP | 41.1% | 16.0% |
| New York--Newark, NY--NJ--CT | Harvey Cedars borough | 11.9% | 16.0% |
| New York--Newark, NY--NJ--CT | Hasbrouck Heights borough | 24.7% | 16.0% |
| New York--Newark, NY--NJ--CT | Hastings-on-Hudson village | 56.2% | 16.0% |
| New York--Newark, NY--NJ--CT | Hauppauge CDP | 35.8% | 16.0% |
| New York--Newark, NY--NJ--CT | Haverstraw village | 10.6% | 16.0% |
| New York--Newark, NY--NJ--CT | Haworth borough | 70.0% | 16.0% |
| New York--Newark, NY--NJ--CT | Hawthorne CDP | 21.6% | 16.0% |
| New York--Newark, NY--NJ--CT | Hawthorne borough | 36.2% | 16.0% |
| New York--Newark, NY--NJ--CT | Head of the Harbor village | 58.4% | 16.0% |
| New York--Newark, NY--NJ--CT | Heathcote CDP | 62.0% | 16.0% |
| New York--Newark, NY--NJ--CT | Helmetta borough | 44.0% | 16.0% |
| New York--Newark, NY--NJ--CT | Hempstead village | 15.3% | 16.0% |
| New York--Newark, NY--NJ--CT | Heritage Hills CDP | 50.3% | 16.0% |
| New York--Newark, NY--NJ--CT | Herricks CDP | 22.8% | 16.0% |
| New York--Newark, NY--NJ--CT | Hewlett Bay Park village | 61.1% | 16.0% |
| New York--Newark, NY--NJ--CT | Hewlett CDP | 26.4% | 16.0% |
| New York--Newark, NY--NJ--CT | Hewlett Harbor village | 53.3% | 16.0% |
| New York--Newark, NY--NJ--CT | Hewlett Neck village | 57.7% | 16.0% |
| New York--Newark, NY--NJ--CT | Hicksville CDP | 18.4% | 16.0% |
| New York--Newark, NY--NJ--CT | High Bridge borough | 69.6% | 16.0% |
| New York--Newark, NY--NJ--CT | Highland Park borough | 23.4% | 16.0% |
| New York--Newark, NY--NJ--CT | Highlands borough | 16.3% | 16.0% |
| New York--Newark, NY--NJ--CT | Hillburn village | 47.4% | 16.0% |
| New York--Newark, NY--NJ--CT | Hillcrest CDP | 41.0% | 16.0% |
| New York--Newark, NY--NJ--CT | Hillsdale borough | 55.7% | 16.0% |
| New York--Newark, NY--NJ--CT | Ho-Ho-Kus borough | 45.6% | 16.0% |
| New York--Newark, NY--NJ--CT | Hoboken city | 7.2% | 16.0% |
| New York--Newark, NY--NJ--CT | Holbrook CDP | 21.9% | 16.0% |
| New York--Newark, NY--NJ--CT | Holiday City South CDP | 12.1% | 16.0% |
| New York--Newark, NY--NJ--CT | Holiday City-Berkeley CDP | 11.5% | 16.0% |
| New York--Newark, NY--NJ--CT | Holiday Heights CDP | 8.0% | 16.0% |
| New York--Newark, NY--NJ--CT | Holtsville CDP | 29.7% | 16.0% |
| New York--Newark, NY--NJ--CT | Hopatcong borough | 65.6% | 16.0% |
| New York--Newark, NY--NJ--CT | Hopewell borough | 43.2% | 16.0% |
| New York--Newark, NY--NJ--CT | Huntington Bay village | 45.7% | 16.0% |
| New York--Newark, NY--NJ--CT | Huntington CDP | 51.6% | 16.0% |
| New York--Newark, NY--NJ--CT | Huntington Station CDP | 38.2% | 16.0% |
| New York--Newark, NY--NJ--CT | Interlaken borough | 58.6% | 16.0% |
| New York--Newark, NY--NJ--CT | Inwood CDP | 10.9% | 16.0% |
| New York--Newark, NY--NJ--CT | Irvington village | 53.1% | 16.0% |
| New York--Newark, NY--NJ--CT | Iselin CDP | 17.2% | 16.0% |
| New York--Newark, NY--NJ--CT | Island Heights borough | 38.3% | 16.0% |
| New York--Newark, NY--NJ--CT | Island Park village | 1.5% | 16.0% |
| New York--Newark, NY--NJ--CT | Islandia village | 29.8% | 16.0% |
| New York--Newark, NY--NJ--CT | Islip CDP | 19.8% | 16.0% |
| New York--Newark, NY--NJ--CT | Islip Terrace CDP | 26.1% | 16.0% |
| New York--Newark, NY--NJ--CT | Jamesburg borough | 26.8% | 16.0% |
| New York--Newark, NY--NJ--CT | Jamesport CDP | 31.5% | 16.0% |
| New York--Newark, NY--NJ--CT | Jefferson Valley-Yorktown CDP | 47.4% | 16.0% |
| New York--Newark, NY--NJ--CT | Jericho CDP | 27.4% | 16.0% |
| New York--Newark, NY--NJ--CT | Jersey City city | 11.8% | 16.0% |
| New York--Newark, NY--NJ--CT | Kaser village | 18.6% | 16.0% |
| New York--Newark, NY--NJ--CT | Katonah CDP | 37.5% | 16.0% |
| New York--Newark, NY--NJ--CT | Keansburg borough | 17.5% | 16.0% |
| New York--Newark, NY--NJ--CT | Kearny town | 11.8% | 16.0% |
| New York--Newark, NY--NJ--CT | Kendall Park CDP | 46.2% | 16.0% |
| New York--Newark, NY--NJ--CT | Kenilworth borough | 25.1% | 16.0% |
| New York--Newark, NY--NJ--CT | Kensington village | 48.5% | 16.0% |
| New York--Newark, NY--NJ--CT | Kenvil CDP | 41.0% | 16.0% |
| New York--Newark, NY--NJ--CT | Keyport borough | 27.0% | 16.0% |
| New York--Newark, NY--NJ--CT | Kings Park CDP | 39.2% | 16.0% |
| New York--Newark, NY--NJ--CT | Kings Point village | 60.9% | 16.0% |
| New York--Newark, NY--NJ--CT | Kingston CDP | 47.2% | 16.0% |
| New York--Newark, NY--NJ--CT | Kinnelon borough | 79.7% | 16.0% |
| New York--Newark, NY--NJ--CT | Lake Carmel CDP | 40.4% | 16.0% |
| New York--Newark, NY--NJ--CT | Lake Como borough | 7.2% | 16.0% |
| New York--Newark, NY--NJ--CT | Lake Grove village | 27.7% | 16.0% |
| New York--Newark, NY--NJ--CT | Lake Mohawk CDP | 70.8% | 16.0% |
| New York--Newark, NY--NJ--CT | Lake Mohegan CDP | 47.2% | 16.0% |
| New York--Newark, NY--NJ--CT | Lake Ronkonkoma CDP | 23.0% | 16.0% |
| New York--Newark, NY--NJ--CT | Lake Success village | 51.4% | 16.0% |
| New York--Newark, NY--NJ--CT | Lake Telemark CDP | 61.8% | 16.0% |
| New York--Newark, NY--NJ--CT | Lakehurst borough | 13.5% | 16.0% |
| New York--Newark, NY--NJ--CT | Lakeview CDP | 23.5% | 16.0% |
| New York--Newark, NY--NJ--CT | Lakewood CDP | 46.2% | 16.0% |
| New York--Newark, NY--NJ--CT | Larchmont village | 39.5% | 16.0% |
| New York--Newark, NY--NJ--CT | Lattingtown village | 62.2% | 16.0% |
| New York--Newark, NY--NJ--CT | Laurel CDP | 16.6% | 16.0% |
| New York--Newark, NY--NJ--CT | Laurel Hollow village | 57.6% | 16.0% |
| New York--Newark, NY--NJ--CT | Laurence Harbor CDP | 34.5% | 16.0% |
| New York--Newark, NY--NJ--CT | Lavallette borough | 1.0% | 16.0% |
| New York--Newark, NY--NJ--CT | Lawrence village | 33.3% | 16.0% |
| New York--Newark, NY--NJ--CT | Lebanon borough | 52.9% | 16.0% |
| New York--Newark, NY--NJ--CT | Leisure Knoll CDP | 7.6% | 16.0% |
| New York--Newark, NY--NJ--CT | Leisure Village CDP | 63.6% | 16.0% |
| New York--Newark, NY--NJ--CT | Leisure Village East CDP | 45.8% | 16.0% |
| New York--Newark, NY--NJ--CT | Leisure Village West CDP | 28.2% | 16.0% |
| New York--Newark, NY--NJ--CT | Leonardo CDP | 35.2% | 16.0% |
| New York--Newark, NY--NJ--CT | Leonia borough | 49.8% | 16.0% |
| New York--Newark, NY--NJ--CT | Levittown CDP | 13.9% | 16.0% |
| New York--Newark, NY--NJ--CT | Lido Beach CDP | 6.8% | 16.0% |
| New York--Newark, NY--NJ--CT | Lincoln Park borough | 59.9% | 16.0% |
| New York--Newark, NY--NJ--CT | Lincolndale CDP | 58.6% | 16.0% |
| New York--Newark, NY--NJ--CT | Lincroft CDP | 70.2% | 16.0% |
| New York--Newark, NY--NJ--CT | Linden city | 20.0% | 16.0% |
| New York--Newark, NY--NJ--CT | Lindenhurst village | 21.2% | 16.0% |
| New York--Newark, NY--NJ--CT | Little Ferry borough | 22.2% | 16.0% |
| New York--Newark, NY--NJ--CT | Little Silver borough | 56.5% | 16.0% |
| New York--Newark, NY--NJ--CT | Lloyd Harbor village | 63.9% | 16.0% |
| New York--Newark, NY--NJ--CT | Loch Arbour village | 21.0% | 16.0% |
| New York--Newark, NY--NJ--CT | Locust Valley CDP | 25.1% | 16.0% |
| New York--Newark, NY--NJ--CT | Lodi borough | 14.7% | 16.0% |
| New York--Newark, NY--NJ--CT | Long Beach city | 0.3% | 16.0% |
| New York--Newark, NY--NJ--CT | Long Branch city | 25.6% | 16.0% |
| New York--Newark, NY--NJ--CT | Long Valley CDP | 69.5% | 16.0% |
| New York--Newark, NY--NJ--CT | Lynbrook village | 18.4% | 16.0% |
| New York--Newark, NY--NJ--CT | Madison Park CDP | 23.3% | 16.0% |
| New York--Newark, NY--NJ--CT | Madison borough | 49.8% | 16.0% |
| New York--Newark, NY--NJ--CT | Mahopac CDP | 59.3% | 16.0% |
| New York--Newark, NY--NJ--CT | Malverne Park Oaks CDP | 34.7% | 16.0% |
| New York--Newark, NY--NJ--CT | Malverne village | 24.0% | 16.0% |
| New York--Newark, NY--NJ--CT | Mamaroneck village | 22.9% | 16.0% |
| New York--Newark, NY--NJ--CT | Manahawkin CDP | 35.2% | 16.0% |
| New York--Newark, NY--NJ--CT | Manasquan borough | 32.8% | 16.0% |
| New York--Newark, NY--NJ--CT | Manhasset CDP | 41.8% | 16.0% |
| New York--Newark, NY--NJ--CT | Manhasset Hills CDP | 35.8% | 16.0% |
| New York--Newark, NY--NJ--CT | Manorhaven village | 15.8% | 16.0% |
| New York--Newark, NY--NJ--CT | Manorville CDP | 26.6% | 16.0% |
| New York--Newark, NY--NJ--CT | Mantoloking borough | 3.2% | 16.0% |
| New York--Newark, NY--NJ--CT | Manville borough | 18.1% | 16.0% |
| New York--Newark, NY--NJ--CT | Martinsville CDP | 74.7% | 16.0% |
| New York--Newark, NY--NJ--CT | Massapequa CDP | 7.0% | 16.0% |
| New York--Newark, NY--NJ--CT | Massapequa Park village | 6.0% | 16.0% |
| New York--Newark, NY--NJ--CT | Mastic Beach CDP | 14.4% | 16.0% |
| New York--Newark, NY--NJ--CT | Mastic CDP | 13.2% | 16.0% |
| New York--Newark, NY--NJ--CT | Matawan borough | 53.1% | 16.0% |
| New York--Newark, NY--NJ--CT | Matinecock village | 64.6% | 16.0% |
| New York--Newark, NY--NJ--CT | Mattituck CDP | 27.4% | 16.0% |
| New York--Newark, NY--NJ--CT | Maywood borough | 36.2% | 16.0% |
| New York--Newark, NY--NJ--CT | Medford CDP | 26.3% | 16.0% |
| New York--Newark, NY--NJ--CT | Melville CDP | 36.1% | 16.0% |
| New York--Newark, NY--NJ--CT | Mendham borough | 59.7% | 16.0% |
| New York--Newark, NY--NJ--CT | Merrick CDP | 6.0% | 16.0% |
| New York--Newark, NY--NJ--CT | Metuchen borough | 26.8% | 16.0% |
| New York--Newark, NY--NJ--CT | Middle Island CDP | 27.7% | 16.0% |
| New York--Newark, NY--NJ--CT | Middlebush CDP | 56.5% | 16.0% |
| New York--Newark, NY--NJ--CT | Middlesex borough | 23.7% | 16.0% |
| New York--Newark, NY--NJ--CT | Midland Park borough | 47.3% | 16.0% |
| New York--Newark, NY--NJ--CT | Mill Neck village | 61.6% | 16.0% |
| New York--Newark, NY--NJ--CT | Miller Place CDP | 29.5% | 16.0% |
| New York--Newark, NY--NJ--CT | Millstone borough | 63.3% | 16.0% |
| New York--Newark, NY--NJ--CT | Milltown borough | 26.8% | 16.0% |
| New York--Newark, NY--NJ--CT | Mineola village | 10.3% | 16.0% |
| New York--Newark, NY--NJ--CT | Monmouth Beach borough | 25.5% | 16.0% |
| New York--Newark, NY--NJ--CT | Monmouth Junction CDP | 45.2% | 16.0% |
| New York--Newark, NY--NJ--CT | Monsey CDP | 37.1% | 16.0% |
| New York--Newark, NY--NJ--CT | Montebello village | 50.9% | 16.0% |
| New York--Newark, NY--NJ--CT | Montrose CDP | 54.0% | 16.0% |
| New York--Newark, NY--NJ--CT | Montvale borough | 58.1% | 16.0% |
| New York--Newark, NY--NJ--CT | Moonachie borough | 18.6% | 16.0% |
| New York--Newark, NY--NJ--CT | Morganville CDP | 76.0% | 16.0% |
| New York--Newark, NY--NJ--CT | Moriches CDP | 18.6% | 16.0% |
| New York--Newark, NY--NJ--CT | Morris Plains borough | 53.8% | 16.0% |
| New York--Newark, NY--NJ--CT | Morristown town | 28.7% | 16.0% |
| New York--Newark, NY--NJ--CT | Mount Arlington borough | 59.9% | 16.0% |
| New York--Newark, NY--NJ--CT | Mount Ivy CDP | 25.5% | 16.0% |
| New York--Newark, NY--NJ--CT | Mount Kisco village | 30.2% | 16.0% |
| New York--Newark, NY--NJ--CT | Mount Sinai CDP | 37.7% | 16.0% |
| New York--Newark, NY--NJ--CT | Mount Vernon city | 18.6% | 16.0% |
| New York--Newark, NY--NJ--CT | Mountain Lakes borough | 73.3% | 16.0% |
| New York--Newark, NY--NJ--CT | Mountainside borough | 46.2% | 16.0% |
| New York--Newark, NY--NJ--CT | Munsey Park village | 60.2% | 16.0% |
| New York--Newark, NY--NJ--CT | Muttontown village | 75.1% | 16.0% |
| New York--Newark, NY--NJ--CT | Nanuet CDP | 44.8% | 16.0% |
| New York--Newark, NY--NJ--CT | Navesink CDP | 56.2% | 16.0% |
| New York--Newark, NY--NJ--CT | Neptune City borough | 19.2% | 16.0% |
| New York--Newark, NY--NJ--CT | Nesconset CDP | 31.2% | 16.0% |
| New York--Newark, NY--NJ--CT | Netcong borough | 37.4% | 16.0% |
| New York--Newark, NY--NJ--CT | New Brunswick city | 8.5% | 16.0% |
| New York--Newark, NY--NJ--CT | New Cassel CDP | 13.6% | 16.0% |
| New York--Newark, NY--NJ--CT | New City CDP | 50.5% | 16.0% |
| New York--Newark, NY--NJ--CT | New Hempstead village | 50.8% | 16.0% |
| New York--Newark, NY--NJ--CT | New Hyde Park village | 14.3% | 16.0% |
| New York--Newark, NY--NJ--CT | New Milford borough | 38.5% | 16.0% |
| New York--Newark, NY--NJ--CT | New Providence borough | 54.4% | 16.0% |
| New York--Newark, NY--NJ--CT | New Rochelle city | 22.5% | 16.0% |
| New York--Newark, NY--NJ--CT | New Square village | 15.6% | 16.0% |
| New York--Newark, NY--NJ--CT | New Suffolk CDP | 39.3% | 16.0% |
| New York--Newark, NY--NJ--CT | New York city | 8.0% | 16.0% |
| New York--Newark, NY--NJ--CT | Newark city | 16.2% | 16.0% |
| New York--Newark, NY--NJ--CT | Nissequogue village | 56.0% | 16.0% |
| New York--Newark, NY--NJ--CT | North Amityville CDP | 26.8% | 16.0% |
| New York--Newark, NY--NJ--CT | North Arlington borough | 13.6% | 16.0% |
| New York--Newark, NY--NJ--CT | North Babylon CDP | 22.6% | 16.0% |
| New York--Newark, NY--NJ--CT | North Bay Shore CDP | 32.2% | 16.0% |
| New York--Newark, NY--NJ--CT | North Beach Haven CDP | 1.7% | 16.0% |
| New York--Newark, NY--NJ--CT | North Bellmore CDP | 13.3% | 16.0% |
| New York--Newark, NY--NJ--CT | North Bellport CDP | 28.4% | 16.0% |
| New York--Newark, NY--NJ--CT | North Caldwell borough | 62.3% | 16.0% |
| New York--Newark, NY--NJ--CT | North Great River CDP | 35.9% | 16.0% |
| New York--Newark, NY--NJ--CT | North Haledon borough | 61.8% | 16.0% |
| New York--Newark, NY--NJ--CT | North Haven village | 33.4% | 16.0% |
| New York--Newark, NY--NJ--CT | North Hills village | 55.9% | 16.0% |
| New York--Newark, NY--NJ--CT | North Lindenhurst CDP | 19.6% | 16.0% |
| New York--Newark, NY--NJ--CT | North Lynbrook CDP | 29.2% | 16.0% |
| New York--Newark, NY--NJ--CT | North Massapequa CDP | 9.5% | 16.0% |
| New York--Newark, NY--NJ--CT | North Merrick CDP | 14.8% | 16.0% |
| New York--Newark, NY--NJ--CT | North Middletown CDP | 32.9% | 16.0% |
| New York--Newark, NY--NJ--CT | North New Hyde Park CDP | 19.5% | 16.0% |
| New York--Newark, NY--NJ--CT | North Patchogue CDP | 18.9% | 16.0% |
| New York--Newark, NY--NJ--CT | North Plainfield borough | 32.0% | 16.0% |
| New York--Newark, NY--NJ--CT | North Sea CDP | 11.2% | 16.0% |
| New York--Newark, NY--NJ--CT | North Valley Stream CDP | 17.0% | 16.0% |
| New York--Newark, NY--NJ--CT | North Wantagh CDP | 12.3% | 16.0% |
| New York--Newark, NY--NJ--CT | Northampton CDP | 27.7% | 16.0% |
| New York--Newark, NY--NJ--CT | Northport village | 49.5% | 16.0% |
| New York--Newark, NY--NJ--CT | Northvale borough | 40.9% | 16.0% |
| New York--Newark, NY--NJ--CT | Northville CDP | 34.6% | 16.0% |
| New York--Newark, NY--NJ--CT | Norwood borough | 58.8% | 16.0% |
| New York--Newark, NY--NJ--CT | Noyack CDP | 12.5% | 16.0% |
| New York--Newark, NY--NJ--CT | Nyack village | 11.6% | 16.0% |
| New York--Newark, NY--NJ--CT | Oakdale CDP | 22.8% | 16.0% |
| New York--Newark, NY--NJ--CT | Oakhurst CDP | 45.0% | 16.0% |
| New York--Newark, NY--NJ--CT | Oakland borough | 61.1% | 16.0% |
| New York--Newark, NY--NJ--CT | Ocean Acres CDP | 25.9% | 16.0% |
| New York--Newark, NY--NJ--CT | Ocean Gate borough | 21.0% | 16.0% |
| New York--Newark, NY--NJ--CT | Ocean Grove CDP | 0.5% | 16.0% |
| New York--Newark, NY--NJ--CT | Oceanport borough | 34.6% | 16.0% |
| New York--Newark, NY--NJ--CT | Oceanside CDP | 12.1% | 16.0% |
| New York--Newark, NY--NJ--CT | Old Bethpage CDP | 23.1% | 16.0% |
| New York--Newark, NY--NJ--CT | Old Bridge CDP | 31.6% | 16.0% |
| New York--Newark, NY--NJ--CT | Old Brookville village | 55.6% | 16.0% |
| New York--Newark, NY--NJ--CT | Old Field village | 50.6% | 16.0% |
| New York--Newark, NY--NJ--CT | Old Tappan borough | 60.8% | 16.0% |
| New York--Newark, NY--NJ--CT | Old Westbury village | 58.2% | 16.0% |
| New York--Newark, NY--NJ--CT | Oradell borough | 52.9% | 16.0% |
| New York--Newark, NY--NJ--CT | Orangeburg CDP | 42.8% | 16.0% |
| New York--Newark, NY--NJ--CT | Ossining village | 15.7% | 16.0% |
| New York--Newark, NY--NJ--CT | Oyster Bay CDP | 32.8% | 16.0% |
| New York--Newark, NY--NJ--CT | Oyster Bay Cove village | 74.7% | 16.0% |
| New York--Newark, NY--NJ--CT | Palisades Park borough | 12.8% | 16.0% |
| New York--Newark, NY--NJ--CT | Panther Valley CDP | 57.8% | 16.0% |
| New York--Newark, NY--NJ--CT | Paramus borough | 50.9% | 16.0% |
| New York--Newark, NY--NJ--CT | Park Ridge borough | 58.6% | 16.0% |
| New York--Newark, NY--NJ--CT | Passaic city | 11.6% | 16.0% |
| New York--Newark, NY--NJ--CT | Patchogue village | 16.9% | 16.0% |
| New York--Newark, NY--NJ--CT | Paterson city | 15.6% | 16.0% |
| New York--Newark, NY--NJ--CT | Peach Lake CDP | 48.2% | 16.0% |
| New York--Newark, NY--NJ--CT | Peapack and Gladstone borough | 59.3% | 16.0% |
| New York--Newark, NY--NJ--CT | Pearl River CDP | 49.7% | 16.0% |
| New York--Newark, NY--NJ--CT | Peekskill city | 26.3% | 16.0% |
| New York--Newark, NY--NJ--CT | Pelham Manor village | 53.7% | 16.0% |
| New York--Newark, NY--NJ--CT | Pelham village | 28.0% | 16.0% |
| New York--Newark, NY--NJ--CT | Perth Amboy city | 6.8% | 16.0% |
| New York--Newark, NY--NJ--CT | Piermont village | 55.1% | 16.0% |
| New York--Newark, NY--NJ--CT | Pine Beach borough | 24.8% | 16.0% |
| New York--Newark, NY--NJ--CT | Pine Lake Park CDP | 25.4% | 16.0% |
| New York--Newark, NY--NJ--CT | Pine Ridge at Crestwood CDP | 18.6% | 16.0% |
| New York--Newark, NY--NJ--CT | Plainedge CDP | 9.9% | 16.0% |
| New York--Newark, NY--NJ--CT | Plainfield city | 32.1% | 16.0% |
| New York--Newark, NY--NJ--CT | Plainsboro Center CDP | 36.1% | 16.0% |
| New York--Newark, NY--NJ--CT | Plainview CDP | 9.6% | 16.0% |
| New York--Newark, NY--NJ--CT | Plandome Heights village | 48.9% | 16.0% |
| New York--Newark, NY--NJ--CT | Plandome Manor village | 54.4% | 16.0% |
| New York--Newark, NY--NJ--CT | Plandome village | 57.1% | 16.0% |
| New York--Newark, NY--NJ--CT | Pleasant Plains CDP | 47.8% | 16.0% |
| New York--Newark, NY--NJ--CT | Pleasantville village | 29.8% | 16.0% |
| New York--Newark, NY--NJ--CT | Point Lookout CDP | 0.1% | 16.0% |
| New York--Newark, NY--NJ--CT | Point Pleasant Beach borough | 12.6% | 16.0% |
| New York--Newark, NY--NJ--CT | Point Pleasant borough | 28.1% | 16.0% |
| New York--Newark, NY--NJ--CT | Pomona village | 61.7% | 16.0% |
| New York--Newark, NY--NJ--CT | Pompton Lakes borough | 39.9% | 16.0% |
| New York--Newark, NY--NJ--CT | Poquott village | 40.9% | 16.0% |
| New York--Newark, NY--NJ--CT | Port Jefferson Station CDP | 37.0% | 16.0% |
| New York--Newark, NY--NJ--CT | Port Jefferson village | 39.0% | 16.0% |
| New York--Newark, NY--NJ--CT | Port Monmouth CDP | 49.0% | 16.0% |
| New York--Newark, NY--NJ--CT | Port Reading CDP | 19.0% | 16.0% |
| New York--Newark, NY--NJ--CT | Port Washington CDP | 40.7% | 16.0% |
| New York--Newark, NY--NJ--CT | Port Washington North village | 32.4% | 16.0% |
| New York--Newark, NY--NJ--CT | Princeton | 55.5% | 16.0% |
| New York--Newark, NY--NJ--CT | Princeton Meadows CDP | 34.1% | 16.0% |
| New York--Newark, NY--NJ--CT | Prospect Park borough | 17.1% | 16.0% |
| New York--Newark, NY--NJ--CT | Quiogue CDP | 16.3% | 16.0% |
| New York--Newark, NY--NJ--CT | Quogue village | 20.6% | 16.0% |
| New York--Newark, NY--NJ--CT | Rahway city | 30.1% | 16.0% |
| New York--Newark, NY--NJ--CT | Ramsey borough | 56.7% | 16.0% |
| New York--Newark, NY--NJ--CT | Ramtown CDP | 47.8% | 16.0% |
| New York--Newark, NY--NJ--CT | Raritan borough | 14.2% | 16.0% |
| New York--Newark, NY--NJ--CT | Red Bank borough | 34.8% | 16.0% |
| New York--Newark, NY--NJ--CT | Remsenburg-Speonk CDP | 22.5% | 16.0% |
| New York--Newark, NY--NJ--CT | Ridge CDP | 26.0% | 16.0% |
| New York--Newark, NY--NJ--CT | Ridgefield Park village | 33.4% | 16.0% |
| New York--Newark, NY--NJ--CT | Ridgefield borough | 34.9% | 16.0% |
| New York--Newark, NY--NJ--CT | Ridgewood village | 55.1% | 16.0% |
| New York--Newark, NY--NJ--CT | Ringwood borough | 78.5% | 16.0% |
| New York--Newark, NY--NJ--CT | River Edge borough | 49.3% | 16.0% |
| New York--Newark, NY--NJ--CT | Riverdale borough | 48.1% | 16.0% |
| New York--Newark, NY--NJ--CT | Riverhead CDP | 18.6% | 16.0% |
| New York--Newark, NY--NJ--CT | Riverside CDP | 31.2% | 16.0% |
| New York--Newark, NY--NJ--CT | Robertsville CDP | 61.4% | 16.0% |
| New York--Newark, NY--NJ--CT | Rockaway borough | 36.1% | 16.0% |
| New York--Newark, NY--NJ--CT | Rockleigh borough | 42.4% | 16.0% |
| New York--Newark, NY--NJ--CT | Rockville Centre village | 18.6% | 16.0% |
| New York--Newark, NY--NJ--CT | Rocky Hill borough | 67.1% | 16.0% |
| New York--Newark, NY--NJ--CT | Rocky Point CDP | 17.3% | 16.0% |
| New York--Newark, NY--NJ--CT | Ronkonkoma CDP | 27.3% | 16.0% |
| New York--Newark, NY--NJ--CT | Roosevelt CDP | 17.4% | 16.0% |
| New York--Newark, NY--NJ--CT | Roseland borough | 52.8% | 16.0% |
| New York--Newark, NY--NJ--CT | Roselle Park borough | 24.8% | 16.0% |
| New York--Newark, NY--NJ--CT | Roselle borough | 36.9% | 16.0% |
| New York--Newark, NY--NJ--CT | Roslyn Estates village | 70.2% | 16.0% |
| New York--Newark, NY--NJ--CT | Roslyn Harbor village | 65.5% | 16.0% |
| New York--Newark, NY--NJ--CT | Roslyn Heights CDP | 37.2% | 16.0% |
| New York--Newark, NY--NJ--CT | Roslyn village | 42.7% | 16.0% |
| New York--Newark, NY--NJ--CT | Rossmoor CDP | 16.0% | 16.0% |
| New York--Newark, NY--NJ--CT | Rumson borough | 53.7% | 16.0% |
| New York--Newark, NY--NJ--CT | Russell Gardens village | 60.7% | 16.0% |
| New York--Newark, NY--NJ--CT | Rutherford borough | 34.9% | 16.0% |
| New York--Newark, NY--NJ--CT | Rye city | 42.0% | 16.0% |
| New York--Newark, NY--NJ--CT | Saddle River borough | 63.8% | 16.0% |
| New York--Newark, NY--NJ--CT | Saddle Rock Estates CDP | 51.5% | 16.0% |
| New York--Newark, NY--NJ--CT | Saddle Rock village | 52.3% | 16.0% |
| New York--Newark, NY--NJ--CT | Sag Harbor village | 19.8% | 16.0% |
| New York--Newark, NY--NJ--CT | Salisbury CDP | 14.4% | 16.0% |
| New York--Newark, NY--NJ--CT | Sands Point village | 70.3% | 16.0% |
| New York--Newark, NY--NJ--CT | Sayreville borough | 18.6% | 16.0% |
| New York--Newark, NY--NJ--CT | Sayville CDP | 18.3% | 16.0% |
| New York--Newark, NY--NJ--CT | Scarsdale village | 34.3% | 16.0% |
| New York--Newark, NY--NJ--CT | Sea Bright borough | 4.4% | 16.0% |
| New York--Newark, NY--NJ--CT | Sea Cliff village | 44.7% | 16.0% |
| New York--Newark, NY--NJ--CT | Sea Girt borough | 32.9% | 16.0% |
| New York--Newark, NY--NJ--CT | Seaford CDP | 9.0% | 16.0% |
| New York--Newark, NY--NJ--CT | Searingtown CDP | 35.4% | 16.0% |
| New York--Newark, NY--NJ--CT | Seaside Heights borough | 0.3% | 16.0% |
| New York--Newark, NY--NJ--CT | Seaside Park borough | 0.3% | 16.0% |
| New York--Newark, NY--NJ--CT | Secaucus town | 16.5% | 16.0% |
| New York--Newark, NY--NJ--CT | Selden CDP | 23.2% | 16.0% |
| New York--Newark, NY--NJ--CT | Setauket-East Setauket CDP | 46.2% | 16.0% |
| New York--Newark, NY--NJ--CT | Sewaren CDP | 31.8% | 16.0% |
| New York--Newark, NY--NJ--CT | Shark River Hills CDP | 53.7% | 16.0% |
| New York--Newark, NY--NJ--CT | Shenorock CDP | 45.2% | 16.0% |
| New York--Newark, NY--NJ--CT | Shinnecock Hills CDP | 25.1% | 16.0% |
| New York--Newark, NY--NJ--CT | Ship Bottom borough | 4.9% | 16.0% |
| New York--Newark, NY--NJ--CT | Shirley CDP | 14.4% | 16.0% |
| New York--Newark, NY--NJ--CT | Shoreham village | 21.4% | 16.0% |
| New York--Newark, NY--NJ--CT | Short Hills CDP | 51.1% | 16.0% |
| New York--Newark, NY--NJ--CT | Shrewsbury borough | 61.5% | 16.0% |
| New York--Newark, NY--NJ--CT | Shrub Oak CDP | 58.4% | 16.0% |
| New York--Newark, NY--NJ--CT | Silver Lake CDP | 12.1% | 16.0% |
| New York--Newark, NY--NJ--CT | Silver Ridge CDP | 27.8% | 16.0% |
| New York--Newark, NY--NJ--CT | Singac CDP | 24.2% | 16.0% |
| New York--Newark, NY--NJ--CT | Six Mile Run CDP | 50.0% | 16.0% |
| New York--Newark, NY--NJ--CT | Skillman CDP | 55.3% | 16.0% |
| New York--Newark, NY--NJ--CT | Sleepy Hollow village | 5.5% | 16.0% |
| New York--Newark, NY--NJ--CT | Sloatsburg village | 62.4% | 16.0% |
| New York--Newark, NY--NJ--CT | Smithtown CDP | 50.5% | 16.0% |
| New York--Newark, NY--NJ--CT | Society Hill CDP | 38.9% | 16.0% |
| New York--Newark, NY--NJ--CT | Somerset CDP | 43.7% | 16.0% |
| New York--Newark, NY--NJ--CT | Somerville borough | 27.3% | 16.0% |
| New York--Newark, NY--NJ--CT | Sound Beach CDP | 18.8% | 16.0% |
| New York--Newark, NY--NJ--CT | South Amboy city | 11.5% | 16.0% |
| New York--Newark, NY--NJ--CT | South Bound Brook borough | 26.3% | 16.0% |
| New York--Newark, NY--NJ--CT | South Farmingdale CDP | 8.1% | 16.0% |
| New York--Newark, NY--NJ--CT | South Floral Park village | 14.4% | 16.0% |
| New York--Newark, NY--NJ--CT | South Hempstead CDP | 19.6% | 16.0% |
| New York--Newark, NY--NJ--CT | South Huntington CDP | 46.8% | 16.0% |
| New York--Newark, NY--NJ--CT | South Nyack village | 29.1% | 16.0% |
| New York--Newark, NY--NJ--CT | South Plainfield borough | 29.9% | 16.0% |
| New York--Newark, NY--NJ--CT | South River borough | 12.4% | 16.0% |
| New York--Newark, NY--NJ--CT | South Toms River borough | 17.9% | 16.0% |
| New York--Newark, NY--NJ--CT | South Valley Stream CDP | 27.5% | 16.0% |
| New York--Newark, NY--NJ--CT | Southampton village | 27.6% | 16.0% |
| New York--Newark, NY--NJ--CT | Sparkill CDP | 50.6% | 16.0% |
| New York--Newark, NY--NJ--CT | Spotswood borough | 37.2% | 16.0% |
| New York--Newark, NY--NJ--CT | Spring Lake Heights borough | 36.6% | 16.0% |
| New York--Newark, NY--NJ--CT | Spring Lake borough | 25.6% | 16.0% |
| New York--Newark, NY--NJ--CT | Spring Valley village | 28.9% | 16.0% |
| New York--Newark, NY--NJ--CT | St. James CDP | 37.1% | 16.0% |
| New York--Newark, NY--NJ--CT | Stanhope borough | 64.2% | 16.0% |
| New York--Newark, NY--NJ--CT | Stewart Manor village | 37.7% | 16.0% |
| New York--Newark, NY--NJ--CT | Stony Brook CDP | 38.5% | 16.0% |
| New York--Newark, NY--NJ--CT | Stony Brook University CDP | 14.0% | 16.0% |
| New York--Newark, NY--NJ--CT | Stony Point CDP | 39.0% | 16.0% |
| New York--Newark, NY--NJ--CT | Strathmore CDP | 43.1% | 16.0% |
| New York--Newark, NY--NJ--CT | Succasunna CDP | 57.6% | 16.0% |
| New York--Newark, NY--NJ--CT | Suffern village | 38.6% | 16.0% |
| New York--Newark, NY--NJ--CT | Summit city | 40.4% | 16.0% |
| New York--Newark, NY--NJ--CT | Surf City borough | 0.7% | 16.0% |
| New York--Newark, NY--NJ--CT | Syosset CDP | 26.6% | 16.0% |
| New York--Newark, NY--NJ--CT | Tappan CDP | 52.0% | 16.0% |
| New York--Newark, NY--NJ--CT | Tarrytown village | 19.2% | 16.0% |
| New York--Newark, NY--NJ--CT | Ten Mile Run CDP | 75.9% | 16.0% |
| New York--Newark, NY--NJ--CT | Tenafly borough | 52.0% | 16.0% |
| New York--Newark, NY--NJ--CT | Terryville CDP | 32.9% | 16.0% |
| New York--Newark, NY--NJ--CT | Teterboro borough | 15.2% | 16.0% |
| New York--Newark, NY--NJ--CT | Thiells CDP | 38.5% | 16.0% |
| New York--Newark, NY--NJ--CT | Thomaston village | 54.2% | 16.0% |
| New York--Newark, NY--NJ--CT | Thornwood CDP | 26.8% | 16.0% |
| New York--Newark, NY--NJ--CT | Tinton Falls borough | 62.7% | 16.0% |
| New York--Newark, NY--NJ--CT | Toms River CDP | 37.5% | 16.0% |
| New York--Newark, NY--NJ--CT | Totowa borough | 26.8% | 16.0% |
| New York--Newark, NY--NJ--CT | Tuckahoe CDP | 16.9% | 16.0% |
| New York--Newark, NY--NJ--CT | Tuckahoe village | 17.9% | 16.0% |
| New York--Newark, NY--NJ--CT | Union Beach borough | 26.9% | 16.0% |
| New York--Newark, NY--NJ--CT | Union City city | 4.3% | 16.0% |
| New York--Newark, NY--NJ--CT | Uniondale CDP | 16.4% | 16.0% |
| New York--Newark, NY--NJ--CT | University Gardens CDP | 34.3% | 16.0% |
| New York--Newark, NY--NJ--CT | Upper Brookville village | 65.5% | 16.0% |
| New York--Newark, NY--NJ--CT | Upper Montclair CDP | 63.0% | 16.0% |
| New York--Newark, NY--NJ--CT | Upper Nyack village | 46.5% | 16.0% |
| New York--Newark, NY--NJ--CT | Upper Saddle River borough | 63.1% | 16.0% |
| New York--Newark, NY--NJ--CT | Valhalla CDP | 22.8% | 16.0% |
| New York--Newark, NY--NJ--CT | Valley Cottage CDP | 44.7% | 16.0% |
| New York--Newark, NY--NJ--CT | Valley Stream village | 12.0% | 16.0% |
| New York--Newark, NY--NJ--CT | Verplanck CDP | 29.4% | 16.0% |
| New York--Newark, NY--NJ--CT | Victory Gardens borough | 36.2% | 16.0% |
| New York--Newark, NY--NJ--CT | Vienna CDP | 80.4% | 16.0% |
| New York--Newark, NY--NJ--CT | Village of the Branch village | 47.7% | 16.0% |
| New York--Newark, NY--NJ--CT | Viola CDP | 48.4% | 16.0% |
| New York--Newark, NY--NJ--CT | Vista Center CDP | 53.7% | 16.0% |
| New York--Newark, NY--NJ--CT | Voorhees CDP | 43.7% | 16.0% |
| New York--Newark, NY--NJ--CT | Wading River CDP | 22.0% | 16.0% |
| New York--Newark, NY--NJ--CT | Waldwick borough | 39.2% | 16.0% |
| New York--Newark, NY--NJ--CT | Wallington borough | 16.3% | 16.0% |
| New York--Newark, NY--NJ--CT | Wanamassa CDP | 37.9% | 16.0% |
| New York--Newark, NY--NJ--CT | Wanaque borough | 53.7% | 16.0% |
| New York--Newark, NY--NJ--CT | Wantagh CDP | 11.8% | 16.0% |
| New York--Newark, NY--NJ--CT | Waretown CDP | 28.9% | 16.0% |
| New York--Newark, NY--NJ--CT | Watchung borough | 77.1% | 16.0% |
| New York--Newark, NY--NJ--CT | Water Mill CDP | 33.4% | 16.0% |
| New York--Newark, NY--NJ--CT | Wesley Hills village | 54.5% | 16.0% |
| New York--Newark, NY--NJ--CT | West Babylon CDP | 21.4% | 16.0% |
| New York--Newark, NY--NJ--CT | West Bay Shore CDP | 28.5% | 16.0% |
| New York--Newark, NY--NJ--CT | West Belmar CDP | 33.1% | 16.0% |
| New York--Newark, NY--NJ--CT | West Freehold CDP | 47.4% | 16.0% |
| New York--Newark, NY--NJ--CT | West Haverstraw village | 24.1% | 16.0% |
| New York--Newark, NY--NJ--CT | West Hempstead CDP | 21.0% | 16.0% |
| New York--Newark, NY--NJ--CT | West Hills CDP | 56.7% | 16.0% |
| New York--Newark, NY--NJ--CT | West Islip CDP | 24.5% | 16.0% |
| New York--Newark, NY--NJ--CT | West Long Branch borough | 28.5% | 16.0% |
| New York--Newark, NY--NJ--CT | West New York town | 2.4% | 16.0% |
| New York--Newark, NY--NJ--CT | West Nyack CDP | 52.9% | 16.0% |
| New York--Newark, NY--NJ--CT | West Sayville CDP | 18.6% | 16.0% |
| New York--Newark, NY--NJ--CT | Westbury village | 16.6% | 16.0% |
| New York--Newark, NY--NJ--CT | Westfield town | 33.1% | 16.0% |
| New York--Newark, NY--NJ--CT | Westhampton Beach village | 12.9% | 16.0% |
| New York--Newark, NY--NJ--CT | Westhampton CDP | 18.2% | 16.0% |
| New York--Newark, NY--NJ--CT | Weston CDP | 40.9% | 16.0% |
| New York--Newark, NY--NJ--CT | Westwood borough | 40.8% | 16.0% |
| New York--Newark, NY--NJ--CT | Wharton borough | 30.2% | 16.0% |
| New York--Newark, NY--NJ--CT | Wheatley Heights CDP | 22.6% | 16.0% |
| New York--Newark, NY--NJ--CT | White House Station CDP | 42.4% | 16.0% |
| New York--Newark, NY--NJ--CT | White Meadow Lake CDP | 62.9% | 16.0% |
| New York--Newark, NY--NJ--CT | White Plains city | 8.8% | 16.0% |
| New York--Newark, NY--NJ--CT | Whittingham CDP | 30.2% | 16.0% |
| New York--Newark, NY--NJ--CT | Williston Park village | 13.5% | 16.0% |
| New York--Newark, NY--NJ--CT | Wood-Ridge borough | 18.9% | 16.0% |
| New York--Newark, NY--NJ--CT | Woodbridge CDP | 22.1% | 16.0% |
| New York--Newark, NY--NJ--CT | Woodbury CDP | 42.0% | 16.0% |
| New York--Newark, NY--NJ--CT | Woodcliff Lake borough | 63.9% | 16.0% |
| New York--Newark, NY--NJ--CT | Woodland Park borough | 34.0% | 16.0% |
| New York--Newark, NY--NJ--CT | Woodmere CDP | 25.3% | 16.0% |
| New York--Newark, NY--NJ--CT | Woodsburgh village | 52.0% | 16.0% |
| New York--Newark, NY--NJ--CT | Wyandanch CDP | 27.8% | 16.0% |
| New York--Newark, NY--NJ--CT | Yaphank CDP | 24.9% | 16.0% |
| New York--Newark, NY--NJ--CT | Yonkers city | 19.6% | 16.0% |
| New York--Newark, NY--NJ--CT | Yorketown CDP | 42.5% | 16.0% |
| New York--Newark, NY--NJ--CT | Yorktown Heights CDP | 24.4% | 16.0% |
| New York--Newark, NY--NJ--CT | Zarephath CDP | 28.3% | 16.0% |
| Ogden--Layton, UT | Bountiful city | 24.8% | 17.7% |
| Ogden--Layton, UT | Brigham City city | 20.2% | 17.7% |
| Ogden--Layton, UT | Centerville city | 22.5% | 17.7% |
| Ogden--Layton, UT | Clearfield city | 13.2% | 17.7% |
| Ogden--Layton, UT | Clinton city | 16.7% | 17.7% |
| Ogden--Layton, UT | Farmington city | 19.8% | 17.7% |
| Ogden--Layton, UT | Farr West city | 15.5% | 17.7% |
| Ogden--Layton, UT | Fruit Heights city | 27.8% | 17.7% |
| Ogden--Layton, UT | Harrisville city | 13.1% | 17.7% |
| Ogden--Layton, UT | Hooper city | 10.4% | 17.7% |
| Ogden--Layton, UT | Kaysville city | 21.1% | 17.7% |
| Ogden--Layton, UT | Layton city | 16.8% | 17.7% |
| Ogden--Layton, UT | Marriott-Slaterville city | 13.1% | 17.7% |
| Ogden--Layton, UT | North Ogden city | 17.1% | 17.7% |
| Ogden--Layton, UT | North Salt Lake city | 21.7% | 17.7% |
| Ogden--Layton, UT | Ogden city | 18.6% | 17.7% |
| Ogden--Layton, UT | Perry city | 14.4% | 17.7% |
| Ogden--Layton, UT | Plain City city | 13.9% | 17.7% |
| Ogden--Layton, UT | Pleasant View city | 14.7% | 17.7% |
| Ogden--Layton, UT | Riverdale city | 21.0% | 17.7% |
| Ogden--Layton, UT | Roy city | 15.4% | 17.7% |
| Ogden--Layton, UT | South Ogden city | 17.7% | 17.7% |
| Ogden--Layton, UT | South Weber city | 13.5% | 17.7% |
| Ogden--Layton, UT | South Willard CDP | 10.8% | 17.7% |
| Ogden--Layton, UT | Sunset city | 16.7% | 17.7% |
| Ogden--Layton, UT | Syracuse city | 15.3% | 17.7% |
| Ogden--Layton, UT | Uintah town | 15.8% | 17.7% |
| Ogden--Layton, UT | Washington Terrace city | 18.9% | 17.7% |
| Ogden--Layton, UT | West Bountiful city | 27.7% | 17.7% |
| Ogden--Layton, UT | West Haven city | 10.6% | 17.7% |
| Ogden--Layton, UT | West Point city | 13.1% | 17.7% |
| Ogden--Layton, UT | Willard city | 15.0% | 17.7% |
| Ogden--Layton, UT | Woods Cross city | 24.3% | 17.7% |
| Oklahoma City, OK | Bethany city | 27.6% | 22.3% |
| Oklahoma City, OK | Choctaw city | 34.7% | 22.3% |
| Oklahoma City, OK | Del City city | 32.3% | 22.3% |
| Oklahoma City, OK | Edmond city | 28.4% | 22.3% |
| Oklahoma City, OK | Forest Park town | 51.9% | 22.3% |
| Oklahoma City, OK | Guthrie city | 20.3% | 22.3% |
| Oklahoma City, OK | Harrah city | 40.8% | 22.3% |
| Oklahoma City, OK | Jones town | 41.3% | 22.3% |
| Oklahoma City, OK | Midwest City city | 25.2% | 22.3% |
| Oklahoma City, OK | Moore city | 15.4% | 22.3% |
| Oklahoma City, OK | Mustang city | 20.6% | 22.3% |
| Oklahoma City, OK | Nichols Hills city | 34.0% | 22.3% |
| Oklahoma City, OK | Nicoma Park city | 36.5% | 22.3% |
| Oklahoma City, OK | Norman city | 31.1% | 22.3% |
| Oklahoma City, OK | Oklahoma City city | 20.7% | 22.3% |
| Oklahoma City, OK | Smith Village town | 39.3% | 22.3% |
| Oklahoma City, OK | Spencer city | 32.4% | 22.3% |
| Oklahoma City, OK | The Village city | 29.8% | 22.3% |
| Oklahoma City, OK | Valley Brook town | 27.5% | 22.3% |
| Oklahoma City, OK | Warr Acres city | 26.9% | 22.3% |
| Oklahoma City, OK | Woodlawn Park town | 53.3% | 22.3% |
| Oklahoma City, OK | Yukon city | 21.0% | 22.3% |
| Omaha, NE--IA | Bellevue city | 14.7% | 11.7% |
| Omaha, NE--IA | Bennington city | 20.8% | 11.7% |
| Omaha, NE--IA | Boys Town village | 16.4% | 11.7% |
| Omaha, NE--IA | Carter Lake city | 7.1% | 11.7% |
| Omaha, NE--IA | Chalco CDP | 19.7% | 11.7% |
| Omaha, NE--IA | Council Bluffs city | 12.7% | 11.7% |
| Omaha, NE--IA | Gretna city | 17.0% | 11.7% |
| Omaha, NE--IA | La Platte CDP | 6.7% | 11.7% |
| Omaha, NE--IA | La Vista city | 6.8% | 11.7% |
| Omaha, NE--IA | Offutt AFB CDP | 5.3% | 11.7% |
| Omaha, NE--IA | Omaha city | 12.3% | 11.7% |
| Omaha, NE--IA | Papillion city | 9.7% | 11.7% |
| Omaha, NE--IA | Ralston city | 8.7% | 11.7% |
| Omaha, NE--IA | Waterloo village | 12.0% | 11.7% |
| Orlando, FL | Alafaya CDP | 43.4% | 43.5% |
| Orlando, FL | Altamonte Springs city | 43.2% | 43.5% |
| Orlando, FL | Apopka city | 43.3% | 43.5% |
| Orlando, FL | Azalea Park CDP | 38.8% | 43.5% |
| Orlando, FL | Bay Hill CDP | 42.5% | 43.5% |
| Orlando, FL | Belle Isle city | 47.6% | 43.5% |
| Orlando, FL | Bithlo CDP | 50.6% | 43.5% |
| Orlando, FL | Casselberry city | 42.9% | 43.5% |
| Orlando, FL | Celebration CDP | 52.3% | 43.5% |
| Orlando, FL | Chuluota CDP | 42.2% | 43.5% |
| Orlando, FL | Clarcona CDP | 59.9% | 43.5% |
| Orlando, FL | Clermont city | 36.7% | 43.5% |
| Orlando, FL | Conway CDP | 47.5% | 43.5% |
| Orlando, FL | Doctor Phillips CDP | 38.7% | 43.5% |
| Orlando, FL | Eatonville town | 47.4% | 43.5% |
| Orlando, FL | Edgewood city | 49.8% | 43.5% |
| Orlando, FL | Fairview Shores CDP | 45.6% | 43.5% |
| Orlando, FL | Fern Park CDP | 40.3% | 43.5% |
| Orlando, FL | Forest City CDP | 48.2% | 43.5% |
| Orlando, FL | Goldenrod CDP | 42.2% | 43.5% |
| Orlando, FL | Gotha CDP | 42.2% | 43.5% |
| Orlando, FL | Groveland city | 20.2% | 43.5% |
| Orlando, FL | Heathrow CDP | 51.9% | 43.5% |
| Orlando, FL | Holden Heights CDP | 45.7% | 43.5% |
| Orlando, FL | Horizon West CDP | 32.7% | 43.5% |
| Orlando, FL | Lake Butler CDP | 45.2% | 43.5% |
| Orlando, FL | Lake Mary city | 50.4% | 43.5% |
| Orlando, FL | Lockhart CDP | 50.3% | 43.5% |
| Orlando, FL | Longwood city | 48.0% | 43.5% |
| Orlando, FL | Maitland city | 50.8% | 43.5% |
| Orlando, FL | Mascotte city | 28.2% | 43.5% |
| Orlando, FL | Midway CDP | 43.5% | 43.5% |
| Orlando, FL | Minneola city | 30.3% | 43.5% |
| Orlando, FL | Montverde town | 51.4% | 43.5% |
| Orlando, FL | Mount Plymouth CDP | 70.4% | 43.5% |
| Orlando, FL | Oak Ridge CDP | 35.2% | 43.5% |
| Orlando, FL | Oakland town | 46.9% | 43.5% |
| Orlando, FL | Ocoee city | 42.1% | 43.5% |
| Orlando, FL | Orlando city | 42.6% | 43.5% |
| Orlando, FL | Orlovista CDP | 40.5% | 43.5% |
| Orlando, FL | Oviedo city | 39.6% | 43.5% |
| Orlando, FL | Paradise Heights CDP | 60.0% | 43.5% |
| Orlando, FL | Pine Castle CDP | 45.7% | 43.5% |
| Orlando, FL | Pine Hills CDP | 31.8% | 43.5% |
| Orlando, FL | Rio Pinar CDP | 52.0% | 43.5% |
| Orlando, FL | Sanford city | 42.0% | 43.5% |
| Orlando, FL | Sky Lake CDP | 28.3% | 43.5% |
| Orlando, FL | Sorrento CDP | 72.3% | 43.5% |
| Orlando, FL | South Apopka CDP | 46.8% | 43.5% |
| Orlando, FL | Taft CDP | 57.6% | 43.5% |
| Orlando, FL | Tangelo Park CDP | 31.3% | 43.5% |
| Orlando, FL | Tangerine CDP | 59.9% | 43.5% |
| Orlando, FL | Tildenville CDP | 63.8% | 43.5% |
| Orlando, FL | Union Park CDP | 46.2% | 43.5% |
| Orlando, FL | University CDP | 32.2% | 43.5% |
| Orlando, FL | Wekiwa Springs CDP | 54.4% | 43.5% |
| Orlando, FL | Windermere town | 60.5% | 43.5% |
| Orlando, FL | Winter Garden city | 34.6% | 43.5% |
| Orlando, FL | Winter Park city | 54.6% | 43.5% |
| Orlando, FL | Winter Springs city | 51.2% | 43.5% |
| Orlando, FL | Zellwood CDP | 38.3% | 43.5% |
| Palm Bay--Melbourne, FL | Cape Canaveral city | 18.6% | 35.3% |
| Palm Bay--Melbourne, FL | Cocoa Beach city | 20.9% | 35.3% |
| Palm Bay--Melbourne, FL | Cocoa West CDP | 34.6% | 35.3% |
| Palm Bay--Melbourne, FL | Cocoa city | 27.5% | 35.3% |
| Palm Bay--Melbourne, FL | Grant-Valkaria town | 52.6% | 35.3% |
| Palm Bay--Melbourne, FL | Indialantic town | 30.2% | 35.3% |
| Palm Bay--Melbourne, FL | Indian Harbour Beach city | 20.4% | 35.3% |
| Palm Bay--Melbourne, FL | June Park CDP | 59.1% | 35.3% |
| Palm Bay--Melbourne, FL | Malabar town | 66.2% | 35.3% |
| Palm Bay--Melbourne, FL | Melbourne Beach town | 28.1% | 35.3% |
| Palm Bay--Melbourne, FL | Melbourne Village town | 85.3% | 35.3% |
| Palm Bay--Melbourne, FL | Melbourne city | 29.2% | 35.3% |
| Palm Bay--Melbourne, FL | Merritt Island CDP | 25.1% | 35.3% |
| Palm Bay--Melbourne, FL | Palm Bay city | 45.1% | 35.3% |
| Palm Bay--Melbourne, FL | Palm Shores town | 35.0% | 35.3% |
| Palm Bay--Melbourne, FL | Patrick AFB CDP | 8.5% | 35.3% |
| Palm Bay--Melbourne, FL | Port St. John CDP | 34.0% | 35.3% |
| Palm Bay--Melbourne, FL | Rockledge city | 34.1% | 35.3% |
| Palm Bay--Melbourne, FL | Satellite Beach city | 19.7% | 35.3% |
| Palm Bay--Melbourne, FL | Sharpes CDP | 42.6% | 35.3% |
| Palm Bay--Melbourne, FL | South Patrick Shores CDP | 27.2% | 35.3% |
| Palm Bay--Melbourne, FL | Viera East CDP | 43.5% | 35.3% |
| Palm Bay--Melbourne, FL | Viera West CDP | 29.8% | 35.3% |
| Palm Bay--Melbourne, FL | West Melbourne city | 34.2% | 35.3% |
| Pensacola, FL--AL | Avalon CDP | 54.4% | 43.3% |
| Pensacola, FL--AL | Bagdad CDP | 57.4% | 43.3% |
| Pensacola, FL--AL | Bellview CDP | 41.1% | 43.3% |
| Pensacola, FL--AL | Brent CDP | 44.9% | 43.3% |
| Pensacola, FL--AL | East Milton CDP | 21.1% | 43.3% |
| Pensacola, FL--AL | Ensley CDP | 47.1% | 43.3% |
| Pensacola, FL--AL | Ferry Pass CDP | 50.7% | 43.3% |
| Pensacola, FL--AL | Floridatown CDP | 47.0% | 43.3% |
| Pensacola, FL--AL | Gonzalez CDP | 49.1% | 43.3% |
| Pensacola, FL--AL | Goulding CDP | 25.6% | 43.3% |
| Pensacola, FL--AL | Gulf Breeze city | 45.7% | 43.3% |
| Pensacola, FL--AL | Lillian CDP | 40.0% | 43.3% |
| Pensacola, FL--AL | Milton city | 37.9% | 43.3% |
| Pensacola, FL--AL | Myrtle Grove CDP | 45.2% | 43.3% |
| Pensacola, FL--AL | Orange Beach city | 48.1% | 43.3% |
| Pensacola, FL--AL | Pace CDP | 46.5% | 43.3% |
| Pensacola, FL--AL | Pea Ridge CDP | 43.3% | 43.3% |
| Pensacola, FL--AL | Pensacola city | 36.2% | 43.3% |
| Pensacola, FL--AL | Point Baker CDP | 59.4% | 43.3% |
| Pensacola, FL--AL | Wallace CDP | 72.0% | 43.3% |
| Pensacola, FL--AL | Warrington CDP | 34.6% | 43.3% |
| Pensacola, FL--AL | West Pensacola CDP | 38.9% | 43.3% |
| Philadelphia, PA--NJ--DE--MD | Aldan borough | 22.3% | 22.2% |
| Philadelphia, PA--NJ--DE--MD | Ambler borough | 19.7% | 22.2% |
| Philadelphia, PA--NJ--DE--MD | Arcadia University CDP | 26.0% | 22.2% |
| Philadelphia, PA--NJ--DE--MD | Arden village | 65.6% | 22.2% |
| Philadelphia, PA--NJ--DE--MD | Ardencroft village | 72.5% | 22.2% |
| Philadelphia, PA--NJ--DE--MD | Ardentown village | 78.5% | 22.2% |
| Philadelphia, PA--NJ--DE--MD | Ardmore CDP | 17.8% | 22.2% |
| Philadelphia, PA--NJ--DE--MD | Ashland CDP | 38.3% | 22.2% |
| Philadelphia, PA--NJ--DE--MD | Atglen borough | 42.2% | 22.2% |
| Philadelphia, PA--NJ--DE--MD | Audubon CDP | 36.3% | 22.2% |
| Philadelphia, PA--NJ--DE--MD | Audubon Park borough | 18.0% | 22.2% |
| Philadelphia, PA--NJ--DE--MD | Audubon borough | 29.1% | 22.2% |
| Philadelphia, PA--NJ--DE--MD | Avondale borough | 24.8% | 22.2% |
| Philadelphia, PA--NJ--DE--MD | Barclay CDP | 59.2% | 22.2% |
| Philadelphia, PA--NJ--DE--MD | Barrington borough | 39.8% | 22.2% |
| Philadelphia, PA--NJ--DE--MD | Bear CDP | 41.0% | 22.2% |
| Philadelphia, PA--NJ--DE--MD | Beckett CDP | 27.5% | 22.2% |
| Philadelphia, PA--NJ--DE--MD | Bellefonte town | 23.1% | 22.2% |
| Philadelphia, PA--NJ--DE--MD | Bellmawr borough | 0.0% | 22.2% |
| Philadelphia, PA--NJ--DE--MD | Berlin borough | 35.3% | 22.2% |
| Philadelphia, PA--NJ--DE--MD | Berwyn CDP | 43.6% | 22.2% |
| Philadelphia, PA--NJ--DE--MD | Beverly city | 21.6% | 22.2% |
| Philadelphia, PA--NJ--DE--MD | Blackwood CDP | 27.4% | 22.2% |
| Philadelphia, PA--NJ--DE--MD | Blue Bell CDP | 43.5% | 22.2% |
| Philadelphia, PA--NJ--DE--MD | Boothwyn CDP | 26.6% | 22.2% |
| Philadelphia, PA--NJ--DE--MD | Bridgeport borough | 1.1% | 22.2% |
| Philadelphia, PA--NJ--DE--MD | Bristol borough | 6.1% | 22.2% |
| Philadelphia, PA--NJ--DE--MD | Brittany Farms-The Highlands CDP | 22.7% | 22.2% |
| Philadelphia, PA--NJ--DE--MD | Brookhaven borough | 16.7% | 22.2% |
| Philadelphia, PA--NJ--DE--MD | Brooklawn borough | 18.1% | 22.2% |
| Philadelphia, PA--NJ--DE--MD | Brookside CDP | 34.9% | 22.2% |
| Philadelphia, PA--NJ--DE--MD | Broomall CDP | 23.1% | 22.2% |
| Philadelphia, PA--NJ--DE--MD | Bryn Athyn borough | 56.9% | 22.2% |
| Philadelphia, PA--NJ--DE--MD | Bryn Mawr CDP | 14.7% | 22.2% |
| Philadelphia, PA--NJ--DE--MD | Burlington city | 18.4% | 22.2% |
| Philadelphia, PA--NJ--DE--MD | Caln CDP | 15.6% | 22.2% |
| Philadelphia, PA--NJ--DE--MD | Camden city | 10.0% | 22.2% |
| Philadelphia, PA--NJ--DE--MD | Carneys Point CDP | 44.0% | 22.2% |
| Philadelphia, PA--NJ--DE--MD | Chalfont borough | 31.8% | 22.2% |
| Philadelphia, PA--NJ--DE--MD | Charlestown town | 47.0% | 22.2% |
| Philadelphia, PA--NJ--DE--MD | Cherry Hill Mall CDP | 36.1% | 22.2% |
| Philadelphia, PA--NJ--DE--MD | Chesapeake City town | 12.8% | 22.2% |
| Philadelphia, PA--NJ--DE--MD | Chesilhurst borough | 73.1% | 22.2% |
| Philadelphia, PA--NJ--DE--MD | Chester Heights borough | 53.4% | 22.2% |
| Philadelphia, PA--NJ--DE--MD | Chester city | 7.9% | 22.2% |
| Philadelphia, PA--NJ--DE--MD | Chesterbrook CDP | 34.9% | 22.2% |
| Philadelphia, PA--NJ--DE--MD | Cheyney University CDP | 35.9% | 22.2% |
| Philadelphia, PA--NJ--DE--MD | Christiana borough | 33.3% | 22.2% |
| Philadelphia, PA--NJ--DE--MD | Churchville CDP | 28.5% | 22.2% |
| Philadelphia, PA--NJ--DE--MD | Claymont CDP | 30.8% | 22.2% |
| Philadelphia, PA--NJ--DE--MD | Clayton borough | 38.8% | 22.2% |
| Philadelphia, PA--NJ--DE--MD | Clementon borough | 47.2% | 22.2% |
| Philadelphia, PA--NJ--DE--MD | Clifton Heights borough | 9.1% | 22.2% |
| Philadelphia, PA--NJ--DE--MD | Coatesville city | 2.3% | 22.2% |
| Philadelphia, PA--NJ--DE--MD | Collegeville borough | 35.8% | 22.2% |
| Philadelphia, PA--NJ--DE--MD | Collingdale borough | 11.2% | 22.2% |
| Philadelphia, PA--NJ--DE--MD | Collings Lakes CDP | 56.0% | 22.2% |
| Philadelphia, PA--NJ--DE--MD | Collingswood borough | 29.4% | 22.2% |
| Philadelphia, PA--NJ--DE--MD | Colwyn borough | 3.9% | 22.2% |
| Philadelphia, PA--NJ--DE--MD | Conshohocken borough | 6.7% | 22.2% |
| Philadelphia, PA--NJ--DE--MD | Cornwells Heights CDP | 24.4% | 22.2% |
| Philadelphia, PA--NJ--DE--MD | Croydon CDP | 19.8% | 22.2% |
| Philadelphia, PA--NJ--DE--MD | Darby borough | 8.9% | 22.2% |
| Philadelphia, PA--NJ--DE--MD | Devon CDP | 45.2% | 22.2% |
| Philadelphia, PA--NJ--DE--MD | Downingtown borough | 11.5% | 22.2% |
| Philadelphia, PA--NJ--DE--MD | Doylestown borough | 22.6% | 22.2% |
| Philadelphia, PA--NJ--DE--MD | Drexel Hill CDP | 16.4% | 22.2% |
| Philadelphia, PA--NJ--DE--MD | Dublin borough | 15.9% | 22.2% |
| Philadelphia, PA--NJ--DE--MD | Eagleview CDP | 24.4% | 22.2% |
| Philadelphia, PA--NJ--DE--MD | Eagleville CDP | 23.5% | 22.2% |
| Philadelphia, PA--NJ--DE--MD | East Greenville borough | 4.6% | 22.2% |
| Philadelphia, PA--NJ--DE--MD | East Lansdowne borough | 16.1% | 22.2% |
| Philadelphia, PA--NJ--DE--MD | Echelon CDP | 19.9% | 22.2% |
| Philadelphia, PA--NJ--DE--MD | Eddington CDP | 18.0% | 22.2% |
| Philadelphia, PA--NJ--DE--MD | Eddystone borough | 2.7% | 22.2% |
| Philadelphia, PA--NJ--DE--MD | Edgemoor CDP | 43.9% | 22.2% |
| Philadelphia, PA--NJ--DE--MD | Elkton town | 25.5% | 22.2% |
| Philadelphia, PA--NJ--DE--MD | Ellisburg CDP | 30.1% | 22.2% |
| Philadelphia, PA--NJ--DE--MD | Elsmere town | 32.4% | 22.2% |
| Philadelphia, PA--NJ--DE--MD | Evansburg CDP | 36.9% | 22.2% |
| Philadelphia, PA--NJ--DE--MD | Exton CDP | 37.5% | 22.2% |
| Philadelphia, PA--NJ--DE--MD | Fairless Hills CDP | 15.6% | 22.2% |
| Philadelphia, PA--NJ--DE--MD | Feasterville CDP | 27.8% | 22.2% |
| Philadelphia, PA--NJ--DE--MD | Florence CDP | 17.6% | 22.2% |
| Philadelphia, PA--NJ--DE--MD | Flourtown CDP | 39.1% | 22.2% |
| Philadelphia, PA--NJ--DE--MD | Folcroft borough | 10.6% | 22.2% |
| Philadelphia, PA--NJ--DE--MD | Folsom CDP | 15.7% | 22.2% |
| Philadelphia, PA--NJ--DE--MD | Folsom borough | 58.2% | 22.2% |
| Philadelphia, PA--NJ--DE--MD | Fort Washington CDP | 45.6% | 22.2% |
| Philadelphia, PA--NJ--DE--MD | Gap CDP | 40.0% | 22.2% |
| Philadelphia, PA--NJ--DE--MD | Gibbsboro borough | 56.7% | 22.2% |
| Philadelphia, PA--NJ--DE--MD | Gibbstown CDP | 14.4% | 22.2% |
| Philadelphia, PA--NJ--DE--MD | Glasgow CDP | 41.9% | 22.2% |
| Philadelphia, PA--NJ--DE--MD | Glassboro borough | 32.3% | 22.2% |
| Philadelphia, PA--NJ--DE--MD | Glendora CDP | 0.0% | 22.2% |
| Philadelphia, PA--NJ--DE--MD | Glenolden borough | 17.7% | 22.2% |
| Philadelphia, PA--NJ--DE--MD | Glenside CDP | 22.9% | 22.2% |
| Philadelphia, PA--NJ--DE--MD | Gloucester City city | 11.3% | 22.2% |
| Philadelphia, PA--NJ--DE--MD | Golden Triangle CDP | 37.8% | 22.2% |
| Philadelphia, PA--NJ--DE--MD | Green Lane borough | 19.6% | 22.2% |
| Philadelphia, PA--NJ--DE--MD | Greentree CDP | 42.9% | 22.2% |
| Philadelphia, PA--NJ--DE--MD | Greenville CDP | 40.6% | 22.2% |
| Philadelphia, PA--NJ--DE--MD | Haddon Heights borough | 41.6% | 22.2% |
| Philadelphia, PA--NJ--DE--MD | Haddonfield borough | 54.3% | 22.2% |
| Philadelphia, PA--NJ--DE--MD | Harleysville CDP | 34.2% | 22.2% |
| Philadelphia, PA--NJ--DE--MD | Hatboro borough | 31.7% | 22.2% |
| Philadelphia, PA--NJ--DE--MD | Hatfield borough | 36.3% | 22.2% |
| Philadelphia, PA--NJ--DE--MD | Haverford College CDP | 47.1% | 22.2% |
| Philadelphia, PA--NJ--DE--MD | Hi-Nella borough | 22.2% | 22.2% |
| Philadelphia, PA--NJ--DE--MD | Hockessin CDP | 40.9% | 22.2% |
| Philadelphia, PA--NJ--DE--MD | Honey Brook borough | 36.3% | 22.2% |
| Philadelphia, PA--NJ--DE--MD | Horsham CDP | 40.6% | 22.2% |
| Philadelphia, PA--NJ--DE--MD | Hulmeville borough | 38.6% | 22.2% |
| Philadelphia, PA--NJ--DE--MD | Ivyland borough | 10.4% | 22.2% |
| Philadelphia, PA--NJ--DE--MD | Jenkintown borough | 29.5% | 22.2% |
| Philadelphia, PA--NJ--DE--MD | Kennett Square borough | 17.1% | 22.2% |
| Philadelphia, PA--NJ--DE--MD | King of Prussia CDP | 14.9% | 22.2% |
| Philadelphia, PA--NJ--DE--MD | Kingston Estates CDP | 36.7% | 22.2% |
| Philadelphia, PA--NJ--DE--MD | Kulpsville CDP | 31.8% | 22.2% |
| Philadelphia, PA--NJ--DE--MD | Lambertville city | 10.0% | 22.2% |
| Philadelphia, PA--NJ--DE--MD | Langhorne Manor borough | 51.1% | 22.2% |
| Philadelphia, PA--NJ--DE--MD | Langhorne borough | 42.8% | 22.2% |
| Philadelphia, PA--NJ--DE--MD | Lansdale borough | 20.2% | 22.2% |
| Philadelphia, PA--NJ--DE--MD | Lansdowne borough | 18.9% | 22.2% |
| Philadelphia, PA--NJ--DE--MD | Laurel Springs borough | 39.8% | 22.2% |
| Philadelphia, PA--NJ--DE--MD | Lawnside borough | 48.1% | 22.2% |
| Philadelphia, PA--NJ--DE--MD | Leisuretowne CDP | 20.9% | 22.2% |
| Philadelphia, PA--NJ--DE--MD | Levittown CDP | 15.0% | 22.2% |
| Philadelphia, PA--NJ--DE--MD | Lima CDP | 47.5% | 22.2% |
| Philadelphia, PA--NJ--DE--MD | Lincoln University CDP | 43.8% | 22.2% |
| Philadelphia, PA--NJ--DE--MD | Lindenwold borough | 33.2% | 22.2% |
| Philadelphia, PA--NJ--DE--MD | Linwood CDP | 6.5% | 22.2% |
| Philadelphia, PA--NJ--DE--MD | Lionville CDP | 37.1% | 22.2% |
| Philadelphia, PA--NJ--DE--MD | Magnolia borough | 30.1% | 22.2% |
| Philadelphia, PA--NJ--DE--MD | Malvern borough | 30.8% | 22.2% |
| Philadelphia, PA--NJ--DE--MD | Maple Glen CDP | 48.1% | 22.2% |
| Philadelphia, PA--NJ--DE--MD | Marcus Hook borough | 2.6% | 22.2% |
| Philadelphia, PA--NJ--DE--MD | Marlton CDP | 38.3% | 22.2% |
| Philadelphia, PA--NJ--DE--MD | Medford Lakes borough | 68.5% | 22.2% |
| Philadelphia, PA--NJ--DE--MD | Media borough | 18.8% | 22.2% |
| Philadelphia, PA--NJ--DE--MD | Merchantville borough | 30.2% | 22.2% |
| Philadelphia, PA--NJ--DE--MD | Millbourne borough | 6.9% | 22.2% |
| Philadelphia, PA--NJ--DE--MD | Modena borough | 39.6% | 22.2% |
| Philadelphia, PA--NJ--DE--MD | Montgomeryville CDP | 44.0% | 22.2% |
| Philadelphia, PA--NJ--DE--MD | Moorestown-Lenola CDP | 51.9% | 22.2% |
| Philadelphia, PA--NJ--DE--MD | Morrisville borough | 33.9% | 22.2% |
| Philadelphia, PA--NJ--DE--MD | Morton borough | 17.7% | 22.2% |
| Philadelphia, PA--NJ--DE--MD | Mount Ephraim borough | 20.1% | 22.2% |
| Philadelphia, PA--NJ--DE--MD | Mullica Hill CDP | 47.4% | 22.2% |
| Philadelphia, PA--NJ--DE--MD | Narberth borough | 16.9% | 22.2% |
| Philadelphia, PA--NJ--DE--MD | National Park borough | 21.4% | 22.2% |
| Philadelphia, PA--NJ--DE--MD | New Britain borough | 26.2% | 22.2% |
| Philadelphia, PA--NJ--DE--MD | New Castle city | 28.0% | 22.2% |
| Philadelphia, PA--NJ--DE--MD | New Hope borough | 20.8% | 22.2% |
| Philadelphia, PA--NJ--DE--MD | Newark city | 22.6% | 22.2% |
| Philadelphia, PA--NJ--DE--MD | Newport town | 31.9% | 22.2% |
| Philadelphia, PA--NJ--DE--MD | Newtown Grant CDP | 16.7% | 22.2% |
| Philadelphia, PA--NJ--DE--MD | Newtown borough | 27.4% | 22.2% |
| Philadelphia, PA--NJ--DE--MD | Norristown borough | 5.1% | 22.2% |
| Philadelphia, PA--NJ--DE--MD | North East town | 21.4% | 22.2% |
| Philadelphia, PA--NJ--DE--MD | North Star CDP | 46.6% | 22.2% |
| Philadelphia, PA--NJ--DE--MD | North Wales borough | 36.7% | 22.2% |
| Philadelphia, PA--NJ--DE--MD | Norwood borough | 14.8% | 22.2% |
| Philadelphia, PA--NJ--DE--MD | Oak Valley CDP | 16.9% | 22.2% |
| Philadelphia, PA--NJ--DE--MD | Oaklyn borough | 26.7% | 22.2% |
| Philadelphia, PA--NJ--DE--MD | Oreland CDP | 33.6% | 22.2% |
| Philadelphia, PA--NJ--DE--MD | Oxford borough | 25.9% | 22.2% |
| Philadelphia, PA--NJ--DE--MD | Palmyra borough | 23.6% | 22.2% |
| Philadelphia, PA--NJ--DE--MD | Paoli CDP | 37.6% | 22.2% |
| Philadelphia, PA--NJ--DE--MD | Parkesburg borough | 43.4% | 22.2% |
| Philadelphia, PA--NJ--DE--MD | Parkside borough | 9.8% | 22.2% |
| Philadelphia, PA--NJ--DE--MD | Paulsboro borough | 13.9% | 22.2% |
| Philadelphia, PA--NJ--DE--MD | Pemberton Heights CDP | 4.1% | 22.2% |
| Philadelphia, PA--NJ--DE--MD | Pemberton borough | 21.0% | 22.2% |
| Philadelphia, PA--NJ--DE--MD | Penn Wynne CDP | 34.9% | 22.2% |
| Philadelphia, PA--NJ--DE--MD | Penndel borough | 25.7% | 22.2% |
| Philadelphia, PA--NJ--DE--MD | Penns Grove borough | 31.8% | 22.2% |
| Philadelphia, PA--NJ--DE--MD | Pennsburg borough | 11.3% | 22.2% |
| Philadelphia, PA--NJ--DE--MD | Pennsville CDP | 35.0% | 22.2% |
| Philadelphia, PA--NJ--DE--MD | Perkasie borough | 29.9% | 22.2% |
| Philadelphia, PA--NJ--DE--MD | Philadelphia city | 2.8% | 22.2% |
| Philadelphia, PA--NJ--DE--MD | Phoenixville borough | 19.5% | 22.2% |
| Philadelphia, PA--NJ--DE--MD | Pike Creek CDP | 45.8% | 22.2% |
| Philadelphia, PA--NJ--DE--MD | Pike Creek Valley CDP | 57.2% | 22.2% |
| Philadelphia, PA--NJ--DE--MD | Pine Hill borough | 40.8% | 22.2% |
| Philadelphia, PA--NJ--DE--MD | Pine Valley borough | 71.1% | 22.2% |
| Philadelphia, PA--NJ--DE--MD | Pitman borough | 32.6% | 22.2% |
| Philadelphia, PA--NJ--DE--MD | Plumsteadville CDP | 24.3% | 22.2% |
| Philadelphia, PA--NJ--DE--MD | Plymouth Meeting CDP | 3.4% | 22.2% |
| Philadelphia, PA--NJ--DE--MD | Pomeroy CDP | 20.8% | 22.2% |
| Philadelphia, PA--NJ--DE--MD | Prospect Park borough | 13.9% | 22.2% |
| Philadelphia, PA--NJ--DE--MD | Ramblewood CDP | 42.8% | 22.2% |
| Philadelphia, PA--NJ--DE--MD | Red Hill borough | 4.2% | 22.2% |
| Philadelphia, PA--NJ--DE--MD | Richboro CDP | 25.5% | 22.2% |
| Philadelphia, PA--NJ--DE--MD | Richwood CDP | 41.9% | 22.2% |
| Philadelphia, PA--NJ--DE--MD | Ridley Park borough | 17.9% | 22.2% |
| Philadelphia, PA--NJ--DE--MD | Rising Sun town | 26.4% | 22.2% |
| Philadelphia, PA--NJ--DE--MD | Riverton borough | 38.4% | 22.2% |
| Philadelphia, PA--NJ--DE--MD | Rockledge borough | 19.6% | 22.2% |
| Philadelphia, PA--NJ--DE--MD | Roebling CDP | 13.3% | 22.2% |
| Philadelphia, PA--NJ--DE--MD | Rose Valley borough | 64.8% | 22.2% |
| Philadelphia, PA--NJ--DE--MD | Royersford borough | 23.8% | 22.2% |
| Philadelphia, PA--NJ--DE--MD | Runnemede borough | 0.0% | 22.2% |
| Philadelphia, PA--NJ--DE--MD | Rutledge borough | 31.0% | 22.2% |
| Philadelphia, PA--NJ--DE--MD | Schwenksville borough | 24.3% | 22.2% |
| Philadelphia, PA--NJ--DE--MD | Sellersville borough | 34.7% | 22.2% |
| Philadelphia, PA--NJ--DE--MD | Sharon Hill borough | 13.5% | 22.2% |
| Philadelphia, PA--NJ--DE--MD | Silverdale borough | 28.3% | 22.2% |
| Philadelphia, PA--NJ--DE--MD | Skippack CDP | 22.4% | 22.2% |
| Philadelphia, PA--NJ--DE--MD | Somerdale borough | 37.0% | 22.2% |
| Philadelphia, PA--NJ--DE--MD | Souderton borough | 17.4% | 22.2% |
| Philadelphia, PA--NJ--DE--MD | South Coatesville borough | 36.7% | 22.2% |
| Philadelphia, PA--NJ--DE--MD | Spring City borough | 24.1% | 22.2% |
| Philadelphia, PA--NJ--DE--MD | Spring House CDP | 56.4% | 22.2% |
| Philadelphia, PA--NJ--DE--MD | Spring Mount CDP | 40.8% | 22.2% |
| Philadelphia, PA--NJ--DE--MD | Springdale CDP | 48.1% | 22.2% |
| Philadelphia, PA--NJ--DE--MD | St. Georges CDP | 61.2% | 22.2% |
| Philadelphia, PA--NJ--DE--MD | Stockton borough | 29.7% | 22.2% |
| Philadelphia, PA--NJ--DE--MD | Stratford borough | 30.7% | 22.2% |
| Philadelphia, PA--NJ--DE--MD | Swarthmore borough | 52.9% | 22.2% |
| Philadelphia, PA--NJ--DE--MD | Swedesboro borough | 26.5% | 22.2% |
| Philadelphia, PA--NJ--DE--MD | Tavistock borough | 57.4% | 22.2% |
| Philadelphia, PA--NJ--DE--MD | Telford borough | 16.0% | 22.2% |
| Philadelphia, PA--NJ--DE--MD | Thorndale CDP | 27.5% | 22.2% |
| Philadelphia, PA--NJ--DE--MD | Toughkenamon CDP | 48.9% | 22.2% |
| Philadelphia, PA--NJ--DE--MD | Trainer borough | 13.8% | 22.2% |
| Philadelphia, PA--NJ--DE--MD | Trappe borough | 47.7% | 22.2% |
| Philadelphia, PA--NJ--DE--MD | Trevose CDP | 31.9% | 22.2% |
| Philadelphia, PA--NJ--DE--MD | Trooper CDP | 33.5% | 22.2% |
| Philadelphia, PA--NJ--DE--MD | Tullytown borough | 12.2% | 22.2% |
| Philadelphia, PA--NJ--DE--MD | Turnersville CDP | 57.6% | 22.2% |
| Philadelphia, PA--NJ--DE--MD | Upland borough | 15.3% | 22.2% |
| Philadelphia, PA--NJ--DE--MD | Victory Lakes CDP | 73.4% | 22.2% |
| Philadelphia, PA--NJ--DE--MD | Village Green-Green Ridge CDP | 21.9% | 22.2% |
| Philadelphia, PA--NJ--DE--MD | Village Shires CDP | 29.4% | 22.2% |
| Philadelphia, PA--NJ--DE--MD | Warminster Heights CDP | 16.7% | 22.2% |
| Philadelphia, PA--NJ--DE--MD | Wenonah borough | 51.4% | 22.2% |
| Philadelphia, PA--NJ--DE--MD | West Chester borough | 7.2% | 22.2% |
| Philadelphia, PA--NJ--DE--MD | West Conshohocken borough | 22.5% | 22.2% |
| Philadelphia, PA--NJ--DE--MD | West Grove borough | 18.6% | 22.2% |
| Philadelphia, PA--NJ--DE--MD | Westville borough | 22.8% | 22.2% |
| Philadelphia, PA--NJ--DE--MD | Westwood CDP | 34.5% | 22.2% |
| Philadelphia, PA--NJ--DE--MD | Williamstown CDP | 32.3% | 22.2% |
| Philadelphia, PA--NJ--DE--MD | Willow Grove CDP | 32.7% | 22.2% |
| Philadelphia, PA--NJ--DE--MD | Wilmington Manor CDP | 26.9% | 22.2% |
| Philadelphia, PA--NJ--DE--MD | Wilmington city | 15.9% | 22.2% |
| Philadelphia, PA--NJ--DE--MD | Woodbourne CDP | 42.1% | 22.2% |
| Philadelphia, PA--NJ--DE--MD | Woodbury Heights borough | 37.8% | 22.2% |
| Philadelphia, PA--NJ--DE--MD | Woodbury city | 27.7% | 22.2% |
| Philadelphia, PA--NJ--DE--MD | Woodlyn CDP | 14.3% | 22.2% |
| Philadelphia, PA--NJ--DE--MD | Woodlynne borough | 18.1% | 22.2% |
| Philadelphia, PA--NJ--DE--MD | Woodside CDP | 44.1% | 22.2% |
| Philadelphia, PA--NJ--DE--MD | Woxall CDP | 50.2% | 22.2% |
| Philadelphia, PA--NJ--DE--MD | Wyncote CDP | 52.0% | 22.2% |
| Philadelphia, PA--NJ--DE--MD | Wyndmoor CDP | 43.4% | 22.2% |
| Philadelphia, PA--NJ--DE--MD | Yardley borough | 44.9% | 22.2% |
| Philadelphia, PA--NJ--DE--MD | Yeadon borough | 7.1% | 22.2% |
| Phoenix--Mesa, AZ | Anthem CDP | 8.4% | 8.9% |
| Phoenix--Mesa, AZ | Apache Junction city | 7.2% | 8.9% |
| Phoenix--Mesa, AZ | Avondale city | 14.0% | 8.9% |
| Phoenix--Mesa, AZ | Carefree town | 2.5% | 8.9% |
| Phoenix--Mesa, AZ | Cave Creek town | 9.7% | 8.9% |
| Phoenix--Mesa, AZ | Chandler city | 10.4% | 8.9% |
| Phoenix--Mesa, AZ | El Mirage city | 5.9% | 8.9% |
| Phoenix--Mesa, AZ | Fountain Hills town | 3.9% | 8.9% |
| Phoenix--Mesa, AZ | Gilbert town | 12.1% | 8.9% |
| Phoenix--Mesa, AZ | Glendale city | 9.1% | 8.9% |
| Phoenix--Mesa, AZ | Gold Canyon CDP | 9.0% | 8.9% |
| Phoenix--Mesa, AZ | Goodyear Village CDP | 1.0% | 8.9% |
| Phoenix--Mesa, AZ | Guadalupe town | 6.4% | 8.9% |
| Phoenix--Mesa, AZ | Komatke CDP | 2.7% | 8.9% |
| Phoenix--Mesa, AZ | Maricopa Colony CDP | 5.5% | 8.9% |
| Phoenix--Mesa, AZ | Mesa city | 8.4% | 8.9% |
| Phoenix--Mesa, AZ | New River CDP | 3.2% | 8.9% |
| Phoenix--Mesa, AZ | Paradise Valley town | 20.6% | 8.9% |
| Phoenix--Mesa, AZ | Peoria city | 7.6% | 8.9% |
| Phoenix--Mesa, AZ | Phoenix city | 8.2% | 8.9% |
| Phoenix--Mesa, AZ | Queen Creek town | 12.5% | 8.9% |
| Phoenix--Mesa, AZ | San Tan Valley CDP | 12.8% | 8.9% |
| Phoenix--Mesa, AZ | Scottsdale city | 9.2% | 8.9% |
| Phoenix--Mesa, AZ | Sun City CDP | 8.0% | 8.9% |
| Phoenix--Mesa, AZ | Sun City West CDP | 6.1% | 8.9% |
| Phoenix--Mesa, AZ | Sun Lakes CDP | 5.5% | 8.9% |
| Phoenix--Mesa, AZ | Surprise city | 8.1% | 8.9% |
| Phoenix--Mesa, AZ | Tempe city | 11.7% | 8.9% |
| Phoenix--Mesa, AZ | Tolleson city | 8.5% | 8.9% |
| Phoenix--Mesa, AZ | Youngtown town | 6.6% | 8.9% |
| Pittsburgh, PA | Adamsburg borough | 20.2% | 31.3% |
| Pittsburgh, PA | Aliquippa city | 22.8% | 31.3% |
| Pittsburgh, PA | Allison Park CDP | 45.1% | 31.3% |
| Pittsburgh, PA | Ambridge borough | 7.5% | 31.3% |
| Pittsburgh, PA | Apollo borough | 8.1% | 31.3% |
| Pittsburgh, PA | Arnold city | 12.9% | 31.3% |
| Pittsburgh, PA | Arona borough | 42.9% | 31.3% |
| Pittsburgh, PA | Aspinwall borough | 10.4% | 31.3% |
| Pittsburgh, PA | Avalon borough | 30.7% | 31.3% |
| Pittsburgh, PA | Baden borough | 36.4% | 31.3% |
| Pittsburgh, PA | Bairdford CDP | 27.2% | 31.3% |
| Pittsburgh, PA | Bakerstown CDP | 42.5% | 31.3% |
| Pittsburgh, PA | Baldwin borough | 34.4% | 31.3% |
| Pittsburgh, PA | Beaver Falls city | 4.5% | 31.3% |
| Pittsburgh, PA | Beaver borough | 15.9% | 31.3% |
| Pittsburgh, PA | Bell Acres borough | 4.4% | 31.3% |
| Pittsburgh, PA | Bellevue borough | 36.8% | 31.3% |
| Pittsburgh, PA | Ben Avon Heights borough | 60.8% | 31.3% |
| Pittsburgh, PA | Ben Avon borough | 50.7% | 31.3% |
| Pittsburgh, PA | Bethel Park municipality | 37.7% | 31.3% |
| Pittsburgh, PA | Big Beaver borough | 31.7% | 31.3% |
| Pittsburgh, PA | Blawnox borough | 6.5% | 31.3% |
| Pittsburgh, PA | Boston CDP | 7.9% | 31.3% |
| Pittsburgh, PA | Brackenridge borough | 7.4% | 31.3% |
| Pittsburgh, PA | Braddock Hills borough | 38.9% | 31.3% |
| Pittsburgh, PA | Braddock borough | 16.9% | 31.3% |
| Pittsburgh, PA | Bradenville CDP | 28.2% | 31.3% |
| Pittsburgh, PA | Bradford Woods borough | 77.3% | 31.3% |
| Pittsburgh, PA | Brentwood borough | 33.4% | 31.3% |
| Pittsburgh, PA | Bridgeville borough | 28.5% | 31.3% |
| Pittsburgh, PA | Bridgewater borough | 17.4% | 31.3% |
| Pittsburgh, PA | Bulger CDP | 61.5% | 31.3% |
| Pittsburgh, PA | Callery borough | 20.2% | 31.3% |
| Pittsburgh, PA | Calumet CDP | 38.4% | 31.3% |
| Pittsburgh, PA | Canonsburg borough | 22.3% | 31.3% |
| Pittsburgh, PA | Carnegie borough | 27.0% | 31.3% |
| Pittsburgh, PA | Carnot-Moon CDP | 49.2% | 31.3% |
| Pittsburgh, PA | Castle Shannon borough | 27.6% | 31.3% |
| Pittsburgh, PA | Cecil-Bishop CDP | 51.0% | 31.3% |
| Pittsburgh, PA | Chalfant borough | 32.6% | 31.3% |
| Pittsburgh, PA | Cheswick borough | 15.3% | 31.3% |
| Pittsburgh, PA | Churchill borough | 33.5% | 31.3% |
| Pittsburgh, PA | Clairton city | 14.6% | 31.3% |
| Pittsburgh, PA | Collinsburg CDP | 55.2% | 31.3% |
| Pittsburgh, PA | Conway borough | 10.7% | 31.3% |
| Pittsburgh, PA | Coraopolis borough | 5.9% | 31.3% |
| Pittsburgh, PA | Crafton borough | 39.9% | 31.3% |
| Pittsburgh, PA | Curtisville CDP | 26.6% | 31.3% |
| Pittsburgh, PA | Darlington borough | 6.5% | 31.3% |
| Pittsburgh, PA | Delmont borough | 28.6% | 31.3% |
| Pittsburgh, PA | Derry borough | 12.8% | 31.3% |
| Pittsburgh, PA | Dormont borough | 16.7% | 31.3% |
| Pittsburgh, PA | Dravosburg borough | 18.8% | 31.3% |
| Pittsburgh, PA | Duquesne city | 13.3% | 31.3% |
| Pittsburgh, PA | East McKeesport borough | 8.3% | 31.3% |
| Pittsburgh, PA | East Pittsburgh borough | 10.2% | 31.3% |
| Pittsburgh, PA | East Rochester borough | 34.6% | 31.3% |
| Pittsburgh, PA | East Vandergrift borough | 4.0% | 31.3% |
| Pittsburgh, PA | East Washington borough | 33.2% | 31.3% |
| Pittsburgh, PA | Eastvale borough | 4.7% | 31.3% |
| Pittsburgh, PA | Economy borough | 57.3% | 31.3% |
| Pittsburgh, PA | Edgewood borough | 41.6% | 31.3% |
| Pittsburgh, PA | Edgeworth borough | 50.4% | 31.3% |
| Pittsburgh, PA | Elizabeth borough | 15.0% | 31.3% |
| Pittsburgh, PA | Elrama CDP | 6.9% | 31.3% |
| Pittsburgh, PA | Emsworth borough | 33.7% | 31.3% |
| Pittsburgh, PA | Enlow CDP | 52.9% | 31.3% |
| Pittsburgh, PA | Etna borough | 9.9% | 31.3% |
| Pittsburgh, PA | Evans City borough | 15.9% | 31.3% |
| Pittsburgh, PA | Everson borough | 14.3% | 31.3% |
| Pittsburgh, PA | Export borough | 20.8% | 31.3% |
| Pittsburgh, PA | Fallston borough | 33.4% | 31.3% |
| Pittsburgh, PA | Finleyville borough | 8.6% | 31.3% |
| Pittsburgh, PA | Forest Hills borough | 40.3% | 31.3% |
| Pittsburgh, PA | Fox Chapel borough | 47.1% | 31.3% |
| Pittsburgh, PA | Franklin Park borough | 55.1% | 31.3% |
| Pittsburgh, PA | Freedom borough | 14.6% | 31.3% |
| Pittsburgh, PA | Freeport borough | 12.2% | 31.3% |
| Pittsburgh, PA | Gastonville CDP | 30.9% | 31.3% |
| Pittsburgh, PA | Gibsonia CDP | 56.9% | 31.3% |
| Pittsburgh, PA | Glassport borough | 7.1% | 31.3% |
| Pittsburgh, PA | Glen Osborne borough | 52.8% | 31.3% |
| Pittsburgh, PA | Glenfield borough | 26.2% | 31.3% |
| Pittsburgh, PA | Glenshaw CDP | 37.1% | 31.3% |
| Pittsburgh, PA | Grapeville CDP | 25.4% | 31.3% |
| Pittsburgh, PA | Green Tree borough | 50.6% | 31.3% |
| Pittsburgh, PA | Greenock CDP | 33.6% | 31.3% |
| Pittsburgh, PA | Greensburg city | 20.5% | 31.3% |
| Pittsburgh, PA | Harmony borough | 19.5% | 31.3% |
| Pittsburgh, PA | Harrison City CDP | 7.7% | 31.3% |
| Pittsburgh, PA | Harwick CDP | 15.9% | 31.3% |
| Pittsburgh, PA | Haysville borough | 86.8% | 31.3% |
| Pittsburgh, PA | Heidelberg borough | 11.4% | 31.3% |
| Pittsburgh, PA | Hendersonville CDP | 40.2% | 31.3% |
| Pittsburgh, PA | Herminie CDP | 18.3% | 31.3% |
| Pittsburgh, PA | Homestead borough | 14.8% | 31.3% |
| Pittsburgh, PA | Homewood borough | 27.9% | 31.3% |
| Pittsburgh, PA | Hostetter CDP | 24.5% | 31.3% |
| Pittsburgh, PA | Houston borough | 15.2% | 31.3% |
| Pittsburgh, PA | Hunker borough | 23.0% | 31.3% |
| Pittsburgh, PA | Hyde Park borough | 12.0% | 31.3% |
| Pittsburgh, PA | Imperial CDP | 53.3% | 31.3% |
| Pittsburgh, PA | Industry borough | 26.1% | 31.3% |
| Pittsburgh, PA | Ingram borough | 32.7% | 31.3% |
| Pittsburgh, PA | Irwin borough | 23.9% | 31.3% |
| Pittsburgh, PA | Jeannette city | 19.5% | 31.3% |
| Pittsburgh, PA | Jefferson Hills borough | 40.9% | 31.3% |
| Pittsburgh, PA | Joffre CDP | 36.6% | 31.3% |
| Pittsburgh, PA | Koppel borough | 3.7% | 31.3% |
| Pittsburgh, PA | Latrobe borough | 11.6% | 31.3% |
| Pittsburgh, PA | Lawrence CDP | 15.0% | 31.3% |
| Pittsburgh, PA | Lawson Heights CDP | 26.3% | 31.3% |
| Pittsburgh, PA | Leechburg borough | 12.9% | 31.3% |
| Pittsburgh, PA | Leetsdale borough | 23.8% | 31.3% |
| Pittsburgh, PA | Level Green CDP | 31.7% | 31.3% |
| Pittsburgh, PA | Liberty borough | 35.4% | 31.3% |
| Pittsburgh, PA | Lincoln borough | 42.8% | 31.3% |
| Pittsburgh, PA | Lower Burrell city | 31.1% | 31.3% |
| Pittsburgh, PA | Loyalhanna CDP | 23.5% | 31.3% |
| Pittsburgh, PA | Manor borough | 28.9% | 31.3% |
| Pittsburgh, PA | Mars borough | 21.0% | 31.3% |
| Pittsburgh, PA | McDonald borough | 18.3% | 31.3% |
| Pittsburgh, PA | McGovern CDP | 25.2% | 31.3% |
| Pittsburgh, PA | McKees Rocks borough | 13.6% | 31.3% |
| Pittsburgh, PA | McKeesport city | 20.4% | 31.3% |
| Pittsburgh, PA | McMurray CDP | 45.8% | 31.3% |
| Pittsburgh, PA | Meadowlands CDP | 14.8% | 31.3% |
| Pittsburgh, PA | Midway borough | 40.4% | 31.3% |
| Pittsburgh, PA | Millvale borough | 28.5% | 31.3% |
| Pittsburgh, PA | Millwood CDP | 29.1% | 31.3% |
| Pittsburgh, PA | Monaca borough | 18.2% | 31.3% |
| Pittsburgh, PA | Monroeville municipality | 36.3% | 31.3% |
| Pittsburgh, PA | Mount Oliver borough | 19.3% | 31.3% |
| Pittsburgh, PA | Mount Pleasant borough | 7.2% | 31.3% |
| Pittsburgh, PA | Munhall borough | 19.1% | 31.3% |
| Pittsburgh, PA | Murrysville municipality | 36.2% | 31.3% |
| Pittsburgh, PA | Muse CDP | 43.3% | 31.3% |
| Pittsburgh, PA | New Brighton borough | 7.1% | 31.3% |
| Pittsburgh, PA | New Kensington city | 23.6% | 31.3% |
| Pittsburgh, PA | New Stanton borough | 30.6% | 31.3% |
| Pittsburgh, PA | Noblestown CDP | 63.5% | 31.3% |
| Pittsburgh, PA | North Apollo borough | 14.5% | 31.3% |
| Pittsburgh, PA | North Braddock borough | 27.2% | 31.3% |
| Pittsburgh, PA | North Irwin borough | 12.4% | 31.3% |
| Pittsburgh, PA | North Vandergrift CDP | 7.3% | 31.3% |
| Pittsburgh, PA | Norvelt CDP | 32.9% | 31.3% |
| Pittsburgh, PA | Oakdale borough | 41.1% | 31.3% |
| Pittsburgh, PA | Oakmont borough | 31.4% | 31.3% |
| Pittsburgh, PA | Oklahoma borough | 28.8% | 31.3% |
| Pittsburgh, PA | Orchard Hills CDP | 22.1% | 31.3% |
| Pittsburgh, PA | Patterson Heights borough | 18.0% | 31.3% |
| Pittsburgh, PA | Penn borough | 13.6% | 31.3% |
| Pittsburgh, PA | Pennsbury Village borough | 42.8% | 31.3% |
| Pittsburgh, PA | Pitcairn borough | 14.2% | 31.3% |
| Pittsburgh, PA | Pittsburgh city | 27.6% | 31.3% |
| Pittsburgh, PA | Pleasant Hills borough | 38.6% | 31.3% |
| Pittsburgh, PA | Pleasant View CDP | 12.8% | 31.3% |
| Pittsburgh, PA | Plum borough | 32.4% | 31.3% |
| Pittsburgh, PA | Port Vue borough | 25.6% | 31.3% |
| Pittsburgh, PA | Rankin borough | 16.0% | 31.3% |
| Pittsburgh, PA | Rennerdale CDP | 35.3% | 31.3% |
| Pittsburgh, PA | Rochester borough | 12.1% | 31.3% |
| Pittsburgh, PA | Rosslyn Farms borough | 60.1% | 31.3% |
| Pittsburgh, PA | Russellton CDP | 42.3% | 31.3% |
| Pittsburgh, PA | Saxonburg borough | 20.3% | 31.3% |
| Pittsburgh, PA | Scottdale borough | 16.9% | 31.3% |
| Pittsburgh, PA | Seven Fields borough | 32.7% | 31.3% |
| Pittsburgh, PA | Sewickley Heights borough | 45.2% | 31.3% |
| Pittsburgh, PA | Sewickley Hills borough | 73.9% | 31.3% |
| Pittsburgh, PA | Sewickley borough | 27.1% | 31.3% |
| Pittsburgh, PA | Sharpsburg borough | 2.9% | 31.3% |
| Pittsburgh, PA | South Greensburg borough | 8.3% | 31.3% |
| Pittsburgh, PA | South Heights borough | 33.5% | 31.3% |
| Pittsburgh, PA | South Park Township CDP | 32.2% | 31.3% |
| Pittsburgh, PA | Southview CDP | 22.2% | 31.3% |
| Pittsburgh, PA | Southwest Greensburg borough | 13.3% | 31.3% |
| Pittsburgh, PA | Springdale borough | 10.7% | 31.3% |
| Pittsburgh, PA | St. Vincent College CDP | 43.7% | 31.3% |
| Pittsburgh, PA | Sturgeon CDP | 47.5% | 31.3% |
| Pittsburgh, PA | Sutersville borough | 29.1% | 31.3% |
| Pittsburgh, PA | Swissvale borough | 25.2% | 31.3% |
| Pittsburgh, PA | Tarentum borough | 9.6% | 31.3% |
| Pittsburgh, PA | Thompsonville CDP | 34.2% | 31.3% |
| Pittsburgh, PA | Thornburg borough | 64.7% | 31.3% |
| Pittsburgh, PA | Trafford borough | 20.8% | 31.3% |
| Pittsburgh, PA | Turtle Creek borough | 21.7% | 31.3% |
| Pittsburgh, PA | Upper St. Clair CDP | 46.9% | 31.3% |
| Pittsburgh, PA | Valencia borough | 15.2% | 31.3% |
| Pittsburgh, PA | Vandergrift borough | 7.9% | 31.3% |
| Pittsburgh, PA | Verona borough | 15.7% | 31.3% |
| Pittsburgh, PA | Versailles borough | 9.9% | 31.3% |
| Pittsburgh, PA | Wall borough | 51.0% | 31.3% |
| Pittsburgh, PA | Washington city | 12.0% | 31.3% |
| Pittsburgh, PA | West Elizabeth borough | 9.0% | 31.3% |
| Pittsburgh, PA | West Homestead borough | 24.4% | 31.3% |
| Pittsburgh, PA | West Leechburg borough | 19.6% | 31.3% |
| Pittsburgh, PA | West Mayfield borough | 17.1% | 31.3% |
| Pittsburgh, PA | West Mifflin borough | 29.0% | 31.3% |
| Pittsburgh, PA | West Newton borough | 17.1% | 31.3% |
| Pittsburgh, PA | West View borough | 23.8% | 31.3% |
| Pittsburgh, PA | Whitaker borough | 16.6% | 31.3% |
| Pittsburgh, PA | White Oak borough | 26.4% | 31.3% |
| Pittsburgh, PA | Whitehall borough | 29.6% | 31.3% |
| Pittsburgh, PA | Wilkinsburg borough | 28.9% | 31.3% |
| Pittsburgh, PA | Wilmerding borough | 13.5% | 31.3% |
| Pittsburgh, PA | Wolfdale CDP | 41.3% | 31.3% |
| Pittsburgh, PA | Youngstown borough | 10.6% | 31.3% |
| Pittsburgh, PA | Youngwood borough | 14.1% | 31.3% |
| Pittsburgh, PA | Zelienople borough | 14.6% | 31.3% |
| Port St. Lucie, FL | Fort Pierce North CDP | 37.3% | 41.2% |
| Port St. Lucie, FL | Fort Pierce South CDP | 34.7% | 41.2% |
| Port St. Lucie, FL | Fort Pierce city | 30.3% | 41.2% |
| Port St. Lucie, FL | Hobe Sound CDP | 41.6% | 41.2% |
| Port St. Lucie, FL | Hutchinson Island South CDP | 19.0% | 41.2% |
| Port St. Lucie, FL | Indian River Estates CDP | 49.3% | 41.2% |
| Port St. Lucie, FL | Jensen Beach CDP | 45.5% | 41.2% |
| Port St. Lucie, FL | North River Shores CDP | 46.4% | 41.2% |
| Port St. Lucie, FL | Ocean Breeze town | 0.2% | 41.2% |
| Port St. Lucie, FL | Palm City CDP | 44.2% | 41.2% |
| Port St. Lucie, FL | Port Salerno CDP | 39.1% | 41.2% |
| Port St. Lucie, FL | Port St. Lucie city | 41.1% | 41.2% |
| Port St. Lucie, FL | Rio CDP | 54.6% | 41.2% |
| Port St. Lucie, FL | River Park CDP | 27.8% | 41.2% |
| Port St. Lucie, FL | Sewall's Point town | 60.6% | 41.2% |
| Port St. Lucie, FL | St. Lucie Village town | 63.4% | 41.2% |
| Port St. Lucie, FL | Stuart city | 36.3% | 41.2% |
| Port St. Lucie, FL | White City CDP | 59.9% | 41.2% |
| Portland, OR--WA | Aloha CDP | 27.8% | 26.8% |
| Portland, OR--WA | Barberton CDP | 37.8% | 26.8% |
| Portland, OR--WA | Battle Ground city | 13.9% | 26.8% |
| Portland, OR--WA | Beavercreek CDP | 69.1% | 26.8% |
| Portland, OR--WA | Beaverton city | 36.1% | 26.8% |
| Portland, OR--WA | Bethany CDP | 21.0% | 26.8% |
| Portland, OR--WA | Brush Prairie CDP | 28.3% | 26.8% |
| Portland, OR--WA | Bull Mountain CDP | 34.4% | 26.8% |
| Portland, OR--WA | Butteville CDP | 59.1% | 26.8% |
| Portland, OR--WA | Camas city | 37.2% | 26.8% |
| Portland, OR--WA | Cedar Hills CDP | 37.3% | 26.8% |
| Portland, OR--WA | Cedar Mill CDP | 44.2% | 26.8% |
| Portland, OR--WA | Cherry Grove CDP | 30.6% | 26.8% |
| Portland, OR--WA | Cornelius city | 6.3% | 26.8% |
| Portland, OR--WA | Damascus CDP | 34.7% | 26.8% |
| Portland, OR--WA | Dollars Corner CDP | 32.6% | 26.8% |
| Portland, OR--WA | Duluth CDP | 29.1% | 26.8% |
| Portland, OR--WA | Durham city | 53.3% | 26.8% |
| Portland, OR--WA | Fairview city | 23.9% | 26.8% |
| Portland, OR--WA | Felida CDP | 29.6% | 26.8% |
| Portland, OR--WA | Fern Prairie CDP | 70.9% | 26.8% |
| Portland, OR--WA | Five Corners CDP | 16.6% | 26.8% |
| Portland, OR--WA | Forest Grove city | 14.6% | 26.8% |
| Portland, OR--WA | Garden Home-Whitford CDP | 52.3% | 26.8% |
| Portland, OR--WA | Gladstone city | 36.2% | 26.8% |
| Portland, OR--WA | Gresham city | 16.2% | 26.8% |
| Portland, OR--WA | Happy Valley city | 44.3% | 26.8% |
| Portland, OR--WA | Hazel Dell CDP | 24.1% | 26.8% |
| Portland, OR--WA | Hillsboro city | 18.0% | 26.8% |
| Portland, OR--WA | Hockinson CDP | 22.1% | 26.8% |
| Portland, OR--WA | Jennings Lodge CDP | 35.2% | 26.8% |
| Portland, OR--WA | Johnson City city | 8.1% | 26.8% |
| Portland, OR--WA | King City city | 22.5% | 26.8% |
| Portland, OR--WA | Lake Oswego city | 60.2% | 26.8% |
| Portland, OR--WA | Lake Shore CDP | 25.0% | 26.8% |
| Portland, OR--WA | Lewisville CDP | 63.0% | 26.8% |
| Portland, OR--WA | Maywood Park city | 54.5% | 26.8% |
| Portland, OR--WA | Meadow Glade CDP | 36.2% | 26.8% |
| Portland, OR--WA | Metzger CDP | 39.1% | 26.8% |
| Portland, OR--WA | Milwaukie city | 32.2% | 26.8% |
| Portland, OR--WA | Minnehaha CDP | 22.8% | 26.8% |
| Portland, OR--WA | Mount Vista CDP | 31.5% | 26.8% |
| Portland, OR--WA | Oak Grove CDP | 36.1% | 26.8% |
| Portland, OR--WA | Oak Hills CDP | 21.8% | 26.8% |
| Portland, OR--WA | Oatfield CDP | 36.2% | 26.8% |
| Portland, OR--WA | Orchards CDP | 11.9% | 26.8% |
| Portland, OR--WA | Oregon City city | 34.8% | 26.8% |
| Portland, OR--WA | Portland city | 25.3% | 26.8% |
| Portland, OR--WA | Raleigh Hills CDP | 42.8% | 26.8% |
| Portland, OR--WA | Rivergrove city | 51.6% | 26.8% |
| Portland, OR--WA | Rockcreek CDP | 24.4% | 26.8% |
| Portland, OR--WA | Salmon Creek CDP | 31.3% | 26.8% |
| Portland, OR--WA | Sherwood city | 34.5% | 26.8% |
| Portland, OR--WA | Stafford CDP | 71.3% | 26.8% |
| Portland, OR--WA | Tigard city | 38.3% | 26.8% |
| Portland, OR--WA | Troutdale city | 22.8% | 26.8% |
| Portland, OR--WA | Tualatin city | 33.0% | 26.8% |
| Portland, OR--WA | Vancouver city | 23.5% | 26.8% |
| Portland, OR--WA | Venersborg CDP | 16.2% | 26.8% |
| Portland, OR--WA | Washougal city | 21.4% | 26.8% |
| Portland, OR--WA | West Haven-Sylvan CDP | 58.1% | 26.8% |
| Portland, OR--WA | West Linn city | 55.5% | 26.8% |
| Portland, OR--WA | West Slope CDP | 51.9% | 26.8% |
| Portland, OR--WA | Wilsonville city | 40.8% | 26.8% |
| Portland, OR--WA | Wood Village city | 12.3% | 26.8% |
| Poughkeepsie--Newburgh, NY--NJ | Arlington CDP | 33.4% | 44.6% |
| Poughkeepsie--Newburgh, NY--NJ | Balmville CDP | 44.9% | 44.6% |
| Poughkeepsie--Newburgh, NY--NJ | Beacon city | 28.2% | 44.6% |
| Poughkeepsie--Newburgh, NY--NJ | Beaver Dam Lake CDP | 53.8% | 44.6% |
| Poughkeepsie--Newburgh, NY--NJ | Brinckerhoff CDP | 42.5% | 44.6% |
| Poughkeepsie--Newburgh, NY--NJ | Clintondale CDP | 50.5% | 44.6% |
| Poughkeepsie--Newburgh, NY--NJ | Cornwall-on-Hudson village | 37.1% | 44.6% |
| Poughkeepsie--Newburgh, NY--NJ | Crown Heights CDP | 37.7% | 44.6% |
| Poughkeepsie--Newburgh, NY--NJ | Fairview CDP | 51.5% | 44.6% |
| Poughkeepsie--Newburgh, NY--NJ | Firthcliffe CDP | 39.5% | 44.6% |
| Poughkeepsie--Newburgh, NY--NJ | Fishkill village | 34.3% | 44.6% |
| Poughkeepsie--Newburgh, NY--NJ | Freedom Plains CDP | 14.3% | 44.6% |
| Poughkeepsie--Newburgh, NY--NJ | Gardnertown CDP | 48.7% | 44.6% |
| Poughkeepsie--Newburgh, NY--NJ | Greenwood Lake village | 31.4% | 44.6% |
| Poughkeepsie--Newburgh, NY--NJ | Harriman village | 27.9% | 44.6% |
| Poughkeepsie--Newburgh, NY--NJ | Haviland CDP | 66.1% | 44.6% |
| Poughkeepsie--Newburgh, NY--NJ | Highland CDP | 66.2% | 44.6% |
| Poughkeepsie--Newburgh, NY--NJ | Hillside Lake CDP | 54.7% | 44.6% |
| Poughkeepsie--Newburgh, NY--NJ | Hopewell Junction CDP | 27.7% | 44.6% |
| Poughkeepsie--Newburgh, NY--NJ | Hyde Park CDP | 59.4% | 44.6% |
| Poughkeepsie--Newburgh, NY--NJ | Kiryas Joel village | 23.7% | 44.6% |
| Poughkeepsie--Newburgh, NY--NJ | Marlboro CDP | 63.4% | 44.6% |
| Poughkeepsie--Newburgh, NY--NJ | Merritt Park CDP | 30.5% | 44.6% |
| Poughkeepsie--Newburgh, NY--NJ | Milton CDP | 59.0% | 44.6% |
| Poughkeepsie--Newburgh, NY--NJ | Monroe village | 40.0% | 44.6% |
| Poughkeepsie--Newburgh, NY--NJ | Mountain Lodge Park CDP | 61.6% | 44.6% |
| Poughkeepsie--Newburgh, NY--NJ | Myers Corner CDP | 59.6% | 44.6% |
| Poughkeepsie--Newburgh, NY--NJ | New Paltz village | 29.3% | 44.6% |
| Poughkeepsie--Newburgh, NY--NJ | New Windsor CDP | 27.2% | 44.6% |
| Poughkeepsie--Newburgh, NY--NJ | Newburgh city | 15.3% | 44.6% |
| Poughkeepsie--Newburgh, NY--NJ | Orange Lake CDP | 38.4% | 44.6% |
| Poughkeepsie--Newburgh, NY--NJ | Pawling village | 37.0% | 44.6% |
| Poughkeepsie--Newburgh, NY--NJ | Plattekill CDP | 65.7% | 44.6% |
| Poughkeepsie--Newburgh, NY--NJ | Pleasant Valley CDP | 67.7% | 44.6% |
| Poughkeepsie--Newburgh, NY--NJ | Poughkeepsie city | 22.7% | 44.6% |
| Poughkeepsie--Newburgh, NY--NJ | Red Oaks Mill CDP | 54.9% | 44.6% |
| Poughkeepsie--Newburgh, NY--NJ | Salisbury Mills CDP | 59.4% | 44.6% |
| Poughkeepsie--Newburgh, NY--NJ | South Blooming Grove village | 44.9% | 44.6% |
| Poughkeepsie--Newburgh, NY--NJ | Spackenkill CDP | 53.5% | 44.6% |
| Poughkeepsie--Newburgh, NY--NJ | Staatsburg CDP | 48.5% | 44.6% |
| Poughkeepsie--Newburgh, NY--NJ | Titusville CDP | 51.7% | 44.6% |
| Poughkeepsie--Newburgh, NY--NJ | Vails Gate CDP | 19.1% | 44.6% |
| Poughkeepsie--Newburgh, NY--NJ | Wallkill CDP | 43.9% | 44.6% |
| Poughkeepsie--Newburgh, NY--NJ | Walton Park CDP | 41.4% | 44.6% |
| Poughkeepsie--Newburgh, NY--NJ | Wappingers Falls village | 24.1% | 44.6% |
| Poughkeepsie--Newburgh, NY--NJ | Washingtonville village | 34.8% | 44.6% |
| Poughkeepsie--Newburgh, NY--NJ | Woodbury village | 48.6% | 44.6% |
| Providence, RI--MA | Attleboro city | 52.8% | 29.3% |
| Providence, RI--MA | Bellingham CDP | 66.2% | 29.3% |
| Providence, RI--MA | Central Falls city | 23.6% | 29.3% |
| Providence, RI--MA | Cranston city | 25.0% | 29.3% |
| Providence, RI--MA | Cumberland Hill CDP | 43.6% | 29.3% |
| Providence, RI--MA | East Providence city | 9.2% | 29.3% |
| Providence, RI--MA | Fall River city | 5.8% | 29.3% |
| Providence, RI--MA | Greenville CDP | 54.6% | 29.3% |
| Providence, RI--MA | Harmony CDP | 45.3% | 29.3% |
| Providence, RI--MA | Harrisville CDP | 45.7% | 29.3% |
| Providence, RI--MA | Kingston CDP | 31.2% | 29.3% |
| Providence, RI--MA | Melville CDP | 13.1% | 29.3% |
| Providence, RI--MA | Narragansett Pier CDP | 36.8% | 29.3% |
| Providence, RI--MA | Newport East CDP | 16.0% | 29.3% |
| Providence, RI--MA | Newport city | 1.9% | 29.3% |
| Providence, RI--MA | North Seekonk CDP | 47.6% | 29.3% |
| Providence, RI--MA | North Westport CDP | 69.2% | 29.3% |
| Providence, RI--MA | Ocean Grove CDP | 12.9% | 29.3% |
| Providence, RI--MA | Pascoag CDP | 49.5% | 29.3% |
| Providence, RI--MA | Pawtucket city | 15.7% | 29.3% |
| Providence, RI--MA | Providence city | 23.0% | 29.3% |
| Providence, RI--MA | Somerset CDP | 30.1% | 29.3% |
| Providence, RI--MA | Tiverton CDP | 24.5% | 29.3% |
| Providence, RI--MA | Valley Falls CDP | 24.1% | 29.3% |
| Providence, RI--MA | Wakefield-Peacedale CDP | 61.0% | 29.3% |
| Providence, RI--MA | Warwick city | 32.2% | 29.3% |
| Providence, RI--MA | Woonsocket city | 16.9% | 29.3% |
| Raleigh, NC | Angier town | 44.7% | 56.7% |
| Raleigh, NC | Apex town | 52.1% | 56.7% |
| Raleigh, NC | Cary town | 63.4% | 56.7% |
| Raleigh, NC | Clayton town | 48.4% | 56.7% |
| Raleigh, NC | Fuquay-Varina town | 51.1% | 56.7% |
| Raleigh, NC | Garner town | 59.1% | 56.7% |
| Raleigh, NC | Holly Springs town | 56.9% | 56.7% |
| Raleigh, NC | Knightdale town | 47.7% | 56.7% |
| Raleigh, NC | Morrisville town | 37.2% | 56.7% |
| Raleigh, NC | Raleigh city | 52.3% | 56.7% |
| Raleigh, NC | Rolesville town | 42.0% | 56.7% |
| Raleigh, NC | Wake Forest town | 49.1% | 56.7% |
| Raleigh, NC | Wendell town | 8.5% | 56.7% |
| Raleigh, NC | Youngsville town | 49.5% | 56.7% |
| Richmond, VA | Ashland town | 53.7% | 52.2% |
| Richmond, VA | Bellwood CDP | 57.6% | 52.2% |
| Richmond, VA | Bensley CDP | 59.7% | 52.2% |
| Richmond, VA | Bon Air CDP | 71.6% | 52.2% |
| Richmond, VA | Brandermill CDP | 68.5% | 52.2% |
| Richmond, VA | Chamberlayne CDP | 51.1% | 52.2% |
| Richmond, VA | Chester CDP | 67.1% | 52.2% |
| Richmond, VA | Colonial Heights city | 37.5% | 52.2% |
| Richmond, VA | Dumbarton CDP | 36.6% | 52.2% |
| Richmond, VA | East Highland Park CDP | 39.6% | 52.2% |
| Richmond, VA | Enon CDP | 61.8% | 52.2% |
| Richmond, VA | Ettrick CDP | 45.8% | 52.2% |
| Richmond, VA | Fort Lee CDP | 25.0% | 52.2% |
| Richmond, VA | Glen Allen CDP | 55.9% | 52.2% |
| Richmond, VA | Highland Springs CDP | 40.3% | 52.2% |
| Richmond, VA | Hopewell city | 50.6% | 52.2% |
| Richmond, VA | Innsbrook CDP | 42.7% | 52.2% |
| Richmond, VA | Lakeside CDP | 51.3% | 52.2% |
| Richmond, VA | Laurel CDP | 38.4% | 52.2% |
| Richmond, VA | Manchester CDP | 59.8% | 52.2% |
| Richmond, VA | Matoaca CDP | 71.3% | 52.2% |
| Richmond, VA | Meadowbrook CDP | 61.9% | 52.2% |
| Richmond, VA | Mechanicsville CDP | 49.6% | 52.2% |
| Richmond, VA | Montrose CDP | 39.6% | 52.2% |
| Richmond, VA | Petersburg city | 46.1% | 52.2% |
| Richmond, VA | Richmond city | 39.6% | 52.2% |
| Richmond, VA | Rockwood CDP | 70.4% | 52.2% |
| Richmond, VA | Sandston CDP | 42.9% | 52.2% |
| Richmond, VA | Short Pump CDP | 36.4% | 52.2% |
| Richmond, VA | Tuckahoe CDP | 59.2% | 52.2% |
| Richmond, VA | Woodlake CDP | 70.7% | 52.2% |
| Richmond, VA | Wyndham CDP | 40.3% | 52.2% |
| Riverside--San Bernardino, CA | Banning city | 22.8% | 23.9% |
| Riverside--San Bernardino, CA | Beaumont city | 30.7% | 23.9% |
| Riverside--San Bernardino, CA | Bloomington CDP | 18.7% | 23.9% |
| Riverside--San Bernardino, CA | Calimesa city | 40.5% | 23.9% |
| Riverside--San Bernardino, CA | Cherry Valley CDP | 36.8% | 23.9% |
| Riverside--San Bernardino, CA | Colton city | 20.9% | 23.9% |
| Riverside--San Bernardino, CA | Corona city | 28.3% | 23.9% |
| Riverside--San Bernardino, CA | Coronita CDP | 21.6% | 23.9% |
| Riverside--San Bernardino, CA | Eastvale city | 20.8% | 23.9% |
| Riverside--San Bernardino, CA | El Cerrito CDP | 26.8% | 23.9% |
| Riverside--San Bernardino, CA | El Sobrante CDP | 23.1% | 23.9% |
| Riverside--San Bernardino, CA | Fontana city | 18.1% | 23.9% |
| Riverside--San Bernardino, CA | Good Hope CDP | 18.7% | 23.9% |
| Riverside--San Bernardino, CA | Grand Terrace city | 28.2% | 23.9% |
| Riverside--San Bernardino, CA | Highgrove CDP | 19.0% | 23.9% |
| Riverside--San Bernardino, CA | Highland city | 26.4% | 23.9% |
| Riverside--San Bernardino, CA | Home Gardens CDP | 23.1% | 23.9% |
| Riverside--San Bernardino, CA | Jurupa Valley city | 18.3% | 23.9% |
| Riverside--San Bernardino, CA | Lake Mathews CDP | 24.2% | 23.9% |
| Riverside--San Bernardino, CA | Loma Linda city | 31.6% | 23.9% |
| Riverside--San Bernardino, CA | March ARB CDP | 25.4% | 23.9% |
| Riverside--San Bernardino, CA | Mead Valley CDP | 17.4% | 23.9% |
| Riverside--San Bernardino, CA | Meadowbrook CDP | 3.5% | 23.9% |
| Riverside--San Bernardino, CA | Mentone CDP | 29.1% | 23.9% |
| Riverside--San Bernardino, CA | Moreno Valley city | 24.4% | 23.9% |
| Riverside--San Bernardino, CA | Muscoy CDP | 24.1% | 23.9% |
| Riverside--San Bernardino, CA | Norco city | 19.9% | 23.9% |
| Riverside--San Bernardino, CA | Perris city | 20.5% | 23.9% |
| Riverside--San Bernardino, CA | Redlands city | 39.1% | 23.9% |
| Riverside--San Bernardino, CA | Rialto city | 20.6% | 23.9% |
| Riverside--San Bernardino, CA | Riverside city | 26.8% | 23.9% |
| Riverside--San Bernardino, CA | San Bernardino city | 25.9% | 23.9% |
| Riverside--San Bernardino, CA | Temescal Valley CDP | 23.6% | 23.9% |
| Riverside--San Bernardino, CA | Woodcrest CDP | 24.3% | 23.9% |
| Riverside--San Bernardino, CA | Yucaipa city | 41.2% | 23.9% |
| Rochester, NY | Brighton CDP | 27.6% | 24.6% |
| Rochester, NY | Canandaigua city | 37.7% | 24.6% |
| Rochester, NY | Crystal Beach CDP | 52.2% | 24.6% |
| Rochester, NY | East Rochester village | 14.3% | 24.6% |
| Rochester, NY | Fairport village | 30.1% | 24.6% |
| Rochester, NY | Gates CDP | 20.6% | 24.6% |
| Rochester, NY | Greece CDP | 22.3% | 24.6% |
| Rochester, NY | Hilton village | 23.1% | 24.6% |
| Rochester, NY | Irondequoit CDP | 19.0% | 24.6% |
| Rochester, NY | Macedon CDP | 61.1% | 24.6% |
| Rochester, NY | North Gates CDP | 22.6% | 24.6% |
| Rochester, NY | Ontario CDP | 62.2% | 24.6% |
| Rochester, NY | Pittsford village | 31.8% | 24.6% |
| Rochester, NY | Rochester city | 15.0% | 24.6% |
| Rochester, NY | Scottsville village | 21.6% | 24.6% |
| Rochester, NY | Spencerport village | 38.3% | 24.6% |
| Rochester, NY | Victor village | 40.9% | 24.6% |
| Rochester, NY | Webster village | 29.2% | 24.6% |
| Sacramento, CA | Antelope CDP | 20.5% | 22.9% |
| Sacramento, CA | Arden-Arcade CDP | 31.4% | 22.9% |
| Sacramento, CA | Cameron Park CDP | 25.6% | 22.9% |
| Sacramento, CA | Carmichael CDP | 34.9% | 22.9% |
| Sacramento, CA | Citrus Heights city | 30.6% | 22.9% |
| Sacramento, CA | Diamond Springs CDP | 16.7% | 22.9% |
| Sacramento, CA | El Dorado Hills CDP | 21.4% | 22.9% |
| Sacramento, CA | Elk Grove city | 20.3% | 22.9% |
| Sacramento, CA | Elverta CDP | 22.9% | 22.9% |
| Sacramento, CA | Fair Oaks CDP | 44.3% | 22.9% |
| Sacramento, CA | Florin CDP | 17.9% | 22.9% |
| Sacramento, CA | Folsom city | 17.9% | 22.9% |
| Sacramento, CA | Foothill Farms CDP | 27.3% | 22.9% |
| Sacramento, CA | Franklin CDP | 28.3% | 22.9% |
| Sacramento, CA | Freeport CDP | 32.2% | 22.9% |
| Sacramento, CA | Fruitridge Pocket CDP | 21.7% | 22.9% |
| Sacramento, CA | Gold River CDP | 43.3% | 22.9% |
| Sacramento, CA | Granite Bay CDP | 41.3% | 22.9% |
| Sacramento, CA | La Riviera CDP | 31.7% | 22.9% |
| Sacramento, CA | Lemon Hill CDP | 19.4% | 22.9% |
| Sacramento, CA | Lincoln city | 15.3% | 22.9% |
| Sacramento, CA | Loomis town | 31.0% | 22.9% |
| Sacramento, CA | Mather CDP | 18.1% | 22.9% |
| Sacramento, CA | McClellan Park CDP | 11.3% | 22.9% |
| Sacramento, CA | Newcastle CDP | 20.9% | 22.9% |
| Sacramento, CA | North Highlands CDP | 17.3% | 22.9% |
| Sacramento, CA | Orangevale CDP | 31.7% | 22.9% |
| Sacramento, CA | Parkway CDP | 13.4% | 22.9% |
| Sacramento, CA | Penryn CDP | 41.5% | 22.9% |
| Sacramento, CA | Rancho Cordova city | 22.7% | 22.9% |
| Sacramento, CA | Rio Linda CDP | 17.4% | 22.9% |
| Sacramento, CA | Rocklin city | 19.7% | 22.9% |
| Sacramento, CA | Rosemont CDP | 23.4% | 22.9% |
| Sacramento, CA | Roseville city | 19.7% | 22.9% |
| Sacramento, CA | Sacramento city | 24.4% | 22.9% |
| Sacramento, CA | Shingle Springs CDP | 39.4% | 22.9% |
| Sacramento, CA | Vineyard CDP | 14.2% | 22.9% |
| Sacramento, CA | West Sacramento city | 22.3% | 22.9% |
| Salt Lake City--West Valley City, UT | Bluffdale city | 18.9% | 20.9% |
| Salt Lake City--West Valley City, UT | Cottonwood Heights city | 17.1% | 20.9% |
| Salt Lake City--West Valley City, UT | Draper city | 19.3% | 20.9% |
| Salt Lake City--West Valley City, UT | Granite CDP | 21.7% | 20.9% |
| Salt Lake City--West Valley City, UT | Herriman city | 16.8% | 20.9% |
| Salt Lake City--West Valley City, UT | Holladay city | 22.4% | 20.9% |
| Salt Lake City--West Valley City, UT | Kearns CDP | 18.5% | 20.9% |
| Salt Lake City--West Valley City, UT | Magna CDP | 20.8% | 20.9% |
| Salt Lake City--West Valley City, UT | Midvale city | 23.2% | 20.9% |
| Salt Lake City--West Valley City, UT | Millcreek city | 18.7% | 20.9% |
| Salt Lake City--West Valley City, UT | Murray city | 25.4% | 20.9% |
| Salt Lake City--West Valley City, UT | Riverton city | 20.6% | 20.9% |
| Salt Lake City--West Valley City, UT | Salt Lake City city | 22.4% | 20.9% |
| Salt Lake City--West Valley City, UT | Sandy city | 21.7% | 20.9% |
| Salt Lake City--West Valley City, UT | South Jordan city | 21.5% | 20.9% |
| Salt Lake City--West Valley City, UT | South Salt Lake city | 19.0% | 20.9% |
| Salt Lake City--West Valley City, UT | Taylorsville city | 24.7% | 20.9% |
| Salt Lake City--West Valley City, UT | West Jordan city | 21.8% | 20.9% |
| Salt Lake City--West Valley City, UT | West Valley City city | 19.6% | 20.9% |
| Salt Lake City--West Valley City, UT | White City CDP | 19.6% | 20.9% |
| San Antonio, TX | Alamo Heights city | 54.9% | 29.6% |
| San Antonio, TX | Balcones Heights city | 17.5% | 29.6% |
| San Antonio, TX | Bulverde city | 59.2% | 29.6% |
| San Antonio, TX | Castle Hills city | 43.0% | 29.6% |
| San Antonio, TX | China Grove town | 33.2% | 29.6% |
| San Antonio, TX | Cibolo city | 21.0% | 29.6% |
| San Antonio, TX | Converse city | 25.9% | 29.6% |
| San Antonio, TX | Cross Mountain CDP | 59.7% | 29.6% |
| San Antonio, TX | Fair Oaks Ranch city | 42.0% | 29.6% |
| San Antonio, TX | Garden Ridge city | 64.4% | 29.6% |
| San Antonio, TX | Helotes city | 47.0% | 29.6% |
| San Antonio, TX | Hill Country Village city | 74.6% | 29.6% |
| San Antonio, TX | Hollywood Park town | 61.4% | 29.6% |
| San Antonio, TX | Kirby city | 29.3% | 29.6% |
| San Antonio, TX | Lackland AFB CDP | 8.4% | 29.6% |
| San Antonio, TX | Lake Dunlap CDP | 36.8% | 29.6% |
| San Antonio, TX | Leon Valley city | 34.0% | 29.6% |
| San Antonio, TX | Live Oak city | 30.5% | 29.6% |
| San Antonio, TX | Marion city | 20.3% | 29.6% |
| San Antonio, TX | McQueeney CDP | 37.7% | 29.6% |
| San Antonio, TX | New Braunfels city | 25.0% | 29.6% |
| San Antonio, TX | Olmos Park city | 46.3% | 29.6% |
| San Antonio, TX | Randolph AFB CDP | 39.0% | 29.6% |
| San Antonio, TX | San Antonio city | 29.9% | 29.6% |
| San Antonio, TX | Santa Clara city | 12.8% | 29.6% |
| San Antonio, TX | Scenic Oaks CDP | 51.8% | 29.6% |
| San Antonio, TX | Schertz city | 33.6% | 29.6% |
| San Antonio, TX | Selma city | 20.7% | 29.6% |
| San Antonio, TX | Shavano Park city | 66.5% | 29.6% |
| San Antonio, TX | Terrell Hills city | 53.9% | 29.6% |
| San Antonio, TX | Timberwood Park CDP | 41.1% | 29.6% |
| San Antonio, TX | Universal City city | 30.8% | 29.6% |
| San Antonio, TX | Von Ormy city | 35.7% | 29.6% |
| San Antonio, TX | Windcrest city | 32.3% | 29.6% |
| San Diego, CA | Alpine CDP | 18.7% | 5.9% |
| San Diego, CA | Bonita CDP | 3.8% | 5.9% |
| San Diego, CA | Bonsall CDP | 11.7% | 5.9% |
| San Diego, CA | Bostonia CDP | 1.3% | 5.9% |
| San Diego, CA | Camp Pendleton North CDP | 9.1% | 5.9% |
| San Diego, CA | Camp Pendleton South CDP | 5.6% | 5.9% |
| San Diego, CA | Carlsbad city | 16.8% | 5.9% |
| San Diego, CA | Casa de Oro-Mount Helix CDP | 8.3% | 5.9% |
| San Diego, CA | Chula Vista city | 1.7% | 5.9% |
| San Diego, CA | Coronado city | 3.4% | 5.9% |
| San Diego, CA | Crest CDP | 15.3% | 5.9% |
| San Diego, CA | Del Mar city | 9.9% | 5.9% |
| San Diego, CA | El Cajon city | 1.0% | 5.9% |
| San Diego, CA | Encinitas city | 13.3% | 5.9% |
| San Diego, CA | Escondido city | 11.4% | 5.9% |
| San Diego, CA | Eucalyptus Hills CDP | 6.9% | 5.9% |
| San Diego, CA | Fairbanks Ranch CDP | 21.0% | 5.9% |
| San Diego, CA | Fallbrook CDP | 24.8% | 5.9% |
| San Diego, CA | Granite Hills CDP | 4.3% | 5.9% |
| San Diego, CA | Harbison Canyon CDP | 14.0% | 5.9% |
| San Diego, CA | Hidden Meadows CDP | 44.2% | 5.9% |
| San Diego, CA | Imperial Beach city | 3.9% | 5.9% |
| San Diego, CA | Jamul CDP | 6.5% | 5.9% |
| San Diego, CA | La Mesa city | 5.4% | 5.9% |
| San Diego, CA | La Presa CDP | 1.3% | 5.9% |
| San Diego, CA | Lake San Marcos CDP | 11.5% | 5.9% |
| San Diego, CA | Lakeside CDP | 2.0% | 5.9% |
| San Diego, CA | Lemon Grove city | 3.9% | 5.9% |
| San Diego, CA | National City city | 3.2% | 5.9% |
| San Diego, CA | Oceanside city | 10.3% | 5.9% |
| San Diego, CA | Poway city | 10.0% | 5.9% |
| San Diego, CA | Rancho San Diego CDP | 2.2% | 5.9% |
| San Diego, CA | Rancho Santa Fe CDP | 24.5% | 5.9% |
| San Diego, CA | San Diego city | 5.8% | 5.9% |
| San Diego, CA | San Marcos city | 6.6% | 5.9% |
| San Diego, CA | Santee city | 1.5% | 5.9% |
| San Diego, CA | Solana Beach city | 17.9% | 5.9% |
| San Diego, CA | Spring Valley CDP | 2.0% | 5.9% |
| San Diego, CA | Vista city | 12.6% | 5.9% |
| San Diego, CA | Winter Gardens CDP | 2.5% | 5.9% |
| San Francisco--Oakland, CA | Alameda city | 5.3% | 4.7% |
| San Francisco--Oakland, CA | Albany city | 6.6% | 4.7% |
| San Francisco--Oakland, CA | Alto CDP | 36.7% | 4.7% |
| San Francisco--Oakland, CA | Ashland CDP | 1.7% | 4.7% |
| San Francisco--Oakland, CA | Atherton town | 53.7% | 4.7% |
| San Francisco--Oakland, CA | Bayview CDP | 4.1% | 4.7% |
| San Francisco--Oakland, CA | Belmont city | 23.5% | 4.7% |
| San Francisco--Oakland, CA | Belvedere city | 42.7% | 4.7% |
| San Francisco--Oakland, CA | Berkeley city | 10.4% | 4.7% |
| San Francisco--Oakland, CA | Black Point-Green Point CDP | 54.5% | 4.7% |
| San Francisco--Oakland, CA | Brisbane city | 14.9% | 4.7% |
| San Francisco--Oakland, CA | Broadmoor CDP | 4.8% | 4.7% |
| San Francisco--Oakland, CA | Burlingame city | 10.6% | 4.7% |
| San Francisco--Oakland, CA | Castro Valley CDP | 6.7% | 4.7% |
| San Francisco--Oakland, CA | Cherryland CDP | 2.1% | 4.7% |
| San Francisco--Oakland, CA | Colma town | 1.7% | 4.7% |
| San Francisco--Oakland, CA | Corte Madera town | 29.0% | 4.7% |
| San Francisco--Oakland, CA | Crockett CDP | 17.7% | 4.7% |
| San Francisco--Oakland, CA | Daly City city | 1.9% | 4.7% |
| San Francisco--Oakland, CA | East Palo Alto city | 5.3% | 4.7% |
| San Francisco--Oakland, CA | East Richmond Heights CDP | 12.6% | 4.7% |
| San Francisco--Oakland, CA | El Cerrito city | 7.9% | 4.7% |
| San Francisco--Oakland, CA | El Sobrante CDP | 22.5% | 4.7% |
| San Francisco--Oakland, CA | Emerald Lake Hills CDP | 38.0% | 4.7% |
| San Francisco--Oakland, CA | Emeryville city | 1.7% | 4.7% |
| San Francisco--Oakland, CA | Fairfax town | 57.0% | 4.7% |
| San Francisco--Oakland, CA | Fairview CDP | 23.5% | 4.7% |
| San Francisco--Oakland, CA | Foster City city | 5.0% | 4.7% |
| San Francisco--Oakland, CA | Fremont city | 5.0% | 4.7% |
| San Francisco--Oakland, CA | Hayward city | 1.9% | 4.7% |
| San Francisco--Oakland, CA | Hercules city | 10.6% | 4.7% |
| San Francisco--Oakland, CA | Highlands-Baywood Park CDP | 22.8% | 4.7% |
| San Francisco--Oakland, CA | Hillsborough town | 44.9% | 4.7% |
| San Francisco--Oakland, CA | Kensington CDP | 31.1% | 4.7% |
| San Francisco--Oakland, CA | Kentfield CDP | 52.2% | 4.7% |
| San Francisco--Oakland, CA | Ladera CDP | 46.8% | 4.7% |
| San Francisco--Oakland, CA | Larkspur city | 32.2% | 4.7% |
| San Francisco--Oakland, CA | Lucas Valley-Marinwood CDP | 22.6% | 4.7% |
| San Francisco--Oakland, CA | Marin City CDP | 28.7% | 4.7% |
| San Francisco--Oakland, CA | Menlo Park city | 22.7% | 4.7% |
| San Francisco--Oakland, CA | Mill Valley city | 40.5% | 4.7% |
| San Francisco--Oakland, CA | Millbrae city | 16.0% | 4.7% |
| San Francisco--Oakland, CA | Milpitas city | 2.0% | 4.7% |
| San Francisco--Oakland, CA | Montalvin Manor CDP | 3.8% | 4.7% |
| San Francisco--Oakland, CA | Newark city | 2.4% | 4.7% |
| San Francisco--Oakland, CA | North Fair Oaks CDP | 5.6% | 4.7% |
| San Francisco--Oakland, CA | North Richmond CDP | 2.9% | 4.7% |
| San Francisco--Oakland, CA | Novato city | 21.8% | 4.7% |
| San Francisco--Oakland, CA | Oakland city | 3.8% | 4.7% |
| San Francisco--Oakland, CA | Pacifica city | 15.2% | 4.7% |
| San Francisco--Oakland, CA | Palo Alto city | 33.3% | 4.7% |
| San Francisco--Oakland, CA | Piedmont city | 21.1% | 4.7% |
| San Francisco--Oakland, CA | Pinole city | 17.3% | 4.7% |
| San Francisco--Oakland, CA | Port Costa CDP | 26.0% | 4.7% |
| San Francisco--Oakland, CA | Portola Valley town | 56.9% | 4.7% |
| San Francisco--Oakland, CA | Redwood City city | 8.6% | 4.7% |
| San Francisco--Oakland, CA | Richmond city | 2.4% | 4.7% |
| San Francisco--Oakland, CA | Rodeo CDP | 7.4% | 4.7% |
| San Francisco--Oakland, CA | Rollingwood CDP | 3.4% | 4.7% |
| San Francisco--Oakland, CA | Ross town | 66.3% | 4.7% |
| San Francisco--Oakland, CA | San Anselmo town | 44.7% | 4.7% |
| San Francisco--Oakland, CA | San Bruno city | 10.2% | 4.7% |
| San Francisco--Oakland, CA | San Carlos city | 15.8% | 4.7% |
| San Francisco--Oakland, CA | San Francisco city | 2.6% | 4.7% |
| San Francisco--Oakland, CA | San Leandro city | 2.4% | 4.7% |
| San Francisco--Oakland, CA | San Lorenzo CDP | 2.9% | 4.7% |
| San Francisco--Oakland, CA | San Mateo city | 7.5% | 4.7% |
| San Francisco--Oakland, CA | San Pablo city | 1.9% | 4.7% |
| San Francisco--Oakland, CA | San Rafael city | 23.2% | 4.7% |
| San Francisco--Oakland, CA | Santa Venetia CDP | 20.8% | 4.7% |
| San Francisco--Oakland, CA | Sausalito city | 37.8% | 4.7% |
| San Francisco--Oakland, CA | Sleepy Hollow CDP | 45.5% | 4.7% |
| San Francisco--Oakland, CA | South San Francisco city | 2.5% | 4.7% |
| San Francisco--Oakland, CA | Strawberry CDP | 32.4% | 4.7% |
| San Francisco--Oakland, CA | Tamalpais-Homestead Valley CDP | 49.4% | 4.7% |
| San Francisco--Oakland, CA | Tara Hills CDP | 7.3% | 4.7% |
| San Francisco--Oakland, CA | Tiburon town | 31.6% | 4.7% |
| San Francisco--Oakland, CA | Union City city | 2.4% | 4.7% |
| San Francisco--Oakland, CA | West Menlo Park CDP | 28.3% | 4.7% |
| San Francisco--Oakland, CA | Woodacre CDP | 65.5% | 4.7% |
| San Francisco--Oakland, CA | Woodside town | 54.8% | 4.7% |
| San Jose, CA | Alum Rock CDP | 2.9% | 4.9% |
| San Jose, CA | Burbank CDP | 3.1% | 4.9% |
| San Jose, CA | Cambrian Park CDP | 7.2% | 4.9% |
| San Jose, CA | Campbell city | 5.2% | 4.9% |
| San Jose, CA | Cupertino city | 6.5% | 4.9% |
| San Jose, CA | East Foothills CDP | 12.4% | 4.9% |
| San Jose, CA | Fruitdale CDP | 6.5% | 4.9% |
| San Jose, CA | Lexington Hills CDP | 83.8% | 4.9% |
| San Jose, CA | Los Altos Hills town | 41.5% | 4.9% |
| San Jose, CA | Los Altos city | 18.9% | 4.9% |
| San Jose, CA | Los Gatos town | 18.5% | 4.9% |
| San Jose, CA | Loyola CDP | 31.0% | 4.9% |
| San Jose, CA | Milpitas city | 2.6% | 4.9% |
| San Jose, CA | Monte Sereno city | 34.2% | 4.9% |
| San Jose, CA | Mountain View city | 9.4% | 4.9% |
| San Jose, CA | Palo Alto city | 14.9% | 4.9% |
| San Jose, CA | San Jose city | 3.9% | 4.9% |
| San Jose, CA | Santa Clara city | 5.8% | 4.9% |
| San Jose, CA | Saratoga city | 15.1% | 4.9% |
| San Jose, CA | Stanford CDP | 24.3% | 4.9% |
| San Jose, CA | Sunnyvale city | 5.0% | 4.9% |
| Sarasota--Bradenton, FL | Anna Maria city | 4.4% | 29.5% |
| Sarasota--Bradenton, FL | Bayshore Gardens CDP | 12.9% | 29.5% |
| Sarasota--Bradenton, FL | Bee Ridge CDP | 32.0% | 29.5% |
| Sarasota--Bradenton, FL | Bradenton Beach city | 1.9% | 29.5% |
| Sarasota--Bradenton, FL | Bradenton city | 31.2% | 29.5% |
| Sarasota--Bradenton, FL | Cortez CDP | 18.7% | 29.5% |
| Sarasota--Bradenton, FL | Desoto Lakes CDP | 35.4% | 29.5% |
| Sarasota--Bradenton, FL | Ellenton CDP | 39.6% | 29.5% |
| Sarasota--Bradenton, FL | Englewood CDP | 18.1% | 29.5% |
| Sarasota--Bradenton, FL | Fruitville CDP | 43.9% | 29.5% |
| Sarasota--Bradenton, FL | Grove City CDP | 9.8% | 29.5% |
| Sarasota--Bradenton, FL | Gulf Gate Estates CDP | 19.7% | 29.5% |
| Sarasota--Bradenton, FL | Holmes Beach city | 1.9% | 29.5% |
| Sarasota--Bradenton, FL | Kensington Park CDP | 20.3% | 29.5% |
| Sarasota--Bradenton, FL | Lake Sarasota CDP | 37.0% | 29.5% |
| Sarasota--Bradenton, FL | Laurel CDP | 19.4% | 29.5% |
| Sarasota--Bradenton, FL | Longboat Key town | 12.8% | 29.5% |
| Sarasota--Bradenton, FL | Manasota Key CDP | 12.7% | 29.5% |
| Sarasota--Bradenton, FL | Memphis CDP | 29.5% | 29.5% |
| Sarasota--Bradenton, FL | Nokomis CDP | 34.1% | 29.5% |
| Sarasota--Bradenton, FL | North Port city | 20.2% | 29.5% |
| Sarasota--Bradenton, FL | North Sarasota CDP | 31.2% | 29.5% |
| Sarasota--Bradenton, FL | Osprey CDP | 29.5% | 29.5% |
| Sarasota--Bradenton, FL | Palmetto city | 13.3% | 29.5% |
| Sarasota--Bradenton, FL | Plantation CDP | 24.2% | 29.5% |
| Sarasota--Bradenton, FL | Ridge Wood Heights CDP | 41.5% | 29.5% |
| Sarasota--Bradenton, FL | Rotonda CDP | 15.7% | 29.5% |
| Sarasota--Bradenton, FL | Samoset CDP | 34.6% | 29.5% |
| Sarasota--Bradenton, FL | Sarasota Springs CDP | 23.3% | 29.5% |
| Sarasota--Bradenton, FL | Sarasota city | 29.2% | 29.5% |
| Sarasota--Bradenton, FL | Siesta Key CDP | 21.9% | 29.5% |
| Sarasota--Bradenton, FL | South Bradenton CDP | 30.9% | 29.5% |
| Sarasota--Bradenton, FL | South Gate Ridge CDP | 35.7% | 29.5% |
| Sarasota--Bradenton, FL | South Sarasota CDP | 33.5% | 29.5% |
| Sarasota--Bradenton, FL | South Venice CDP | 37.1% | 29.5% |
| Sarasota--Bradenton, FL | Southgate CDP | 19.2% | 29.5% |
| Sarasota--Bradenton, FL | The Meadows CDP | 35.5% | 29.5% |
| Sarasota--Bradenton, FL | Vamo CDP | 36.9% | 29.5% |
| Sarasota--Bradenton, FL | Venice Gardens CDP | 23.7% | 29.5% |
| Sarasota--Bradenton, FL | Venice city | 25.3% | 29.5% |
| Sarasota--Bradenton, FL | West Bradenton CDP | 35.1% | 29.5% |
| Sarasota--Bradenton, FL | West Samoset CDP | 25.4% | 29.5% |
| Sarasota--Bradenton, FL | Whitfield CDP | 19.6% | 29.5% |
| Seattle, WA | Alderton CDP | 44.2% | 35.2% |
| Seattle, WA | Alderwood Manor CDP | 42.6% | 35.2% |
| Seattle, WA | Algona city | 23.3% | 35.2% |
| Seattle, WA | Ames Lake CDP | 72.7% | 35.2% |
| Seattle, WA | Artondale CDP | 74.8% | 35.2% |
| Seattle, WA | Auburn city | 23.3% | 35.2% |
| Seattle, WA | Beaux Arts Village town | 74.3% | 35.2% |
| Seattle, WA | Bellevue city | 40.4% | 35.2% |
| Seattle, WA | Black Diamond city | 45.0% | 35.2% |
| Seattle, WA | Bonney Lake city | 39.2% | 35.2% |
| Seattle, WA | Bothell East CDP | 48.8% | 35.2% |
| Seattle, WA | Bothell West CDP | 45.5% | 35.2% |
| Seattle, WA | Bothell city | 51.4% | 35.2% |
| Seattle, WA | Boulevard Park CDP | 47.5% | 35.2% |
| Seattle, WA | Brier city | 52.3% | 35.2% |
| Seattle, WA | Browns Point CDP | 30.9% | 35.2% |
| Seattle, WA | Bryn Mawr-Skyway CDP | 32.2% | 35.2% |
| Seattle, WA | Buckley city | 11.0% | 35.2% |
| Seattle, WA | Burien city | 38.3% | 35.2% |
| Seattle, WA | Burley CDP | 76.6% | 35.2% |
| Seattle, WA | Canterwood CDP | 71.9% | 35.2% |
| Seattle, WA | Carnation city | 17.5% | 35.2% |
| Seattle, WA | Cathcart CDP | 54.6% | 35.2% |
| Seattle, WA | Chain Lake CDP | 50.5% | 35.2% |
| Seattle, WA | Clearview CDP | 65.8% | 35.2% |
| Seattle, WA | Clover Creek CDP | 50.3% | 35.2% |
| Seattle, WA | Clyde Hill city | 44.1% | 35.2% |
| Seattle, WA | Cottage Lake CDP | 65.1% | 35.2% |
| Seattle, WA | Covington city | 22.7% | 35.2% |
| Seattle, WA | Dash Point CDP | 39.4% | 35.2% |
| Seattle, WA | Des Moines city | 35.7% | 35.2% |
| Seattle, WA | DuPont city | 29.7% | 35.2% |
| Seattle, WA | Duvall city | 30.7% | 35.2% |
| Seattle, WA | East Renton Highlands CDP | 52.1% | 35.2% |
| Seattle, WA | Eastmont CDP | 43.0% | 35.2% |
| Seattle, WA | Edgewood city | 50.0% | 35.2% |
| Seattle, WA | Edmonds city | 43.7% | 35.2% |
| Seattle, WA | Elk Plain CDP | 52.5% | 35.2% |
| Seattle, WA | Enumclaw city | 9.3% | 35.2% |
| Seattle, WA | Esperance CDP | 42.2% | 35.2% |
| Seattle, WA | Everett city | 24.1% | 35.2% |
| Seattle, WA | Fairwood CDP | 39.6% | 35.2% |
| Seattle, WA | Fall City CDP | 22.8% | 35.2% |
| Seattle, WA | Federal Way city | 43.2% | 35.2% |
| Seattle, WA | Fife Heights CDP | 57.8% | 35.2% |
| Seattle, WA | Fife city | 25.0% | 35.2% |
| Seattle, WA | Fircrest city | 28.5% | 35.2% |
| Seattle, WA | Fort Lewis CDP | 15.0% | 35.2% |
| Seattle, WA | Fox Island CDP | 64.5% | 35.2% |
| Seattle, WA | Frederickson CDP | 36.9% | 35.2% |
| Seattle, WA | Gig Harbor city | 46.3% | 35.2% |
| Seattle, WA | Graham CDP | 47.1% | 35.2% |
| Seattle, WA | High Bridge CDP | 78.6% | 35.2% |
| Seattle, WA | Hobart CDP | 64.1% | 35.2% |
| Seattle, WA | Hunts Point town | 64.3% | 35.2% |
| Seattle, WA | Issaquah city | 36.4% | 35.2% |
| Seattle, WA | Kenmore city | 50.8% | 35.2% |
| Seattle, WA | Kent city | 31.6% | 35.2% |
| Seattle, WA | Key Center CDP | 80.0% | 35.2% |
| Seattle, WA | Kirkland city | 42.6% | 35.2% |
| Seattle, WA | Lake Forest Park city | 64.0% | 35.2% |
| Seattle, WA | Lake Holm CDP | 60.5% | 35.2% |
| Seattle, WA | Lake Morton-Berrydale CDP | 54.1% | 35.2% |
| Seattle, WA | Lake Stickney CDP | 34.3% | 35.2% |
| Seattle, WA | Lake Tapps CDP | 51.1% | 35.2% |
| Seattle, WA | Lakeland North CDP | 41.9% | 35.2% |
| Seattle, WA | Lakeland South CDP | 50.1% | 35.2% |
| Seattle, WA | Lakewood city | 26.3% | 35.2% |
| Seattle, WA | Larch Way CDP | 38.5% | 35.2% |
| Seattle, WA | Lynnwood city | 37.5% | 35.2% |
| Seattle, WA | Maltby CDP | 64.6% | 35.2% |
| Seattle, WA | Maple Heights-Lake Desire CDP | 45.4% | 35.2% |
| Seattle, WA | Maple Valley city | 30.1% | 35.2% |
| Seattle, WA | Maplewood CDP | 72.3% | 35.2% |
| Seattle, WA | Martha Lake CDP | 44.7% | 35.2% |
| Seattle, WA | McChord AFB CDP | 13.7% | 35.2% |
| Seattle, WA | McMillin CDP | 37.5% | 35.2% |
| Seattle, WA | Meadowdale CDP | 54.6% | 35.2% |
| Seattle, WA | Medina city | 53.8% | 35.2% |
| Seattle, WA | Mercer Island city | 52.2% | 35.2% |
| Seattle, WA | Midland CDP | 33.6% | 35.2% |
| Seattle, WA | Mill Creek East CDP | 44.1% | 35.2% |
| Seattle, WA | Mill Creek city | 50.7% | 35.2% |
| Seattle, WA | Milton city | 36.9% | 35.2% |
| Seattle, WA | Mirrormont CDP | 85.2% | 35.2% |
| Seattle, WA | Monroe North CDP | 67.3% | 35.2% |
| Seattle, WA | Monroe city | 13.9% | 35.2% |
| Seattle, WA | Mountlake Terrace city | 39.8% | 35.2% |
| Seattle, WA | Mukilteo city | 50.6% | 35.2% |
| Seattle, WA | Newcastle city | 54.6% | 35.2% |
| Seattle, WA | Normandy Park city | 52.8% | 35.2% |
| Seattle, WA | North Fort Lewis CDP | 22.6% | 35.2% |
| Seattle, WA | North Lynnwood CDP | 43.5% | 35.2% |
| Seattle, WA | North Puyallup CDP | 27.7% | 35.2% |
| Seattle, WA | Orting city | 13.7% | 35.2% |
| Seattle, WA | Pacific city | 20.6% | 35.2% |
| Seattle, WA | Parkland CDP | 27.7% | 35.2% |
| Seattle, WA | Picnic Point CDP | 53.7% | 35.2% |
| Seattle, WA | Prairie Heights CDP | 36.2% | 35.2% |
| Seattle, WA | Prairie Ridge CDP | 34.6% | 35.2% |
| Seattle, WA | Purdy CDP | 80.4% | 35.2% |
| Seattle, WA | Puyallup city | 34.1% | 35.2% |
| Seattle, WA | Raft Island CDP | 62.3% | 35.2% |
| Seattle, WA | Ravensdale CDP | 25.0% | 35.2% |
| Seattle, WA | Redmond city | 41.0% | 35.2% |
| Seattle, WA | Renton city | 31.9% | 35.2% |
| Seattle, WA | Rosedale CDP | 75.5% | 35.2% |
| Seattle, WA | Ruston town | 8.0% | 35.2% |
| Seattle, WA | Sammamish city | 47.1% | 35.2% |
| Seattle, WA | SeaTac city | 29.0% | 35.2% |
| Seattle, WA | Seattle city | 28.4% | 35.2% |
| Seattle, WA | Shadow Lake CDP | 40.9% | 35.2% |
| Seattle, WA | Shoreline city | 44.3% | 35.2% |
| Seattle, WA | Silver Firs CDP | 40.0% | 35.2% |
| Seattle, WA | South Hill CDP | 45.5% | 35.2% |
| Seattle, WA | South Prairie town | 26.2% | 35.2% |
| Seattle, WA | Spanaway CDP | 31.5% | 35.2% |
| Seattle, WA | Stansberry Lake CDP | 75.1% | 35.2% |
| Seattle, WA | Steilacoom town | 29.3% | 35.2% |
| Seattle, WA | Summit CDP | 43.5% | 35.2% |
| Seattle, WA | Summit View CDP | 46.6% | 35.2% |
| Seattle, WA | Sumner city | 20.2% | 35.2% |
| Seattle, WA | Tacoma city | 18.5% | 35.2% |
| Seattle, WA | Tukwila city | 30.6% | 35.2% |
| Seattle, WA | Union Hill-Novelty Hill CDP | 64.5% | 35.2% |
| Seattle, WA | University Place city | 31.3% | 35.2% |
| Seattle, WA | Waller CDP | 47.7% | 35.2% |
| Seattle, WA | Wauna CDP | 73.2% | 35.2% |
| Seattle, WA | White Center CDP | 31.2% | 35.2% |
| Seattle, WA | Wollochet CDP | 53.3% | 35.2% |
| Seattle, WA | Woodinville city | 47.9% | 35.2% |
| Seattle, WA | Woods Creek CDP | 52.5% | 35.2% |
| Seattle, WA | Woodway city | 71.8% | 35.2% |
| Seattle, WA | Yarrow Point town | 51.2% | 35.2% |
| Springfield, MA--CT | Agawam Town city | 45.6% | 36.0% |
| Springfield, MA--CT | Amherst Center CDP | 18.9% | 36.0% |
| Springfield, MA--CT | Belchertown CDP | 69.1% | 36.0% |
| Springfield, MA--CT | Broad Brook CDP | 53.7% | 36.0% |
| Springfield, MA--CT | Chicopee city | 26.0% | 36.0% |
| Springfield, MA--CT | Easthampton Town city | 33.2% | 36.0% |
| Springfield, MA--CT | Granby CDP | 68.4% | 36.0% |
| Springfield, MA--CT | Hazardville CDP | 35.3% | 36.0% |
| Springfield, MA--CT | Holyoke city | 19.2% | 36.0% |
| Springfield, MA--CT | Longmeadow CDP | 50.4% | 36.0% |
| Springfield, MA--CT | Monson Center CDP | 47.4% | 36.0% |
| Springfield, MA--CT | North Amherst CDP | 42.0% | 36.0% |
| Springfield, MA--CT | Northampton city | 41.5% | 36.0% |
| Springfield, MA--CT | Palmer Town city | 59.0% | 36.0% |
| Springfield, MA--CT | Russell CDP | 80.4% | 36.0% |
| Springfield, MA--CT | Sherwood Manor CDP | 43.2% | 36.0% |
| Springfield, MA--CT | Somers CDP | 55.8% | 36.0% |
| Springfield, MA--CT | South Amherst CDP | 57.5% | 36.0% |
| Springfield, MA--CT | Southwood Acres CDP | 39.3% | 36.0% |
| Springfield, MA--CT | Springfield city | 23.8% | 36.0% |
| Springfield, MA--CT | Suffield Depot CDP | 62.5% | 36.0% |
| Springfield, MA--CT | Thompsonville CDP | 20.8% | 36.0% |
| Springfield, MA--CT | West Springfield Town city | 40.8% | 36.0% |
| Springfield, MA--CT | Westfield city | 43.2% | 36.0% |
| Springfield, MA--CT | Wilbraham CDP | 67.2% | 36.0% |
| Springfield, MA--CT | Windsor Locks CDP | 37.4% | 36.0% |
| St. Louis, MO--IL | Affton CDP | 46.2% | 37.0% |
| St. Louis, MO--IL | Alorton village | 9.5% | 37.0% |
| St. Louis, MO--IL | Arnold city | 56.2% | 37.0% |
| St. Louis, MO--IL | Ballwin city | 48.5% | 37.0% |
| St. Louis, MO--IL | Barnhart CDP | 85.3% | 37.0% |
| St. Louis, MO--IL | Bel-Nor village | 36.6% | 37.0% |
| St. Louis, MO--IL | Bel-Ridge village | 40.6% | 37.0% |
| St. Louis, MO--IL | Bella Villa city | 44.8% | 37.0% |
| St. Louis, MO--IL | Bellefontaine Neighbors city | 48.9% | 37.0% |
| St. Louis, MO--IL | Bellerive Acres city | 43.0% | 37.0% |
| St. Louis, MO--IL | Belleville city | 33.2% | 37.0% |
| St. Louis, MO--IL | Berkeley city | 5.1% | 37.0% |
| St. Louis, MO--IL | Beverly Hills city | 51.1% | 37.0% |
| St. Louis, MO--IL | Black Jack city | 37.9% | 37.0% |
| St. Louis, MO--IL | Breckenridge Hills city | 44.3% | 37.0% |
| St. Louis, MO--IL | Brentwood city | 74.8% | 37.0% |
| St. Louis, MO--IL | Bridgeton city | 14.8% | 37.0% |
| St. Louis, MO--IL | Brooklyn village | 0.3% | 37.0% |
| St. Louis, MO--IL | Byrnes Mill city | 46.6% | 37.0% |
| St. Louis, MO--IL | Cahokia village | 24.3% | 37.0% |
| St. Louis, MO--IL | Calverton Park city | 30.0% | 37.0% |
| St. Louis, MO--IL | Caseyville village | 22.6% | 37.0% |
| St. Louis, MO--IL | Castle Point CDP | 44.8% | 37.0% |
| St. Louis, MO--IL | Cedar Hill CDP | 66.1% | 37.0% |
| St. Louis, MO--IL | Centreville city | 10.1% | 37.0% |
| St. Louis, MO--IL | Champ village | 3.7% | 37.0% |
| St. Louis, MO--IL | Charlack city | 33.3% | 37.0% |
| St. Louis, MO--IL | Chesterfield city | 42.9% | 37.0% |
| St. Louis, MO--IL | Clarkson Valley city | 73.4% | 37.0% |
| St. Louis, MO--IL | Clayton city | 59.7% | 37.0% |
| St. Louis, MO--IL | Collinsville city | 14.4% | 37.0% |
| St. Louis, MO--IL | Columbia city | 31.0% | 37.0% |
| St. Louis, MO--IL | Concord CDP | 49.7% | 37.0% |
| St. Louis, MO--IL | Cool Valley city | 18.4% | 37.0% |
| St. Louis, MO--IL | Cottleville city | 23.3% | 37.0% |
| St. Louis, MO--IL | Country Club Hills city | 59.0% | 37.0% |
| St. Louis, MO--IL | Country Life Acres village | 48.3% | 37.0% |
| St. Louis, MO--IL | Crestwood city | 39.8% | 37.0% |
| St. Louis, MO--IL | Creve Coeur city | 37.2% | 37.0% |
| St. Louis, MO--IL | Crystal City city | 50.7% | 37.0% |
| St. Louis, MO--IL | Crystal Lake Park city | 41.2% | 37.0% |
| St. Louis, MO--IL | Dardenne Prairie city | 20.2% | 37.0% |
| St. Louis, MO--IL | Dellwood city | 65.8% | 37.0% |
| St. Louis, MO--IL | Des Peres city | 40.1% | 37.0% |
| St. Louis, MO--IL | Dupo village | 0.7% | 37.0% |
| St. Louis, MO--IL | East St. Louis city | 18.6% | 37.0% |
| St. Louis, MO--IL | Edmundson city | 21.9% | 37.0% |
| St. Louis, MO--IL | Edwardsville city | 42.5% | 37.0% |
| St. Louis, MO--IL | Ellisville city | 49.2% | 37.0% |
| St. Louis, MO--IL | Fairmont City village | 5.5% | 37.0% |
| St. Louis, MO--IL | Fairview Heights city | 52.9% | 37.0% |
| St. Louis, MO--IL | Fenton city | 33.0% | 37.0% |
| St. Louis, MO--IL | Ferguson city | 46.0% | 37.0% |
| St. Louis, MO--IL | Festus city | 66.9% | 37.0% |
| St. Louis, MO--IL | Flint Hill city | 26.0% | 37.0% |
| St. Louis, MO--IL | Flordell Hills city | 57.2% | 37.0% |
| St. Louis, MO--IL | Florissant city | 36.8% | 37.0% |
| St. Louis, MO--IL | Freeburg village | 41.2% | 37.0% |
| St. Louis, MO--IL | Frontenac city | 48.2% | 37.0% |
| St. Louis, MO--IL | Glasgow Village CDP | 54.4% | 37.0% |
| St. Louis, MO--IL | Glen Carbon village | 37.0% | 37.0% |
| St. Louis, MO--IL | Glen Echo Park village | 45.9% | 37.0% |
| St. Louis, MO--IL | Glendale city | 52.5% | 37.0% |
| St. Louis, MO--IL | Granite City city | 10.6% | 37.0% |
| St. Louis, MO--IL | Grantwood Village town | 53.1% | 37.0% |
| St. Louis, MO--IL | Green Park city | 21.8% | 37.0% |
| St. Louis, MO--IL | Greendale city | 66.8% | 37.0% |
| St. Louis, MO--IL | Hanley Hills village | 17.6% | 37.0% |
| St. Louis, MO--IL | Hazelwood city | 20.6% | 37.0% |
| St. Louis, MO--IL | Herculaneum city | 60.5% | 37.0% |
| St. Louis, MO--IL | High Ridge CDP | 82.0% | 37.0% |
| St. Louis, MO--IL | Hillsdale village | 56.7% | 37.0% |
| St. Louis, MO--IL | Horine CDP | 81.2% | 37.0% |
| St. Louis, MO--IL | Huntleigh city | 53.1% | 37.0% |
| St. Louis, MO--IL | Imperial CDP | 70.0% | 37.0% |
| St. Louis, MO--IL | Jennings city | 49.6% | 37.0% |
| St. Louis, MO--IL | Kimmswick city | 38.6% | 37.0% |
| St. Louis, MO--IL | Kinloch city | 1.1% | 37.0% |
| St. Louis, MO--IL | Kirkwood city | 48.9% | 37.0% |
| St. Louis, MO--IL | Ladue city | 61.9% | 37.0% |
| St. Louis, MO--IL | Lake St. Louis city | 27.1% | 37.0% |
| St. Louis, MO--IL | Lakeshire city | 46.4% | 37.0% |
| St. Louis, MO--IL | Lebanon city | 56.8% | 37.0% |
| St. Louis, MO--IL | Lemay CDP | 39.9% | 37.0% |
| St. Louis, MO--IL | Mackenzie village | 62.2% | 37.0% |
| St. Louis, MO--IL | Madison city | 0.0% | 37.0% |
| St. Louis, MO--IL | Manchester city | 40.1% | 37.0% |
| St. Louis, MO--IL | Maplewood city | 35.5% | 37.0% |
| St. Louis, MO--IL | Marlborough village | 53.4% | 37.0% |
| St. Louis, MO--IL | Maryland Heights city | 31.6% | 37.0% |
| St. Louis, MO--IL | Maryville village | 51.2% | 37.0% |
| St. Louis, MO--IL | Mehlville CDP | 46.6% | 37.0% |
| St. Louis, MO--IL | Mitchell CDP | 20.1% | 37.0% |
| St. Louis, MO--IL | Moline Acres city | 54.1% | 37.0% |
| St. Louis, MO--IL | Murphy CDP | 62.0% | 37.0% |
| St. Louis, MO--IL | Normandy city | 36.4% | 37.0% |
| St. Louis, MO--IL | Northwoods city | 34.6% | 37.0% |
| St. Louis, MO--IL | Norwood Court town | 84.4% | 37.0% |
| St. Louis, MO--IL | O'Fallon city | 23.1% | 37.0% |
| St. Louis, MO--IL | Oakland city | 35.8% | 37.0% |
| St. Louis, MO--IL | Oakville CDP | 57.2% | 37.0% |
| St. Louis, MO--IL | Old Jamestown CDP | 38.7% | 37.0% |
| St. Louis, MO--IL | Olivette city | 36.7% | 37.0% |
| St. Louis, MO--IL | Olympian Village city | 59.7% | 37.0% |
| St. Louis, MO--IL | Overland city | 42.2% | 37.0% |
| St. Louis, MO--IL | Pagedale city | 51.1% | 37.0% |
| St. Louis, MO--IL | Parkdale village | 94.4% | 37.0% |
| St. Louis, MO--IL | Pasadena Hills city | 54.1% | 37.0% |
| St. Louis, MO--IL | Pasadena Park village | 59.4% | 37.0% |
| St. Louis, MO--IL | Peaceful Village village | 57.9% | 37.0% |
| St. Louis, MO--IL | Pevely city | 69.3% | 37.0% |
| St. Louis, MO--IL | Pine Lawn city | 53.9% | 37.0% |
| St. Louis, MO--IL | Pontoon Beach village | 20.6% | 37.0% |
| St. Louis, MO--IL | Richmond Heights city | 38.8% | 37.0% |
| St. Louis, MO--IL | Riverview village | 41.8% | 37.0% |
| St. Louis, MO--IL | Rock Hill city | 63.1% | 37.0% |
| St. Louis, MO--IL | Sappington CDP | 54.2% | 37.0% |
| St. Louis, MO--IL | Sauget village | 24.5% | 37.0% |
| St. Louis, MO--IL | Scotsdale town | 97.6% | 37.0% |
| St. Louis, MO--IL | Scott AFB CDP | 46.0% | 37.0% |
| St. Louis, MO--IL | Shiloh village | 34.1% | 37.0% |
| St. Louis, MO--IL | Shrewsbury city | 53.2% | 37.0% |
| St. Louis, MO--IL | South Roxana village | 10.2% | 37.0% |
| St. Louis, MO--IL | Spanish Lake CDP | 51.1% | 37.0% |
| St. Louis, MO--IL | St. Ann city | 37.3% | 37.0% |
| St. Louis, MO--IL | St. Charles city | 18.3% | 37.0% |
| St. Louis, MO--IL | St. George CDP | 50.1% | 37.0% |
| St. Louis, MO--IL | St. John city | 39.6% | 37.0% |
| St. Louis, MO--IL | St. Louis city | 22.3% | 37.0% |
| St. Louis, MO--IL | St. Paul city | 54.5% | 37.0% |
| St. Louis, MO--IL | St. Peters city | 30.7% | 37.0% |
| St. Louis, MO--IL | Sunset Hills city | 53.8% | 37.0% |
| St. Louis, MO--IL | Swansea village | 33.5% | 37.0% |
| St. Louis, MO--IL | Sycamore Hills village | 47.6% | 37.0% |
| St. Louis, MO--IL | Town and Country city | 47.4% | 37.0% |
| St. Louis, MO--IL | Troy city | 34.7% | 37.0% |
| St. Louis, MO--IL | Twin Oaks village | 31.6% | 37.0% |
| St. Louis, MO--IL | University City city | 42.3% | 37.0% |
| St. Louis, MO--IL | Uplands Park village | 45.3% | 37.0% |
| St. Louis, MO--IL | Valley Park city | 46.8% | 37.0% |
| St. Louis, MO--IL | Velda City city | 49.1% | 37.0% |
| St. Louis, MO--IL | Velda Village Hills city | 51.1% | 37.0% |
| St. Louis, MO--IL | Venice city | 0.7% | 37.0% |
| St. Louis, MO--IL | Vinita Park city | 25.9% | 37.0% |
| St. Louis, MO--IL | Warson Woods city | 64.2% | 37.0% |
| St. Louis, MO--IL | Washington Park village | 31.9% | 37.0% |
| St. Louis, MO--IL | Webster Groves city | 53.9% | 37.0% |
| St. Louis, MO--IL | Weldon Spring Heights town | 29.5% | 37.0% |
| St. Louis, MO--IL | Weldon Spring city | 25.6% | 37.0% |
| St. Louis, MO--IL | Wellston city | 33.1% | 37.0% |
| St. Louis, MO--IL | Wentzville city | 25.2% | 37.0% |
| St. Louis, MO--IL | Westwood village | 35.4% | 37.0% |
| St. Louis, MO--IL | Wilbur Park village | 46.8% | 37.0% |
| St. Louis, MO--IL | Wildwood city | 68.1% | 37.0% |
| St. Louis, MO--IL | Winchester city | 12.1% | 37.0% |
| St. Louis, MO--IL | Woodson Terrace city | 31.7% | 37.0% |
| Syracuse, NY | Baldwinsville village | 37.5% | 35.7% |
| Syracuse, NY | Brewerton CDP | 39.2% | 35.7% |
| Syracuse, NY | Bridgeport CDP | 20.3% | 35.7% |
| Syracuse, NY | Camillus village | 41.6% | 35.7% |
| Syracuse, NY | Central Square village | 40.9% | 35.7% |
| Syracuse, NY | East Syracuse village | 24.6% | 35.7% |
| Syracuse, NY | Fairmount CDP | 43.5% | 35.7% |
| Syracuse, NY | Fayetteville village | 31.4% | 35.7% |
| Syracuse, NY | Galeville CDP | 35.0% | 35.7% |
| Syracuse, NY | Lakeland CDP | 39.5% | 35.7% |
| Syracuse, NY | Liverpool village | 40.0% | 35.7% |
| Syracuse, NY | Lyncourt CDP | 22.5% | 35.7% |
| Syracuse, NY | Manlius village | 34.5% | 35.7% |
| Syracuse, NY | Marcellus village | 46.1% | 35.7% |
| Syracuse, NY | Mattydale CDP | 39.0% | 35.7% |
| Syracuse, NY | Minoa village | 24.0% | 35.7% |
| Syracuse, NY | Nedrow CDP | 39.1% | 35.7% |
| Syracuse, NY | North Syracuse village | 40.8% | 35.7% |
| Syracuse, NY | Phoenix village | 39.3% | 35.7% |
| Syracuse, NY | Seneca Knolls CDP | 43.4% | 35.7% |
| Syracuse, NY | Solvay village | 24.3% | 35.7% |
| Syracuse, NY | Syracuse city | 24.4% | 35.7% |
| Syracuse, NY | Village Green CDP | 39.0% | 35.7% |
| Syracuse, NY | Westvale CDP | 44.8% | 35.7% |
| Tampa--St. Petersburg, FL | Apollo Beach CDP | 24.9% | 33.5% |
| Tampa--St. Petersburg, FL | Balm CDP | 18.4% | 33.5% |
| Tampa--St. Petersburg, FL | Bardmoor CDP | 20.0% | 33.5% |
| Tampa--St. Petersburg, FL | Bay Pines CDP | 16.6% | 33.5% |
| Tampa--St. Petersburg, FL | Bayonet Point CDP | 18.4% | 33.5% |
| Tampa--St. Petersburg, FL | Beacon Square CDP | 20.2% | 33.5% |
| Tampa--St. Petersburg, FL | Bear Creek CDP | 18.6% | 33.5% |
| Tampa--St. Petersburg, FL | Belleair Beach city | 18.7% | 33.5% |
| Tampa--St. Petersburg, FL | Belleair Bluffs city | 24.6% | 33.5% |
| Tampa--St. Petersburg, FL | Belleair Shore town | 26.2% | 33.5% |
| Tampa--St. Petersburg, FL | Belleair town | 40.0% | 33.5% |
| Tampa--St. Petersburg, FL | Bloomingdale CDP | 42.4% | 33.5% |
| Tampa--St. Petersburg, FL | Brandon CDP | 35.4% | 33.5% |
| Tampa--St. Petersburg, FL | Carrollwood CDP | 41.6% | 33.5% |
| Tampa--St. Petersburg, FL | Cheval CDP | 48.4% | 33.5% |
| Tampa--St. Petersburg, FL | Citrus Park CDP | 44.0% | 33.5% |
| Tampa--St. Petersburg, FL | Clearwater city | 25.5% | 33.5% |
| Tampa--St. Petersburg, FL | Connerton CDP | 64.9% | 33.5% |
| Tampa--St. Petersburg, FL | Dover CDP | 44.1% | 33.5% |
| Tampa--St. Petersburg, FL | Dunedin city | 30.8% | 33.5% |
| Tampa--St. Petersburg, FL | East Lake CDP | 45.6% | 33.5% |
| Tampa--St. Petersburg, FL | East Lake-Orient Park CDP | 51.7% | 33.5% |
| Tampa--St. Petersburg, FL | Egypt Lake-Leto CDP | 27.6% | 33.5% |
| Tampa--St. Petersburg, FL | Elfers CDP | 13.1% | 33.5% |
| Tampa--St. Petersburg, FL | Feather Sound CDP | 34.8% | 33.5% |
| Tampa--St. Petersburg, FL | Fish Hawk CDP | 53.5% | 33.5% |
| Tampa--St. Petersburg, FL | Gibsonton CDP | 47.9% | 33.5% |
| Tampa--St. Petersburg, FL | Greenbriar CDP | 27.6% | 33.5% |
| Tampa--St. Petersburg, FL | Gulfport city | 19.4% | 33.5% |
| Tampa--St. Petersburg, FL | Harbor Bluffs CDP | 20.7% | 33.5% |
| Tampa--St. Petersburg, FL | Holiday CDP | 5.9% | 33.5% |
| Tampa--St. Petersburg, FL | Hudson CDP | 34.3% | 33.5% |
| Tampa--St. Petersburg, FL | Indian Rocks Beach city | 24.0% | 33.5% |
| Tampa--St. Petersburg, FL | Indian Shores town | 13.9% | 33.5% |
| Tampa--St. Petersburg, FL | Jasmine Estates CDP | 8.2% | 33.5% |
| Tampa--St. Petersburg, FL | Kenneth City town | 15.7% | 33.5% |
| Tampa--St. Petersburg, FL | Key Vista CDP | 41.7% | 33.5% |
| Tampa--St. Petersburg, FL | Keystone CDP | 46.3% | 33.5% |
| Tampa--St. Petersburg, FL | Lake Magdalene CDP | 52.8% | 33.5% |
| Tampa--St. Petersburg, FL | Land O' Lakes CDP | 45.9% | 33.5% |
| Tampa--St. Petersburg, FL | Largo city | 23.3% | 33.5% |
| Tampa--St. Petersburg, FL | Lealman CDP | 24.2% | 33.5% |
| Tampa--St. Petersburg, FL | Lutz CDP | 67.4% | 33.5% |
| Tampa--St. Petersburg, FL | Madeira Beach city | 7.9% | 33.5% |
| Tampa--St. Petersburg, FL | Mango CDP | 56.0% | 33.5% |
| Tampa--St. Petersburg, FL | Meadow Oaks CDP | 33.8% | 33.5% |
| Tampa--St. Petersburg, FL | Moon Lake CDP | 53.6% | 33.5% |
| Tampa--St. Petersburg, FL | New Port Richey East CDP | 34.0% | 33.5% |
| Tampa--St. Petersburg, FL | New Port Richey city | 22.2% | 33.5% |
| Tampa--St. Petersburg, FL | North Redington Beach town | 2.5% | 33.5% |
| Tampa--St. Petersburg, FL | Northdale CDP | 45.2% | 33.5% |
| Tampa--St. Petersburg, FL | Odessa CDP | 46.5% | 33.5% |
| Tampa--St. Petersburg, FL | Oldsmar city | 39.4% | 33.5% |
| Tampa--St. Petersburg, FL | Palm Harbor CDP | 37.8% | 33.5% |
| Tampa--St. Petersburg, FL | Palm River-Clair Mel CDP | 38.1% | 33.5% |
| Tampa--St. Petersburg, FL | Pebble Creek CDP | 52.5% | 33.5% |
| Tampa--St. Petersburg, FL | Pinellas Park city | 25.8% | 33.5% |
| Tampa--St. Petersburg, FL | Plant City city | 48.0% | 33.5% |
| Tampa--St. Petersburg, FL | Port Richey city | 33.4% | 33.5% |
| Tampa--St. Petersburg, FL | Progress Village CDP | 36.3% | 33.5% |
| Tampa--St. Petersburg, FL | Redington Beach town | 8.1% | 33.5% |
| Tampa--St. Petersburg, FL | Redington Shores town | 1.2% | 33.5% |
| Tampa--St. Petersburg, FL | Ridgecrest CDP | 31.5% | 33.5% |
| Tampa--St. Petersburg, FL | River Ridge CDP | 40.3% | 33.5% |
| Tampa--St. Petersburg, FL | Riverview CDP | 37.1% | 33.5% |
| Tampa--St. Petersburg, FL | Ruskin CDP | 44.0% | 33.5% |
| Tampa--St. Petersburg, FL | Safety Harbor city | 51.5% | 33.5% |
| Tampa--St. Petersburg, FL | Seffner CDP | 47.8% | 33.5% |
| Tampa--St. Petersburg, FL | Seminole city | 15.0% | 33.5% |
| Tampa--St. Petersburg, FL | South Highpoint CDP | 42.5% | 33.5% |
| Tampa--St. Petersburg, FL | South Pasadena city | 15.8% | 33.5% |
| Tampa--St. Petersburg, FL | St. Pete Beach city | 10.8% | 33.5% |
| Tampa--St. Petersburg, FL | St. Petersburg city | 26.5% | 33.5% |
| Tampa--St. Petersburg, FL | Sun City Center CDP | 21.3% | 33.5% |
| Tampa--St. Petersburg, FL | Tampa city | 38.2% | 33.5% |
| Tampa--St. Petersburg, FL | Tarpon Springs city | 22.5% | 33.5% |
| Tampa--St. Petersburg, FL | Temple Terrace city | 54.5% | 33.5% |
| Tampa--St. Petersburg, FL | Thonotosassa CDP | 65.4% | 33.5% |
| Tampa--St. Petersburg, FL | Tierra Verde CDP | 22.7% | 33.5% |
| Tampa--St. Petersburg, FL | Town 'n' Country CDP | 20.2% | 33.5% |
| Tampa--St. Petersburg, FL | Treasure Island city | 2.6% | 33.5% |
| Tampa--St. Petersburg, FL | Trinity CDP | 25.8% | 33.5% |
| Tampa--St. Petersburg, FL | University CDP | 41.9% | 33.5% |
| Tampa--St. Petersburg, FL | Valrico CDP | 40.8% | 33.5% |
| Tampa--St. Petersburg, FL | Wesley Chapel CDP | 53.9% | 33.5% |
| Tampa--St. Petersburg, FL | West Lealman CDP | 27.2% | 33.5% |
| Tampa--St. Petersburg, FL | Westchase CDP | 38.4% | 33.5% |
| Tampa--St. Petersburg, FL | Wimauma CDP | 40.3% | 33.5% |
| Toledo, OH--MI | Curtice CDP | 26.6% | 21.3% |
| Toledo, OH--MI | Harbor View village | 24.7% | 21.3% |
| Toledo, OH--MI | Holland village | 14.9% | 21.3% |
| Toledo, OH--MI | Lambertville CDP | 63.2% | 21.3% |
| Toledo, OH--MI | Maumee city | 13.9% | 21.3% |
| Toledo, OH--MI | Millbury village | 13.0% | 21.3% |
| Toledo, OH--MI | Northwood city | 13.5% | 21.3% |
| Toledo, OH--MI | Oregon city | 16.3% | 21.3% |
| Toledo, OH--MI | Ottawa Hills village | 34.1% | 21.3% |
| Toledo, OH--MI | Perrysburg city | 17.9% | 21.3% |
| Toledo, OH--MI | Rossford city | 17.9% | 21.3% |
| Toledo, OH--MI | Stony Ridge CDP | 29.6% | 21.3% |
| Toledo, OH--MI | Swanton village | 25.8% | 21.3% |
| Toledo, OH--MI | Sylvania city | 52.7% | 21.3% |
| Toledo, OH--MI | Temperance CDP | 52.8% | 21.3% |
| Toledo, OH--MI | Toledo city | 18.5% | 21.3% |
| Toledo, OH--MI | Walbridge village | 10.8% | 21.3% |
| Toledo, OH--MI | Waterville city | 29.4% | 21.3% |
| Toledo, OH--MI | Whitehouse village | 27.5% | 21.3% |
| Tucson, AZ | Casas Adobes CDP | 4.6% | 4.7% |
| Tucson, AZ | Catalina CDP | 8.5% | 4.7% |
| Tucson, AZ | Catalina Foothills CDP | 7.4% | 4.7% |
| Tucson, AZ | Drexel Heights CDP | 3.2% | 4.7% |
| Tucson, AZ | Flowing Wells CDP | 4.0% | 4.7% |
| Tucson, AZ | Marana town | 3.7% | 4.7% |
| Tucson, AZ | Oro Valley town | 6.1% | 4.7% |
| Tucson, AZ | Saddlebrooke CDP | 3.6% | 4.7% |
| Tucson, AZ | South Tucson city | 1.9% | 4.7% |
| Tucson, AZ | Summit CDP | 2.0% | 4.7% |
| Tucson, AZ | Tanque Verde CDP | 11.1% | 4.7% |
| Tucson, AZ | Tucson Estates CDP | 1.9% | 4.7% |
| Tucson, AZ | Tucson city | 5.0% | 4.7% |
| Tucson, AZ | Valencia West CDP | 1.3% | 4.7% |
| Tulsa, OK | Bixby city | 5.1% | 9.8% |
| Tulsa, OK | Broken Arrow city | 9.7% | 9.8% |
| Tulsa, OK | Catoosa city | 4.0% | 9.8% |
| Tulsa, OK | Collinsville city | 2.7% | 9.8% |
| Tulsa, OK | Coweta city | 2.6% | 9.8% |
| Tulsa, OK | Glenpool city | 6.3% | 9.8% |
| Tulsa, OK | Jenks city | 4.1% | 9.8% |
| Tulsa, OK | Kiefer town | 9.9% | 9.8% |
| Tulsa, OK | Oakhurst CDP | 6.5% | 9.8% |
| Tulsa, OK | Owasso city | 7.3% | 9.8% |
| Tulsa, OK | Sand Springs city | 3.2% | 9.8% |
| Tulsa, OK | Sapulpa city | 7.6% | 9.8% |
| Tulsa, OK | Sperry town | 5.1% | 9.8% |
| Tulsa, OK | Tulsa city | 12.6% | 9.8% |
| Tulsa, OK | Turley CDP | 17.0% | 9.8% |
| Virginia Beach, VA | Bethel Manor CDP | 1.9% | 32.4% |
| Virginia Beach, VA | Carrollton CDP | 51.1% | 32.4% |
| Virginia Beach, VA | Chesapeake city | 35.1% | 32.4% |
| Virginia Beach, VA | Gloucester Point CDP | 52.1% | 32.4% |
| Virginia Beach, VA | Hampton city | 37.2% | 32.4% |
| Virginia Beach, VA | Newport News city | 41.4% | 32.4% |
| Virginia Beach, VA | Norfolk city | 23.7% | 32.4% |
| Virginia Beach, VA | Poquoson city | 59.4% | 32.4% |
| Virginia Beach, VA | Portsmouth city | 31.7% | 32.4% |
| Virginia Beach, VA | Suffolk city | 40.5% | 32.4% |
| Virginia Beach, VA | Virginia Beach city | 25.1% | 32.4% |
| Virginia Beach, VA | Yorktown CDP | 60.8% | 32.4% |
| Washington, DC--VA--MD | Accokeek CDP | 15.9% | 41.8% |
| Washington, DC--VA--MD | Adelphi CDP | 38.8% | 41.8% |
| Washington, DC--VA--MD | Alexandria city | 27.7% | 41.8% |
| Washington, DC--VA--MD | Andrews AFB CDP | 40.6% | 41.8% |
| Washington, DC--VA--MD | Annandale CDP | 49.1% | 41.8% |
| Washington, DC--VA--MD | Aquia Harbour CDP | 63.4% | 41.8% |
| Washington, DC--VA--MD | Arlington CDP | 32.3% | 41.8% |
| Washington, DC--VA--MD | Ashburn CDP | 34.9% | 41.8% |
| Washington, DC--VA--MD | Ashton-Sandy Spring CDP | 72.7% | 41.8% |
| Washington, DC--VA--MD | Aspen Hill CDP | 45.2% | 41.8% |
| Washington, DC--VA--MD | Bailey's Crossroads CDP | 29.3% | 41.8% |
| Washington, DC--VA--MD | Belle Haven CDP | 46.8% | 41.8% |
| Washington, DC--VA--MD | Belmont CDP | 35.8% | 41.8% |
| Washington, DC--VA--MD | Beltsville CDP | 45.3% | 41.8% |
| Washington, DC--VA--MD | Berwyn Heights town | 59.3% | 41.8% |
| Washington, DC--VA--MD | Bethesda CDP | 51.8% | 41.8% |
| Washington, DC--VA--MD | Bladensburg town | 39.6% | 41.8% |
| Washington, DC--VA--MD | Boswell's Corner CDP | 56.6% | 41.8% |
| Washington, DC--VA--MD | Bowie city | 45.6% | 41.8% |
| Washington, DC--VA--MD | Brambleton CDP | 15.9% | 41.8% |
| Washington, DC--VA--MD | Brandywine CDP | 48.2% | 41.8% |
| Washington, DC--VA--MD | Brentwood town | 38.0% | 41.8% |
| Washington, DC--VA--MD | Broadlands CDP | 42.1% | 41.8% |
| Washington, DC--VA--MD | Brock Hall CDP | 62.0% | 41.8% |
| Washington, DC--VA--MD | Brookeville town | 70.5% | 41.8% |
| Washington, DC--VA--MD | Brookmont CDP | 66.9% | 41.8% |
| Washington, DC--VA--MD | Buckhall CDP | 63.4% | 41.8% |
| Washington, DC--VA--MD | Bull Run CDP | 35.6% | 41.8% |
| Washington, DC--VA--MD | Burke CDP | 51.9% | 41.8% |
| Washington, DC--VA--MD | Burke Centre CDP | 55.3% | 41.8% |
| Washington, DC--VA--MD | Burtonsville CDP | 57.9% | 41.8% |
| Washington, DC--VA--MD | Cabin John CDP | 69.0% | 41.8% |
| Washington, DC--VA--MD | Calverton CDP | 46.7% | 41.8% |
| Washington, DC--VA--MD | Camp Springs CDP | 53.3% | 41.8% |
| Washington, DC--VA--MD | Capitol Heights town | 49.7% | 41.8% |
| Washington, DC--VA--MD | Cascades CDP | 33.1% | 41.8% |
| Washington, DC--VA--MD | Centreville CDP | 41.6% | 41.8% |
| Washington, DC--VA--MD | Chantilly CDP | 33.7% | 41.8% |
| Washington, DC--VA--MD | Cherry Hill CDP | 33.1% | 41.8% |
| Washington, DC--VA--MD | Cheverly town | 57.5% | 41.8% |
| Washington, DC--VA--MD | Chevy Chase CDP | 57.4% | 41.8% |
| Washington, DC--VA--MD | Chevy Chase Section Five village | 60.5% | 41.8% |
| Washington, DC--VA--MD | Chevy Chase Section Three village | 49.9% | 41.8% |
| Washington, DC--VA--MD | Chevy Chase View town | 59.3% | 41.8% |
| Washington, DC--VA--MD | Chevy Chase Village town | 59.3% | 41.8% |
| Washington, DC--VA--MD | Chevy Chase town | 55.6% | 41.8% |
| Washington, DC--VA--MD | Chillum CDP | 34.1% | 41.8% |
| Washington, DC--VA--MD | Clarksburg CDP | 19.0% | 41.8% |
| Washington, DC--VA--MD | Clifton town | 65.9% | 41.8% |
| Washington, DC--VA--MD | Clinton CDP | 41.5% | 41.8% |
| Washington, DC--VA--MD | Cloverly CDP | 69.2% | 41.8% |
| Washington, DC--VA--MD | Colesville CDP | 60.7% | 41.8% |
| Washington, DC--VA--MD | College Park city | 27.6% | 41.8% |
| Washington, DC--VA--MD | Colmar Manor town | 26.8% | 41.8% |
| Washington, DC--VA--MD | Coral Hills CDP | 43.9% | 41.8% |
| Washington, DC--VA--MD | Cottage City town | 46.4% | 41.8% |
| Washington, DC--VA--MD | Countryside CDP | 52.4% | 41.8% |
| Washington, DC--VA--MD | County Center CDP | 32.3% | 41.8% |
| Washington, DC--VA--MD | Croom CDP | 42.7% | 41.8% |
| Washington, DC--VA--MD | Crosspointe CDP | 53.9% | 41.8% |
| Washington, DC--VA--MD | Dale City CDP | 44.8% | 41.8% |
| Washington, DC--VA--MD | Damascus CDP | 73.6% | 41.8% |
| Washington, DC--VA--MD | Darnestown CDP | 74.0% | 41.8% |
| Washington, DC--VA--MD | Derwood CDP | 54.6% | 41.8% |
| Washington, DC--VA--MD | District Heights city | 46.7% | 41.8% |
| Washington, DC--VA--MD | Dranesville CDP | 48.0% | 41.8% |
| Washington, DC--VA--MD | Dulles Town Center CDP | 15.4% | 41.8% |
| Washington, DC--VA--MD | Dumfries town | 27.8% | 41.8% |
| Washington, DC--VA--MD | Dunn Loring CDP | 51.9% | 41.8% |
| Washington, DC--VA--MD | East Riverdale CDP | 48.2% | 41.8% |
| Washington, DC--VA--MD | Edmonston town | 43.6% | 41.8% |
| Washington, DC--VA--MD | Fair Lakes CDP | 36.7% | 41.8% |
| Washington, DC--VA--MD | Fair Oaks CDP | 29.4% | 41.8% |
| Washington, DC--VA--MD | Fairfax Station CDP | 61.0% | 41.8% |
| Washington, DC--VA--MD | Fairfax city | 39.9% | 41.8% |
| Washington, DC--VA--MD | Fairland CDP | 49.4% | 41.8% |
| Washington, DC--VA--MD | Fairmount Heights town | 41.5% | 41.8% |
| Washington, DC--VA--MD | Fairwood CDP | 53.9% | 41.8% |
| Washington, DC--VA--MD | Falls Church city | 49.0% | 41.8% |
| Washington, DC--VA--MD | Floris CDP | 43.8% | 41.8% |
| Washington, DC--VA--MD | Forest Glen CDP | 39.7% | 41.8% |
| Washington, DC--VA--MD | Forest Heights town | 54.6% | 41.8% |
| Washington, DC--VA--MD | Forestville CDP | 41.9% | 41.8% |
| Washington, DC--VA--MD | Fort Belvoir CDP | 28.6% | 41.8% |
| Washington, DC--VA--MD | Fort Hunt CDP | 59.7% | 41.8% |
| Washington, DC--VA--MD | Fort Washington CDP | 65.5% | 41.8% |
| Washington, DC--VA--MD | Four Corners CDP | 55.5% | 41.8% |
| Washington, DC--VA--MD | Franconia CDP | 44.6% | 41.8% |
| Washington, DC--VA--MD | Franklin Farm CDP | 48.0% | 41.8% |
| Washington, DC--VA--MD | Friendly CDP | 54.4% | 41.8% |
| Washington, DC--VA--MD | Friendship Heights Village CDP | 15.5% | 41.8% |
| Washington, DC--VA--MD | Gainesville CDP | 39.7% | 41.8% |
| Washington, DC--VA--MD | Gaithersburg city | 36.5% | 41.8% |
| Washington, DC--VA--MD | Garrett Park town | 76.7% | 41.8% |
| Washington, DC--VA--MD | George Mason CDP | 34.0% | 41.8% |
| Washington, DC--VA--MD | Germantown CDP | 45.5% | 41.8% |
| Washington, DC--VA--MD | Glassmanor CDP | 48.1% | 41.8% |
| Washington, DC--VA--MD | Glen Echo town | 59.5% | 41.8% |
| Washington, DC--VA--MD | Glenarden city | 33.1% | 41.8% |
| Washington, DC--VA--MD | Glenmont CDP | 42.8% | 41.8% |
| Washington, DC--VA--MD | Glenn Dale CDP | 69.6% | 41.8% |
| Washington, DC--VA--MD | Great Falls CDP | 67.7% | 41.8% |
| Washington, DC--VA--MD | Greenbelt city | 48.1% | 41.8% |
| Washington, DC--VA--MD | Greenbriar CDP | 27.4% | 41.8% |
| Washington, DC--VA--MD | Groveton CDP | 40.8% | 41.8% |
| Washington, DC--VA--MD | Hamilton town | 54.2% | 41.8% |
| Washington, DC--VA--MD | Hayfield CDP | 51.0% | 41.8% |
| Washington, DC--VA--MD | Haymarket town | 33.3% | 41.8% |
| Washington, DC--VA--MD | Herndon town | 33.9% | 41.8% |
| Washington, DC--VA--MD | Hillandale CDP | 64.7% | 41.8% |
| Washington, DC--VA--MD | Hillcrest Heights CDP | 37.0% | 41.8% |
| Washington, DC--VA--MD | Huntington CDP | 31.9% | 41.8% |
| Washington, DC--VA--MD | Hyattsville city | 37.2% | 41.8% |
| Washington, DC--VA--MD | Hybla Valley CDP | 44.5% | 41.8% |
| Washington, DC--VA--MD | Idylwood CDP | 43.7% | 41.8% |
| Washington, DC--VA--MD | Independent Hill CDP | 64.5% | 41.8% |
| Washington, DC--VA--MD | Kemp Mill CDP | 47.3% | 41.8% |
| Washington, DC--VA--MD | Kensington town | 51.6% | 41.8% |
| Washington, DC--VA--MD | Kettering CDP | 42.8% | 41.8% |
| Washington, DC--VA--MD | Kings Park CDP | 62.4% | 41.8% |
| Washington, DC--VA--MD | Kings Park West CDP | 51.5% | 41.8% |
| Washington, DC--VA--MD | Kingstowne CDP | 51.2% | 41.8% |
| Washington, DC--VA--MD | Konterra CDP | 58.3% | 41.8% |
| Washington, DC--VA--MD | Lake Arbor CDP | 51.5% | 41.8% |
| Washington, DC--VA--MD | Lake Barcroft CDP | 61.2% | 41.8% |
| Washington, DC--VA--MD | Lake Ridge CDP | 46.6% | 41.8% |
| Washington, DC--VA--MD | Landover CDP | 47.6% | 41.8% |
| Washington, DC--VA--MD | Landover Hills town | 57.3% | 41.8% |
| Washington, DC--VA--MD | Langley Park CDP | 20.0% | 41.8% |
| Washington, DC--VA--MD | Lanham CDP | 54.9% | 41.8% |
| Washington, DC--VA--MD | Lansdowne CDP | 34.3% | 41.8% |
| Washington, DC--VA--MD | Largo CDP | 42.3% | 41.8% |
| Washington, DC--VA--MD | Laurel Hill CDP | 35.0% | 41.8% |
| Washington, DC--VA--MD | Layhill CDP | 60.4% | 41.8% |
| Washington, DC--VA--MD | Laytonsville town | 72.1% | 41.8% |
| Washington, DC--VA--MD | Leesburg town | 32.9% | 41.8% |
| Washington, DC--VA--MD | Leisure World CDP | 33.5% | 41.8% |
| Washington, DC--VA--MD | Lincolnia CDP | 37.0% | 41.8% |
| Washington, DC--VA--MD | Linton Hall CDP | 34.4% | 41.8% |
| Washington, DC--VA--MD | Loch Lomond CDP | 38.7% | 41.8% |
| Washington, DC--VA--MD | Long Branch CDP | 55.4% | 41.8% |
| Washington, DC--VA--MD | Lorton CDP | 36.9% | 41.8% |
| Washington, DC--VA--MD | Loudoun Valley Estates CDP | 29.3% | 41.8% |
| Washington, DC--VA--MD | Lowes Island CDP | 37.7% | 41.8% |
| Washington, DC--VA--MD | Manassas Park city | 36.1% | 41.8% |
| Washington, DC--VA--MD | Manassas city | 35.7% | 41.8% |
| Washington, DC--VA--MD | Mantua CDP | 65.6% | 41.8% |
| Washington, DC--VA--MD | Marlboro Meadows CDP | 65.7% | 41.8% |
| Washington, DC--VA--MD | Marlboro Village CDP | 52.2% | 41.8% |
| Washington, DC--VA--MD | Marlow Heights CDP | 34.4% | 41.8% |
| Washington, DC--VA--MD | Marlton CDP | 59.3% | 41.8% |
| Washington, DC--VA--MD | Martin's Additions village | 60.6% | 41.8% |
| Washington, DC--VA--MD | Marumsco CDP | 41.4% | 41.8% |
| Washington, DC--VA--MD | Mason Neck CDP | 74.2% | 41.8% |
| Washington, DC--VA--MD | McLean CDP | 59.9% | 41.8% |
| Washington, DC--VA--MD | McNair CDP | 13.7% | 41.8% |
| Washington, DC--VA--MD | Melwood CDP | 52.2% | 41.8% |
| Washington, DC--VA--MD | Merrifield CDP | 24.9% | 41.8% |
| Washington, DC--VA--MD | Mitchellville CDP | 56.9% | 41.8% |
| Washington, DC--VA--MD | Montclair CDP | 51.4% | 41.8% |
| Washington, DC--VA--MD | Montgomery Village CDP | 48.8% | 41.8% |
| Washington, DC--VA--MD | Moorefield Station CDP | 0.0% | 41.8% |
| Washington, DC--VA--MD | Morningside town | 71.0% | 41.8% |
| Washington, DC--VA--MD | Mount Airy town | 51.5% | 41.8% |
| Washington, DC--VA--MD | Mount Rainier city | 31.3% | 41.8% |
| Washington, DC--VA--MD | Mount Vernon CDP | 54.5% | 41.8% |
| Washington, DC--VA--MD | National Harbor CDP | 45.2% | 41.8% |
| Washington, DC--VA--MD | Neabsco CDP | 39.9% | 41.8% |
| Washington, DC--VA--MD | New Baltimore CDP | 59.6% | 41.8% |
| Washington, DC--VA--MD | New Carrollton city | 48.3% | 41.8% |
| Washington, DC--VA--MD | Newington CDP | 57.5% | 41.8% |
| Washington, DC--VA--MD | Newington Forest CDP | 65.1% | 41.8% |
| Washington, DC--VA--MD | Nokesville CDP | 56.3% | 41.8% |
| Washington, DC--VA--MD | North Bethesda CDP | 43.1% | 41.8% |
| Washington, DC--VA--MD | North Brentwood town | 32.7% | 41.8% |
| Washington, DC--VA--MD | North Chevy Chase village | 70.7% | 41.8% |
| Washington, DC--VA--MD | North Kensington CDP | 52.4% | 41.8% |
| Washington, DC--VA--MD | North Potomac CDP | 56.4% | 41.8% |
| Washington, DC--VA--MD | North Springfield CDP | 47.0% | 41.8% |
| Washington, DC--VA--MD | Oak Grove CDP | 14.5% | 41.8% |
| Washington, DC--VA--MD | Oakton CDP | 42.8% | 41.8% |
| Washington, DC--VA--MD | Occoquan town | 48.4% | 41.8% |
| Washington, DC--VA--MD | Olney CDP | 60.5% | 41.8% |
| Washington, DC--VA--MD | Oxon Hill CDP | 42.5% | 41.8% |
| Washington, DC--VA--MD | Peppermill Village CDP | 46.2% | 41.8% |
| Washington, DC--VA--MD | Pimmit Hills CDP | 45.1% | 41.8% |
| Washington, DC--VA--MD | Potomac CDP | 64.7% | 41.8% |
| Washington, DC--VA--MD | Potomac Mills CDP | 40.8% | 41.8% |
| Washington, DC--VA--MD | Quantico Base CDP | 70.5% | 41.8% |
| Washington, DC--VA--MD | Quantico town | 16.1% | 41.8% |
| Washington, DC--VA--MD | Queen Anne CDP | 54.5% | 41.8% |
| Washington, DC--VA--MD | Queensland CDP | 63.9% | 41.8% |
| Washington, DC--VA--MD | Ravensworth CDP | 53.5% | 41.8% |
| Washington, DC--VA--MD | Redland CDP | 50.5% | 41.8% |
| Washington, DC--VA--MD | Reston CDP | 53.9% | 41.8% |
| Washington, DC--VA--MD | Riverdale Park town | 50.3% | 41.8% |
| Washington, DC--VA--MD | Rockville city | 43.2% | 41.8% |
| Washington, DC--VA--MD | Rosaryville CDP | 42.6% | 41.8% |
| Washington, DC--VA--MD | Rose Hill CDP | 52.7% | 41.8% |
| Washington, DC--VA--MD | Seabrook CDP | 40.1% | 41.8% |
| Washington, DC--VA--MD | Seat Pleasant city | 46.8% | 41.8% |
| Washington, DC--VA--MD | Seven Corners CDP | 42.5% | 41.8% |
| Washington, DC--VA--MD | Silver Hill CDP | 14.1% | 41.8% |
| Washington, DC--VA--MD | Silver Spring CDP | 39.5% | 41.8% |
| Washington, DC--VA--MD | Somerset town | 65.4% | 41.8% |
| Washington, DC--VA--MD | South Kensington CDP | 57.3% | 41.8% |
| Washington, DC--VA--MD | South Riding CDP | 28.7% | 41.8% |
| Washington, DC--VA--MD | South Run CDP | 68.5% | 41.8% |
| Washington, DC--VA--MD | Spencerville CDP | 67.9% | 41.8% |
| Washington, DC--VA--MD | Springdale CDP | 63.4% | 41.8% |
| Washington, DC--VA--MD | Springfield CDP | 40.6% | 41.8% |
| Washington, DC--VA--MD | Stafford Courthouse CDP | 53.9% | 41.8% |
| Washington, DC--VA--MD | Sterling CDP | 28.8% | 41.8% |
| Washington, DC--VA--MD | Stone Ridge CDP | 16.7% | 41.8% |
| Washington, DC--VA--MD | Sudley CDP | 30.0% | 41.8% |
| Washington, DC--VA--MD | Sugarland Run CDP | 36.6% | 41.8% |
| Washington, DC--VA--MD | Suitland CDP | 35.6% | 41.8% |
| Washington, DC--VA--MD | Summerfield CDP | 49.1% | 41.8% |
| Washington, DC--VA--MD | Takoma Park city | 59.6% | 41.8% |
| Washington, DC--VA--MD | Temple Hills CDP | 45.2% | 41.8% |
| Washington, DC--VA--MD | Travilah CDP | 68.6% | 41.8% |
| Washington, DC--VA--MD | Triangle CDP | 43.7% | 41.8% |
| Washington, DC--VA--MD | Tysons CDP | 31.2% | 41.8% |
| Washington, DC--VA--MD | University Center CDP | 45.0% | 41.8% |
| Washington, DC--VA--MD | University Park town | 65.5% | 41.8% |
| Washington, DC--VA--MD | Upper Marlboro town | 49.9% | 41.8% |
| Washington, DC--VA--MD | Vienna town | 49.5% | 41.8% |
| Washington, DC--VA--MD | Wakefield CDP | 63.8% | 41.8% |
| Washington, DC--VA--MD | Walker Mill CDP | 49.2% | 41.8% |
| Washington, DC--VA--MD | Warrenton town | 42.7% | 41.8% |
| Washington, DC--VA--MD | Washington Grove town | 85.5% | 41.8% |
| Washington, DC--VA--MD | Washington city | 20.9% | 41.8% |
| Washington, DC--VA--MD | West Falls Church CDP | 41.9% | 41.8% |
| Washington, DC--VA--MD | West Springfield CDP | 53.0% | 41.8% |
| Washington, DC--VA--MD | Westphalia CDP | 60.1% | 41.8% |
| Washington, DC--VA--MD | Wheaton CDP | 37.4% | 41.8% |
| Washington, DC--VA--MD | White Oak CDP | 43.2% | 41.8% |
| Washington, DC--VA--MD | Wolf Trap CDP | 67.0% | 41.8% |
| Washington, DC--VA--MD | Woodbridge CDP | 42.1% | 41.8% |
| Washington, DC--VA--MD | Woodburn CDP | 46.7% | 41.8% |
| Washington, DC--VA--MD | Woodlawn CDP | 43.2% | 41.8% |
| Washington, DC--VA--MD | Woodmore CDP | 68.1% | 41.8% |
| Washington, DC--VA--MD | Yorkshire CDP | 54.0% | 41.8% |
| Wichita, KS | Andover city | 31.3% | 29.7% |
| Wichita, KS | Bel Aire city | 24.9% | 29.7% |
| Wichita, KS | Derby city | 32.4% | 29.7% |
| Wichita, KS | Eastborough city | 50.5% | 29.7% |
| Wichita, KS | Goddard city | 17.1% | 29.7% |
| Wichita, KS | Haysville city | 22.0% | 29.7% |
| Wichita, KS | Kechi city | 27.2% | 29.7% |
| Wichita, KS | Maize city | 26.5% | 29.7% |
| Wichita, KS | McConnell AFB CDP | 13.7% | 29.7% |
| Wichita, KS | Mulvane city | 33.1% | 29.7% |
| Wichita, KS | Oaklawn-Sunview CDP | 46.2% | 29.7% |
| Wichita, KS | Park City city | 28.2% | 29.7% |
| Wichita, KS | Valley Center city | 33.2% | 29.7% |
| Wichita, KS | Wichita city | 29.8% | 29.7% |
| Winston-Salem, NC | Bermuda Run town | 36.9% | 60.3% |
| Winston-Salem, NC | Bethania town | 82.6% | 60.3% |
| Winston-Salem, NC | Clemmons village | 57.4% | 60.3% |
| Winston-Salem, NC | Germanton CDP | 58.2% | 60.3% |
| Winston-Salem, NC | Kernersville town | 47.0% | 60.3% |
| Winston-Salem, NC | King city | 57.4% | 60.3% |
| Winston-Salem, NC | Lewisville town | 68.5% | 60.3% |
| Winston-Salem, NC | Lexington city | 57.5% | 60.3% |
| Winston-Salem, NC | Midway town | 65.0% | 60.3% |
| Winston-Salem, NC | Oak Ridge town | 66.5% | 60.3% |
| Winston-Salem, NC | Rural Hall town | 57.9% | 60.3% |
| Winston-Salem, NC | Tobaccoville village | 60.8% | 60.3% |
| Winston-Salem, NC | Walkertown town | 55.6% | 60.3% |
| Winston-Salem, NC | Wallburg town | 65.1% | 60.3% |
| Winston-Salem, NC | Welcome CDP | 69.4% | 60.3% |
| Winston-Salem, NC | Winston-Salem city | 61.6% | 60.3% |
| Worcester, MA--CT | Clinton CDP | 25.8% | 47.7% |
| Worcester, MA--CT | Danielson borough | 39.3% | 47.7% |
| Worcester, MA--CT | East Brooklyn CDP | 74.1% | 47.7% |
| Worcester, MA--CT | East Douglas CDP | 68.4% | 47.7% |
| Worcester, MA--CT | Fiskdale CDP | 75.5% | 47.7% |
| Worcester, MA--CT | Moosup CDP | 59.5% | 47.7% |
| Worcester, MA--CT | North Grosvenor Dale CDP | 47.2% | 47.7% |
| Worcester, MA--CT | Northborough CDP | 52.9% | 47.7% |
| Worcester, MA--CT | Oxford CDP | 58.3% | 47.7% |
| Worcester, MA--CT | Putnam CDP | 58.9% | 47.7% |
| Worcester, MA--CT | Quinebaug CDP | 67.1% | 47.7% |
| Worcester, MA--CT | Rutland CDP | 51.4% | 47.7% |
| Worcester, MA--CT | South Lancaster CDP | 59.6% | 47.7% |
| Worcester, MA--CT | South Woodstock CDP | 84.3% | 47.7% |
| Worcester, MA--CT | Southbridge Town city | 36.3% | 47.7% |
| Worcester, MA--CT | Spencer CDP | 46.8% | 47.7% |
| Worcester, MA--CT | Sturbridge CDP | 74.2% | 47.7% |
| Worcester, MA--CT | Upton CDP | 54.8% | 47.7% |
| Worcester, MA--CT | Wauregan CDP | 57.8% | 47.7% |
| Worcester, MA--CT | Webster CDP | 42.2% | 47.7% |
| Worcester, MA--CT | Westborough CDP | 37.4% | 47.7% |
| Worcester, MA--CT | Whitinsville CDP | 62.0% | 47.7% |
| Worcester, MA--CT | Worcester city | 26.4% | 47.7% |
| Youngstown, OH--PA | Austintown CDP | 16.5% | 18.0% |
| Youngstown, OH--PA | Boardman CDP | 20.0% | 18.0% |
| Youngstown, OH--PA | Bolindale CDP | 22.6% | 18.0% |
| Youngstown, OH--PA | Brookfield Center CDP | 19.6% | 18.0% |
| Youngstown, OH--PA | Campbell city | 10.9% | 18.0% |
| Youngstown, OH--PA | Canfield city | 30.7% | 18.0% |
| Youngstown, OH--PA | Champion Heights CDP | 27.6% | 18.0% |
| Youngstown, OH--PA | Churchill CDP | 39.9% | 18.0% |
| Youngstown, OH--PA | Clark borough | 13.2% | 18.0% |
| Youngstown, OH--PA | Cortland city | 33.1% | 18.0% |
| Youngstown, OH--PA | Farrell city | 4.2% | 18.0% |
| Youngstown, OH--PA | Girard city | 17.5% | 18.0% |
| Youngstown, OH--PA | Hermitage city | 17.8% | 18.0% |
| Youngstown, OH--PA | Hilltop CDP | 27.1% | 18.0% |
| Youngstown, OH--PA | Howland Center CDP | 37.8% | 18.0% |
| Youngstown, OH--PA | Hubbard city | 14.5% | 18.0% |
| Youngstown, OH--PA | Leavittsburg CDP | 45.6% | 18.0% |
| Youngstown, OH--PA | Lowellville village | 8.5% | 18.0% |
| Youngstown, OH--PA | Maplewood Park CDP | 32.7% | 18.0% |
| Youngstown, OH--PA | Masury CDP | 16.9% | 18.0% |
| Youngstown, OH--PA | McDonald village | 15.2% | 18.0% |
| Youngstown, OH--PA | McKinley Heights CDP | 22.9% | 18.0% |
| Youngstown, OH--PA | Mineral Ridge CDP | 25.8% | 18.0% |
| Youngstown, OH--PA | Morgandale CDP | 23.8% | 18.0% |
| Youngstown, OH--PA | New Middletown village | 16.7% | 18.0% |
| Youngstown, OH--PA | Newton Falls village | 27.9% | 18.0% |
| Youngstown, OH--PA | Niles city | 15.6% | 18.0% |
| Youngstown, OH--PA | Poland village | 26.2% | 18.0% |
| Youngstown, OH--PA | Sharon city | 5.4% | 18.0% |
| Youngstown, OH--PA | Sharpsville borough | 7.6% | 18.0% |
| Youngstown, OH--PA | South Canal CDP | 44.0% | 18.0% |
| Youngstown, OH--PA | Struthers city | 9.8% | 18.0% |
| Youngstown, OH--PA | Warren city | 16.2% | 18.0% |
| Youngstown, OH--PA | West Hill CDP | 13.7% | 18.0% |
| Youngstown, OH--PA | West Middlesex borough | 15.1% | 18.0% |
| Youngstown, OH--PA | Wheatland borough | 11.3% | 18.0% |
| Youngstown, OH--PA | Youngstown city | 14.1% | 18.0% |
