# Supplementary material for: The tree cover and temperature disparity in US urbanized areas: Quantifying the association with income across 5,723 communities
Source: PLoS One. 2021 Apr 28;16(4):e0249715. doi: 10.1371/journal.pone.0249715 (PMC8081227; doi:10.1371/journal.pone.0249715)
Supplement: S4 Table — Table is sorted by the interquartile range, so urbanized areas with the greatest tree cover gap between low- and high-income census blocks are on top. Also shown is the surface temperature difference between low- and high-income blocks. (DOCX) [file pone.0249715.s007.docx]

|  | **Forest cover (%)** | | |  | **Temperature (Celsius)** |
| --- | --- | --- | --- | --- | --- |
| **Urbanized Area** | ***Poorest census blocks*** | ***City median*** | ***Richest census blocks*** | ***Inter-quartile Range*** | ***Difference (Poorest - richest blocks)*** |
| Bridgeport--Stamford, CT--NY | 14.8% | 44.5% | 69.0% | 54.2% | 4.8 |
| Baltimore, MD | 21.3% | 39.9% | 61.5% | 40.2% | 3.0 |
| New Haven, CT | 20.9% | 38.9% | 61.0% | 40.1% | 3.0 |
| Worcester, MA--CT | 21.0% | 47.7% | 60.3% | 39.3% | 4.1 |
| Hartford, CT | 18.0% | 39.1% | 57.0% | 39.0% | 3.9 |
| Springfield, MA--CT | 18.7% | 36.0% | 54.8% | 36.1% | 3.1 |
| Philadelphia, PA--NJ--DE--MD | 4.6% | 22.2% | 40.1% | 35.5% | 4.1 |
| Boston, MA--NH--RI | 13.7% | 32.3% | 48.8% | 35.1% | 3.7 |
| Poughkeepsie--Newburgh, NY--NJ | 21.6% | 44.6% | 53.4% | 31.8% | 3.4 |
| Providence, RI--MA | 17.5% | 29.3% | 46.8% | 29.3% | 5.4 |
| Milwaukee, WI | 19.5% | 31.8% | 48.8% | 29.3% | 2.5 |
| Albany--Schenectady, NY | 11.1% | 24.6% | 40.0% | 28.9% | 3.1 |
| Allentown, PA--NJ | 3.2% | 16.6% | 31.8% | 28.6% | 3.0 |
| Austin, TX | 26.0% | 35.6% | 51.6% | 25.6% | 1.3 |
| Lancaster, PA | 20.6% | 36.9% | 46.0% | 25.4% | 2.6 |
| Buffalo, NY | 8.6% | 16.8% | 32.4% | 23.8% | 2.1 |
| Grand Rapids, MI | 40.6% | 49.8% | 61.7% | 21.1% | 1.7 |
| Cleveland, OH | 12.9% | 21.3% | 33.0% | 20.2% | 2.5 |
| Detroit, MI | 24.6% | 29.9% | 44.6% | 19.9% | 2.2 |
| Rochester, NY | 16.6% | 24.6% | 36.3% | 19.7% | 2.0 |
| Birmingham, AL | 51.9% | 64.1% | 70.9% | 19.0% | 1.8 |
| New York--Newark, NY--NJ--CT | 9.7% | 16.0% | 28.3% | 18.6% | 1.9 |
| Portland, OR--WA | 20.1% | 26.8% | 38.4% | 18.4% | 2.2 |
| Pittsburgh, PA | 21.8% | 31.3% | 39.8% | 17.9% | 2.4 |
| Concord, CA | 9.2% | 13.9% | 27.1% | 17.9% | 2.3 |
| Syracuse, NY | 24.1% | 35.7% | 41.9% | 17.9% | 2.1 |
| Cincinnati, OH--KY--IN | 37.4% | 46.3% | 54.6% | 17.2% | 1.5 |
| Harrisburg, PA | 30.4% | 36.3% | 46.2% | 15.8% | 2.1 |
| Toledo, OH--MI | 12.6% | 21.3% | 28.0% | 15.4% | 1.4 |
| Youngstown, OH--PA | 13.1% | 18.0% | 28.0% | 14.9% | 1.5 |
| Dayton, OH | 18.4% | 24.5% | 32.6% | 14.2% | 1.4 |
| Seattle, WA | 26.1% | 35.2% | 39.5% | 13.4% | 2.0 |
| Minneapolis--St. Paul, MN--WI | 39.4% | 47.5% | 52.8% | 13.4% | 1.5 |
| Indianapolis, IN | 19.5% | 24.0% | 32.6% | 13.0% | 0.8 |
| Miami, FL | 24.5% | 30.2% | 37.5% | 13.0% | 1.4 |
| Flint, MI | 49.0% | 53.7% | 61.9% | 12.8% | 1.7 |
| Richmond, VA | 42.9% | 52.2% | 55.6% | 12.8% | 1.1 |
| Chattanooga, TN--GA | 53.5% | 61.6% | 66.0% | 12.5% | 1.9 |
| Chicago, IL--IN | 23.1% | 27.1% | 35.6% | 12.5% | 1.6 |
| San Antonio, TX | 26.7% | 29.6% | 39.1% | 12.4% | 1.2 |
| Columbia, SC | 36.8% | 44.2% | 49.0% | 12.2% | 1.2 |
| Nashville-Davidson, TN | 56.4% | 62.5% | 68.4% | 11.9% | 0.6 |
| St. Louis, MO--IL | 30.2% | 37.0% | 42.1% | 11.9% | 1.2 |
| Orlando, FL | 37.1% | 43.5% | 48.9% | 11.8% | 0.8 |
| Akron, OH | 20.9% | 26.6% | 32.7% | 11.8% | 1.9 |
| Augusta-Richmond County, GA--SC | 43.7% | 48.1% | 55.1% | 11.4% | 1.2 |
| Hickory, NC | 48.7% | 53.5% | 59.8% | 11.1% | 0.1 |
| Washington, DC--VA--MD | 35.2% | 41.8% | 46.2% | 11.1% | 0.8 |
| Columbus, OH | 9.9% | 13.0% | 20.9% | 11.0% | 1.4 |
| Louisville/Jefferson County, KY--IN | 32.3% | 34.9% | 42.9% | 10.6% | 1.2 |
| Port St. Lucie, FL | 36.6% | 41.2% | 47.2% | 10.6% | 1.2 |
| Raleigh, NC | 53.6% | 56.7% | 63.7% | 10.1% | 0.5 |
| San Diego, CA | 3.3% | 5.9% | 12.8% | 9.5% | 2.4 |
| Virginia Beach, VA | 28.1% | 32.4% | 37.5% | 9.5% | 1.1 |
| Winston-Salem, NC | 56.4% | 60.3% | 65.8% | 9.4% | 1.1 |
| San Francisco--Oakland, CA | 2.3% | 4.7% | 11.4% | 9.1% | 1.9 |
| Riverside--San Bernardino, CA | 20.5% | 23.9% | 29.4% | 8.9% | 2.5 |
| Asheville, NC | 53.9% | 59.0% | 62.5% | 8.6% | 0.3 |
| Sacramento, CA | 20.4% | 22.9% | 28.7% | 8.2% | 1.5 |
| Kansas City, MO--KS | 34.0% | 36.6% | 42.2% | 8.2% | 0.2 |
| Houston, TX | 24.3% | 28.1% | 32.5% | 8.1% | 0.6 |
| Charlotte, NC--SC | 52.6% | 55.7% | 60.7% | 8.1% | 0.3 |
| San Jose, CA | 2.7% | 4.9% | 10.8% | 8.1% | 2.5 |
| Denver--Aurora, CO | 18.5% | 21.8% | 26.3% | 7.9% | 1.6 |
| Memphis, TN--MS--AR | 51.3% | 53.5% | 59.1% | 7.8% | 0.5 |
| Sarasota--Bradenton, FL | 25.9% | 29.5% | 32.8% | 6.9% | 0.9 |
| Omaha, NE--IA | 8.1% | 11.7% | 15.0% | 6.9% | 0.7 |
| New Orleans, LA | 38.2% | 40.8% | 44.8% | 6.6% | 0.0 |
| Barnstable Town, MA | 51.4% | 56.5% | 57.8% | 6.4% | 1.0 |
| Ogden--Layton, UT | 16.2% | 17.7% | 22.4% | 6.1% | 1.6 |
| Los Angeles--Long Beach--Anaheim, CA | 16.8% | 17.2% | 22.9% | 6.1% | 3.1 |
| Wichita, KS | 28.9% | 29.7% | 34.8% | 5.9% | 0.6 |
| Bonita Springs, FL | 18.8% | 22.7% | 24.3% | 5.6% | 1.1 |
| Pensacola, FL--AL | 38.1% | 43.3% | 43.6% | 5.5% | 0.4 |
| Greenville, SC | 32.8% | 37.1% | 38.0% | 5.3% | 0.7 |
| Baton Rouge, LA | 58.1% | 63.3% | 63.3% | 5.3% | 0.3 |
| Tampa--St. Petersburg, FL | 31.7% | 33.5% | 36.7% | 5.1% | 0.7 |
| Jacksonville, FL | 42.0% | 44.0% | 46.5% | 4.5% | 1.0 |
| Phoenix--Mesa, AZ | 6.8% | 8.9% | 10.7% | 3.9% | 2.1 |
| Tulsa, OK | 9.0% | 9.8% | 12.9% | 3.9% | 0.5 |
| Huntsville, AL | 49.9% | 51.8% | 53.5% | 3.7% | 1.0 |
| Myrtle Beach--Socastee, SC--NC | 37.8% | 41.2% | 41.1% | 3.4% | 0.2 |
| Atlanta, GA | 60.2% | 62.0% | 62.9% | 2.7% | 0.6 |
| Las Vegas--Henderson, NV | 5.3% | 6.0% | 8.0% | 2.7% | 2.3 |
| Tucson, AZ | 3.4% | 4.7% | 6.0% | 2.6% | 0.5 |
| El Paso, TX--NM | 4.6% | 5.4% | 6.6% | 2.0% | 0.3 |
| Albuquerque, NM | 2.8% | 3.7% | 4.7% | 1.8% | 1.1 |
| Mobile, AL | 59.3% | 60.1% | 61.0% | 1.7% | 0.0 |
| Salt Lake City--West Valley City, UT | 19.9% | 20.9% | 21.5% | 1.6% | 1.5 |
| Knoxville, TN | 61.8% | 63.4% | 63.2% | 1.4% | 0.7 |
| McAllen, TX | 6.9% | 6.9% | 7.6% | 0.7% | -0.5 |
| Fayetteville, NC | 49.0% | 48.5% | 49.4% | 0.4% | 0.0 |
| Little Rock, AR | 67.4% | 67.4% | 67.0% | -0.3% | 0.2 |
| Dallas--Fort Worth--Arlington, TX | 29.7% | 26.2% | 27.9% | -1.8% | 0.6 |
| Oklahoma City, OK | 25.4% | 22.3% | 23.0% | -2.4% | 0.3 |
| Charleston--North Charleston, SC | 37.3% | 39.7% | 34.4% | -2.9% | 0.8 |
| Des Moines, IA | 34.1% | 29.9% | 28.4% | -5.7% | -0.2 |
| Palm Bay--Melbourne, FL | 38.1% | 35.3% | 31.5% | -6.6% | 0.0 |
| Jackson, MS | 62.2% | 56.3% | 55.6% | -6.6% | 0.0 |
| Cape Coral, FL | 25.8% | 19.0% | 18.4% | -7.5% | 0.6 |
