# Supplementary material for: The tree cover and temperature disparity in US urbanized areas: Quantifying the association with income across 5,723 communities
Source: PLoS One. 2021 Apr 28;16(4):e0249715. doi: 10.1371/journal.pone.0249715 (PMC8081227; doi:10.1371/journal.pone.0249715)
Supplement: S5 Table — For each urbanized area, we separately ran a regression predicting tree cover (arcsin transformed) as a function of population density category, income category, built-up intensity (BUI) category, and majority white status. Our statistical analysis included census blocks in all four income categories (quantiles, defined within each urbanized area). In this table we show the effect on tree cover of the highest category in each group relative to the baseline (the lowest category). For instance, for Akron, OH the highest population density category had 0.30 fewer units of tree cover (arcsine transformed) than the lowest population density category. Significant effects (one-tailed, P<0.05) are shown with an asterisk. Note that for some urbanized areas, there were no census blocks in the highest BUI category. At the median tree cover nationally (27%) a 0.1, 0.2, and 0.3 increase in arcsine transformed tree cover equaled an increase of 9.3%,19.1%, and 29.1% in tree cover, respectively. (DOCX) [file pone.0249715.s008.docx]

|  | **Effect on forest cover (arcsin transformed) of highest category compared to baseline.** | | | |
| --- | --- | --- | --- | --- |
| **Urbanized area** | ***Population Density*** | ***Income*** | ***Building Use Intensity (BUI)*** | ***Majority White*** |
| Akron, OH | -0.30* | 0.05* | -0.06* | -0.03 |
| Albany--Schenectady, NY | -0.24* | 0.14* | -0.08* | 0.01 |
| Albuquerque, NM | -0.03* | 0.04* | 0.04 | 0.03* |
| Allentown, PA--NJ | -0.25* | 0.16* | -0.10* | 0.01* |
| Asheville, NC | -0.31* | 0.13* | -0.08* | 0.04* |
| Atlanta, GA | -0.35* | 0.01* | -0.02* | 0.01* |
| Augusta-Richmond County, GA--SC | -0.35* | 0.04* | -0.10* | 0.05* |
| Austin, TX | -0.12* | 0.16* | 0.05 | 0.06* |
| Baltimore, MD | -0.33* | 0.15* | -0.14* | -0.03 |
| Barnstable Town, MA | -0.48* | -0.01 | -0.17* | 0.08* |
| Baton Rouge, LA | -0.27* | 0.03* | -0.21* | 0.03* |
| Birmingham, AL | -0.30* | 0.12* | -0.08* | 0.06* |
| Bonita Springs, FL | -0.10* | 0.10* | -0.07* | -0.08 |
| Boston, MA--NH--RI | -0.39* | 0.11* | -0.20* | 0.01* |
| Bridgeport--Stamford, CT--NY | -0.31* | 0.12* | -0.30* | 0.11* |
| Buffalo, NY | -0.18* | 0.15* | -0.22* | -0.01 |
| Cape Coral, FL | -0.15* | -0.12 | -0.08* | -0.02 |
| Charleston--North Charleston, SC | -0.24* | 0.01 | -0.18* | 0.01 |
| Charlotte, NC--SC | -0.38* | 0.03* | -0.02* | 0.04* |
| Chattanooga, TN--GA | -0.33* | 0.03* | -0.23* | 0.02* |
| Chicago, IL--IN | -0.15* | 0.10* | -0.01* | 0.03* |
| Cincinnati, OH--KY--IN | -0.28* | 0.12* | -0.06* | -0.03 |
| Cleveland, OH | -0.24* | 0.07* | -0.08* | 0.04* |
| Columbia, SC | -0.30* | 0.04* | 0.06 | 0.03* |
| Columbus, OH | -0.25* | 0.05* | 0.00 | 0.03* |
| Concord, CA | -0.14* | 0.18* | -0.01 | 0.09* |
| Dallas--Fort Worth--Arlington, TX | -0.20* | -0.06 | 0.05 | 0.06* |
| Dayton, OH | -0.30* | 0.08* | -0.10* | 0.03* |
| Denver--Aurora, CO | -0.07* | 0.06* | 0.14 | 0.05* |
| Des Moines, IA | -0.07* | -0.02 | 0.00 | 0.05* |
| Detroit, MI | -0.31* | 0.16* | -0.06* | -0.03 |
| El Paso, TX--NM | -0.08* | 0.04* | 0.02 | 0.07* |
| Fayetteville, NC | -0.40* | -0.03 | NA | 0.04* |
| Flint, MI | -0.38* | 0.07* | NA | -0.02 |
| Grand Rapids, MI | -0.32* | 0.17* | -0.03* | 0.04* |
| Greenville, SC | -0.18* | 0.04* | NA | 0.02* |
| Harrisburg, PA | -0.11* | 0.11* | 0.01 | -0.02 |
| Hartford, CT | -0.35* | 0.07* | -0.18* | 0.03* |
| Hickory, NC | -0.37* | 0.09* | -0.27* | 0.01 |
| Houston, TX | -0.18* | -0.02 | 0.02 | 0.10* |
| Huntsville, AL | -0.36* | 0.01 | -0.03* | 0.02* |
| Indianapolis, IN | -0.34* | 0.04* | -0.06* | -0.01 |
| Jackson, MS | -0.39* | -0.03 | NA | -0.05 |
| Jacksonville, FL | -0.29* | -0.05 | -0.16* | 0.00 |
| Kansas City, MO--KS | -0.28* | 0.06* | 0.04 | 0.04* |
| Knoxville, TN | -0.33* | 0.02* | -0.20* | 0.02* |
| Lancaster, PA | -0.26* | 0.10* | -0.03* | 0.04* |
| Las Vegas--Henderson, NV | 0.00 | 0.04* | 0.08 | 0.04* |
| Little Rock, AR | -0.27* | -0.03 | -0.10* | 0.06* |
| Los Angeles--Long Beach--Anaheim, CA | -0.04* | 0.06* | -0.04* | 0.03* |
| Louisville/Jefferson County, KY--IN | -0.19* | 0.08* | -0.03* | 0.00 |
| McAllen, TX | -0.06* | 0.00 | 0.01 | -0.11 |
| Memphis, TN--MS--AR | -0.24* | 0.00 | -0.03* | 0.04* |
| Miami, FL | -0.14* | 0.01* | -0.08* | 0.11* |
| Milwaukee, WI | -0.30* | 0.13* | -0.07* | 0.00 |
| Minneapolis--St. Paul, MN--WI | -0.35* | 0.04* | -0.07* | 0.03* |
| Mobile, AL | -0.36* | 0.00 | -0.06* | 0.04* |
| Myrtle Beach--Socastee, SC--NC | -0.28* | -0.04 | -0.13* | -0.01 |
| Nashville-Davidson, TN | -0.27* | 0.07* | -0.02* | 0.03* |
| New Haven, CT | -0.29* | 0.07* | -0.15* | 0.00 |
| New Orleans, LA | -0.29* | -0.03 | NA | -0.05 |
| New York--Newark, NY--NJ--CT | -0.26* | 0.06* | -0.13* | 0.02* |
| Ogden--Layton, UT | -0.03* | 0.09* | 0.06 | 0.03* |
| Oklahoma City, OK | -0.16* | -0.01 | -0.05* | -0.03 |
| Omaha, NE--IA | -0.10* | 0.06* | -0.06* | 0.02* |
| Orlando, FL | -0.21* | 0.03* | 0.02 | 0.06* |
| Palm Bay--Melbourne, FL | -0.29* | -0.10 | -0.17* | 0.02* |
| Pensacola, FL--AL | -0.35* | -0.03 | -0.20* | 0.04* |
| Philadelphia, PA--NJ--DE--MD | -0.34* | 0.11* | -0.13* | 0.00 |
| Phoenix--Mesa, AZ | 0.03 | 0.06* | 0.10 | 0.01* |
| Pittsburgh, PA | -0.20* | 0.15* | 0.07 | -0.06 |
| Port St. Lucie, FL | -0.31* | 0.04* | -0.14* | -0.02 |
| Portland, OR--WA | -0.26* | 0.19* | -0.04* | 0.06* |
| Poughkeepsie--Newburgh, NY--NJ | -0.31* | -0.01 | -0.12* | 0.07* |
| Providence, RI--MA | -0.26* | 0.06* | -0.16* | -0.08 |
| Raleigh, NC | -0.42* | 0.04* | 0.01 | 0.07* |
| Richmond, VA | -0.35* | 0.10* | -0.17* | 0.04* |
| Riverside--San Bernardino, CA | 0.01 | 0.09* | 0.01 | 0.07* |
| Rochester, NY | -0.21* | 0.09* | -0.12* | 0.02* |
| Sacramento, CA | 0.04 | 0.09* | 0.06 | 0.06* |
| Salt Lake City--West Valley City, UT | 0.02 | 0.03* | 0.04 | 0.02* |
| San Antonio, TX | -0.12* | 0.09* | 0.02 | 0.09* |
| San Diego, CA | -0.09* | 0.08* | -0.07* | 0.02* |
| San Francisco--Oakland, CA | -0.17* | 0.16* | -0.14* | 0.09* |
| San Jose, CA | -0.09* | 0.14* | -0.15* | 0.08* |
| Sarasota--Bradenton, FL | -0.21* | 0.01 | -0.08* | -0.08 |
| Seattle, WA | -0.25* | 0.16* | -0.07* | 0.02* |
| Springfield, MA--CT | -0.34* | 0.09* | -0.07* | 0.01 |
| St. Louis, MO--IL | -0.04* | 0.10* | -0.09* | 0.02* |
| Syracuse, NY | -0.25* | 0.05* | -0.13* | -0.01 |
| Tampa--St. Petersburg, FL | -0.21* | 0.03* | -0.14* | -0.04 |
| Toledo, OH--MI | -0.25* | 0.14* | 0.04 | 0.05* |
| Tucson, AZ | 0.05 | 0.07* | 0.08 | 0.03* |
| Tulsa, OK | -0.13* | 0.04* | 0.09 | -0.01 |
| Virginia Beach, VA | -0.29* | -0.01 | -0.13* | 0.04* |
| Washington, DC--VA--MD | -0.32* | 0.04* | -0.09* | 0.06* |
| Wichita, KS | -0.23* | 0.11* | -0.05* | -0.01 |
| Winston-Salem, NC | -0.38* | 0.05* | -0.18* | 0.03* |
| Worcester, MA--CT | -0.33* | 0.06* | -0.16* | 0.06* |
