# Supplementary material for: The tree cover and temperature disparity in US urbanized areas: Quantifying the association with income across 5,723 communities
Source: PLoS One. 2021 Apr 28;16(4):e0249715. doi: 10.1371/journal.pone.0249715 (PMC8081227; doi:10.1371/journal.pone.0249715)
Supplement: S6 Table — Shown are data stratified by low- and high-income blocks, by population density class, by urbanized area. Also shown is an estimate of the tree cover gap (%) between low- and high-income blocks, and the needed tree cover to close the gap. (DOCX) [file pone.0249715.s009.docx]

| **Urbanized Area** | **Population density category (people/km^2^)** | **Tree cover (%) in low-income census blocks** | **Tree cover (%) in high-income census blocks** | **Population of low-income census blocks** | **Area (km^2^) of low-income census blocks** | **Forest cover (%) gap** | **Needed forest canopy to close gap (km^2^)** |
| --- | --- | --- | --- | --- | --- | --- | --- |
| Akron, OH | 1 (Very Low: < 2000) | 27.4% | 34.3% | 38,958 | 700.7 | 6.9% | 48.3 |
| Akron, OH | 2 (Low: 2000-4000) | 21.5% | 23.6% | 67,898 | 65.7 | 2.0% | 1.3 |
| Akron, OH | 3 (Medium:4000-8000) | 17.0% | 13.7% | 25,066 | 8.7 | 0.0% | 0.0 |
| Akron, OH | 4 (High: >8000) | 2.6% | 12.0% | 10,562 | 1.0 | 9.4% | 0.1 |
| Albany--Schenectady, NY | 1 (Very Low: < 2000) | 26.3% | 44.5% | 21,267 | 625.2 | 18.2% | 113.8 |
| Albany--Schenectady, NY | 2 (Low: 2000-4000) | 12.8% | 22.8% | 29,763 | 42.1 | 10.0% | 4.2 |
| Albany--Schenectady, NY | 3 (Medium:4000-8000) | 10.4% | 15.7% | 60,776 | 19.5 | 5.3% | 1.0 |
| Albany--Schenectady, NY | 4 (High: >8000) | 7.4% | 8.6% | 37,518 | 5.1 | 1.2% | 0.1 |
| Albuquerque, NM | 1 (Very Low: < 2000) | 2.5% | 4.2% | 48,470 | 387.1 | 1.7% | 6.4 |
| Albuquerque, NM | 2 (Low: 2000-4000) | 3.6% | 5.6% | 59,130 | 113.4 | 2.0% | 2.3 |
| Albuquerque, NM | 3 (Medium:4000-8000) | 2.2% | 2.5% | 59,821 | 25.2 | 0.3% | 0.1 |
| Albuquerque, NM | 4 (High: >8000) | 4.2% | 3.6% | 17,940 | 2.7 | 0.0% | 0.0 |
| Allentown, PA--NJ | 1 (Very Low: < 2000) | 20.7% | 35.0% | 15,819 | 715.3 | 14.3% | 102.4 |
| Allentown, PA--NJ | 2 (Low: 2000-4000) | 7.4% | 20.0% | 21,432 | 54.5 | 12.7% | 6.9 |
| Allentown, PA--NJ | 3 (Medium:4000-8000) | 5.2% | 7.8% | 46,338 | 19.6 | 2.7% | 0.5 |
| Allentown, PA--NJ | 4 (High: >8000) | 1.1% | 3.9% | 83,212 | 8.7 | 2.8% | 0.2 |
| Asheville, NC | 1 (Very Low: < 2000) | 56.4% | 64.3% | 57,087 | 609.5 | 7.9% | 48.1 |
| Asheville, NC | 2 (Low: 2000-4000) | 39.6% | 45.5% | 8,058 | 9.2 | 5.8% | 0.5 |
| Asheville, NC | 3 (Medium:4000-8000) | 28.7% | 32.5% | 3,511 | 1.4 | 3.8% | 0.1 |
| Asheville, NC | 4 (High: >8000) | 13.2% | 8.0% | 1,323 | 0.3 | 0.0% | 0.0 |
| Atlanta, GA | 1 (Very Low: < 2000) | 67.1% | 65.5% | 628,371 | 5,878.4 | 0.0% | 0.0 |
| Atlanta, GA | 2 (Low: 2000-4000) | 56.4% | 54.6% | 293,435 | 270.9 | 0.0% | 0.0 |
| Atlanta, GA | 3 (Medium:4000-8000) | 47.5% | 47.5% | 125,897 | 57.1 | 0.1% | 0.0 |
| Atlanta, GA | 4 (High: >8000) | 34.9% | 28.6% | 87,241 | 14.2 | 0.0% | 0.0 |
| Augusta-Richmond County, GA--SC | 1 (Very Low: < 2000) | 48.4% | 56.2% | 65,519 | 567.7 | 7.9% | 44.6 |
| Augusta-Richmond County, GA--SC | 2 (Low: 2000-4000) | 38.2% | 41.1% | 20,932 | 22.1 | 3.0% | 0.7 |
| Augusta-Richmond County, GA--SC | 3 (Medium:4000-8000) | 22.3% | 25.4% | 4,728 | 2.7 | 3.1% | 0.1 |
| Augusta-Richmond County, GA--SC | 4 (High: >8000) | 15.3% | 70.0% | 5,521 | 0.8 | 54.8% | 0.4 |
| Austin, TX | 1 (Very Low: < 2000) | 24.9% | 56.7% | 91,228 | 942.0 | 31.8% | 299.5 |
| Austin, TX | 2 (Low: 2000-4000) | 31.3% | 47.4% | 102,179 | 189.9 | 16.1% | 30.5 |
| Austin, TX | 3 (Medium:4000-8000) | 29.5% | 37.2% | 93,075 | 39.4 | 7.7% | 3.0 |
| Austin, TX | 4 (High: >8000) | 21.2% | 21.4% | 55,505 | 6.0 | 0.2% | 0.0 |
| Baltimore, MD | 1 (Very Low: < 2000) | 47.0% | 70.1% | 49,783 | 1,238.3 | 23.1% | 285.6 |
| Baltimore, MD | 2 (Low: 2000-4000) | 37.7% | 51.1% | 90,371 | 200.5 | 13.4% | 26.9 |
| Baltimore, MD | 3 (Medium:4000-8000) | 23.2% | 32.3% | 147,715 | 73.0 | 9.2% | 6.7 |
| Baltimore, MD | 4 (High: >8000) | 14.0% | 10.2% | 264,145 | 38.9 | 0.0% | 0.0 |
| Barnstable Town, MA | 1 (Very Low: < 2000) | 54.5% | 57.9% | 53,585 | 641.2 | 3.4% | 21.6 |
| Barnstable Town, MA | 2 (Low: 2000-4000) | 28.9% | 31.1% | 6,509 | 5.3 | 2.3% | 0.1 |
| Barnstable Town, MA | 3 (Medium:4000-8000) | 18.0% | 12.9% | 1,013 | 0.4 | 0.0% | 0.0 |
| Barnstable Town, MA | 4 (High: >8000) | 1.5% | 1.6% | 498 | 0.1 | 0.1% | 0.0 |
| Baton Rouge, LA | 1 (Very Low: < 2000) | 65.8% | 66.5% | 62,803 | 785.7 | 0.7% | 5.6 |
| Baton Rouge, LA | 2 (Low: 2000-4000) | 61.0% | 48.4% | 50,383 | 51.8 | 0.0% | 0.0 |
| Baton Rouge, LA | 3 (Medium:4000-8000) | 44.6% | 44.2% | 23,325 | 7.0 | 0.0% | 0.0 |
| Baton Rouge, LA | 4 (High: >8000) | 28.9% | 40.0% | 15,110 | 1.8 | 11.1% | 0.2 |
| Birmingham, AL | 1 (Very Low: < 2000) | 60.8% | 73.6% | 96,858 | 1,047.3 | 12.8% | 134.2 |
| Birmingham, AL | 2 (Low: 2000-4000) | 50.3% | 58.1% | 53,292 | 49.2 | 7.9% | 3.9 |
| Birmingham, AL | 3 (Medium:4000-8000) | 38.4% | 53.0% | 20,372 | 8.0 | 14.7% | 1.2 |
| Birmingham, AL | 4 (High: >8000) | 22.1% | 31.1% | 16,803 | 2.6 | 9.0% | 0.2 |
| Bonita Springs, FL | 1 (Very Low: < 2000) | 23.8% | 26.7% | 28,987 | 401.5 | 2.9% | 11.8 |
| Bonita Springs, FL | 2 (Low: 2000-4000) | 17.5% | 12.0% | 31,429 | 25.1 | 0.0% | 0.0 |
| Bonita Springs, FL | 3 (Medium:4000-8000) | 16.2% | 6.5% | 12,639 | 3.6 | 0.0% | 0.0 |
| Bonita Springs, FL | 4 (High: >8000) | 18.0% | 19.1% | 5,939 | 1.0 | 1.2% | 0.0 |
| Boston, MA--NH--RI | 1 (Very Low: < 2000) | 53.3% | 67.6% | 141,033 | 3,991.1 | 14.3% | 570.0 |
| Boston, MA--NH--RI | 2 (Low: 2000-4000) | 27.2% | 37.1% | 136,954 | 254.3 | 9.9% | 25.3 |
| Boston, MA--NH--RI | 3 (Medium:4000-8000) | 15.7% | 19.1% | 259,167 | 125.7 | 3.5% | 4.4 |
| Boston, MA--NH--RI | 4 (High: >8000) | 7.6% | 7.7% | 508,319 | 71.7 | 0.1% | 0.1 |
| Bridgeport--Stamford, CT--NY | 1 (Very Low: < 2000) | 44.7% | 73.9% | 22,686 | 1,051.4 | 29.2% | 307.4 |
| Bridgeport--Stamford, CT--NY | 2 (Low: 2000-4000) | 32.5% | 35.7% | 44,083 | 60.5 | 3.2% | 2.0 |
| Bridgeport--Stamford, CT--NY | 3 (Medium:4000-8000) | 16.9% | 13.8% | 76,459 | 27.9 | 0.0% | 0.0 |
| Bridgeport--Stamford, CT--NY | 4 (High: >8000) | 7.6% | 3.2% | 88,104 | 10.7 | 0.0% | 0.0 |
| Buffalo, NY | 1 (Very Low: < 2000) | 12.9% | 43.0% | 26,287 | 705.1 | 30.1% | 212.2 |
| Buffalo, NY | 2 (Low: 2000-4000) | 10.6% | 19.9% | 73,023 | 115.1 | 9.3% | 10.7 |
| Buffalo, NY | 3 (Medium:4000-8000) | 7.3% | 9.6% | 116,339 | 39.8 | 2.3% | 0.9 |
| Buffalo, NY | 4 (High: >8000) | 7.4% | 9.1% | 18,906 | 2.8 | 1.7% | 0.0 |
| Cape Coral, FL | 1 (Very Low: < 2000) | 27.6% | 20.0% | 99,624 | 712.6 | 0.0% | 0.0 |
| Cape Coral, FL | 2 (Low: 2000-4000) | 20.8% | 14.3% | 22,852 | 30.9 | 0.0% | 0.0 |
| Cape Coral, FL | 3 (Medium:4000-8000) | 17.7% | 12.4% | 6,216 | 3.2 | 0.0% | 0.0 |
| Cape Coral, FL | 4 (High: >8000) | 14.4% | 10.6% | 3,564 | 0.7 | 0.0% | 0.0 |
| Charleston--North Charleston, SC | 1 (Very Low: < 2000) | 43.6% | 38.6% | 63,503 | 575.6 | 0.0% | 0.0 |
| Charleston--North Charleston, SC | 2 (Low: 2000-4000) | 38.8% | 25.9% | 41,267 | 49.4 | 0.0% | 0.0 |
| Charleston--North Charleston, SC | 3 (Medium:4000-8000) | 24.3% | 20.6% | 19,468 | 8.9 | 0.0% | 0.0 |
| Charleston--North Charleston, SC | 4 (High: >8000) | 6.7% | 25.5% | 12,274 | 1.6 | 18.8% | 0.3 |
| Charlotte, NC--SC | 1 (Very Low: < 2000) | 58.9% | 63.2% | 188,374 | 1,574.1 | 4.4% | 68.7 |
| Charlotte, NC--SC | 2 (Low: 2000-4000) | 48.4% | 50.4% | 74,493 | 91.0 | 2.0% | 1.8 |
| Charlotte, NC--SC | 3 (Medium:4000-8000) | 35.3% | 24.1% | 38,339 | 14.9 | 0.0% | 0.0 |
| Charlotte, NC--SC | 4 (High: >8000) | 22.6% | 13.5% | 11,697 | 3.4 | 0.0% | 0.0 |
| Chattanooga, TN--GA | 1 (Very Low: < 2000) | 63.4% | 68.2% | 60,210 | 667.9 | 4.8% | 32.1 |
| Chattanooga, TN--GA | 2 (Low: 2000-4000) | 47.6% | 46.6% | 20,555 | 16.6 | 0.0% | 0.0 |
| Chattanooga, TN--GA | 3 (Medium:4000-8000) | 32.2% | 42.2% | 8,961 | 2.3 | 10.0% | 0.2 |
| Chattanooga, TN--GA | 4 (High: >8000) | 17.8% | 13.9% | 5,684 | 0.7 | 0.0% | 0.0 |
| Chicago, IL--IN | 1 (Very Low: < 2000) | 33.8% | 46.9% | 176,326 | 3,371.9 | 13.1% | 442.0 |
| Chicago, IL--IN | 2 (Low: 2000-4000) | 30.0% | 39.8% | 373,882 | 918.5 | 9.8% | 90.2 |
| Chicago, IL--IN | 3 (Medium:4000-8000) | 24.6% | 27.2% | 635,533 | 309.5 | 2.7% | 8.3 |
| Chicago, IL--IN | 4 (High: >8000) | 19.7% | 18.6% | 965,733 | 166.0 | 0.0% | 0.0 |
| Cincinnati, OH--KY--IN | 1 (Very Low: < 2000) | 52.9% | 58.3% | 134,888 | 1,602.2 | 5.5% | 87.4 |
| Cincinnati, OH--KY--IN | 2 (Low: 2000-4000) | 37.4% | 40.5% | 154,190 | 151.9 | 3.0% | 4.6 |
| Cincinnati, OH--KY--IN | 3 (Medium:4000-8000) | 30.3% | 28.4% | 86,891 | 30.0 | 0.0% | 0.0 |
| Cincinnati, OH--KY--IN | 4 (High: >8000) | 20.4% | 24.5% | 30,690 | 4.4 | 4.1% | 0.2 |
| Cleveland, OH | 1 (Very Low: < 2000) | 20.5% | 36.5% | 76,822 | 1,536.7 | 16.0% | 246.0 |
| Cleveland, OH | 2 (Low: 2000-4000) | 13.1% | 22.4% | 172,022 | 192.3 | 9.3% | 17.9 |
| Cleveland, OH | 3 (Medium:4000-8000) | 11.4% | 13.2% | 168,911 | 56.1 | 1.9% | 1.0 |
| Cleveland, OH | 4 (High: >8000) | 8.7% | 0.7% | 28,061 | 5.5 | 0.0% | 0.0 |
| Columbia, SC | 1 (Very Low: < 2000) | 43.7% | 50.4% | 73,941 | 808.3 | 6.7% | 53.9 |
| Columbia, SC | 2 (Low: 2000-4000) | 37.5% | 37.3% | 24,942 | 34.8 | 0.0% | 0.0 |
| Columbia, SC | 3 (Medium:4000-8000) | 17.9% | 25.9% | 18,662 | 5.9 | 8.0% | 0.5 |
| Columbia, SC | 4 (High: >8000) | 12.6% | 19.5% | 20,674 | 2.0 | 6.9% | 0.1 |
| Columbus, OH | 1 (Very Low: < 2000) | 13.1% | 26.0% | 74,936 | 861.5 | 12.9% | 111.4 |
| Columbus, OH | 2 (Low: 2000-4000) | 11.5% | 16.3% | 143,624 | 201.6 | 4.7% | 9.6 |
| Columbus, OH | 3 (Medium:4000-8000) | 8.2% | 5.5% | 75,655 | 35.5 | 0.0% | 0.0 |
| Columbus, OH | 4 (High: >8000) | 3.5% | 1.9% | 48,095 | 7.1 | 0.0% | 0.0 |
| Concord, CA | 1 (Very Low: < 2000) | 11.2% | 32.4% | 22,824 | 319.9 | 21.2% | 67.8 |
| Concord, CA | 2 (Low: 2000-4000) | 10.2% | 16.3% | 62,916 | 76.3 | 6.1% | 4.6 |
| Concord, CA | 3 (Medium:4000-8000) | 8.3% | 12.7% | 39,651 | 18.3 | 4.5% | 0.8 |
| Concord, CA | 4 (High: >8000) | 4.6% | 14.1% | 27,654 | 4.8 | 9.4% | 0.4 |
| Dallas--Fort Worth--Arlington, TX | 1 (Very Low: < 2000) | 36.7% | 38.1% | 259,604 | 2,592.3 | 1.4% | 37.0 |
| Dallas--Fort Worth--Arlington, TX | 2 (Low: 2000-4000) | 36.8% | 23.5% | 490,578 | 752.4 | 0.0% | 0.0 |
| Dallas--Fort Worth--Arlington, TX | 3 (Medium:4000-8000) | 24.0% | 18.6% | 294,039 | 150.7 | 0.0% | 0.0 |
| Dallas--Fort Worth--Arlington, TX | 4 (High: >8000) | 14.4% | 15.3% | 235,699 | 42.3 | 0.9% | 0.4 |
| Dayton, OH | 1 (Very Low: < 2000) | 24.7% | 35.1% | 58,739 | 682.3 | 10.5% | 71.6 |
| Dayton, OH | 2 (Low: 2000-4000) | 18.6% | 19.6% | 85,158 | 94.6 | 1.0% | 1.0 |
| Dayton, OH | 3 (Medium:4000-8000) | 9.9% | 11.7% | 25,995 | 9.5 | 1.8% | 0.2 |
| Dayton, OH | 4 (High: >8000) | 8.6% | 8.5% | 11,807 | 1.5 | 0.0% | 0.0 |
| Denver--Aurora, CO | 1 (Very Low: < 2000) | 17.2% | 28.1% | 70,305 | 847.8 | 10.8% | 91.7 |
| Denver--Aurora, CO | 2 (Low: 2000-4000) | 20.2% | 28.7% | 246,812 | 376.9 | 8.5% | 32.2 |
| Denver--Aurora, CO | 3 (Medium:4000-8000) | 17.1% | 19.2% | 184,327 | 91.3 | 2.1% | 1.9 |
| Denver--Aurora, CO | 4 (High: >8000) | 16.9% | 17.0% | 92,255 | 18.0 | 0.0% | 0.0 |
| Des Moines, IA | 1 (Very Low: < 2000) | 35.7% | 29.4% | 36,227 | 328.7 | 0.0% | 0.0 |
| Des Moines, IA | 2 (Low: 2000-4000) | 33.2% | 24.9% | 56,851 | 72.4 | 0.0% | 0.0 |
| Des Moines, IA | 3 (Medium:4000-8000) | 33.6% | 32.2% | 15,119 | 6.5 | 0.0% | 0.0 |
| Des Moines, IA | 4 (High: >8000) | 43.0% | 24.2% | 5,388 | 1.1 | 0.0% | 0.0 |
| Detroit, MI | 1 (Very Low: < 2000) | 27.7% | 49.7% | 163,196 | 2,282.8 | 21.9% | 500.5 |
| Detroit, MI | 2 (Low: 2000-4000) | 28.3% | 34.5% | 458,668 | 578.9 | 6.2% | 35.7 |
| Detroit, MI | 3 (Medium:4000-8000) | 18.3% | 18.2% | 256,693 | 90.5 | 0.0% | 0.0 |
| Detroit, MI | 4 (High: >8000) | 6.6% | 7.4% | 57,130 | 10.4 | 0.9% | 0.1 |
| El Paso, TX--NM | 1 (Very Low: < 2000) | 4.2% | 5.5% | 63,316 | 346.2 | 1.3% | 4.4 |
| El Paso, TX--NM | 2 (Low: 2000-4000) | 5.3% | 7.7% | 68,081 | 118.3 | 2.4% | 2.9 |
| El Paso, TX--NM | 3 (Medium:4000-8000) | 4.7% | 5.8% | 52,339 | 39.7 | 1.1% | 0.5 |
| El Paso, TX--NM | 4 (High: >8000) | 3.3% | 5.3% | 17,480 | 3.4 | 2.0% | 0.1 |
| Fayetteville, NC | 1 (Very Low: < 2000) | 52.3% | 52.1% | 60,807 | 414.4 | 0.0% | 0.0 |
| Fayetteville, NC | 2 (Low: 2000-4000) | 31.6% | 30.7% | 11,027 | 18.6 | 0.0% | 0.0 |
| Fayetteville, NC | 3 (Medium:4000-8000) | 17.5% | 5.8% | 4,284 | 2.4 | 0.0% | 0.0 |
| Fayetteville, NC | 4 (High: >8000) | 15.7% | 14.1% | 1,498 | 0.3 | 0.0% | 0.0 |
| Flint, MI | 1 (Very Low: < 2000) | 53.2% | 62.4% | 37,918 | 516.5 | 9.2% | 47.6 |
| Flint, MI | 2 (Low: 2000-4000) | 48.1% | 42.0% | 42,838 | 33.7 | 0.0% | 0.0 |
| Flint, MI | 3 (Medium:4000-8000) | 42.4% | 18.0% | 5,528 | 2.9 | 0.0% | 0.0 |
| Flint, MI | 4 (High: >8000) | 18.2% | 12.2% | 3,136 | 0.6 | 0.0% | 0.0 |
| Grand Rapids, MI | 1 (Very Low: < 2000) | 46.3% | 63.8% | 32,391 | 560.7 | 17.5% | 98.2 |
| Grand Rapids, MI | 2 (Low: 2000-4000) | 43.7% | 52.7% | 57,670 | 62.2 | 9.0% | 5.6 |
| Grand Rapids, MI | 3 (Medium:4000-8000) | 37.3% | 34.7% | 46,337 | 14.2 | 0.0% | 0.0 |
| Grand Rapids, MI | 4 (High: >8000) | 33.7% | 9.0% | 6,428 | 1.0 | 0.0% | 0.0 |
| Greenville, SC | 1 (Very Low: < 2000) | 38.3% | 39.8% | 66,836 | 716.0 | 1.5% | 10.7 |
| Greenville, SC | 2 (Low: 2000-4000) | 25.6% | 21.5% | 20,028 | 18.0 | 0.0% | 0.0 |
| Greenville, SC | 3 (Medium:4000-8000) | 20.8% | 17.9% | 10,407 | 3.5 | 0.0% | 0.0 |
| Greenville, SC | 4 (High: >8000) | 20.7% | 29.0% | 3,997 | 0.6 | 8.3% | 0.1 |
| Harrisburg, PA | 1 (Very Low: < 2000) | 38.6% | 48.4% | 26,926 | 548.9 | 9.8% | 54.0 |
| Harrisburg, PA | 2 (Low: 2000-4000) | 30.1% | 30.2% | 22,487 | 35.0 | 0.1% | 0.0 |
| Harrisburg, PA | 3 (Medium:4000-8000) | 28.4% | 18.9% | 32,700 | 10.8 | 0.0% | 0.0 |
| Harrisburg, PA | 4 (High: >8000) | 26.8% | 31.6% | 30,047 | 3.5 | 4.8% | 0.2 |
| Hartford, CT | 1 (Very Low: < 2000) | 37.7% | 60.7% | 33,214 | 1,118.8 | 23.0% | 257.8 |
| Hartford, CT | 2 (Low: 2000-4000) | 24.4% | 29.4% | 51,118 | 65.0 | 5.0% | 3.2 |
| Hartford, CT | 3 (Medium:4000-8000) | 17.4% | 17.2% | 84,035 | 23.8 | 0.0% | 0.0 |
| Hartford, CT | 4 (High: >8000) | 9.7% | 5.7% | 65,677 | 7.3 | 0.0% | 0.0 |
| Hickory, NC | 1 (Very Low: < 2000) | 50.8% | 60.6% | 46,070 | 613.2 | 9.9% | 60.4 |
| Hickory, NC | 2 (Low: 2000-4000) | 36.4% | 33.2% | 5,628 | 5.0 | 0.0% | 0.0 |
| Hickory, NC | 3 (Medium:4000-8000) | 30.4% | 37.4% | 1,393 | 0.6 | 7.0% | 0.0 |
| Hickory, NC | 4 (High: >8000) | 19.0% | 6.8% | 561 | 0.1 | 0.0% | 0.0 |
| Houston, TX | 1 (Very Low: < 2000) | 32.4% | 34.7% | 252,349 | 2,565.3 | 2.4% | 60.4 |
| Houston, TX | 2 (Low: 2000-4000) | 32.0% | 37.2% | 355,385 | 642.9 | 5.2% | 33.4 |
| Houston, TX | 3 (Medium:4000-8000) | 25.1% | 21.5% | 291,837 | 158.2 | 0.0% | 0.0 |
| Houston, TX | 4 (High: >8000) | 11.9% | 16.3% | 337,206 | 39.4 | 4.4% | 1.7 |
| Huntsville, AL | 1 (Very Low: < 2000) | 54.4% | 55.6% | 41,300 | 434.2 | 1.2% | 5.3 |
| Huntsville, AL | 2 (Low: 2000-4000) | 52.3% | 32.5% | 17,905 | 14.2 | 0.0% | 0.0 |
| Huntsville, AL | 3 (Medium:4000-8000) | 27.8% | 34.6% | 7,222 | 2.6 | 6.8% | 0.2 |
| Huntsville, AL | 4 (High: >8000) | 23.9% | 15.8% | 6,899 | 0.7 | 0.0% | 0.0 |
| Indianapolis, IN | 1 (Very Low: < 2000) | 28.3% | 35.7% | 129,908 | 1,379.6 | 7.4% | 101.7 |
| Indianapolis, IN | 2 (Low: 2000-4000) | 19.1% | 21.8% | 151,010 | 157.7 | 2.7% | 4.3 |
| Indianapolis, IN | 3 (Medium:4000-8000) | 11.5% | 7.4% | 64,474 | 25.7 | 0.0% | 0.0 |
| Indianapolis, IN | 4 (High: >8000) | 6.8% | 0.9% | 27,194 | 4.9 | 0.0% | 0.0 |
| Jackson, MS | 1 (Very Low: < 2000) | 70.2% | 56.9% | 47,940 | 484.8 | 0.0% | 0.0 |
| Jackson, MS | 2 (Low: 2000-4000) | 59.8% | 38.8% | 23,307 | 25.7 | 0.0% | 0.0 |
| Jackson, MS | 3 (Medium:4000-8000) | 59.4% | 21.4% | 8,147 | 3.6 | 0.0% | 0.0 |
| Jackson, MS | 4 (High: >8000) | 15.7% | 10.8% | 8,298 | 1.1 | 0.0% | 0.0 |
| Jacksonville, FL | 1 (Very Low: < 2000) | 47.9% | 50.5% | 134,517 | 1,072.7 | 2.6% | 27.5 |
| Jacksonville, FL | 2 (Low: 2000-4000) | 40.1% | 33.7% | 93,198 | 106.2 | 0.0% | 0.0 |
| Jacksonville, FL | 3 (Medium:4000-8000) | 24.2% | 19.1% | 23,698 | 12.3 | 0.0% | 0.0 |
| Jacksonville, FL | 4 (High: >8000) | 8.2% | 13.3% | 17,127 | 2.7 | 5.1% | 0.1 |
| Kansas City, MO--KS | 1 (Very Low: < 2000) | 42.5% | 46.9% | 164,895 | 1,152.8 | 4.4% | 50.8 |
| Kansas City, MO--KS | 2 (Low: 2000-4000) | 33.8% | 36.9% | 145,232 | 203.6 | 3.2% | 6.4 |
| Kansas City, MO--KS | 3 (Medium:4000-8000) | 20.5% | 17.0% | 55,839 | 22.6 | 0.0% | 0.0 |
| Kansas City, MO--KS | 4 (High: >8000) | 16.3% | 13.3% | 14,931 | 3.4 | 0.0% | 0.0 |
| Knoxville, TN | 1 (Very Low: < 2000) | 68.2% | 64.3% | 91,527 | 985.3 | 0.0% | 0.0 |
| Knoxville, TN | 2 (Low: 2000-4000) | 57.9% | 46.3% | 26,483 | 22.2 | 0.0% | 0.0 |
| Knoxville, TN | 3 (Medium:4000-8000) | 41.1% | 18.3% | 10,809 | 3.9 | 0.0% | 0.0 |
| Knoxville, TN | 4 (High: >8000) | 22.3% | 32.5% | 11,467 | 1.1 | 10.2% | 0.1 |
| Lancaster, PA | 1 (Very Low: < 2000) | 42.9% | 49.2% | 24,068 | 558.1 | 6.3% | 35.3 |
| Lancaster, PA | 2 (Low: 2000-4000) | 32.4% | 36.1% | 18,714 | 28.9 | 3.7% | 1.1 |
| Lancaster, PA | 3 (Medium:4000-8000) | 15.9% | 19.6% | 22,279 | 8.4 | 3.7% | 0.3 |
| Lancaster, PA | 4 (High: >8000) | 12.3% | 8.0% | 35,614 | 3.8 | 0.0% | 0.0 |
| Las Vegas--Henderson, NV | 1 (Very Low: < 2000) | 3.2% | 7.5% | 34,304 | 505.4 | 4.3% | 21.7 |
| Las Vegas--Henderson, NV | 2 (Low: 2000-4000) | 5.3% | 8.7% | 128,273 | 244.4 | 3.4% | 8.4 |
| Las Vegas--Henderson, NV | 3 (Medium:4000-8000) | 5.2% | 7.5% | 201,287 | 115.1 | 2.3% | 2.6 |
| Las Vegas--Henderson, NV | 4 (High: >8000) | 6.2% | 8.3% | 107,864 | 17.4 | 2.1% | 0.4 |
| Little Rock, AR | 1 (Very Low: < 2000) | 70.8% | 69.5% | 61,558 | 521.1 | 0.0% | 0.0 |
| Little Rock, AR | 2 (Low: 2000-4000) | 67.6% | 60.5% | 35,443 | 41.6 | 0.0% | 0.0 |
| Little Rock, AR | 3 (Medium:4000-8000) | 31.9% | 50.2% | 6,788 | 4.3 | 18.3% | 0.8 |
| Little Rock, AR | 4 (High: >8000) | 41.5% | 6.6% | 4,609 | 0.6 | 0.0% | 0.0 |
| Los Angeles--Long Beach--Anaheim, CA | 1 (Very Low: < 2000) | 11.1% | 33.1% | 62,667 | 1,528.2 | 22.0% | 335.6 |
| Los Angeles--Long Beach--Anaheim, CA | 2 (Low: 2000-4000) | 13.6% | 22.9% | 178,030 | 971.9 | 9.3% | 90.5 |
| Los Angeles--Long Beach--Anaheim, CA | 3 (Medium:4000-8000) | 14.7% | 19.1% | 852,249 | 703.5 | 4.4% | 30.9 |
| Los Angeles--Long Beach--Anaheim, CA | 4 (High: >8000) | 17.9% | 19.5% | 1,947,701 | 337.4 | 1.5% | 5.1 |
| Louisville/Jefferson County, KY--IN | 1 (Very Low: < 2000) | 35.0% | 46.9% | 84,083 | 947.4 | 11.9% | 112.4 |
| Louisville/Jefferson County, KY--IN | 2 (Low: 2000-4000) | 34.5% | 33.9% | 96,043 | 116.1 | 0.0% | 0.0 |
| Louisville/Jefferson County, KY--IN | 3 (Medium:4000-8000) | 27.1% | 26.2% | 45,227 | 18.0 | 0.0% | 0.0 |
| Louisville/Jefferson County, KY--IN | 4 (High: >8000) | 20.5% | 16.2% | 17,772 | 2.9 | 0.0% | 0.0 |
| McAllen, TX | 1 (Very Low: < 2000) | 5.8% | 7.3% | 96,100 | 743.8 | 1.5% | 11.3 |
| McAllen, TX | 2 (Low: 2000-4000) | 9.0% | 9.3% | 48,881 | 76.5 | 0.2% | 0.2 |
| McAllen, TX | 3 (Medium:4000-8000) | 10.1% | 7.9% | 28,170 | 23.4 | 0.0% | 0.0 |
| McAllen, TX | 4 (High: >8000) | 1.2% | 4.6% | 9,231 | 1.7 | 3.5% | 0.1 |
| Memphis, TN--MS--AR | 1 (Very Low: < 2000) | 55.6% | 65.0% | 105,589 | 890.0 | 9.3% | 83.1 |
| Memphis, TN--MS--AR | 2 (Low: 2000-4000) | 52.0% | 44.3% | 109,802 | 136.1 | 0.0% | 0.0 |
| Memphis, TN--MS--AR | 3 (Medium:4000-8000) | 38.3% | 35.0% | 22,716 | 13.9 | 0.0% | 0.0 |
| Memphis, TN--MS--AR | 4 (High: >8000) | 27.6% | 25.3% | 28,018 | 4.6 | 0.0% | 0.0 |
| Miami, FL | 1 (Very Low: < 2000) | 29.0% | 43.4% | 114,140 | 1,625.5 | 14.4% | 234.1 |
| Miami, FL | 2 (Low: 2000-4000) | 29.9% | 34.5% | 425,224 | 657.9 | 4.6% | 30.3 |
| Miami, FL | 3 (Medium:4000-8000) | 25.4% | 29.4% | 462,071 | 256.2 | 4.0% | 10.2 |
| Miami, FL | 4 (High: >8000) | 14.9% | 20.0% | 374,114 | 69.7 | 5.1% | 3.6 |
| Milwaukee, WI | 1 (Very Low: < 2000) | 29.5% | 55.1% | 18,518 | 1,024.7 | 25.7% | 263.0 |
| Milwaukee, WI | 2 (Low: 2000-4000) | 26.3% | 36.8% | 67,886 | 133.0 | 10.5% | 14.0 |
| Milwaukee, WI | 3 (Medium:4000-8000) | 21.3% | 16.2% | 162,752 | 60.4 | 0.0% | 0.0 |
| Milwaukee, WI | 4 (High: >8000) | 12.5% | 6.4% | 94,743 | 12.2 | 0.0% | 0.0 |
| Minneapolis--St. Paul, MN--WI | 1 (Very Low: < 2000) | 52.7% | 58.5% | 149,458 | 1,844.6 | 5.8% | 107.4 |
| Minneapolis--St. Paul, MN--WI | 2 (Low: 2000-4000) | 43.6% | 45.3% | 234,557 | 310.1 | 1.7% | 5.1 |
| Minneapolis--St. Paul, MN--WI | 3 (Medium:4000-8000) | 34.0% | 29.7% | 184,032 | 65.6 | 0.0% | 0.0 |
| Minneapolis--St. Paul, MN--WI | 4 (High: >8000) | 21.3% | 12.8% | 95,207 | 13.6 | 0.0% | 0.0 |
| Mobile, AL | 1 (Very Low: < 2000) | 63.0% | 62.1% | 54,299 | 465.4 | 0.0% | 0.0 |
| Mobile, AL | 2 (Low: 2000-4000) | 55.5% | 57.3% | 22,621 | 23.3 | 1.7% | 0.4 |
| Mobile, AL | 3 (Medium:4000-8000) | 31.3% | 40.6% | 3,857 | 2.3 | 9.3% | 0.2 |
| Mobile, AL | 4 (High: >8000) | 9.7% | 10.6% | 3,291 | 1.0 | 0.9% | 0.0 |
| Myrtle Beach--Socastee, SC--NC | 1 (Very Low: < 2000) | 41.6% | 42.5% | 37,564 | 398.8 | 0.8% | 3.4 |
| Myrtle Beach--Socastee, SC--NC | 2 (Low: 2000-4000) | 28.4% | 27.2% | 9,711 | 10.2 | 0.0% | 0.0 |
| Myrtle Beach--Socastee, SC--NC | 3 (Medium:4000-8000) | 14.0% | 14.6% | 4,971 | 1.7 | 0.6% | 0.0 |
| Myrtle Beach--Socastee, SC--NC | 4 (High: >8000) | 14.5% | 29.0% | 1,742 | 0.2 | 14.5% | 0.0 |
| Nashville-Davidson, TN | 1 (Very Low: < 2000) | 62.0% | 70.2% | 111,909 | 1,186.0 | 8.2% | 96.8 |
| Nashville-Davidson, TN | 2 (Low: 2000-4000) | 58.3% | 52.6% | 78,767 | 81.9 | 0.0% | 0.0 |
| Nashville-Davidson, TN | 3 (Medium:4000-8000) | 43.9% | 33.1% | 31,656 | 12.0 | 0.0% | 0.0 |
| Nashville-Davidson, TN | 4 (High: >8000) | 35.7% | 34.1% | 20,733 | 3.2 | 0.0% | 0.0 |
| New Haven, CT | 1 (Very Low: < 2000) | 40.5% | 65.3% | 16,963 | 645.3 | 24.8% | 159.8 |
| New Haven, CT | 2 (Low: 2000-4000) | 37.1% | 33.2% | 29,427 | 38.0 | 0.0% | 0.0 |
| New Haven, CT | 3 (Medium:4000-8000) | 20.3% | 20.3% | 43,517 | 15.9 | 0.1% | 0.0 |
| New Haven, CT | 4 (High: >8000) | 14.4% | 3.0% | 49,272 | 5.5 | 0.0% | 0.0 |
| New Orleans, LA | 1 (Very Low: < 2000) | 48.6% | 52.1% | 39,455 | 268.9 | 3.4% | 9.2 |
| New Orleans, LA | 2 (Low: 2000-4000) | 41.1% | 42.8% | 86,039 | 141.6 | 1.7% | 2.4 |
| New Orleans, LA | 3 (Medium:4000-8000) | 35.8% | 44.6% | 68,424 | 42.5 | 8.8% | 3.7 |
| New Orleans, LA | 4 (High: >8000) | 23.5% | 35.7% | 31,534 | 6.4 | 12.3% | 0.8 |
| New York--Newark, NY--NJ--CT | 1 (Very Low: < 2000) | 36.9% | 55.9% | 107,581 | 5,874.9 | 19.0% | 1,116.5 |
| New York--Newark, NY--NJ--CT | 2 (Low: 2000-4000) | 27.9% | 32.0% | 169,510 | 1,024.1 | 4.1% | 42.4 |
| New York--Newark, NY--NJ--CT | 3 (Medium:4000-8000) | 19.6% | 17.9% | 404,863 | 459.2 | 0.0% | 0.0 |
| New York--Newark, NY--NJ--CT | 4 (High: >8000) | 8.6% | 3.2% | 3,903,507 | 453.1 | 0.0% | 0.0 |
| Ogden--Layton, UT | 1 (Very Low: < 2000) | 13.2% | 23.1% | 39,591 | 370.4 | 9.9% | 36.5 |
| Ogden--Layton, UT | 2 (Low: 2000-4000) | 17.9% | 22.3% | 70,761 | 91.0 | 4.4% | 4.0 |
| Ogden--Layton, UT | 3 (Medium:4000-8000) | 16.4% | 18.4% | 22,870 | 9.7 | 2.0% | 0.2 |
| Ogden--Layton, UT | 4 (High: >8000) | 13.8% | 18.9% | 4,285 | 0.8 | 5.1% | 0.0 |
| Oklahoma City, OK | 1 (Very Low: < 2000) | 27.1% | 25.4% | 63,809 | 693.6 | 0.0% | 0.0 |
| Oklahoma City, OK | 2 (Low: 2000-4000) | 27.4% | 17.8% | 111,599 | 138.4 | 0.0% | 0.0 |
| Oklahoma City, OK | 3 (Medium:4000-8000) | 16.8% | 14.7% | 27,480 | 12.1 | 0.0% | 0.0 |
| Oklahoma City, OK | 4 (High: >8000) | 6.1% | 5.0% | 12,968 | 2.4 | 0.0% | 0.0 |
| Omaha, NE--IA | 1 (Very Low: < 2000) | 11.1% | 17.0% | 40,920 | 388.2 | 5.9% | 22.9 |
| Omaha, NE--IA | 2 (Low: 2000-4000) | 9.5% | 14.2% | 87,203 | 131.5 | 4.7% | 6.2 |
| Omaha, NE--IA | 3 (Medium:4000-8000) | 5.2% | 6.2% | 37,691 | 14.5 | 1.0% | 0.1 |
| Omaha, NE--IA | 4 (High: >8000) | 2.2% | 5.6% | 18,481 | 3.2 | 3.3% | 0.1 |
| Orlando, FL | 1 (Very Low: < 2000) | 46.7% | 51.9% | 127,824 | 1,051.0 | 5.1% | 54.1 |
| Orlando, FL | 2 (Low: 2000-4000) | 36.6% | 38.5% | 140,281 | 167.6 | 1.9% | 3.2 |
| Orlando, FL | 3 (Medium:4000-8000) | 30.2% | 29.5% | 54,237 | 25.7 | 0.0% | 0.0 |
| Orlando, FL | 4 (High: >8000) | 22.5% | 26.3% | 55,769 | 10.5 | 3.8% | 0.4 |
| Palm Bay--Melbourne, FL | 1 (Very Low: < 2000) | 44.2% | 35.6% | 85,376 | 498.4 | 0.0% | 0.0 |
| Palm Bay--Melbourne, FL | 2 (Low: 2000-4000) | 24.3% | 17.7% | 22,115 | 35.2 | 0.0% | 0.0 |
| Palm Bay--Melbourne, FL | 3 (Medium:4000-8000) | 16.0% | 18.7% | 4,604 | 2.4 | 2.7% | 0.1 |
| Palm Bay--Melbourne, FL | 4 (High: >8000) | 21.6% | 10.1% | 2,964 | 0.7 | 0.0% | 0.0 |
| Pensacola, FL--AL | 1 (Very Low: < 2000) | 45.5% | 45.5% | 54,540 | 490.8 | 0.0% | 0.0 |
| Pensacola, FL--AL | 2 (Low: 2000-4000) | 33.8% | 27.5% | 14,152 | 18.2 | 0.0% | 0.0 |
| Pensacola, FL--AL | 3 (Medium:4000-8000) | 6.4% | 21.2% | 2,205 | 1.0 | 14.9% | 0.1 |
| Pensacola, FL--AL | 4 (High: >8000) | 1.8% | 10.7% | 14,032 | 1.4 | 8.9% | 0.1 |
| Philadelphia, PA--NJ--DE--MD | 1 (Very Low: < 2000) | 32.9% | 47.9% | 81,233 | 3,842.2 | 15.0% | 575.4 |
| Philadelphia, PA--NJ--DE--MD | 2 (Low: 2000-4000) | 20.8% | 28.9% | 141,052 | 427.8 | 8.1% | 34.8 |
| Philadelphia, PA--NJ--DE--MD | 3 (Medium:4000-8000) | 9.5% | 14.7% | 250,894 | 144.1 | 5.1% | 7.4 |
| Philadelphia, PA--NJ--DE--MD | 4 (High: >8000) | 2.3% | 2.4% | 888,500 | 107.0 | 0.1% | 0.1 |
| Phoenix--Mesa, AZ | 1 (Very Low: < 2000) | 5.9% | 10.8% | 105,545 | 1,749.2 | 4.8% | 84.2 |
| Phoenix--Mesa, AZ | 2 (Low: 2000-4000) | 6.9% | 10.5% | 329,251 | 534.8 | 3.6% | 19.4 |
| Phoenix--Mesa, AZ | 3 (Medium:4000-8000) | 6.6% | 11.0% | 334,023 | 124.6 | 4.4% | 5.5 |
| Phoenix--Mesa, AZ | 4 (High: >8000) | 8.7% | 11.9% | 138,844 | 20.6 | 3.3% | 0.7 |
| Pittsburgh, PA | 1 (Very Low: < 2000) | 36.7% | 43.6% | 124,499 | 1,911.2 | 7.0% | 133.0 |
| Pittsburgh, PA | 2 (Low: 2000-4000) | 20.5% | 33.2% | 130,387 | 149.4 | 12.7% | 19.0 |
| Pittsburgh, PA | 3 (Medium:4000-8000) | 16.0% | 25.3% | 125,282 | 51.6 | 9.3% | 4.8 |
| Pittsburgh, PA | 4 (High: >8000) | 15.8% | 20.3% | 54,056 | 8.5 | 4.5% | 0.4 |
| Port St. Lucie, FL | 1 (Very Low: < 2000) | 40.7% | 50.5% | 60,944 | 450.4 | 9.7% | 43.8 |
| Port St. Lucie, FL | 2 (Low: 2000-4000) | 31.0% | 26.3% | 24,028 | 22.1 | 0.0% | 0.0 |
| Port St. Lucie, FL | 3 (Medium:4000-8000) | 26.6% | 25.8% | 6,096 | 2.1 | 0.0% | 0.0 |
| Port St. Lucie, FL | 4 (High: >8000) | 5.8% | 13.5% | 4,267 | 0.4 | 7.7% | 0.0 |
| Portland, OR--WA | 1 (Very Low: < 2000) | 23.4% | 53.7% | 80,543 | 804.1 | 30.3% | 243.9 |
| Portland, OR--WA | 2 (Low: 2000-4000) | 21.7% | 34.1% | 204,089 | 281.6 | 12.4% | 34.9 |
| Portland, OR--WA | 3 (Medium:4000-8000) | 18.8% | 22.4% | 128,900 | 70.0 | 3.6% | 2.5 |
| Portland, OR--WA | 4 (High: >8000) | 11.8% | 5.8% | 50,141 | 10.2 | 0.0% | 0.0 |
| Poughkeepsie--Newburgh, NY--NJ | 1 (Very Low: < 2000) | 49.1% | 56.9% | 24,121 | 753.9 | 7.8% | 59.0 |
| Poughkeepsie--Newburgh, NY--NJ | 2 (Low: 2000-4000) | 26.3% | 28.3% | 15,974 | 21.2 | 2.0% | 0.4 |
| Poughkeepsie--Newburgh, NY--NJ | 3 (Medium:4000-8000) | 20.4% | 21.6% | 28,119 | 7.8 | 1.2% | 0.1 |
| Poughkeepsie--Newburgh, NY--NJ | 4 (High: >8000) | 13.2% | 0.0% | 39,039 | 3.6 | 0.0% | 0.0 |
| Providence, RI--MA | 1 (Very Low: < 2000) | 33.9% | 55.1% | 24,835 | 1,117.9 | 21.2% | 237.0 |
| Providence, RI--MA | 2 (Low: 2000-4000) | 21.6% | 22.5% | 38,876 | 96.1 | 0.9% | 0.9 |
| Providence, RI--MA | 3 (Medium:4000-8000) | 17.2% | 10.4% | 100,742 | 40.7 | 0.0% | 0.0 |
| Providence, RI--MA | 4 (High: >8000) | 15.0% | 11.8% | 132,131 | 14.6 | 0.0% | 0.0 |
| Raleigh, NC | 1 (Very Low: < 2000) | 60.5% | 70.8% | 103,629 | 1,068.0 | 10.3% | 110.4 |
| Raleigh, NC | 2 (Low: 2000-4000) | 51.9% | 51.0% | 74,370 | 86.9 | 0.0% | 0.0 |
| Raleigh, NC | 3 (Medium:4000-8000) | 39.6% | 36.7% | 31,528 | 13.4 | 0.0% | 0.0 |
| Raleigh, NC | 4 (High: >8000) | 37.9% | 13.2% | 13,050 | 2.4 | 0.0% | 0.0 |
| Richmond, VA | 1 (Very Low: < 2000) | 56.1% | 63.2% | 107,563 | 1,004.3 | 7.2% | 72.0 |
| Richmond, VA | 2 (Low: 2000-4000) | 42.8% | 36.9% | 65,123 | 65.6 | 0.0% | 0.0 |
| Richmond, VA | 3 (Medium:4000-8000) | 28.3% | 22.7% | 34,623 | 16.5 | 0.0% | 0.0 |
| Richmond, VA | 4 (High: >8000) | 9.9% | 10.6% | 31,078 | 4.4 | 0.6% | 0.0 |
| Riverside--San Bernardino, CA | 1 (Very Low: < 2000) | 16.7% | 32.2% | 86,223 | 784.6 | 15.4% | 121.2 |
| Riverside--San Bernardino, CA | 2 (Low: 2000-4000) | 20.3% | 29.4% | 143,479 | 260.4 | 9.1% | 23.7 |
| Riverside--San Bernardino, CA | 3 (Medium:4000-8000) | 21.6% | 25.1% | 200,754 | 111.0 | 3.5% | 3.9 |
| Riverside--San Bernardino, CA | 4 (High: >8000) | 21.8% | 31.4% | 53,488 | 9.8 | 9.6% | 0.9 |
| Rochester, NY | 1 (Very Low: < 2000) | 22.7% | 38.9% | 34,915 | 679.9 | 16.2% | 110.2 |
| Rochester, NY | 2 (Low: 2000-4000) | 16.9% | 21.2% | 52,558 | 56.4 | 4.3% | 2.4 |
| Rochester, NY | 3 (Medium:4000-8000) | 15.5% | 14.0% | 85,499 | 25.2 | 0.0% | 0.0 |
| Rochester, NY | 4 (High: >8000) | 11.8% | 10.6% | 11,006 | 2.1 | 0.0% | 0.0 |
| Sacramento, CA | 1 (Very Low: < 2000) | 16.0% | 31.6% | 45,268 | 676.0 | 15.6% | 105.6 |
| Sacramento, CA | 2 (Low: 2000-4000) | 20.9% | 26.1% | 168,691 | 254.2 | 5.2% | 13.2 |
| Sacramento, CA | 3 (Medium:4000-8000) | 20.7% | 22.7% | 171,905 | 87.1 | 1.9% | 1.7 |
| Sacramento, CA | 4 (High: >8000) | 20.9% | 37.1% | 44,624 | 7.9 | 16.2% | 1.3 |
| Salt Lake City--West Valley City, UT | 1 (Very Low: < 2000) | 14.8% | 20.5% | 37,434 | 352.7 | 5.7% | 20.0 |
| Salt Lake City--West Valley City, UT | 2 (Low: 2000-4000) | 21.2% | 21.9% | 109,794 | 173.7 | 0.7% | 1.2 |
| Salt Lake City--West Valley City, UT | 3 (Medium:4000-8000) | 19.9% | 22.8% | 75,974 | 36.2 | 2.9% | 1.0 |
| Salt Lake City--West Valley City, UT | 4 (High: >8000) | 16.8% | 23.6% | 32,739 | 5.6 | 6.8% | 0.4 |
| San Antonio, TX | 1 (Very Low: < 2000) | 25.4% | 43.2% | 88,915 | 981.1 | 17.8% | 175.0 |
| San Antonio, TX | 2 (Low: 2000-4000) | 28.8% | 34.4% | 217,753 | 265.0 | 5.6% | 14.9 |
| San Antonio, TX | 3 (Medium:4000-8000) | 25.6% | 24.3% | 115,070 | 62.0 | 0.0% | 0.0 |
| San Antonio, TX | 4 (High: >8000) | 12.1% | 18.2% | 20,870 | 4.1 | 6.1% | 0.3 |
| San Diego, CA | 1 (Very Low: < 2000) | 5.1% | 16.3% | 46,765 | 1,092.7 | 11.2% | 121.9 |
| San Diego, CA | 2 (Low: 2000-4000) | 3.4% | 12.5% | 134,077 | 301.8 | 9.1% | 27.5 |
| San Diego, CA | 3 (Medium:4000-8000) | 3.4% | 9.3% | 272,330 | 145.8 | 5.9% | 8.5 |
| San Diego, CA | 4 (High: >8000) | 3.0% | 4.8% | 285,495 | 46.6 | 1.8% | 0.8 |
| San Francisco--Oakland, CA | 1 (Very Low: < 2000) | 6.2% | 43.7% | 32,019 | 555.7 | 37.5% | 208.3 |
| San Francisco--Oakland, CA | 2 (Low: 2000-4000) | 3.7% | 21.9% | 79,585 | 222.0 | 18.2% | 40.4 |
| San Francisco--Oakland, CA | 3 (Medium:4000-8000) | 2.2% | 9.6% | 309,876 | 182.3 | 7.4% | 13.5 |
| San Francisco--Oakland, CA | 4 (High: >8000) | 2.1% | 3.5% | 400,529 | 93.1 | 1.3% | 1.2 |
| San Jose, CA | 1 (Very Low: < 2000) | 3.4% | 21.8% | 19,032 | 264.9 | 18.3% | 48.5 |
| San Jose, CA | 2 (Low: 2000-4000) | 2.9% | 9.7% | 51,912 | 188.1 | 6.8% | 12.8 |
| San Jose, CA | 3 (Medium:4000-8000) | 2.4% | 7.3% | 190,030 | 100.4 | 4.9% | 4.9 |
| San Jose, CA | 4 (High: >8000) | 3.3% | 7.3% | 153,983 | 30.6 | 4.1% | 1.3 |
| Sarasota--Bradenton, FL | 1 (Very Low: < 2000) | 26.9% | 35.5% | 71,524 | 694.4 | 8.6% | 59.5 |
| Sarasota--Bradenton, FL | 2 (Low: 2000-4000) | 28.5% | 22.2% | 64,156 | 55.2 | 0.0% | 0.0 |
| Sarasota--Bradenton, FL | 3 (Medium:4000-8000) | 20.3% | 18.1% | 19,915 | 7.1 | 0.0% | 0.0 |
| Sarasota--Bradenton, FL | 4 (High: >8000) | 18.2% | 16.5% | 8,006 | 1.6 | 0.0% | 0.0 |
| Seattle, WA | 1 (Very Low: < 2000) | 38.0% | 56.9% | 225,195 | 1,754.8 | 19.0% | 332.7 |
| Seattle, WA | 2 (Low: 2000-4000) | 23.9% | 36.4% | 288,288 | 400.3 | 12.5% | 50.1 |
| Seattle, WA | 3 (Medium:4000-8000) | 24.5% | 29.3% | 154,905 | 91.5 | 4.8% | 4.4 |
| Seattle, WA | 4 (High: >8000) | 18.5% | 14.0% | 100,994 | 22.3 | 0.0% | 0.0 |
| Springfield, MA--CT | 1 (Very Low: < 2000) | 44.3% | 57.6% | 15,927 | 738.2 | 13.3% | 98.2 |
| Springfield, MA--CT | 2 (Low: 2000-4000) | 27.2% | 28.5% | 30,487 | 42.7 | 1.3% | 0.5 |
| Springfield, MA--CT | 3 (Medium:4000-8000) | 18.1% | 23.2% | 53,196 | 17.8 | 5.1% | 0.9 |
| Springfield, MA--CT | 4 (High: >8000) | 11.9% | 13.5% | 57,134 | 5.6 | 1.7% | 0.1 |
| St. Louis, MO--IL | 1 (Very Low: < 2000) | 33.2% | 43.5% | 168,827 | 1,638.9 | 10.3% | 168.7 |
| St. Louis, MO--IL | 2 (Low: 2000-4000) | 34.0% | 39.9% | 223,724 | 261.8 | 5.9% | 15.5 |
| St. Louis, MO--IL | 3 (Medium:4000-8000) | 22.3% | 37.6% | 96,944 | 42.4 | 15.3% | 6.5 |
| St. Louis, MO--IL | 4 (High: >8000) | 16.6% | 28.2% | 48,135 | 8.8 | 11.5% | 1.0 |
| Syracuse, NY | 1 (Very Low: < 2000) | 36.3% | 44.4% | 12,935 | 380.0 | 8.2% | 31.1 |
| Syracuse, NY | 2 (Low: 2000-4000) | 32.0% | 33.7% | 29,745 | 43.4 | 1.7% | 0.7 |
| Syracuse, NY | 3 (Medium:4000-8000) | 23.4% | 24.9% | 42,814 | 12.7 | 1.5% | 0.2 |
| Syracuse, NY | 4 (High: >8000) | 10.5% | 8.9% | 18,565 | 2.1 | 0.0% | 0.0 |
| Tampa--St. Petersburg, FL | 1 (Very Low: < 2000) | 44.1% | 45.8% | 201,131 | 1,764.8 | 1.7% | 30.4 |
| Tampa--St. Petersburg, FL | 2 (Low: 2000-4000) | 28.6% | 27.2% | 259,977 | 327.9 | 0.0% | 0.0 |
| Tampa--St. Petersburg, FL | 3 (Medium:4000-8000) | 24.9% | 19.7% | 88,560 | 48.1 | 0.0% | 0.0 |
| Tampa--St. Petersburg, FL | 4 (High: >8000) | 14.1% | 12.1% | 58,498 | 10.7 | 0.0% | 0.0 |
| Toledo, OH--MI | 1 (Very Low: < 2000) | 18.4% | 29.9% | 25,056 | 435.5 | 11.5% | 50.1 |
| Toledo, OH--MI | 2 (Low: 2000-4000) | 13.2% | 21.1% | 59,652 | 64.9 | 7.9% | 5.1 |
| Toledo, OH--MI | 3 (Medium:4000-8000) | 10.3% | 10.2% | 36,345 | 13.2 | 0.0% | 0.0 |
| Toledo, OH--MI | 4 (High: >8000) | 5.0% | 10.1% | 6,286 | 0.7 | 5.2% | 0.0 |
| Tucson, AZ | 1 (Very Low: < 2000) | 2.9% | 5.6% | 40,754 | 649.7 | 2.7% | 17.5 |
| Tucson, AZ | 2 (Low: 2000-4000) | 3.4% | 7.1% | 91,826 | 114.9 | 3.7% | 4.3 |
| Tucson, AZ | 3 (Medium:4000-8000) | 3.4% | 5.7% | 56,289 | 22.8 | 2.3% | 0.5 |
| Tucson, AZ | 4 (High: >8000) | 5.2% | 6.2% | 20,933 | 3.1 | 1.1% | 0.0 |
| Tulsa, OK | 1 (Very Low: < 2000) | 11.1% | 14.0% | 65,911 | 634.7 | 2.9% | 18.4 |
| Tulsa, OK | 2 (Low: 2000-4000) | 10.6% | 9.3% | 67,578 | 88.3 | 0.0% | 0.0 |
| Tulsa, OK | 3 (Medium:4000-8000) | 3.7% | 12.1% | 22,721 | 9.8 | 8.3% | 0.8 |
| Tulsa, OK | 4 (High: >8000) | 5.4% | 4.9% | 7,374 | 1.8 | 0.0% | 0.0 |
| Virginia Beach, VA | 1 (Very Low: < 2000) | 40.1% | 44.9% | 79,859 | 842.5 | 4.8% | 40.0 |
| Virginia Beach, VA | 2 (Low: 2000-4000) | 31.7% | 23.4% | 131,729 | 179.9 | 0.0% | 0.0 |
| Virginia Beach, VA | 3 (Medium:4000-8000) | 23.2% | 15.5% | 94,286 | 41.5 | 0.0% | 0.0 |
| Virginia Beach, VA | 4 (High: >8000) | 13.8% | 9.6% | 50,148 | 8.1 | 0.0% | 0.0 |
| Washington, DC--VA--MD | 1 (Very Low: < 2000) | 53.7% | 61.5% | 165,986 | 2,236.5 | 7.8% | 173.7 |
| Washington, DC--VA--MD | 2 (Low: 2000-4000) | 43.1% | 47.1% | 268,800 | 445.1 | 4.0% | 18.0 |
| Washington, DC--VA--MD | 3 (Medium:4000-8000) | 33.8% | 27.9% | 327,052 | 153.0 | 0.0% | 0.0 |
| Washington, DC--VA--MD | 4 (High: >8000) | 21.8% | 18.4% | 384,163 | 73.1 | 0.0% | 0.0 |
| Wichita, KS | 1 (Very Low: < 2000) | 27.4% | 35.0% | 37,378 | 354.5 | 7.6% | 27.0 |
| Wichita, KS | 2 (Low: 2000-4000) | 31.5% | 34.8% | 61,263 | 75.0 | 3.3% | 2.5 |
| Wichita, KS | 3 (Medium:4000-8000) | 26.6% | 24.3% | 14,949 | 5.5 | 0.0% | 0.0 |
| Wichita, KS | 4 (High: >8000) | 13.2% | 13.5% | 4,624 | 0.6 | 0.3% | 0.0 |
| Winston-Salem, NC | 1 (Very Low: < 2000) | 61.6% | 66.9% | 63,167 | 738.2 | 5.4% | 39.7 |
| Winston-Salem, NC | 2 (Low: 2000-4000) | 53.8% | 55.4% | 19,955 | 19.1 | 1.6% | 0.3 |
| Winston-Salem, NC | 3 (Medium:4000-8000) | 36.9% | 21.4% | 9,649 | 2.9 | 0.0% | 0.0 |
| Winston-Salem, NC | 4 (High: >8000) | 34.0% | 12.0% | 5,260 | 0.7 | 0.0% | 0.0 |
| Worcester, MA--CT | 1 (Very Low: < 2000) | 55.3% | 63.8% | 22,099 | 688.1 | 8.5% | 58.8 |
| Worcester, MA--CT | 2 (Low: 2000-4000) | 35.0% | 30.3% | 22,279 | 30.8 | 0.0% | 0.0 |
| Worcester, MA--CT | 3 (Medium:4000-8000) | 19.7% | 20.7% | 35,924 | 10.2 | 1.0% | 0.1 |
| Worcester, MA--CT | 4 (High: >8000) | 12.7% | 7.4% | 42,372 | 4.5 | 0.0% | 0.0 |
| Youngstown, OH--PA | 1 (Very Low: < 2000) | 18.0% | 29.4% | 53,263 | 516.1 | 11.4% | 58.9 |
| Youngstown, OH--PA | 2 (Low: 2000-4000) | 10.0% | 10.7% | 37,121 | 34.8 | 0.7% | 0.2 |
| Youngstown, OH--PA | 3 (Medium:4000-8000) | 2.8% | 7.4% | 4,650 | 2.2 | 4.6% | 0.1 |
| Youngstown, OH--PA | 4 (High: >8000) | 1.1% | 24.4% | 2,090 | 0.2 | 23.4% | 0.0 |
